# A knowledge-based multivariate statistical method for examining gene-brain-behavioral/cognitive relationships: Imaging genetics generalized structured component analysis

**DOI:** 10.1371/journal.pone.0247592

**Published:** 2021-03-10

**Authors:** Heungsun Hwang, Gyeongcheol Cho, Min Jin Jin, Ji Hoon Ryoo, Younyoung Choi, Seung Hwan Lee

**Affiliations:** 1 Department of Psychology, McGill University, Montreal, Quebec, Canada; 2 Institute of Liberal Education, Kongju National University, Gongju, Korea; 3 Department of Education, Yonsei University, Seoul, Korea; 4 Department of Counseling Psychology, Hanyang Cyber University, Seoul, Korea; 5 Department of Psychiatry, Inje University Ilsan-Paik Hospital and Inje University, Goyang, Korea; University of Modena and Reggio Emilia, ITALY

## Abstract

With advances in neuroimaging and genetics, imaging genetics is a naturally emerging field that combines genetic and neuroimaging data with behavioral or cognitive outcomes to examine genetic influence on altered brain functions associated with behavioral or cognitive variation. We propose a statistical approach, termed imaging genetics generalized structured component analysis (IG-GSCA), which allows researchers to investigate such gene-brain-behavior/cognitive associations, taking into account well-documented biological characteristics (e.g., genetic pathways, gene-environment interactions, etc.) and methodological complexities (e.g., multicollinearity) in imaging genetic studies. We begin by describing the conceptual and technical underpinnings of IG-GSCA. We then apply the approach for investigating how nine depression-related genes and their interactions with an environmental variable (experience of potentially traumatic events) influence the thickness variations of 53 brain regions, which in turn affect depression severity in a sample of Korean participants. Our analysis shows that a dopamine receptor gene and an interaction between a serotonin transporter gene and the environment variable have statistically significant effects on a few brain regions’ variations that have statistically significant negative impacts on depression severity. These relationships are largely supported by previous studies. We also conduct a simulation study to safeguard whether IG-GSCA can recover parameters as expected in a similar situation.

## Introduction

Imaging genetics is a rapidly emerging field that integrates genetic and neuroimaging data with behavioral or cognitive outcomes to examine genetic influence on the variation of brain function, which is in turn associated with behavioral or cognitive variation [1]. This field has made remarkable progress in recent years [2], showing its potential for studying disease- or task-specific “gene-brain-behavior/cognition (G-B-B/C)” relationships. For example, variation in the Apolipoprotein E (APOE) gene was associated with altered activity in brain regions, such as the hippocampus, parietal and prefrontal cortex, during a memory task [3]. A functional variation in the catechol-O-methyltransferase (COMT) gene was related to differential brain activity in the dorsolateral prefrontal cortex, anterior cingulate cortex, and parietal cortex during cognitive control tasks [4].

Imaging genetic studies have increasingly involved a number of genotypes, such as single nucleotide polymorphisms (SNPs), and a number of brain-based phenotypes, such as voxel-level variations (shortly voxels hereafter), in accordance with accumulated evidence that multiple genotypes can be associated with a single phenotype and a single genotype can be associated with multiple phenotypes [5]. Thus, multivariate techniques have been main statistical tools for imaging genetic studies [6, 7], including canonical correlation analysis [8], partial least squares [9], reduced-rank regression [10], and independent component analysis [11, 12]. These techniques generally aim to obtain (low-dimensional) linear combinations or components of genetic and imaging data and examine the associations between the resultant genetic and imaging components.

Despite their usefulness, the scope and flexibility of the conventional multivariate techniques are limited in several ways. First, they do not explicitly account for various well-documented biological characteristics, such as genetic and molecular pathways (e.g., which SNPs occur in which genes), while extracting genetic and imaging components. As a result, the extracted components are often difficult to interpret, lacking direct biological meaning [6]. Second, they largely remain descriptive in nature, focusing on how genetic and imaging components are correlated to each other. This makes it difficult to statistically examine the influence of a component on another (e.g., which genetic components have effects on which imaging components and how the effects look like). Third, although in principle it is possible to extend the multivariate techniques to the analysis of more than two datasets, they have typically been applied to genetic and imaging datasets only to extract their components and associate them. Subsequently, the extracted genetic and/or imaging components are used to predict behavioral or cognitive outcomes through the adoption of regression analysis or machine learning algorithms [13]. This sequential or two-step approach does not guarantee that the extracted genetic and imaging components are optimal for predicting behavioral or cognitive phenotypes because they are obtained separately without considering prediction of such phenotypes. It would be more desirable to develop a unified framework for incorporating all genetic, imaging, and behavioral/cognitive phenotypes into analyses simultaneously, so that genetic and imaging components are extracted to be highly associated with each other as well as to predict behavioral/cognitive phenotypes well. To address these limitations, it would be necessary to develop a unified and path-analytic approach for specifying and testing more biologically plausible G-B-B/C relationships.

In this paper, we propose a general multivariate approach, termed imaging genetics generalized structured component analysis (IG-GSCA), for such unified analyses of path-analytic relationships among all the three sources of data (genetic, imaging, and behavioral/cognitive) in a more biologically meaningful manner. As will be discussed in more detail in Section 2, IG-GSCA allows researchers to specify and examine various biologically plausible G-B-B/C relationships based on knowledge accumulated from previous studies, for example, in genome-wide whole brain association [14] and connectivity analysis [15, 16].

As the name denotes, this approach is methodologically built on generalized structured component analysis (GSCA) [17, 18] that is a multivariate method for modeling and testing path-analytic relationships between observed variables and components thereof based on prior knowledge or theory. GSCA is well-suited to our data analytic purposes for several reasons. First, in imaging genetics, genetic and brain phenotypes, such as SNPs and voxels, represent observed measurements at specific locations in the genome and brain, indicating that a set of SNPs or voxels constitutes a gene or brain region. That is, in a statistical model, a gene or brain region may be regarded as a component of SNPs and voxels [19–21]. GSCA can be used to obtain such genetic and imaging components based on prior biological knowledge (e.g., which SNPs occur in which genes, or which voxels form which brain regions). Also, GSCA provides the unique individual scores of genetic and imaging components, which may represent gene- or brain-level scores of individuals associated with a specific behavioral or cognitive outcome. The provision of these individual scores may have important empirical implications. For example, clinicians may use the scores as proxies for individual gene- or brain-level vulnerabilities associated with risk for chronic diseases. Moreover, GSCA is less likely to suffer from non-convergence in small samples, complex models, and/or in the presence of multicollinearity [18], which are not uncommon in imaging genetic studies [22]. In addition, recent extensions of GSCA can be very useful for imaging genetic studies. For example, imaging genetic studies can often involve the specification of interaction terms (e.g., gene-gene interactions, gene-environment interactions, etc.). GSCA has been extended to incorporate various component interaction terms effectively [23]. Moreover, specifying a number of genes and/or brain regions as well as their potential interactions simultaneously is likely to lead to the issue of multicollinearity. GSCA has been combined with regularization to address the multicollinearity issue [18, 20, 24].

GSCA has been applied to examine the directional relationships between SNPs, genes, and behavioral phenotypes [20, 21]. It also has been used for examining directional relationships among brain regions [19, 25]. However, GSCA has never been employed for connecting genetic, imaging, and behavioral/cognitive phenotypes simultaneously. Furthermore, we integrate the aforementioned extensions of GSCA, such as testing interaction effects and regularization, for more efficient model specification and testing of the directional associations among the three data sources. Thus, IG-GSCA is a GSCA method tailored for the path-analytic analysis of imaging genetic data in a unified manner.

Owing to its generality and flexibility, structural equation modeling (SEM) [26, 27] can also be considered for such knowledge-based path-analytic analyses of imaging genetic data. Nonetheless, SEM may be less suitable for these analyses than IG-GSCA for several reasons. Most notably, SEM will specify a gene or brain region as a (common) factor that explains the covariation of SNPs or voxels only [28, 29], under the assumption that a gene or brain region exists independently of SNPs or voxels [30]. This indicates that assigning different SNPs or voxels to a gene or brain region should not change the gene’s or brain region’s meaning [31], which does not appear biologically plausible. As stated earlier, instead, it seems more reasonable to specify a gene or brain region as a weighted composite or biological cluster of SNPs or voxels, as postulated in IG-GSCA. However, this way of specifying a gene or brain region is not compatible with SEM, leading to identification problems in general [32]. Moreover, SEM cannot provide unique individual gene- or brain-level scores because of the factor score indeterminacy problem [33, 34]. Furthermore, it suffers from non-convergence particularly in small samples, complex models, and/or in the presence of multicollinearity [35, 36].

A few studies used SEM for associating genetic and imaging data with behavioral or cognitive variables [28, 37]. However, they applied a series of SEM or SEM and other statistical methods (e.g., regression) sequentially to examine the associations among these data, regardless of whether SEM is a suitable method for the studies. Conversely, as noted earlier, IG-GSCA is a unified statistical framework for researchers to be able to simultaneously associate genetic, imaging, and behavioral/cognitive data in a biologically plausible fashion.

The remainder of the paper proceeds as follows. We begin by discussing both conceptual and technical underpinnings of IG-GSCA, including its model specification and parameter estimation. We then apply IG-GSCA to real imaging genetics data collected from a sample of Korean participants in order to investigate the effects of gene-level variations on the thickness differences of brain regions, which in turn influence depression severity. We also conduct a simulation study to safeguard whether IG-GSCA performs as expected. We consider a model similar to the one specified in the real data analysis and examine IG-GSCA’s parameter recovery under different sample sizes. Lastly, we summarize the implications of IG-GSCA and discuss directions for future research.

## Method

### Model specification

It is crucial to build a model bridging all three main constituents of imaging genetics (i.e., genetic, brain, and behavioral/cognitive phenotypes) in a biologically plausible manner based on knowledge accumulated from previous literature or researchers’ hypotheses. In model specification, we should begin with the fundamental premise of imaging genetics that brain-based phenotypes serve as intermediate phenotypes between genotypes and behavioral/cognitive phenotypes, indicating that the influence of genetic variation on behaviour/cognition is mediated through brain phenotypes (i.e., indirect effects of genetic variation). Genome-wide whole brain association studies can be used to obtain information about genotypes that are associated with brain phenotypes relevant to specific behavioral or cognitive variation.

We can also consider various characteristics of each constituent. For example, it may be reasonable to assume from genetic studies that several SNPs often occur in a gene, rather than a single SNP per gene. A substantial amount of information on genetic pathways has already been gathered for researchers to specify which SNPs are linked to which genes in different diseases [14]. As discussed earlier, SNPs can be considered observed variables, whereas a gene can be a weighted sum of SNPs (i.e., a component). It is also known that multiple genes can be associated with a single phenotype (polygenicity); a single gene can be involved in multiple phenotypes (pleiotropy); and the effect of one gene can be modified by another gene, indicating gene-gene interactions (epistasis) [38].

In imaging studies, it is well recognized that a particular behavioral or cognitive task is associated with neural networks of multiple brain regions, rather than isolated brain regions [15]. Connectivity analysis can describe relationships between brain regions within a network [39]. There are two different approaches to connectivity analysis–functional vs. effective connectivity [16]. Functional connectivity analysis generally focuses on an inter-correlational pattern or inter-regional coupling between brain regions (e.g., activities in brain region A correlate with those in brain region B). It can offer insight into correlations between different brain regions but is limited in that it does not account for directionality between interacting regions. Conversely, effective connectivity analysis focuses on directional relationships between brain regions selected based on a hypothesis or prior knowledge about their importance in completing a task (e.g., activities in region A exert influence on those in region B). This approach can be used to better explain functional integration within a distributed neural system, allowing quantifications and stronger inferences of directed connections of different brain region activities [15]. Thus, it may be more desirable to explicitly incorporate directional neural network information given by previous effective connectivity studies. Furthermore, we can include more than one behavioral/cognitive phenotype at the same time to consider their potential correlations as well as the effects of genotypes and/or brain-based phenotypes on multiple behavioral/cognitive phenotypes.

In IG-GSCA, we incorporate such theoretical considerations into sets of mathematical equations, also called sub-models. Specifically, as in GSCA, it will involve three sub-models–weighted relation, measurement, and structural. The weighed relation model is used to explicitly define a component as a weighted sum of observed variables. The measurement model specifies the relationships between observed and components, whereas the structural model is to express the relationships between components.

For simplicity, hereafter, let us assume that all genetic observed variables indicate SNPs and imaging observed variables are called voxels, whereas all genetic and imaging components are genes and brain regions, respectively. Let **z** denote a *J* by 1 vector of all observed variables, including SNPs, voxels, and behavioral/cognitive phenotypes. Let **γ** denote a *P* by 1 vector of all components, including genes, brain regions, and behavioral/cognitive traits. We assume that all observed variables and components are standardized to have zero means and unit variances.

The weighted relation model is generally written as follows.

γ=Wz, (1)

where **W** is a *P* by *J* matrix of weights assigned to *J* observed variables. This sub-model is unique to IG-GSCA (or GSCA), which is distinct from (factor-based) SEM.

The measurement model is generally written as

z=Cγ+ε, (2)

where **C** is a *J* by *P* matrix of loadings relating *P* components to *J* observed variables, and **ε** is a *J* by 1 vector of the residuals of all observed variables left unexplained by their components. The structural model is generally expressed as

γ=Bγ+ζ, (3)

where **B** is a *P* by *P* matrix of path coefficients relating *P* components among themselves, and **ζ** is a *P* by 1 vector of the residuals of all components left unexplained by their independent components. The combination of (1) and (2) can be seen as the constrained principal component analysis model [40] in that components of observed variables in (1) are obtained in such a way that they explain the maximum variances of the observed variables, signified by loadings in (2), as well as some elements of the **W** and **C** matrices are typically constrained to fixed values (e.g., zero) based on prior knowledge, as illustrated below.

**Fig 1:**
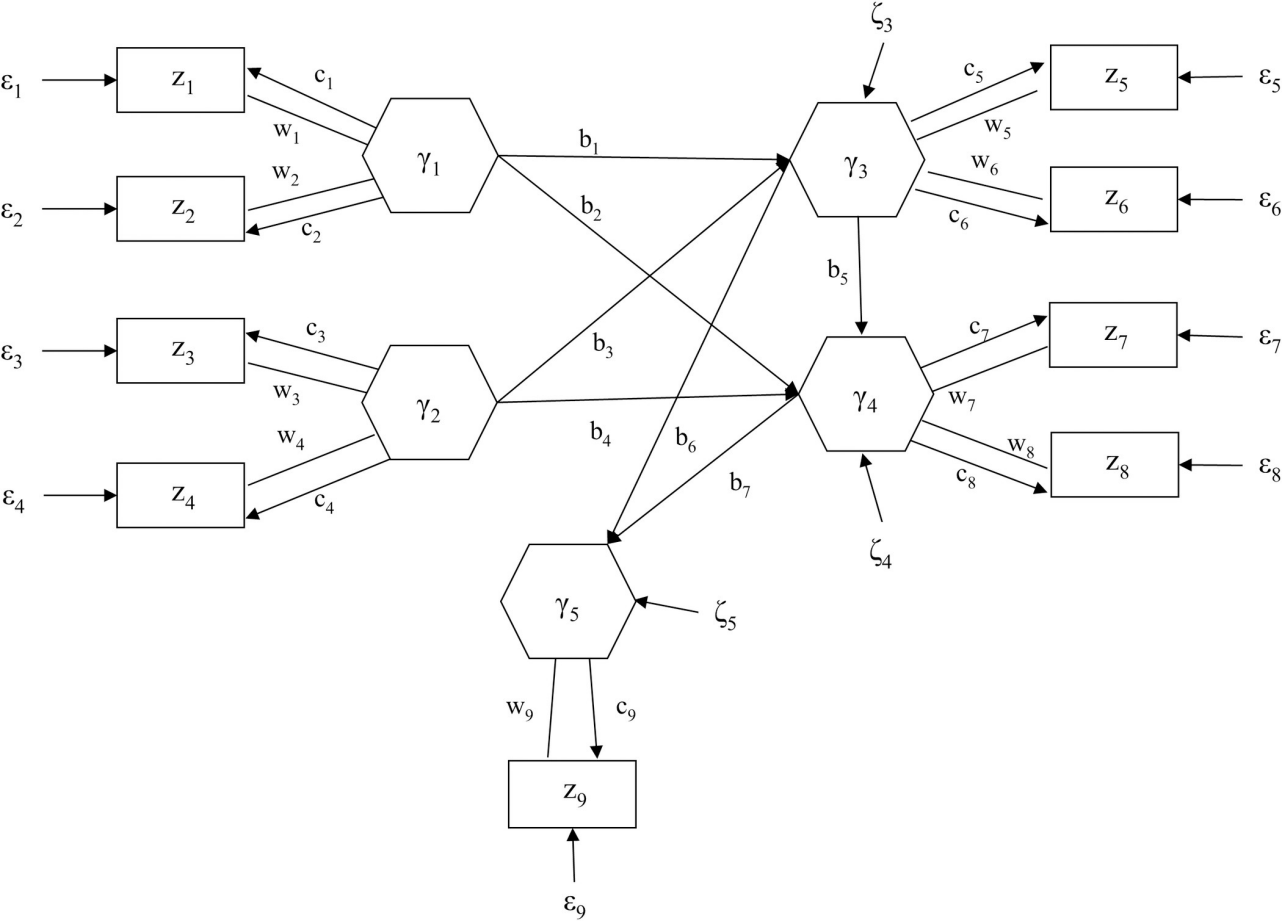
A path diagram of a prototype IG-GSCA model.

To exemplify these sub-models, we contemplate a prototype model depicted in Fig 1. In the figure, a box indicates an observed variable and a hexagon represents a component. An arrow signifies that the variable at the base of an arrow affects the variable at the head of the arrow, whereas a straight line indicates a weight assigned to each observed variable. This model contains two genes (γ_1_ and γ_2_) and two brain regions (γ_3_ and γ_4_), each of which is a weighed sum of two observed variables (SNPs or voxels). It includes one observed behavioral outcome. The model shows that the two genes affect the two brain regions, one brain region influences the other brain region, and both brain regions influence the behavioral outcome.

The weighted relation model for the prototype model can be expressed as

$$\left[\begin{array}{c} \gamma_{1}\\ \gamma_{2}\\ \gamma_{3}\\ \gamma_{4}\\ \gamma_{5} \end{array}\right]=\left[\begin{array}{ccccccccc} w_{1} & w_{2} & 0 & 0 & 0 & 0 & 0 & 0 & 0\\ 0 & 0 & w_{3} & w_{4} & 0 & 0 & 0 & 0 & 0\\ 0 & 0 & 0 & 0 & w_{5} & w_{6} & 0 & 0 & 0\\ 0 & 0 & 0 & 0 & 0 & 0 & w_{7} & w_{8} & 0\\ 0 & 0 & 0 & 0 & 0 & 0 & 0 & 0 & w_{9} \end{array}\right]\left[\begin{array}{c} z_{1}\\ z_{2}\\ z_{3}\\ z_{4}\\ z_{5}\\ z_{6}\\ z_{7}\\ z_{8}\\ z_{9} \end{array}\right]$$

γ=Wz. (4)

The measurement model for the prototype model can be written as follows

$$\left[\begin{array}{c} z_{1}\\ z_{2}\\ z_{3}\\ z_{4}\\ z_{5}\\ z_{6}\\ z_{7}\\ z_{8}\\ z_{9} \end{array}\right]=\left[\begin{array}{ccccc} c_{1} & 0 & 0 & 0 & 0\\ c_{2} & 0 & 0 & 0 & 0\\ 0 & c_{3} & 0 & 0 & 0\\ 0 & c_{4} & 0 & 0 & 0\\ 0 & 0 & c_{5} & 0 & 0\\ 0 & 0 & c_{6} & 0 & 0\\ 0 & 0 & 0 & c_{7} & 0\\ 0 & 0 & 0 & c_{8} & 0\\ 0 & 0 & 0 & 0 & c_{7} \end{array}\right]\left[\begin{array}{c} \gamma_{1}\\ \gamma_{2}\\ \gamma_{3}\\ \gamma_{4}\\ \gamma_{5} \end{array}\right]+\left[\begin{array}{c} \varepsilon_{1}\\ \varepsilon_{2}\\ \varepsilon_{3}\\ \varepsilon_{4}\\ \varepsilon_{5}\\ \varepsilon_{6}\\ \varepsilon_{7}\\ \varepsilon_{8}\\ \varepsilon_{9} \end{array}\right]$$

z=Cγ+ε. (5)

Finally, the structural model for the prototype can be expressed as

$$\left[\begin{array}{c} \gamma_{1}\\ \gamma_{2}\\ \gamma_{3}\\ \gamma_{4}\\ \gamma_{5} \end{array}\right]=\left[\begin{array}{ccccc} 0 & 0 & 0 & 0 & 0\\ 0 & 0 & 0 & 0 & 0\\ b_{1} & b_{2} & 0 & 0 & 0\\ b_{3} & b_{4} & b_{5} & 0 & 0\\ 0 & 0 & b_{6} & b_{7} & 0 \end{array}\right]\left[\begin{array}{c} \gamma_{1}\\ \gamma_{2}\\ \gamma_{3}\\ \gamma_{4}\\ \gamma_{5} \end{array}\right]+\left[\begin{array}{c} 0\\ 0\\ \zeta_{3}\\ \zeta_{4}\\ \zeta_{5} \end{array}\right]$$

γ=Bγ+ζ. (6)

This sub-model contains a series of regression models for all dependent components.

IG-GSCA combines the three sub-models into a single model, as follows.

$$\begin{array}{c} \left[\begin{array}{c} z\\ \gamma \end{array}\right]=\left[\begin{array}{c} C\\ B \end{array}\right]\gamma +\left[\begin{array}{c} \varepsilon \\ \zeta \end{array}\right]\\ \left[\begin{array}{c} z\\ Wz \end{array}\right]=\left[\begin{array}{c} C\\ B \end{array}\right]Wz+\left[\begin{array}{c} \varepsilon \\ \zeta \end{array}\right]\\ \left[\begin{array}{c} I\\ W \end{array}\right]z=\left[\begin{array}{c} C\\ B \end{array}\right]Wz+\left[\begin{array}{c} \varepsilon \\ \zeta \end{array}\right]\\ \mathrm{Vz}=\mathrm{AWz}+e, \end{array}$$ (7)

where **I** is an identity matrix, **V** = $\left[\begin{array}{c} I\\ W \end{array}\right]$, and **A** = $\left[\begin{array}{c} C\\ B \end{array}\right]$, and **e** = $\left[\begin{array}{c} \varepsilon \\ \xi \end{array}\right]$. We call (7) the IG-GSCA model, which enables to accommodate a variety of hypothesized G-B-B/C relationships.

In the prototype model, for simplicity, we consider only main effects of each component. However, we can also consider interaction effects of components, for example, gene-gene or gene-environment interactions. For example, let γ_12_ denote a gene-gene interaction term that is defined as the product of the two genes (i.e., γ_12_ = γ_1_γ_2_). Let γ* = [γ; γ_12_] denote a vector consisting of all components and the component interaction term. Then, the weighted relation model is given as

$$\gamma^{*}=\left[\begin{array}{cc} W & 0\\ 0 & 1 \end{array}\right]\left[\begin{array}{c} z\\ \gamma_{12} \end{array}\right]=W^{*}z^{*},$$ (8)

where W* = $\left[\begin{array}{cc} W & 0\\ 0 & 1 \end{array}\right]$, and z* = $\left[\begin{array}{c} z\\ \gamma_{12} \end{array}\right]$. The measurement model is generally given as

$$z=\left[C,\;0\right]\;\left[\begin{array}{c} \gamma \\ \gamma_{12} \end{array}\right]+\varepsilon =C^{*}\gamma^{*}+\varepsilon ,$$ (9)

where C* = [**C**, **0**]. The structural model is generally expressed as follows.

$$\gamma^{*}=B^{*}\gamma^{*}+\zeta^{*},$$ (10)

where B* consists of additional path coefficients relating γ_12_ to other variables. The model (7) can easily accommodate component interaction terms because the above sub-models are essentially of the same form as (1), (2), and (3).

### Parameter estimation

The unknown parameters of IG-GSCA include weights in **W**, loadings in **C**, and path coefficients in **B**. As illustrated in the previous section, the **W**, **C** and **B** matrices include fixed values (e.g., zeros) to express hypothesized relationships between variables, making it difficult to estimate the parameters in closed form. Instead, they are to be estimated iteratively. Moreover, components (e.g., genes and brain regions) and their interaction terms tend to be highly correlated to one another, leading to multicollinearity.

Let **z**_*i*_ denote a vector of indicators measured on a single observation of a sample of *N* observations (*i* = 1, …, *N*). To estimate the parameters, we aim to minimize the following penalized least squares criterion

$$\varphi =\frac{\tau }{2}\sum_{i=1}^{N}\left(Vz_{i}-\mathrm{AW}z_{i})'(Vz_{i}-\mathrm{AW}z_{i}\right)+\lambda 1'\left|B^{\tau }\right|1,$$ (11)

subject to ∑i=1Ndiag(γiγi')=NI, where **1** is a vector of ones of appropriate order, and λ is a non-negative tuning parameter for path coefficients. In (11), for any matrix **X**, | **X** | denotes the absolute value of **X**. When *τ* = 2, **1**'|**B**^*τ*^|**1** become the ridge or *L*_2_ penalty [41], whereas when *τ* = 1, it is equivalent to the lasso or *L*_1_ penalty [42]. Ridge or *L*_2_ regularization has been widely used to deal with multicollinearity, whereas lasso or *L*_1_ regularization is used for variable selection [43]. We are typically interested in dealing with multicollinearity, while keeping our model specification intact. It is known that within a certain range of the tuning parameter, the ridge estimator always exhibits a smaller mean square error than the ordinary least squares estimator [41]. This tendency becomes salient in the presence of multicollinearity [44]. Nonetheless, if variable section is of concern, lasso regularization can be adopted to select subsets of components, facilitating the parsimony and interpretability of the model.

We apply an alternating regularized least squares algorithm [24] to minimize this criterion. This algorithm will repeat three steps until convergence. In each step, one set of the parameters will be updated with the other sets fixed. If an interaction term of components is included, the algorithm estimates weights, considering that the component interaction term shares the same weights as those for its interacting components because it is the product of these components, each of which is a weighted sum of observed variables [23]. For instance, if γ_12_ is a gene-gene interaction between γ_1_ and γ_2_ in the prototype model, i.e., γ_12_ = γ_1_γ_2_ = (z_1_w_1_ + z_2_w_2_)(z_3_w_3_ + z_4_w_4_), then γ_12_ shares w_1_ and w_2_ with γ_1_, and w_3_ and w_4_ with γ_2_.

We employ *K*-fold cross validation [45] to determine the value of λ in an automatic manner. We use the bootstrap method [46] to estimate the standard errors or confidence intervals of the parameter estimates without resorting to a distributional assumption. The standard errors or confidence intervals can be used for testing the statistical significance of the parameter estimates. Upon convergence, IG-GSCA provides unique individual component scores as shown in (1).

## Example: Gene-brain-depression data

### Data overview

#### Participants

In a sample of 231 Korean participants, healthy volunteers were 137 (59.3%), who were recruited from community advertisements, whereas post-traumatic stress disorder (PTSD) patients were 94 (40.7%), who were recruited from notices on the bulletin board in a university hospital in a suburban area of Seoul, South Korea. The PTSD patients were diagnosed based on the Diagnostic and Statistical Manual of Mental Disorders, Fifth Edition (DSM-5) by a psychiatrist, and healthy participants were also evaluated using the DSM-5 by a psychiatrist. Participants were excluded if they were pregnant, intellectually disabled, drug-abusing, taking medications with potentially psychoactive effects, or at high risk for suicide. The sample consisted of 75 (32.5%) men and 156 (67.5%) women with a mean age of 46.10 years (SD = 13.49). All the participants signed a written form of informed consent, approved by the Institutional Review Board at Inje University Ilsan Paik Hospital prior to the start of the research (IRB no. 2015-07-025).

#### Measures

*Psychiatric and behavioral measures*. To measure the severity of depression as the outcome variable, the Korean translation of the Hospital Anxiety Depression Scale (HADS) [47] was administered. The HADS is a self-report rating scale and comprised of a set of seven questions for anxiety (HADS-A) and a set of seven questions for depression (HADS-D). The total sum score of the seven items in the HADS-D was used in the study.

To measure the exposure to traumatic events as an independent variable, the Korean validated version of Life Events Checklist (LEC) was used to assess the experience of potentially traumatic events (PTEs) [48]. The LEC comprised of 17 items of PTEs concerning experiencing, witnessing, and learning about PTEs. We used the items of PTE experience since other responses could be confusing to some respondents.

To control for the effect of alcohol-related problems as a covariate, the Alcohol Use Disorders Identification Test (AUDIT) was used to assess alcohol consumption, drinking behaviours, and alcohol-related problems. The AUDIT is a 10-item screening tool developed by the World Health Organization (WHO), and well-validated in Korea [49]. The AUDIT is assessed with a 5-point Likert scale ranging from 0 (“never”) to 4 (“4 or more times a week”). Table 1 provides a summary of demographic, psychological, and behavioral characteristics of the participants.

**Table 1:** A summary of participants’ demographic, psychological, and behavioral characteristics.

	Total participants	Healthy participants	PTSD participants
	(N = 231)	(N = 137)	(N = 94)
	Mean ± SD or N (%)		
Sex			
Male	75 (32.5)	40 (29.2)	35 (37.2)
Female	156 (67.5)	97 (70.8)	59 (62.8)
Age	46.10 ± 13.49	47.05 ± 13.58	44.72 ± 13.32
AUDIT	3.04 ± 3.61	2.80 ± 3.43	3.38 ± 3.86
PTE	3.72 ± 2.47	3.10 ± 2.35	4.63 ± 2.37
Depression	8.80 ± 4.59	6.80 ± 3.71	11.71 ± 418

PTSD—post-traumatic stress disorder; AUDIT—alcohol use disorders identification test; PTE—potentially traumatic events.

*DNA genotyping*. All participants had their blood sampled to extract DNA using NanoDrop® ND-1000 UV-Vis Spectrophotometer. Then, genomic DNA were diluted to 10 ng/㎕ concentration at 96 well PCR plates. TaqMan SNP Genotyping Assays were obtained from Applied Biosystems (Waltham, MA). The probes were labeled with FAM or VIC dye at the 5’ end and a minor-groove binder and non-fluorescent quencher at the 3’ end. 2 μL of DNA was added to each 5 μL PCR reaction at 384 well reaction plates. SNP genotyping reactions were performed on ABI PRISM 7900HT Real-time PCR system. After the PCR amplification, allelic discrimination is performed at the same machines (ABI 7900HT). The allelic discrimination is an end point plate read. The SDS v2.4 software calculates the fluorescence measurements made during the plate read and plots Rn values based on the signals from each well. A total of 18 SNPs from 9 different DNAs were obtained for the study. For all SNPs, the wild, hetero, and mutant genotypes were coded as 1, 2, and 3, respectively. Nine genes selected based on their relations with depression include: SLC6A4 [50], FKBP5 [51], ADCYAP1R1 [52], BDNF [53], COMT [54], HTR3A [55], DRD2 [56], NR3C1 [57], and OXTR [58]. Table 2 exhibits the names and frequencies of all the genes considered in the study.

**Table 2:** List of genes and SNPs included in the gene-brain-depression data.

Gene	SNP	wild [N(%)]			hetero [N(%)]			mutant [N(%)]	
SLC6A4	rs25531	AA	174 (75.3%)	AG		57 (24.7)	GG		0 (0%)
FKBP5	rs9296158	GG	115 (49.8%)	AG		98 (42.4%)	AA		18(7.8%)
	rs3800373	AA	148 (64.1%)	AC		71 (30.7%)	CC		12 (5.2%)
	rs1360780	CC	144 (62.3%)	CT		74 (32.0%)	TT		13 (5.6%)
	rs9470080	CC	111 (48.1%)	CT		100 (43.2%)	TT		20 (8.7%)
	rs4713916	GG	146 (63.2%)	AG		74 (32.0%)	AA		11 (4.8%)
	rs4713919	GG	130 (56.3%)	AG		82 (35.5%)	AA		19 (8.2%)
	rs6902321	TT	118 (51.1%)	CT		97 (42.0%)	CC		16 (6.9%)
	rs56311918	TT	168 (72.7%)	CT		59 (25.5%)	CC		4 (1.7%)
	rs3798345	CC	157 (68.0%)	CT		66 (28.5%)	TT		8 (3.5%)
ADCYAP1R1	rs2267735	CC	59 (25.5%)	CG		119 (51.5%)	GG		53 (22.9%)
BDNF	rs6265	CC	75 (32.5%)	CT		108 (46.8%)	TT		48 (20.8%)
COMT	rs4680	GG	116 (50.2%)	AG		102 (44.2%)	AA		13 (5.6%)
	rs4633	CC	119 (51.5%)	CT		100 (43.3%)	TT		12 (5.2%)
HTR3A	rs1062613	CC	194 (84.0%)	CT		34 (14.7%)	TT		3 (1.3%)
DRD2	rs2075652	GG	86 (37.2%)	GA		99 (42.9%)	AA		46 (19.9%)
NR3C1	rs258747	AA	125 (54.1%)	AG		89 (38.5%)	GG		17 (7.4%)
OXTR	rs53576	AA	92 (39.8%)	AG		99 (42.9%)	GG		40 (17.3%)

*MRI acquisition and processing and voxel-based morphometry*. MRI was performed using a 1.5 T scanner (Magneton Avanto, Siemens, Erlangen, Germany). Head motion was minimized with restraining foam pads provided by the manufacturer. High-resolution T1-weighted MRI images were acquired with the acquisition parameters of a 227 × 384 acquisition matrix, a 210 × 250 field-of-view, 0.9 × 0.7 × 1.2 voxel size, a total of 87,168 voxels, a TE of 3.42 ms, a TR of 1,900 ms, 1.2 mm slice thickness, and a flip angle of 15°.

All images were inspected visually for motion or other artifacts before and after preprocessing. The voxel-based volumetry (VBM) was conducted using CAT12 (http://dbm.neuro.uni-jena.de/cat/) implemented in SPM12 (Wellcome Department of Cognitive Neurology, London, UK). SPM12 tissue probability maps were used for the initial spatial registration. The structural T1 images were regularized with an ICBM East Asian template and normalized using the DARTEL algorithm [59]. The images were then segmented into gray matter, white matter, and cerebrospinal fluid [60]. Jacobian-transformed tissue probability maps were used to modulate images. The projection-based thickness method was applied to the SPM analysis to estimate the cortical thickness for the left and right hemispheres [61]. The cortical thickness was extracted using the Destrieux atlas, which is the default FreeSurfer atlas. The Destrieux atlas consists of 74 cortical areas in the left and right hemispheres, including both gyri and sulci. Segmentation was automatically conducted using probabilistic methods [62]. A total of 53 regions of interest (ROIs), which mostly represent the thickness of cortical gyri and limbic sulci, were selected from the atlas for the study. Table 3 shows the name, mean, and standard deviation of each ROI.

**Table 3:** List of regions of interest (ROIs) included in the gene-brain-depression data.

ROI	Hemisphere	Mean ± SD	ROI	Hemisphere	Mean ± SD
precentral gyrus	Left-	2.75 ± 0.19	Inferior temporal gyrus (T3)	Left-	3.13 ± 0.16
	Right-	2.73 ± 0.22		Right-	3.15 ± 0.17
subcentral gyrus	Left-	2.75 ± 0.17	lateral occipito-temporal gyrus (fusiform gyrus, O4-T4)	Left-	2.88 ± 0.18
	Right-	2.75 ± 0.18		Right-	2.88 ± 0.17
inferior frontal gyrus (or F3)	Left-	2.39 ± 0.27	lingual gyrus (O5)	Left-	1.95 ± 0.13
	Right-	2.44 ± 0.25		Right-	2.06 ± 0.14
triangular part of the inferior frontal gyrus	Left-	2.71 ± 0.21	parahippocampal gyrus (or T5)	Left-	3.13 ± 0.24
	Right-	2.73 ± 0.19		Right-	3.23 ± 0.25
opercular part of the inferior frontal gyrus	Left-	2.80 ± 0.17	cuneus (O6)	Left-	1.77 ± 0.13
	Right-	2.79 ± 0.16		Right-	1.83 ± 0.13
orbital part of the inferior frontal gyrus	Left-	2.82 ± 0.22	occipital pole	Left-	1.94 ± 0.15
	Right-	2.88 ± 0.25		Right-	1.95 ± 0.13
middle frontal gyrus (or F2)	Left-	2.71 ± 0.17	temporal pole	Left-	3.44 ± 0.19
	Right-	2.70 ± 0.16		Right-	3.45 ± 0.24
superior frontal gyrus	Left-	3.04 ± 0.16	postcentral gyrus	Left-	2.12 ± 0.16
	Right-	3.01 ± 0.18		Right-	2.10 ± 0.17
gyrus rectus	Left-	2.78 ± 0.20	supramarginal gyrus	Left-	2.76 ± 0.16
	Right-	2.73 ± 0.19		Right-	2.77 ± 0.15
transverse frontopolar gyrus or gyri	Left-	2.73 ± 0.27	angular gyrus	Left-	2.68 ± 0.16
	Right-	2.72 ± 0.25		Right-	2.66 ± 0.15
medial orbital sulcus	Left-	2.42 ± 0.19	superior parietal lobule (or P1)	Left-	2.37 ± 0.16
	Right-	2.40 ± 0.18		Right-	2.36 ± 0.16
4 orbital gyri	Left-	2.86 ± 0.15	precuneus	Left-	2.52 ± 0.16
	Right-	2.91 ± 0.16		Right-	2.52 ± 0.16
superior circular sulcus of the insula	Left-	2.67 ± 0.12	paracentral lobule and sulcus	Left-	2.29 ± 0.22
	Right-	2.67 ± 0.14		Right-	2.33 ± 0.21
anterior circular sulcus of the insula	Left-	2.84 ±0.20	subcentral gyrus and sulci	Left-	2.75 ± 0.17
	Right-	2.87 ± 0.22		Right-	2.75 ± 0.18
inferior circular sulcus of the insula	Left-	2.71 ± 0.21	Marginal branch (or part) of the cingulate sulcus	Left-	2.24 ± 0.16
	Right-	2.61 ± 0.22		Right-	2.25 ± 0.16
vertical ramus of anterior segment of lateral sulcus	Left-	2.44 ± 0.23	subparietal sulcus	Left-	2.40 ± 0.18
	Right-	2.53 ± 0.27		Right-	2.46 ± 0.20
horizontal ramus of anterior segment of lateral sulcus	Left-	2.33 ± 0.30	calcarine sulcus	Left-	1.72 ± 0.12
	Right-	2.35 ± 0.23		Right-	1.81 ± 0.14
posterior segment of the lateral sulcus	Left-	2.43 ± 0.17	medial occipitotemporal sulcus (or collateral sulcus)	Left-	2.35 ± 0.17
	Right-	2.46 ± 0.17		Right-	2.37 ± 0.20
the short insular gyri	Left-	3.54 ± 0.20	lateral occipito-temporal (or fusiform) sulcus	Left-	2.55 ± 0.18
	Right-	3.42 ± 0.22		Right-	2.57 ± 0.16
long insular gyrus	Left-	3.27 ± 0.24	subcallosal area or gyrus	Left-	2.74 ± 0.25
	Right-	3.32 ± 0.30		Right-	2.74 ± 0.35
transverse temporal gyrus (or Heschl’s gyrus)	Left-	2.39 ± 0.24	pericallosal sulcus or sulcus of the corpus callosum	Left-	2.26 ± 0.31
	Right-	2.42 ± 0.24		Right-	2.24 ± 0.35
Planum temporale or temporal plane of the superior temporal gyrus	Left-	2.53 ± 0.22	Anterior part of the cingulate gyrus and sulcus (ACC)	Left-	2.88 ± 0.17
	Right-	2.56 ± 0.21		Right-	2.85 ± 0.20
Planum polare of the superior temporal gyrus	Left-	3.38 ± 0.26	Middle-anterior part of the cingulate gyrus and sulcus (aMCC)	Left-	2.78 ± 0.23
	Right-	3.27 ± 0.24		Right-	2.85 ± 0.20
lateral aspect of the superior temporal gyrus	Left-	3.11 ± 0.19	Middle-posterior part of the cingulate gyrus and sulcus (pMCC)	Left-	2.62 ± 0.16
	Right-	3.12 ± 0.20		Right-	2.66 ± 0.16
middle temporal gyrus	Left-	3.07 ± 0.17	Posterior-dorsal part of the cingulate gyrus (dPCC)	Left-	2.91 ± 0.17
	Right-	3.07 ± 0.15		Right-	2.91 ± 0.17
superior occipital gyrus (O1)	Left-	2.17 ± 0.20	Posterior-ventral part of the cingulate gyrus (vPCC or isthmus)	Left-	2.64 ± 0.25
	Right-	2.21 ± 0.16		Right-	2.67 ± 0.25
middle occipital gyrus (O2, lateral occipital gyrus)	Left-	2.57 ± 0.14			
	Right-	2.63 ± 0.13			

### Model specification

**Fig 2:**
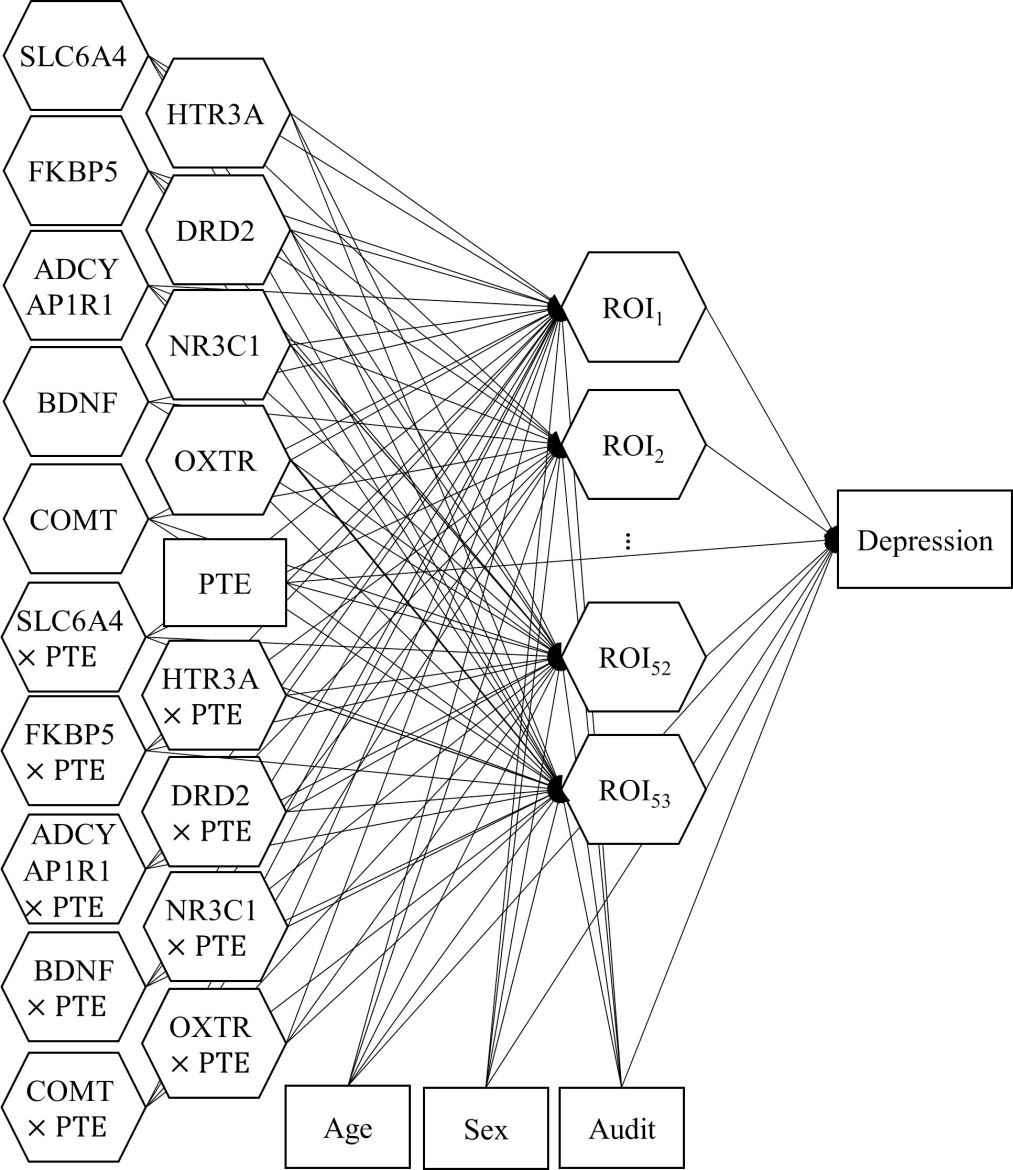
The structural model specified for the gene-brain-depression data. All weights and residual terms are omitted to make the figure concise.

Depression symptoms are known to be linked to altered brain structures [63], which may be influenced by the number of exposure to stressful or traumatic life events [64, 65], genetic polymorphism [66], and the interaction of both—gene-environment interactions [67–69]. Also, these relations may be affected by covariates such as age [70, 71], sex [72, 73], and alcohol-related problems [74, 75]. Accordingly, we hypothesized that the PTE, genetic polymorphism, and their interactions directly influenced the cortical thickness of the ROIs, which in turn had direct effects on depression severity, while controlling for the effects of age, sex, and AUDIT on both cortical thickness and depression severity. We also assumed that the PTE influenced depression severity directly. Fig 2 displays the hypothesized structural model. As shown in the figure, the model consisted of nine genetic components (i.e., genes) and 53 imaging components (i.e., ROIs). Each gene was associated with its observed variables (i.e., SNPs). The number of SNPs per gene ranged from one to nine. Each ROI was associated with two observed variables that denoted its left and right sides of the brain. Nine gene-environment interactions between the genes and PTE were considered that also influenced the ROIs.

### Results

We applied IG-GSCA to fit the specified model to the data. We chose λ = 136 based on five-fold cross validation. We used 4000 bootstrap samples to estimate the standard errors and 95% confidence intervals of the parameter estimates. As shown in Table 4, all weight estimates were statistically significant, suggesting that all observed variables contributed to forming their corresponding components. In addition, all the loading estimates were statistically significant and large in magnitude (> .75). This indicates that all components were obtained to explain the variances of their observed variables well.

**Table 4:** The estimates of weights and loadings and their standard errors and 95% confidence intervals.

Name		Weights				Loadings			
Component	Observed variable	Estimate	SE	95% CI		Estimate	SE	95% CI	
*SLC6A4*	rs25531	1.00	0.00	1.00	1.00	1.00	0.00	1.00	1.00
*FKBP5*	rs9296158	0.15	0.05	0.05	0.27	0.89	0.02	0.85	0.92
	rs3800373	0.10	0.04	0.01	0.18	0.88	0.03	0.82	0.93
	rs1360780	0.13	0.05	0.04	0.25	0.92	0.01	0.89	0.95
	rs9470080	0.14	0.07	0.00	0.27	0.91	0.01	0.88	0.93
	rs4713916	0.14	0.06	0.02	0.26	0.93	0.01	0.90	0.95
	rs4713919	0.10	0.03	0.04	0.17	0.85	0.03	0.80	0.90
	rs6902321	0.11	0.05	0.01	0.21	0.89	0.02	0.85	0.92
	rs56311918	0.15	0.03	0.10	0.21	0.84	0.03	0.78	0.88
	rs3798345	0.11	0.05	0.01	0.20	0.89	0.02	0.84	0.93
*ADCYAP1R1*	rs2267735	1.00	0.00	1.00	1.00	1.00	0.00	1.00	1.00
*BDNF*	rs6265	1.00	0.00	1.00	1.00	1.00	0.00	1.00	1.00
*COMT*	rs4680	0.47	0.13	0.22	0.73	0.98	0.01	0.95	1.00
	rs4633	0.54	0.13	0.29	0.79	0.98	0.01	0.96	1.00
*HTR3A*	rs1062613	1.00	0.00	1.00	1.00	1.00	0.00	1.00	1.00
*DRD2*	rs2075652	1.00	0.00	1.00	1.00	1.00	0.00	1.00	1.00
*NR3C1*	rs258747	1.00	0.00	1.00	1.00	1.00	0.00	1.00	1.00
*OXTR*	rs53576	1.00	0.00	1.00	1.00	1.00	0.00	1.00	1.00
PTE	post traumatic events	1.00	0.00	1.00	1.00	1.00	0.00	1.00	1.00
precentral gyrus	Left precentral gyrus	0.53	0.02	0.50	0.57	0.91	0.02	0.86	0.94
	Right precentral gyrus	0.56	0.02	0.53	0.61	0.92	0.02	0.89	0.94
subcentral gyrus	Left subcentral gyrus	0.55	0.02	0.51	0.59	0.90	0.01	0.87	0.92
	Right subcentral gyrus	0.56	0.02	0.52	0.60	0.90	0.01	0.87	0.93
inferior frontal gyrus (or F3)	Left inferior frontal gyrus (vertical)	0.61	0.03	0.55	0.66	0.81	0.03	0.74	0.85
	Right inferior frontal gyrus (vertical)	0.62	0.03	0.57	0.70	0.82	0.02	0.77	0.86
triangular part of the inferior frontal gyrus	Left triangular part of the inferior frontal gyrus	0.57	0.02	0.53	0.62	0.91	0.01	0.88	0.93
	Right triangular part of the inferior frontal gyrus	0.53	0.02	0.49	0.58	0.89	0.01	0.86	0.92
opercular part of the inferior frontal gyrus	Left opercular part of the inferior frontal gyrus	0.56	0.02	0.52	0.60	0.88	0.02	0.83	0.91
	Right opercular part of the inferior frontal gyrus	0.58	0.02	0.53	0.62	0.88	0.02	0.85	0.91
orbital part of the inferior frontal gyrus	Left orbital part of the inferior frontal gyrus	0.62	0.02	0.57	0.66	0.87	0.02	0.83	0.89
	Right orbital part of the inferior frontal gyrus	0.56	0.02	0.52	0.61	0.83	0.02	0.79	0.87
middle frontal gyrus (or F2)	Left middle frontal gyrus (or F2)	0.52	0.02	0.48	0.57	0.95	0.01	0.93	0.97
	Right middle frontal gyrus (or F2)	0.53	0.02	0.49	0.57	0.95	0.01	0.94	0.97
superior frontal gyrus	Left superior frontal gyrus	0.53	0.03	0.48	0.58	0.97	0.00	0.96	0.98
	Right superior frontal gyrus	0.50	0.03	0.45	0.55	0.97	0.00	0.96	0.98
gyrus rectus	Left gyrus rectus	0.57	0.02	0.53	0.61	0.90	0.02	0.86	0.93
	Right gyrus rectus	0.55	0.02	0.51	0.60	0.89	0.02	0.86	0.92
transverse frontopolar gyrus or gyri	Left transverse frontopolar gyrus or gyri	0.55	0.02	0.52	0.59	0.92	0.01	0.90	0.94
	Right transverse frontopolar gyrus or gyri	0.53	0.02	0.49	0.57	0.92	0.01	0.89	0.94
medial orbital sulcus	Left medial orbital sulcus	0.55	0.02	0.51	0.59	0.88	0.02	0.84	0.91
	Right medial orbital sulcus	0.58	0.02	0.54	0.62	0.89	0.02	0.86	0.92
4 orbital gyri	Left 4 orbital gyri	0.54	0.02	0.50	0.57	0.91	0.01	0.88	0.93
	Right 4 orbital gyri	0.56	0.02	0.52	0.60	0.92	0.01	0.89	0.94
superior circular sulcus of the insula	Left superior(circular sulcus of the insula)	0.54	0.02	0.51	0.58	0.90	0.01	0.87	0.92
	Right superior(circular sulcus of the insula)	0.56	0.02	0.53	0.60	0.91	0.01	0.88	0.93
anterior circular sulcus of the insula	Left anterior(circular sulcus of the insula)	0.60	0.03	0.54	0.67	0.82	0.03	0.75	0.86
	Right anterior(circular sulcus of the insula)	0.61	0.03	0.55	0.68	0.83	0.03	0.76	0.87
inferior circular sulcus of the insula	Left inferior(circular sulcus of the insula)	0.57	0.02	0.52	0.62	0.91	0.01	0.88	0.93
	Right inferior(circular sulcus of the insula)	0.54	0.02	0.50	0.58	0.90	0.02	0.85	0.93
vertical ramus of anterior segment of lateral sulcus	Left vertical ramus of anterior segment of lateral sulcus	0.61	0.03	0.55	0.66	0.81	0.03	0.74	0.85
	Right vertical ramus of anterior segment of lateral sulcus	0.62	0.03	0.57	0.70	0.82	0.02	0.77	0.86
horizontal ramus of anterior segment of lateral sulcus	Left horizontal ramus of anterior segment of lateral sulcus	0.66	0.05	0.56	0.74	0.78	0.05	0.66	0.84
	Right horizontal ramus of anterior segment of lateral sulcus	0.64	0.06	0.56	0.75	0.76	0.05	0.69	0.84
posterior segment of the lateral sulcus	Left posterior segment of the lateral sulcus	0.55	0.02	0.51	0.60	0.87	0.02	0.83	0.90
	Right posterior segment of the lateral sulcus	0.59	0.02	0.55	0.63	0.89	0.02	0.85	0.91
the short insular gyri	Left short insular gyri	0.62	0.03	0.57	0.68	0.85	0.02	0.81	0.88
	Right short insular gyri	0.57	0.02	0.53	0.63	0.82	0.03	0.76	0.86
long insular gyrus	Left long insular gyrus	0.61	0.02	0.56	0.66	0.87	0.02	0.83	0.90
	Right long insular gyrus	0.56	0.02	0.52	0.61	0.84	0.02	0.79	0.88
transverse temporal gyrus (or Heschl’s gyrus)	Left transverse temporal gyrus (or Heschl’s gyrus)	0.57	0.02	0.53	0.62	0.86	0.02	0.83	0.89
	Right transverse temporal gyrus (or Heschl’s gyrus)	0.58	0.02	0.54	0.63	0.87	0.02	0.83	0.90
Planum temporale or temporal plane of the superior temporal gyrus	Left Planum temporale or temporal plane of the superior temporal gyrus	0.57	0.02	0.53	0.62	0.85	0.02	0.81	0.89
	Right Planum temporale or temporal plane of the superior temporal gyrus	0.60	0.02	0.55	0.64	0.87	0.02	0.82	0.90
Planum polare of the superior temporal gyrus	Left Planum polare of the superior temporal gyrus	0.60	0.03	0.54	0.66	0.83	0.03	0.77	0.87
	Right Planum polare of the superior temporal gyrus	0.61	0.03	0.55	0.67	0.83	0.02	0.78	0.87
lateral aspect of the superior temporal gyrus	Left lateral aspect of the superior temporal gyrus	0.55	0.02	0.51	0.59	0.89	0.02	0.86	0.92
	Right lateral aspect of the superior temporal gyrus	0.57	0.02	0.53	0.60	0.90	0.02	0.86	0.93
middle temporal gyrus	Left middle temporal gyrus	0.56	0.02	0.52	0.60	0.91	0.01	0.89	0.93
	Right middle temporal gyrus	0.54	0.02	0.50	0.58	0.90	0.01	0.88	0.93
superior occipital gyrus (O1)	Left superior occipital gyrus (O1)	0.58	0.02	0.54	0.63	0.87	0.02	0.83	0.90
	Right superior occipital gyrus (O1)	0.57	0.02	0.53	0.62	0.86	0.02	0.83	0.89
middle occipital gyrus (O2, lateral occipital gyrus)	Left middle occipital gyrus (O2, lateral occipital gyrus)	0.57	0.02	0.53	0.61	0.88	0.02	0.84	0.91
	Right middle occipital gyrus (O2, lateral occipital gyrus)	0.57	0.02	0.53	0.61	0.88	0.02	0.84	0.91
Inferior temporal gyrus (T3)	Left Inferior temporal gyrus (T3)	0.58	0.02	0.54	0.63	0.87	0.01	0.84	0.90
	Right Inferior temporal gyrus (T3)	0.57	0.02	0.53	0.60	0.87	0.02	0.83	0.90
lateral occipito-temporal gyrus (fusiform gyrus, O4-T4)	Left lateral occipito-temporal gyrus (fusiform gyrus, O4-T4)	0.56	0.02	0.52	0.60	0.88	0.02	0.84	0.91
	Right lateral occipito-temporal gyrus (fusiform gyrus, O4-T4)	0.58	0.02	0.54	0.62	0.88	0.02	0.85	0.91
lingual gyrus (O5)	Left lingual gyrus (O5)	0.58	0.02	0.54	0.61	0.90	0.01	0.87	0.93
	Right lingual gyrus (O5)	0.54	0.02	0.50	0.58	0.89	0.02	0.86	0.92
parahippocampal gyrus (or T5)	Left parahippocampal gyrus (or T5)	0.56	0.02	0.51	0.60	0.88	0.02	0.84	0.92
	Right parahippocampal gyrus (or T5)	0.57	0.02	0.53	0.61	0.89	0.02	0.85	0.93
cuneus (O6)	Left cuneus (O6)	0.53	0.02	0.49	0.58	0.89	0.02	0.86	0.92
	Right cuneus (O6)	0.58	0.02	0.54	0.61	0.91	0.02	0.87	0.94
occipital pole	Left occipital pole	0.59	0.02	0.55	0.63	0.87	0.02	0.82	0.90
	Right occipital pole	0.57	0.02	0.53	0.62	0.86	0.02	0.81	0.90
temporal pole	Left temporal pole	0.59	0.02	0.54	0.63	0.87	0.02	0.83	0.91
	Right temporal pole	0.56	0.03	0.52	0.62	0.86	0.02	0.82	0.90
postcentral gyrus	Left postcentral gyrus	0.55	0.02	0.51	0.59	0.90	0.01	0.87	0.92
	Right postcentral gyrus	0.56	0.02	0.52	0.60	0.90	0.01	0.88	0.93
supramarginal gyrus	Left supramarginal gyrus	0.54	0.02	0.49	0.58	0.91	0.01	0.88	0.93
	Right supramarginal gyrus	0.56	0.02	0.52	0.60	0.92	0.01	0.89	0.94
angular gyrus	Left angular gyrus	0.55	0.02	0.51	0.59	0.89	0.02	0.86	0.92
	Right angular gyrus	0.57	0.02	0.53	0.61	0.90	0.01	0.87	0.92
superior parietal lobule (or P1)	Left superior parietal lobule (or P1)	0.51	0.02	0.47	0.55	0.93	0.01	0.91	0.95
	Right superior parietal lobule (or P1)	0.56	0.02	0.52	0.60	0.94	0.01	0.92	0.96
precuneus	Left precuneus	0.55	0.02	0.51	0.59	0.94	0.01	0.93	0.96
	Right precuneus	0.52	0.02	0.48	0.55	0.93	0.01	0.91	0.95
paracentral lobule and sulcus	Left paracentral lobule and sulcus	0.55	0.02	0.51	0.59	0.93	0.01	0.91	0.95
	Right paracentral lobule and sulcus	0.53	0.02	0.49	0.57	0.92	0.01	0.90	0.94
subcentral gyrus and sulci	Left subcentral gyrus and sulci	0.55	0.02	0.51	0.59	0.90	0.01	0.87	0.92
	Right subcentral gyrus and sulci	0.56	0.02	0.52	0.60	0.90	0.01	0.87	0.93
Marginal branch (or part) of the cingulate sulcus	Left marginal branch (or part) of the cingulate sulcus	0.55	0.02	0.51	0.59	0.90	0.01	0.87	0.93
	Right marginal branch (or part) of the cingulate sulcus	0.56	0.02	0.52	0.59	0.91	0.01	0.88	0.93
subparietal sulcus	Left subparietal sulcus	0.60	0.02	0.56	0.64	0.88	0.01	0.84	0.90
	Right subparietal sulcus	0.55	0.02	0.52	0.60	0.85	0.02	0.81	0.89
calcarine sulcus	Left calcarine sulcus	0.55	0.02	0.51	0.58	0.91	0.02	0.88	0.94
	Right calcarine sulcus	0.55	0.02	0.51	0.59	0.91	0.01	0.88	0.94
medial occipitotemporal sulcus (or collateral sulcus)	Left medial occipitotemporal sulcus (or collateral sulcus)	0.58	0.04	0.50	0.66	0.87	0.03	0.81	0.93
	Right medial occipitotemporal sulcus (or collateral sulcus)	0.57	0.02	0.52	0.62	0.86	0.04	0.79	0.93
lateral occipito-temporal (or fusiform) sulcus	Left lateral occipito-temporal (or fusiform) sulcus	0.63	0.04	0.56	0.70	0.79	0.04	0.68	0.84
	Right lateral occipito-temporal (or fusiform) sulcus	0.64	0.05	0.58	0.74	0.79	0.05	0.72	0.84
subcallosal area or gyrus	Left subcallosal area or gyrus	0.58	0.04	0.51	0.65	0.75	0.05	0.64	0.82
	Right subcallosal area or gyrus	0.68	0.04	0.61	0.78	0.83	0.03	0.77	0.87
pericallosal sulcus or sulcus of the corpus callosum	Left pericallosal sulcus or sulcus of the corpus callosum	0.59	0.02	0.55	0.64	0.87	0.02	0.83	0.91
	Right pericallosal sulcus or sulcus of the corpus callosum	0.57	0.02	0.52	0.61	0.86	0.02	0.81	0.89
anterior part of the cingulate gyrus and sulcus (ACC)	Left anterior part of the cingulate gyrus and sulcus (ACC)	0.53	0.02	0.50	0.57	0.93	0.01	0.90	0.95
	Right anterior part of the cingulate gyrus and sulcus (ACC)	0.54	0.02	0.51	0.58	0.93	0.01	0.91	0.95
middle-anterior part of the cingulate gyrus and sulcus (aMCC)	Left middle-anterior part of the cingulate gyrus and sulcus (aMCC)	0.52	0.02	0.49	0.57	0.90	0.02	0.85	0.94
	Right middle-anterior part of the cingulate gyrus and sulcus (aMCC)	0.58	0.02	0.54	0.62	0.92	0.02	0.87	0.95
middle-posterior part of the cingulate gyrus and sulcus (pMCC)	Left middle-posterior part of the cingulate gyrus and sulcus (pMCC)	0.58	0.02	0.54	0.62	0.91	0.01	0.89	0.93
	Right middle-posterior part of the cingulate gyrus and sulcus (pMCC)	0.53	0.02	0.49	0.57	0.89	0.01	0.86	0.92
posterior-dorsal part of the cingulate gyrus and sulcus (dPCC)	Left posterior-dorsal part of the cingulate gyrus and sulcus (dPCC)	0.59	0.02	0.54	0.63	0.90	0.01	0.87	0.92
	Right posterior-dorsal part of the cingulate gyrus and sulcus (dPCC)	0.54	0.02	0.50	0.58	0.88	0.02	0.84	0.91
posterior-ventral part of the cingulate gyrus and sulcus (vPCC or isthmus)	Left posterior-ventral part of the cingulate gyrus and sulcus (vPCC or isthmus)	0.56	0.02	0.51	0.61	0.86	0.02	0.81	0.89
	Right posterior-ventral part of the cingulate gyrus and sulcus (vPCC or isthmus)	0.59	0.02	0.55	0.64	0.87	0.02	0.83	0.90
Depression	HAD-Depression	1.00	0.00	1.00	1.00	1.00	0.00	1.00	1.00
gender	gender	1.00	0.00	1.00	1.00	1.00	0.00	1.00	1.00
age	age	1.00	0.00	1.00	1.00	1.00	0.00	1.00	1.00
AUDIT	AUDITpre	1.00	0.00	1.00	1.00	1.00	0.00	1.00	1.00

Genes and ROIs are components, and SNPs and brain hemispheres are observed variable.

The specified model included a total of 1,246 path coefficients. One hundred eighty-four of their estimates turned out to be statistically significant. To conserve space, we focus here on reporting and interpreting statistically significant path coefficient estimates that constituted the hypothesized G-B-B/C pathways linking the genes, ROIs, and depression severity, presented in Table 5. The full results of all the path coefficient estimates can be found in S1 Table.

**Table 5:** Statistically significant estimates of the path coefficients that constitute the linkages from genes to ROIs to depression severity, and their standard errors and 95% confidence intervals.

Path coefficients		Estimate	SE	95% CI	
DRD2 (gene)	Middle-posterior part (pMCC)	0.07	0.03	0.01	0.14
DRD2 (gene)	Triangular part of the inferior frontal gyrus	0.11	0.03	0.04	0.17
PTE	Triangular part of the inferior frontal gyrus	-0.08	0.03	-0.14	-0.01
PTE x SLC6A4 (gene)	Triangular part of the inferior frontal gyrus	0.06	0.03	0.00	0.02
Triangular part of the inferior frontal gyrus	Depression	-0.07	0.03	-0.12	0.00
Anterior (circular sulcus of the insula)	Depression	-0.11	0.03	-0.17	-0.05
Middle-posterior part (pMCC)	Depression	-0.08	0.03	-0.14	-0.01
PTE	Depression	0.14	0.03	0.07	0.20

In the specified gene and brain function relationships, the dopamine receptor D2 (DRD2) gene had positive influences on the middle-posterior part of the cingulate gyrus and sulcus (pMCC) (*b* = .07, SE = .03, 95% CI = [.01, .14]) and the triangular part of the inferior frontal gyrus (*b* = .11, SE = .03, 95% CI = [.04, .17]), suggesting that people with the mutant allele in DRD2 are likely to have a thicker triangular part of the inferior frontal gyrus and pMCC. This finding is consistent with previous research that people with the wild genotype of the DRD2 gene (GG) had reduced activity in the inferior frontal gyrus [76] and reduced connectivity in pMCC [77] relative to people with the hetero or mutant genotype. In the specified brain function and depression relationships, three ROIs, such as the triangular part of the inferior frontal gyrus (*b* = -.07, SE = .03, 95% CI = [-.12, -.00]), the anterior circular sulcus of the insula (*b* = -.11, SE = .03, 95% CI = [-.17, -.05]), and pMCC (*b* = -.08, SE = .03, 95% CI = [-.14, -.01]), turned out to be negatively associated with depression severity, indicating that the thinner these ROIs are, the higher level of depression on average. This is consistent with previous findings that revealed a significantly thinner or smaller triangular part of the inferior frontal gyrus [78], anterior insula [79], and pMCC [80] in depressive people than those in healthy people. Moreover, PTE had a negative effect on the triangular part of the inferior frontal gyrus (*b* = -.08, SE = .03, 95% CI = [-.14, -.01]), suggesting that traumatic stressful events may diminish the thickness of this part. This finding is supported by previous research that traumatic experiences were associated with a thinner or smaller triangular part of the inferior frontal gyrus [81, 82]. Lastly, PTE had a positive impact on depression severity (*b* = .14, SE = .03, 95% CI = [.07, .20]). This also indicates that the effect of PTE on depression severity was not fully mediated by the ROIs. The association between stressful or traumatic experiences and depression has been found in numerous studies [83–86].

The gene-environment interaction between PTE and the serotonin transporter gene (SLC6A4) had a significant effect on the triangular part of the inferior frontal gyrus (*b* = .06, SE = .03, 95% CI = [.00, .02]). We additionally investigated the conditional effects of PTE on the triangular part of the inferior frontal gyrus at different levels of the serotonin transporter gene. Specifically, the conditional effects were tested when rs25531 was AA (the wild genotype) and AG (the hetero genotype), since SLC6A4 had only one observed variable rs25531, whose values were AA and AG in the data. It turned out that PTE had a negative effect on the triangular part of the inferior frontal gyrus only when rs25531 was AA (*b* = -.11, SE = .04, 95% CI = [-.18, -.03]). Although there are few studies regarding the interaction between rs25531 and an environment on brain structures, many other studies revealed that the wild allele (A) of serotonin transporter genes could be considered a risk allele and be associated with thinner or smaller brain [87, 88].

In addition, DRD2 had an indirect effect on depression severity mediated through the triangular part of the inferior frontal gyrus (indirect effect = -.01, SE = .00, 95% CI = [-.02, -.00]). This indicates that mutation of the DRD2 gene may render the person less susceptible to depression through thickening the triangular part of the inferior frontal gyrus. This finding supports gene–brain–behaviour relationships of dopamine genes, which are known to be associated with neural changes in reward-related regions, which could play an essential role in the pathogenesis of depression [89]. Lastly, Table 6 shows how much variance of each dependent component is explained by its independent variables (average *R*^2^ = .20).

**Table 6:** R^2^ for all dependent variables that include ROIs and depression severity.

Dependent variables	R^2^	Dependent variables	R^2^
precentral gyrus	0.19	lateral occipito-temporal gyrus (fusiform gyrus, O4-T4)	0.11
subcentral gyrus	0.22	lingual gyrus (O5)	0.18
inferior frontal gyrus (or F3)	0.18	parahippocampal gyrus (or T5)	0.11
triangular part of the inferior frontal gyrus	0.30	cuneus (O6)	0.13
opercular part of the inferior frontal gyrus	0.30	occipital pole	0.09
orbital part of the inferior frontal gyrus	0.29	temporal pole	0.12
middle frontal gyrus (or F2)	0.27	postcentral gyrus	0.23
superior frontal gyrus	0.33	supramarginal gyrus	0.25
gyrus rectus	0.22	angular gyrus	0.28
transverse frontopolar gyrus or gyri	0.23	superior parietal lobule (or P1)	0.23
medial orbital sulcus	0.21	precuneus	0.20
4 orbital gyri	0.27	paracentral lobule and sulcus	0.23
superior circular sulcus of the insula	0.18	subcentral gyrus and sulci	0.22
anterior circular sulcus of the insula	0.13	Marginal branch (or part) of the cingulate sulcus	0.23
inferior circular sulcus of the insula	0.31	subparietal sulcus	0.11
vertical ramus of anterior segment of lateral sulcus	0.18	calcarine sulcus	0.27
horizontal ramus of anterior segment of lateral sulcus	0.16	medial occipitotemporal sulcus (or collateral sulcus)	0.25
posterior segment of the lateral sulcus	0.26	lateral occipito-temporal (or fusiform) sulcus	0.11
the short insular gyri	0.15	subcallosal area or gyrus	0.11
long insular gyrus	0.22	pericallosal sulcus or sulcus of the corpus callosum	0.08
transverse temporal gyrus (or Heschl’s gyrus)	0.15	anterior part of the cingulate gyrus and sulcus (ACC)	0.17
Planum temporale or temporal plane of the superior temporal gyrus	0.23	middle-anterior part of the cingulate gyrus and sulcus (aMCC)	0.21
Planum polare of the superior temporal gyrus	0.20	middle-posterior part of the cingulate gyrus and sulcus (pMCC)	0.22
lateral aspect of the superior temporal gyrus	0.18	posterior-dorsal part of the cingulate gyrus and sulcus (dPCC)	0.19
middle temporal gyrus	0.17	posterior-ventral part of the cingulate gyrus and sulcus (vPCC or isthmus)	0.18
superior occipital gyrus (O1)	0.09	Depression	0.27
middle occipital gyrus (O2, lateral occipital gyrus)	0.09	Average R^2^	0.20
Inferior temporal gyrus (T3)	0.11		

## Simulation study

**Fig 3:**
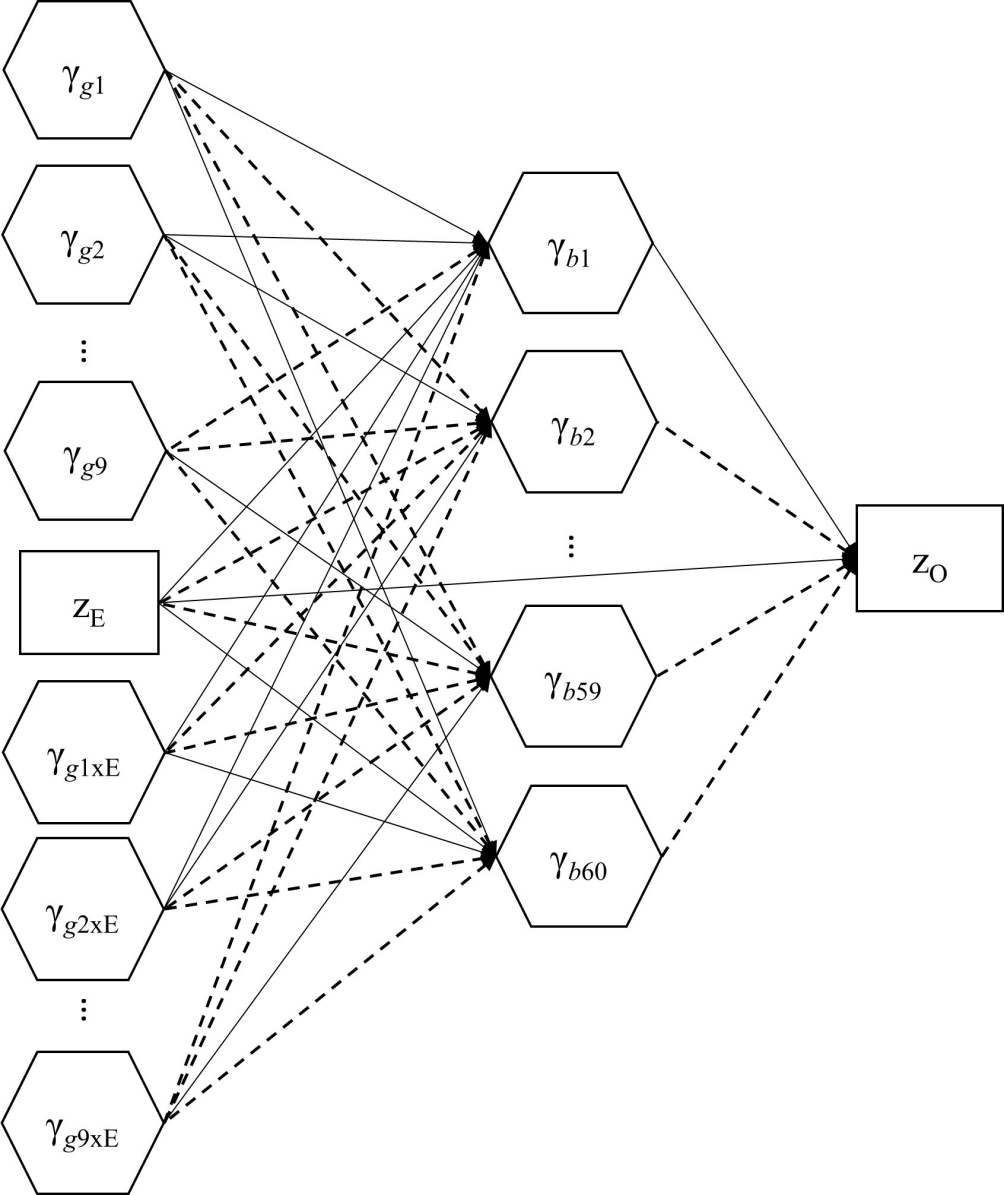
The structural model specified for the simulations study. All weights and residual terms are omitted. A non-zero path coefficient is denoted by an arrow, whereas a zero path coefficient is by a dashed arrow.

We conducted a simulation study to examine whether IG-GSCA could perform as expected, particularly in terms of parameter recovery. In this study, we contemplated a model that was quite similar to, yet on a slightly larger scale, the one specified in the real data analysis. As displayed in Fig 3, the model included nine genes, which were associated with one, two, or four SNPs, and sixty brain ROIs, each of which was linked to two brain-level observed variables. It also included an independent observed variable representing an environmental variable. The genes and environmental variable were specified to affect the 60 ROIs, which in turn were to influence an outcome variable that represents a behavioral or cognitive variable of interest. The environmental variable also had a direct effect on the outcome variable. The model further included the interaction term of each gene and the environmental variable (i.e., a total of nine gene-environment interaction terms), which influenced each ROI. In the model, a zero path coefficient is denoted by a dashed arrow.

We considered four levels of sample size (*N* = 250, 500, 1000, and 2000), for each of which we drew 1000 samples randomly based on a data generating procedure, whose detailed description is provided in S1 Appendix. As in the real data analysis, we applied ridge-type regularization based on five-fold cross validation.

As parameter recovery measures, we calculated finite-sample properties, such as bias, standard deviation (SD), and root mean square error (RMSE), of the IG-GSCA parameter estimates. To conserve space, we focus here on reporting the average values of these properties for loading and path coefficient estimates per sample size. All the properties of individual parameter estimates are provided in S2 Table.

Table 7 presents the average bias, SD, and RMSE values of loading and path coefficient estimates per sample size. On average, the biases of the loading estimates for both sets of components (i.e., genes and ROIs) were virtually zero across all sample sizes, and their SD and RMSE values deceased and became close to zero as the sample size increased. On the other hand, in general, IG-GSCA’s estimates for non-zero path coefficients seemed to be slightly biased in smaller samples. This seems to be due to the adoption of ridge-type regularization, which tends to yield biased estimates particularly in small samples [44]. Nonetheless, their average bias decreased with the sample size and became close to zero when *N* = 2000. This tendency is also expected because multicollinearity can be of less concern in large samples. The average SD and RMSE values of the path coefficient estimates also decreased when the sample size increased. IG-GSCA’s estimates for zero path coefficients were unbiased regardless of the sample size and their SD and RMSE deceased when the sample size increased.

**Table 7:** Average biases, Standard Deviations (SD), and Root Mean Square Errors (RMSE) of loadings and path coefficients estimated from IG-GSCA over different sample sizes in the simulation study.

Loadings		Loadings for Genes			Loadings for ROIs		
		Bias	SD	RMSE	Bias	SD	RMSE
	N = 250	0.001	0.020	0.020	0.001	0.013	0.013
	N = 500	0.001	0.014	0.014	0.001	0.009	0.009
	N = 1000	0.000	0.010	0.010	0.000	0.006	0.006
	N = 2000	0.000	0.007	0.007	0.000	0.004	0.004
Path coefficients		Effects of Genes and Environment on ROIs					
		Zeros			Nonzeros		
		Bias	SD	RMSE	Bias	SD	RMSE
	N = 250	0.003	0.042	0.042	0.032	0.043	0.055
	N = 500	0.002	0.030	0.030	0.017	0.033	0.038
	N = 1000	0.002	0.021	0.022	0.008	0.024	0.026
	N = 2000	0.002	0.015	0.015	0.004	0.022	0.022
		Effects of Gene-Environment Interactions on ROIs					
		Zeros			Nonzeros		
		Bias	SD	RMSE	Bias	SD	RMSE
	N = 250	0.003	0.042	0.043	0.027	0.046	0.054
	N = 500	0.002	0.030	0.030	0.014	0.035	0.038
	N = 1000	0.002	0.021	0.021	0.007	0.025	0.026
	N = 2000	0.002	0.015	0.015	0.004	0.022	0.022
		Effects of ROIs on Outcome					
		Zeros			Nonzeros		
		Bias	SD	RMSE	Bias	SD	RMSE
	N = 250	0.007	0.049	0.050	0.057	0.052	0.078
	N = 500	0.004	0.037	0.037	0.028	0.041	0.050
	N = 1000	0.002	0.027	0.027	0.013	0.029	0.031
	N = 2000	0.002	0.019	0.019	0.006	0.022	0.022

## Conclusions

We proposed a flexible statistical approach, named IG-GSCA, for examining the associations among genetic, imaging and behavioral/cognitive data in a unified manner. As demonstrated in Section 3, IG-GSCA was able to specify complex directional relationships among genes, ROIs, and depression severity in a more biologically plausible way based on previous knowledge, and to identify the influences of a gene (DRD2) and a gene-environment interaction (PTE x SLC6A4) on several brain regions, which in turn affected depression severity. In addition, our simulation study showed that IG-GSCA performed as expected in terms of parameter recovery under a model similar to the one specified for the real data analysis.

IG-GSCA can be a useful tool for researchers in imaging genetics to study the neurobiological basis of individual behavioral or cognitive differences, addressing various issues inherent to current multivariate methodologies (e.g., less biologically interpretable, descriptive, or sequential). It also has the potential to inform clinicians about specific genetic or brain-level vulnerabilities associated with risk for chronic diseases later in life, which are proxied by individuals’ genetic or imaging component scores in disease-specific imaging genetics models.

Despite its technical and empirical implications, IG-GSCA can be refined and extended in many ways to further enhance its generality and flexibility. For example, genetic and imaging data can often be hierarchically structured such that their individual-level cases are grouped within higher-level units. For example, individuals’ genetic variation and brain activity can be measured across different experimental groups or time points. In such hierarchical/multilevel data, the individual-level measures nested within the same higher-level unit are likely to be more similar than those in different units, thus leading to dependency among individual-level measures within the same unit. Ignoring this dependency in parameter estimation can yield biased results [90]. In its base form, IG-GSCA will estimate parameters under the assumption that all observations are independent, ignoring potential nested structures in genetic and imaging data. It can be extended to explicitly account for such nested structures by permitting parameters to vary across higher-level units.

In addition, IG-GSCA currently posits that a component is always associated with a set of observed variables (e.g., a gene with SNPs, or a brain region with voxels). This type of component is called a first-order component, which is directly linked to observed variables [18]. In genetic studies, it may also be reasonable to assume that multiple genes in turn constitute a biological pathway [20]. Then, such a pathway can be seen as a ‘second-order’ component, which is related only to their first-order components (genes). In neuroimaging studies, it becomes common to utilize multiple neuroimaging modalities (e.g., structured magnetic resonance imaging, electroencephalography, etc.) to measure activities of brain regions. In this case, we may consider higher-order components integrated over brain regions from each modality [91]. IG-GSCA can be extended to take into account such higher-order genetic or imaging components.

Furthermore, imaging data have increasingly been treated as smooth functions or curves that vary over a continuum (e.g., time and/or space), rather than conventional multivariate data (a collection of discrete observations). For example, functional magnetic resonance imaging records blood-oxygen level dependent signals per voxel continuously over a great number of time points (scans), indicating that these signals can be represented as bivariate functions of time (scans) and space (voxels) [92, 93]. Similarly, SNPs have been considered smooth functions of space (physical positions) [94]. IG-GSCA is geared only for the analysis of multivariate data. It can be generalized to the analysis of genetic and imaging data as functions in the measurement model, accounting for their distinctive characteristics (e.g., smoothness), in a way similar to functional GSCA [95].

IG-GSCA in this paper has not paid attention to the analysis of longitudinal data. For example, it does not take into account the dynamic nature of temporally (serially) correlated data that are prevalent particularly in brain connectivity studies [16]. IG-GSCA may be extended to incorporate autoregressive modeling to consider the dynamic relationships in time series data, as proposed in dynamic GSCA [19]. Moreover, IG-GSCA can be readily extended to accommodate growth curve models [96, 97], as GSCA can deal with the same models [18].

IG-GSCA currently estimates parameters by aggregating the data across observations under the implicit assumption that all observations come from a single homogenous population. In some cases, however, it may be more reasonable to assume that observations are drawn from (unknown) heterogeneous subgroups in the population, which exhibit different path-analytic relationships among observed variables and components [98–100]. Thus, future work is needed to simultaneously combine IG-GSCA with cluster analysis to capture such cluster-level heterogeneity, inspired by the development of fuzzy clusterwise GSCA [98].

In closing, IG-GSCA can be a useful tool for imaging genetic studies that aim to associate both genetic and imaging data with behavioral/cognitive outcomes simultaneously. It is more general than regression models, enabling to combine SNPs to genes and voxels to brain regions and examine various gene-brain-behavior/cognition relationships, in a biologically plausible manner. Also, this approach can be more beneficial for such complex path-analytic association studies of the three sources of data, as compared to (factor-based) SEM. Although we have discussed several limitations of IG-GSCA, we may address these technical issues in future research by adapting many prior developments in GSCA, contributing to making IG-GSCA applicable and useful for a greater variety of imaging genetic studies. In addition, we hope to develop a software program for IG-GSCA in a user-friendly format, such as an R package, in the near future. This will make the approach more accessible to researchers in imaging genetics, facilitating its applications to more diverse real-world problems and more thorough investigations of its empirical utility in the field.

## Supporting information

S1 Table The entire path coefficient estimates, their standard errors and 95% confidence intervals (direct effects only) in the empirical study. (DOCX)

S2 Table Biases, Standard Deviations (SD), and Root Mean Square Errors (RMSE) of loadings and path coefficients estimated from IG-GSCA over different sample sizes in the simulation study. (DOCX)

S1 Appendix The data generation procedure for the simulation study. (DOCX)
