# Supplementary material for: A knowledge-based multivariate statistical method for examining gene-brain-behavioral/cognitive relationships: Imaging genetics generalized structured component analysis
Source: PLoS One. 2021 Mar 10;16(3):e0247592. doi: 10.1371/journal.pone.0247592 (PMC7946325; doi:10.1371/journal.pone.0247592)
Supplement: S1 Table — (DOCX) [file pone.0247592.s001.docx]

S1 Table. The entire path coefficient estimates, their standard errors and 95% confidence intervals (direct effects only) in the empirical study. * implies the path coefficient estimate in the row is significant.

| **Path coefficients** | |  | **Estimate** | **SE** | **95% CI** | |
| --- | --- | --- | --- | --- | --- | --- |
| SLC6A4 (gene) | precentral gyrus |  | -0.02 | 0.04 | -0.10 | 0.04 |
| SLC6A4 (gene) | subcentral gyrus |  | 0.02 | 0.04 | -0.05 | 0.09 |
| SLC6A4 (gene) | inferior frontal gyrus (or F3) |  | 0.03 | 0.04 | -0.05 | 0.10 |
| SLC6A4 (gene) | triangular part of the inferior frontal gyrus |  | 0.01 | 0.04 | -0.06 | 0.08 |
| SLC6A4 (gene) | opercular part of the inferior frontal gyrus | * | 0.07 | 0.03 | 0.00 | 0.14 |
| SLC6A4 (gene) | orbital part | * | -0.07 | 0.03 | -0.13 | 0.00 |
| SLC6A4 (gene) | middle frontal gyrus (or F2) |  | 0.01 | 0.04 | -0.07 | 0.08 |
| SLC6A4 (gene) | superior frontal gyrus |  | 0.01 | 0.03 | -0.06 | 0.08 |
| SLC6A4 (gene) | gyrus rectus |  | 0.03 | 0.04 | -0.05 | 0.10 |
| SLC6A4 (gene) | transverse frontopolar gyrus or gyri |  | 0.01 | 0.04 | -0.05 | 0.08 |
| SLC6A4 (gene) | medial orbital sulcus |  | -0.01 | 0.03 | -0.08 | 0.06 |
| SLC6A4 (gene) | 4 orbital gyri |  | -0.04 | 0.04 | -0.11 | 0.03 |
| SLC6A4 (gene) | superior (circular sulcus of the insula) |  | 0.00 | 0.04 | -0.07 | 0.08 |
| SLC6A4 (gene) | anterior (circular sulcus of the insula) |  | -0.03 | 0.04 | -0.11 | 0.05 |
| SLC6A4 (gene) | inferior (circular sulcus of the insula) |  | 0.00 | 0.03 | -0.05 | 0.06 |
| SLC6A4 (gene) | vertical ramus of anterior segment of lateral sulcus |  | 0.03 | 0.04 | -0.05 | 0.10 |
| SLC6A4 (gene) | horizontal ramus of anterior segment of lateral sulcus |  | -0.04 | 0.04 | -0.10 | 0.04 |
| SLC6A4 (gene) | posterior segment of the lateral sulcus |  | 0.02 | 0.03 | -0.05 | 0.09 |
| SLC6A4 (gene) | the short insular gyri |  | 0.04 | 0.04 | -0.02 | 0.11 |
| SLC6A4 (gene) | long insular gyrus |  | 0.05 | 0.04 | -0.03 | 0.12 |
| SLC6A4 (gene) | transverse temporal gyrus (or Heschl's gyrus) |  | 0.01 | 0.04 | -0.07 | 0.08 |
| SLC6A4 (gene) | planum temporale or temporal plane of the superior temporal gyrus |  | 0.01 | 0.04 | -0.07 | 0.08 |
| SLC6A4 (gene) | planum polare of the superior temporal gyrus |  | 0.05 | 0.04 | -0.02 | 0.13 |
| SLC6A4 (gene) | lateral aspect of the superior temporal gyrus |  | 0.05 | 0.04 | -0.02 | 0.13 |
| SLC6A4 (gene) | middle temporal gyrus |  | 0.04 | 0.04 | -0.03 | 0.12 |
| SLC6A4 (gene) | superior occipital gyrus (O1) |  | 0.02 | 0.04 | -0.06 | 0.10 |
| SLC6A4 (gene) | middle occipital gyrus (O2, lateral occipital gyrus) |  | 0.02 | 0.04 | -0.06 | 0.09 |
| SLC6A4 (gene) | inferior temporal gyrus (T3) |  | 0.00 | 0.04 | -0.08 | 0.08 |
| SLC6A4 (gene) | lateral occipito-temporal gyrus (fusiform gyrus, O4-T4) |  | -0.01 | 0.04 | -0.08 | 0.07 |
| SLC6A4 (gene) | lingual gyrus (O5) |  | 0.05 | 0.04 | -0.03 | 0.13 |
| SLC6A4 (gene) | parahippocampal gyrus (or T5) |  | 0.01 | 0.04 | -0.07 | 0.08 |
| SLC6A4 (gene) | cuneus (O6) |  | 0.02 | 0.04 | -0.06 | 0.10 |
| SLC6A4 (gene) | occipital pole |  | -0.01 | 0.04 | -0.08 | 0.06 |
| SLC6A4 (gene) | temporal pole |  | 0.05 | 0.05 | -0.04 | 0.14 |
| SLC6A4 (gene) | postcentral gyrus |  | 0.00 | 0.04 | -0.06 | 0.07 |
| SLC6A4 (gene) | supramarginal gyrus |  | -0.03 | 0.04 | -0.11 | 0.04 |
| SLC6A4 (gene) | angular gyrus |  | 0.03 | 0.04 | -0.04 | 0.10 |
| SLC6A4 (gene) | superior parietal lobule (or P1) |  | 0.05 | 0.04 | -0.03 | 0.12 |
| SLC6A4 (gene) | precuneus |  | 0.03 | 0.04 | -0.04 | 0.11 |
| SLC6A4 (gene) | paracentral lobule and sulcus |  | 0.03 | 0.04 | -0.04 | 0.09 |
| SLC6A4 (gene) | subcentral gyrus and sulci |  | 0.02 | 0.04 | -0.05 | 0.09 |
| SLC6A4 (gene) | marginal branch (or part) of the cingulate sulcus |  | -0.01 | 0.04 | -0.08 | 0.06 |
| SLC6A4 (gene) | subparietal sulcus |  | -0.02 | 0.04 | -0.10 | 0.06 |
| SLC6A4 (gene) | calcarine sulcus |  | 0.05 | 0.04 | -0.03 | 0.13 |
| SLC6A4 (gene) | medial occipitotemporalsulcus (or collateral sulcus) | * | 0.10 | 0.04 | 0.02 | 0.18 |
| SLC6A4 (gene) | lateral occipito-temporal (or fusiform) sulcus |  | 0.03 | 0.04 | -0.04 | 0.10 |
| SLC6A4 (gene) | subcallosal area or gyrus |  | -0.01 | 0.04 | -0.09 | 0.06 |
| SLC6A4 (gene) | pericallosal sulcus or sulcus of the corpus callosum |  | 0.02 | 0.04 | -0.06 | 0.10 |
| SLC6A4 (gene) | anterior (ACC) |  | -0.01 | 0.04 | -0.08 | 0.07 |
| SLC6A4 (gene) | middle-anterior (aMCC) |  | 0.00 | 0.03 | -0.06 | 0.07 |
| SLC6A4 (gene) | middle-posterior (pMCC) |  | 0.05 | 0.04 | -0.02 | 0.12 |
| SLC6A4 (gene) | posterior-dorsal (dPCC) |  | -0.04 | 0.04 | -0.11 | 0.04 |
| SLC6A4 (gene) | posterior-ventral (vPCC or isthmus) |  | 0.00 | 0.04 | -0.08 | 0.09 |
| FKBP5 (gene) | precentral gyrus |  | -0.05 | 0.04 | -0.12 | 0.03 |
| FKBP5 (gene) | subcentral gyrus |  | 0.01 | 0.04 | -0.07 | 0.08 |
| FKBP5 (gene) | inferior frontal gyrus (or F3) |  | -0.06 | 0.03 | -0.13 | 0.01 |
| FKBP5 (gene) | triangular part of the inferior frontal gyrus |  | -0.01 | 0.03 | -0.07 | 0.06 |
| FKBP5 (gene) | opercular part of the inferior frontal gyrus |  | -0.01 | 0.04 | -0.08 | 0.07 |
| FKBP5 (gene) | orbital part |  | -0.01 | 0.03 | -0.08 | 0.05 |
| FKBP5 (gene) | middle frontal gyrus (or F2) |  | -0.03 | 0.04 | -0.10 | 0.04 |
| FKBP5 (gene) | superior frontal gyrus |  | 0.00 | 0.03 | -0.06 | 0.07 |
| FKBP5 (gene) | gyrus rectus |  | 0.03 | 0.04 | -0.05 | 0.10 |
| FKBP5 (gene) | transverse frontopolar gyrus or gyri |  | -0.06 | 0.03 | -0.12 | 0.01 |
| FKBP5 (gene) | medial orbital sulcus |  | 0.02 | 0.04 | -0.05 | 0.09 |
| FKBP5 (gene) | 4 orbital gyri |  | -0.03 | 0.04 | -0.11 | 0.04 |
| FKBP5 (gene) | superior (circular sulcus of the insula) |  | 0.02 | 0.04 | -0.05 | 0.09 |
| FKBP5 (gene) | anterior (circular sulcus of the insula) |  | -0.01 | 0.04 | -0.09 | 0.06 |
| FKBP5 (gene) | inferior (circular sulcus of the insula) |  | 0.03 | 0.03 | -0.03 | 0.10 |
| FKBP5 (gene) | vertical ramus of anterior segment of lateral sulcus |  | -0.06 | 0.03 | -0.13 | 0.01 |
| FKBP5 (gene) | horizontal ramus of anterior segment of lateral sulcus |  | 0.05 | 0.04 | -0.03 | 0.12 |
| FKBP5 (gene) | posterior segment of the lateral sulcus |  | 0.02 | 0.04 | -0.05 | 0.09 |
| FKBP5 (gene) | the short insular gyri |  | 0.02 | 0.04 | -0.06 | 0.09 |
| FKBP5 (gene) | long insular gyrus |  | 0.00 | 0.04 | -0.07 | 0.07 |
| FKBP5 (gene) | transverse temporal gyrus (or Heschl's gyrus) |  | -0.05 | 0.04 | -0.12 | 0.03 |
| FKBP5 (gene) | planum temporale or temporal plane of the superior temporal gyrus |  | 0.01 | 0.04 | -0.06 | 0.09 |
| FKBP5 (gene) | planum polare of the superior temporal gyrus |  | -0.05 | 0.04 | -0.13 | 0.02 |
| FKBP5 (gene) | lateral aspect of the superior temporal gyrus |  | 0.01 | 0.04 | -0.08 | 0.08 |
| FKBP5 (gene) | middle temporal gyrus |  | 0.00 | 0.04 | -0.08 | 0.07 |
| FKBP5 (gene) | superior occipital gyrus (O1) |  | -0.02 | 0.04 | -0.09 | 0.05 |
| FKBP5 (gene) | middle occipital gyrus (O2, lateral occipital gyrus) |  | 0.00 | 0.04 | -0.08 | 0.08 |
| FKBP5 (gene) | inferior temporal gyrus (T3) |  | -0.03 | 0.04 | -0.10 | 0.04 |
| FKBP5 (gene) | lateral occipito-temporal gyrus (fusiform gyrus, O4-T4) |  | 0.03 | 0.04 | -0.04 | 0.11 |
| FKBP5 (gene) | lingual gyrus (O5) |  | 0.04 | 0.04 | -0.03 | 0.12 |
| FKBP5 (gene) | parahippocampal gyrus (or T5) |  | 0.02 | 0.04 | -0.05 | 0.10 |
| FKBP5 (gene) | cuneus (O6) |  | 0.05 | 0.04 | -0.03 | 0.13 |
| FKBP5 (gene) | occipital pole |  | 0.04 | 0.04 | -0.03 | 0.11 |
| FKBP5 (gene) | temporal pole |  | 0.01 | 0.04 | -0.07 | 0.09 |
| FKBP5 (gene) | postcentral gyrus |  | -0.04 | 0.04 | -0.11 | 0.03 |
| FKBP5 (gene) | supramarginal gyrus |  | -0.02 | 0.04 | -0.10 | 0.05 |
| FKBP5 (gene) | angular gyrus |  | 0.00 | 0.04 | -0.07 | 0.07 |
| FKBP5 (gene) | superior parietal lobule (or P1) |  | 0.00 | 0.04 | -0.07 | 0.07 |
| FKBP5 (gene) | precuneus |  | 0.02 | 0.03 | -0.05 | 0.08 |
| FKBP5 (gene) | paracentral lobule and sulcus |  | 0.01 | 0.04 | -0.06 | 0.08 |
| FKBP5 (gene) | subcentral gyrus and sulci |  | 0.01 | 0.04 | -0.07 | 0.08 |
| FKBP5 (gene) | marginal branch (or part) of the cingulate sulcus |  | 0.02 | 0.04 | -0.05 | 0.10 |
| FKBP5 (gene) | subparietal sulcus |  | 0.04 | 0.03 | -0.03 | 0.11 |
| FKBP5 (gene) | calcarine sulcus |  | 0.03 | 0.03 | -0.04 | 0.09 |
| FKBP5 (gene) | medial occipitotemporal sulcus (or collateral sulcus) |  | 0.05 | 0.03 | -0.02 | 0.11 |
| FKBP5 (gene) | lateral occipito-temporal (or fusiform) sulcus |  | 0.00 | 0.04 | -0.08 | 0.08 |
| FKBP5 (gene) | subcallosal area or gyrus |  | -0.03 | 0.04 | -0.10 | 0.05 |
| FKBP5 (gene) | pericallosal sulcus or sulcus of the corpus callosum |  | 0.00 | 0.04 | -0.08 | 0.07 |
| FKBP5 (gene) | anterior (ACC) |  | 0.00 | 0.04 | -0.07 | 0.07 |
| FKBP5 (gene) | middle-anterior (aMCC) |  | 0.06 | 0.03 | 0.00 | 0.13 |
| FKBP5 (gene) | middle-posterior (pMCC) |  | 0.05 | 0.03 | -0.02 | 0.12 |
| FKBP5 (gene) | posterior-dorsal (dPCC) |  | 0.04 | 0.04 | -0.03 | 0.12 |
| FKBP5 (gene) | posterior-ventral (vPCC or isthmus) |  | 0.02 | 0.04 | -0.05 | 0.09 |
| ADCYAP1R1 (gene) | precentral gyrus |  | 0.01 | 0.03 | -0.06 | 0.08 |
| ADCYAP1R1 (gene) | subcentral gyrus |  | 0.02 | 0.04 | -0.05 | 0.09 |
| ADCYAP1R1 (gene) | inferior frontal gyrus (or F3) |  | -0.04 | 0.04 | -0.11 | 0.03 |
| ADCYAP1R1 (gene) | triangular part of the inferior frontal gyrus |  | -0.03 | 0.04 | -0.11 | 0.04 |
| ADCYAP1R1 (gene) | opercular part of the inferior frontal gyrus |  | 0.03 | 0.03 | -0.03 | 0.10 |
| ADCYAP1R1 (gene) | orbital part |  | -0.03 | 0.04 | -0.11 | 0.04 |
| ADCYAP1R1 (gene) | middle frontal gyrus (or F2) |  | 0.04 | 0.04 | -0.03 | 0.11 |
| ADCYAP1R1 (gene) | superior frontal gyrus |  | 0.04 | 0.03 | -0.02 | 0.10 |
| ADCYAP1R1 (gene) | gyrus rectus |  | -0.03 | 0.04 | -0.11 | 0.05 |
| ADCYAP1R1 (gene) | transverse frontopolar gyrus or gyri |  | -0.03 | 0.04 | -0.11 | 0.04 |
| ADCYAP1R1 (gene) | medial orbital sulcus | * | -0.10 | 0.04 | -0.17 | -0.02 |
| ADCYAP1R1 (gene) | 4 orbital gyri |  | -0.01 | 0.04 | -0.08 | 0.06 |
| ADCYAP1R1 (gene) | superior (circular sulcus of the insula) |  | -0.04 | 0.04 | -0.12 | 0.03 |
| ADCYAP1R1 (gene) | anterior (circular sulcus of the insula) |  | 0.02 | 0.04 | -0.06 | 0.10 |
| ADCYAP1R1 (gene) | inferior (circular sulcus of the insula) |  | -0.04 | 0.04 | -0.12 | 0.03 |
| ADCYAP1R1 (gene) | vertical ramus of anterior segment of lateral sulcus |  | -0.04 | 0.04 | -0.11 | 0.03 |
| ADCYAP1R1 (gene) | horizontal ramus of anterior segment of lateral sulcus |  | -0.05 | 0.04 | -0.13 | 0.02 |
| ADCYAP1R1 (gene) | posterior segment of the lateral sulcus |  | -0.02 | 0.03 | -0.09 | 0.05 |
| ADCYAP1R1 (gene) | the short insular gyri |  | -0.02 | 0.04 | -0.09 | 0.06 |
| ADCYAP1R1 (gene) | long insular gyrus | * | -0.07 | 0.04 | -0.15 | 0.00 |
| ADCYAP1R1 (gene) | transverse temporal gyrus (or Heschl's gyrus) |  | -0.02 | 0.04 | -0.09 | 0.05 |
| ADCYAP1R1 (gene) | planum temporale or temporal plane of the superior temporal gyrus |  | -0.01 | 0.04 | -0.07 | 0.06 |
| ADCYAP1R1 (gene) | planum polare of the superior temporal gyrus |  | -0.02 | 0.04 | -0.09 | 0.05 |
| ADCYAP1R1 (gene) | lateral aspect of the superior temporal gyrus |  | -0.01 | 0.04 | -0.08 | 0.06 |
| ADCYAP1R1 (gene) | middle temporal gyrus |  | 0.01 | 0.04 | -0.07 | 0.08 |
| ADCYAP1R1 (gene) | superior occipital gyrus (O1) |  | 0.01 | 0.04 | -0.08 | 0.09 |
| ADCYAP1R1 (gene) | middle occipital gyrus (O2, lateral occipital gyrus) |  | 0.01 | 0.04 | -0.07 | 0.07 |
| ADCYAP1R1 (gene) | inferior temporal gyrus (T3) |  | -0.02 | 0.04 | -0.10 | 0.05 |
| ADCYAP1R1 (gene) | lateral occipito-temporal gyrus (fusiform gyrus, O4-T4) |  | -0.01 | 0.04 | -0.08 | 0.07 |
| ADCYAP1R1 (gene) | lingual gyrus (O5) |  | -0.01 | 0.04 | -0.08 | 0.06 |
| ADCYAP1R1 (gene) | parahippocampal gyrus (or T5) |  | 0.00 | 0.04 | -0.08 | 0.08 |
| ADCYAP1R1 (gene) | cuneus (O6) |  | 0.01 | 0.04 | -0.07 | 0.09 |
| ADCYAP1R1 (gene) | occipital pole |  | -0.05 | 0.04 | -0.13 | 0.03 |
| ADCYAP1R1 (gene) | temporal pole |  | -0.04 | 0.04 | -0.11 | 0.04 |
| ADCYAP1R1 (gene) | postcentral gyrus |  | 0.03 | 0.04 | -0.04 | 0.10 |
| ADCYAP1R1 (gene) | supramarginal gyrus |  | 0.02 | 0.03 | -0.05 | 0.09 |
| ADCYAP1R1 (gene) | angular gyrus |  | 0.03 | 0.04 | -0.04 | 0.10 |
| ADCYAP1R1 (gene) | superior parietal lobule (or P1) |  | 0.06 | 0.04 | -0.01 | 0.13 |
| ADCYAP1R1 (gene) | precuneus |  | 0.06 | 0.04 | -0.01 | 0.13 |
| ADCYAP1R1 (gene) | paracentral lobule and sulcus |  | 0.03 | 0.03 | -0.04 | 0.09 |
| ADCYAP1R1 (gene) | subcentral gyrus and sulci |  | 0.02 | 0.04 | -0.05 | 0.09 |
| ADCYAP1R1 (gene) | marginal branch (or part) of the cingulate sulcus | * | 0.07 | 0.04 | 0.00 | 0.14 |
| ADCYAP1R1 (gene) | subparietal sulcus |  | 0.01 | 0.04 | -0.07 | 0.09 |
| ADCYAP1R1 (gene) | calcarine sulcus |  | 0.01 | 0.03 | -0.06 | 0.07 |
| ADCYAP1R1 (gene) | medial occipitotemporal sulcus (or collateral sulcus) |  | 0.01 | 0.04 | -0.06 | 0.09 |
| ADCYAP1R1 (gene) | lateral occipito-temporal (or fusiform) sulcus |  | 0.04 | 0.04 | -0.04 | 0.11 |
| ADCYAP1R1 (gene) | subcallosal area or gyrus |  | -0.07 | 0.04 | -0.15 | 0.00 |
| ADCYAP1R1 (gene) | pericallosal sulcus or sulcus of the corpus callosum |  | -0.03 | 0.04 | -0.11 | 0.05 |
| ADCYAP1R1 (gene) | anterior (ACC) |  | -0.04 | 0.04 | -0.12 | 0.04 |
| ADCYAP1R1 (gene) | middle-anterior (aMCC) |  | 0.00 | 0.04 | -0.09 | 0.08 |
| ADCYAP1R1 (gene) | middle-posterior (pMCC) |  | -0.03 | 0.03 | -0.10 | 0.04 |
| ADCYAP1R1 (gene) | posterior-dorsal (dPCC) |  | 0.01 | 0.04 | -0.06 | 0.08 |
| ADCYAP1R1 (gene) | posterior-ventral (vPCC or isthmus) |  | -0.01 | 0.04 | -0.09 | 0.07 |
| BDNF (gene) | precentral gyrus |  | 0.01 | 0.04 | -0.06 | 0.08 |
| BDNF (gene) | subcentral gyrus |  | 0.01 | 0.03 | -0.05 | 0.08 |
| BDNF (gene) | inferior frontal gyrus (or F3) |  | 0.04 | 0.04 | -0.03 | 0.11 |
| BDNF (gene) | triangular part of the inferior frontal gyrus |  | 0.01 | 0.03 | -0.06 | 0.07 |
| BDNF (gene) | opercular part of the inferior frontal gyrus |  | 0.02 | 0.03 | -0.05 | 0.08 |
| BDNF (gene) | orbital part |  | 0.06 | 0.03 | -0.01 | 0.13 |
| BDNF (gene) | middle frontal gyrus (or F2) |  | -0.02 | 0.03 | -0.09 | 0.04 |
| BDNF (gene) | superior frontal gyrus |  | 0.01 | 0.03 | -0.05 | 0.07 |
| BDNF (gene) | gyrus rectus |  | 0.06 | 0.03 | 0.00 | 0.13 |
| BDNF (gene) | transverse frontopolar gyrus or gyri |  | 0.02 | 0.04 | -0.05 | 0.09 |
| BDNF (gene) | medial orbital sulcus |  | 0.03 | 0.03 | -0.04 | 0.10 |
| BDNF (gene) | 4 orbital gyri | * | 0.07 | 0.03 | 0.01 | 0.13 |
| BDNF (gene) | superior (circular sulcus of the insula) |  | 0.02 | 0.04 | -0.06 | 0.09 |
| BDNF (gene) | anterior (circular sulcus of the insula) |  | 0.06 | 0.04 | -0.02 | 0.14 |
| BDNF (gene) | inferior (circular sulcus of the insula) |  | 0.02 | 0.04 | -0.05 | 0.09 |
| BDNF (gene) | vertical ramus of anterior segment of lateral sulcus |  | 0.04 | 0.04 | -0.03 | 0.11 |
| BDNF (gene) | horizontal ramus of anterior segment of lateral sulcus |  | -0.01 | 0.04 | -0.08 | 0.06 |
| BDNF (gene) | posterior segment of the lateral sulcus |  | -0.02 | 0.04 | -0.08 | 0.05 |
| BDNF (gene) | the short insular gyri |  | 0.07 | 0.04 | -0.01 | 0.14 |
| BDNF (gene) | long insular gyrus |  | 0.03 | 0.04 | -0.04 | 0.10 |
| BDNF (gene) | transverse temporal gyrus (or Heschl's gyrus) |  | 0.01 | 0.04 | -0.06 | 0.08 |
| BDNF (gene) | planum temporale or temporal plane of the superior temporal gyrus |  | 0.05 | 0.04 | -0.02 | 0.13 |
| BDNF (gene) | planum polare of the superior temporal gyrus |  | 0.04 | 0.04 | -0.03 | 0.11 |
| BDNF (gene) | lateral aspect of the superior temporal gyrus |  | 0.03 | 0.04 | -0.06 | 0.10 |
| BDNF (gene) | middle temporal gyrus |  | 0.04 | 0.04 | -0.03 | 0.11 |
| BDNF (gene) | superior occipital gyrus (O1) | * | -0.07 | 0.04 | -0.15 | 0.00 |
| BDNF (gene) | middle occipital gyrus (O2, lateral occipital gyrus) |  | -0.01 | 0.04 | -0.08 | 0.07 |
| BDNF (gene) | inferior temporal gyrus (T3) |  | 0.04 | 0.04 | -0.04 | 0.11 |
| BDNF (gene) | lateral occipito-temporal gyrus (fusiform gyrus, O4-T4) |  | 0.00 | 0.04 | -0.08 | 0.07 |
| BDNF (gene) | lingual gyrus (O5) |  | -0.02 | 0.04 | -0.10 | 0.05 |
| BDNF (gene) | parahippocampal gyrus (or T5) |  | 0.02 | 0.04 | -0.07 | 0.10 |
| BDNF (gene) | cuneus (O6) |  | -0.03 | 0.04 | -0.11 | 0.05 |
| BDNF (gene) | occipital pole |  | -0.02 | 0.04 | -0.10 | 0.05 |
| BDNF (gene) | temporal pole | * | 0.08 | 0.04 | 0.00 | 0.16 |
| BDNF (gene) | postcentral gyrus |  | -0.01 | 0.03 | -0.07 | 0.06 |
| BDNF (gene) | supramarginal gyrus |  | 0.00 | 0.04 | -0.07 | 0.07 |
| BDNF (gene) | angular gyrus |  | 0.01 | 0.03 | -0.06 | 0.08 |
| BDNF (gene) | superior parietal lobule (or P1) |  | -0.01 | 0.04 | -0.09 | 0.06 |
| BDNF (gene) | precuneus |  | -0.02 | 0.04 | -0.09 | 0.05 |
| BDNF (gene) | paracentral lobule and sulcus |  | -0.01 | 0.04 | -0.08 | 0.06 |
| BDNF (gene) | subcentral gyrus and sulci |  | 0.01 | 0.03 | -0.05 | 0.08 |
| BDNF (gene) | marginal branch (or part) of the cingulate sulcus |  | -0.01 | 0.04 | -0.08 | 0.06 |
| BDNF (gene) | subparietal sulcus |  | 0.02 | 0.04 | -0.06 | 0.10 |
| BDNF (gene) | calcarine sulcus |  | -0.01 | 0.04 | -0.08 | 0.06 |
| BDNF (gene) | medial occipitotemporal sulcus (or collateral sulcus) |  | -0.02 | 0.04 | -0.10 | 0.05 |
| BDNF (gene) | lateral occipito-temporal (or fusiform) sulcus |  | 0.07 | 0.04 | -0.02 | 0.14 |
| BDNF (gene) | subcallosal area or gyrus | * | 0.09 | 0.04 | 0.01 | 0.16 |
| BDNF (gene) | pericallosal sulcus or sulcus of the corpus callosum |  | -0.02 | 0.05 | -0.11 | 0.06 |
| BDNF (gene) | anterior (ACC) |  | 0.03 | 0.04 | -0.05 | 0.11 |
| BDNF (gene) | middle-anterior (aMCC) |  | 0.05 | 0.04 | -0.03 | 0.12 |
| BDNF (gene) | middle-posterior (pMCC) |  | 0.02 | 0.04 | -0.05 | 0.09 |
| BDNF (gene) | posterior-dorsal (dPCC) |  | 0.01 | 0.04 | -0.07 | 0.08 |
| BDNF (gene) | posterior-ventral (vPCC or isthmus) |  | -0.04 | 0.04 | -0.11 | 0.03 |
| COMT (gene) | precentral gyrus |  | 0.08 | 0.04 | -0.01 | 0.15 |
| COMT (gene) | subcentral gyrus |  | -0.01 | 0.04 | -0.08 | 0.06 |
| COMT (gene) | inferior frontal gyrus (or F3) |  | 0.01 | 0.04 | -0.07 | 0.09 |
| COMT (gene) | triangular part of the inferior frontal gyrus |  | 0.00 | 0.03 | -0.06 | 0.07 |
| COMT (gene) | opercular part of the inferior frontal gyrus |  | 0.03 | 0.03 | -0.03 | 0.10 |
| COMT (gene) | orbital part |  | 0.01 | 0.03 | -0.06 | 0.07 |
| COMT (gene) | middle frontal gyrus (or F2) |  | -0.01 | 0.03 | -0.07 | 0.06 |
| COMT (gene) | superior frontal gyrus |  | -0.01 | 0.03 | -0.08 | 0.05 |
| COMT (gene) | gyrus rectus |  | -0.01 | 0.03 | -0.07 | 0.06 |
| COMT (gene) | transverse frontopolar gyrus or gyri |  | 0.00 | 0.03 | -0.06 | 0.07 |
| COMT (gene) | medial orbital sulcus |  | 0.02 | 0.04 | -0.05 | 0.09 |
| COMT (gene) | 4 orbital gyri |  | -0.02 | 0.03 | -0.08 | 0.05 |
| COMT (gene) | superior (circular sulcus of the insula) |  | 0.02 | 0.04 | -0.05 | 0.10 |
| COMT (gene) | anterior (circular sulcus of the insula) |  | 0.02 | 0.04 | -0.05 | 0.09 |
| COMT (gene) | inferior (circular sulcus of the insula) |  | 0.04 | 0.03 | -0.03 | 0.10 |
| COMT (gene) | vertical ramus of anterior segment of lateral sulcus |  | 0.01 | 0.04 | -0.07 | 0.09 |
| COMT (gene) | horizontal ramus of anterior segment of lateral sulcus |  | -0.02 | 0.04 | -0.09 | 0.06 |
| COMT (gene) | posterior segment of the lateral sulcus |  | 0.04 | 0.03 | -0.03 | 0.10 |
| COMT (gene) | the short insular gyri |  | 0.03 | 0.04 | -0.05 | 0.11 |
| COMT (gene) | long insular gyrus |  | 0.03 | 0.04 | -0.05 | 0.10 |
| COMT (gene) | transverse temporal gyrus (or Heschl's gyrus) |  | 0.02 | 0.04 | -0.06 | 0.09 |
| COMT (gene) | planum temporale or temporal plane of the superior temporal gyrus |  | -0.02 | 0.04 | -0.09 | 0.06 |
| COMT (gene) | planum polare of the superior temporal gyrus |  | 0.05 | 0.04 | -0.03 | 0.12 |
| COMT (gene) | lateral aspect of the superior temporal gyrus |  | -0.02 | 0.04 | -0.09 | 0.06 |
| COMT (gene) | middle temporal gyrus |  | -0.06 | 0.04 | -0.13 | 0.01 |
| COMT (gene) | superior occipital gyrus (O1) |  | -0.05 | 0.04 | -0.13 | 0.04 |
| COMT (gene) | middle occipital gyrus (O2, lateral occipital gyrus) |  | 0.00 | 0.04 | -0.09 | 0.08 |
| COMT (gene) | inferior temporal gyrus (T3) |  | -0.01 | 0.04 | -0.09 | 0.07 |
| COMT (gene) | lateral occipito-temporal gyrus (fusiform gyrus, O4-T4) |  | 0.02 | 0.04 | -0.05 | 0.10 |
| COMT (gene) | lingual gyrus (O5) |  | 0.04 | 0.04 | -0.03 | 0.12 |
| COMT (gene) | parahippocampal gyrus (or T5) |  | -0.05 | 0.04 | -0.13 | 0.03 |
| COMT (gene) | cuneus (O6) |  | 0.02 | 0.04 | -0.05 | 0.09 |
| COMT (gene) | occipital pole |  | 0.02 | 0.04 | -0.05 | 0.10 |
| COMT (gene) | temporal pole |  | 0.02 | 0.04 | -0.06 | 0.10 |
| COMT (gene) | postcentral gyrus |  | 0.00 | 0.04 | -0.07 | 0.08 |
| COMT (gene) | supramarginal gyrus |  | 0.01 | 0.04 | -0.06 | 0.08 |
| COMT (gene) | angular gyrus |  | -0.01 | 0.04 | -0.08 | 0.06 |
| COMT (gene) | superior parietal lobule (or P1) |  | 0.01 | 0.04 | -0.06 | 0.09 |
| COMT (gene) | precuneus |  | -0.01 | 0.04 | -0.08 | 0.07 |
| COMT (gene) | paracentral lobule and sulcus |  | 0.01 | 0.04 | -0.07 | 0.09 |
| COMT (gene) | subcentral gyrus and sulci |  | -0.01 | 0.04 | -0.08 | 0.06 |
| COMT (gene) | marginal branch (or part) of the cingulate sulcus |  | -0.05 | 0.04 | -0.12 | 0.02 |
| COMT (gene) | subparietal sulcus |  | -0.07 | 0.04 | -0.15 | 0.00 |
| COMT (gene) | calcarine sulcus |  | -0.04 | 0.03 | -0.11 | 0.03 |
| COMT (gene) | medial occipitotemporal sulcus (or collateral sulcus) |  | 0.02 | 0.04 | -0.05 | 0.10 |
| COMT (gene) | lateral occipito-temporal (or fusiform) sulcus |  | -0.03 | 0.04 | -0.11 | 0.05 |
| COMT (gene) | subcallosal area or gyrus |  | 0.03 | 0.04 | -0.04 | 0.11 |
| COMT (gene) | pericallosal sulcus or sulcus of the corpus callosum |  | -0.03 | 0.04 | -0.11 | 0.04 |
| COMT (gene) | anterior (ACC) |  | -0.01 | 0.04 | -0.08 | 0.06 |
| COMT (gene) | middle-anterior (aMCC) |  | 0.00 | 0.04 | -0.08 | 0.06 |
| COMT (gene) | middle-posterior (pMCC) |  | 0.02 | 0.04 | -0.05 | 0.09 |
| COMT (gene) | posterior-dorsal (dPCC) |  | 0.02 | 0.04 | -0.05 | 0.09 |
| COMT (gene) | posterior-ventral (vPCC or isthmus) |  | 0.00 | 0.04 | -0.08 | 0.07 |
| HTR3A (gene) | precentral gyrus |  | 0.00 | 0.03 | -0.07 | 0.06 |
| HTR3A (gene) | subcentral gyrus |  | -0.04 | 0.03 | -0.11 | 0.03 |
| HTR3A (gene) | inferior frontal gyrus (or F3) |  | 0.02 | 0.04 | -0.05 | 0.09 |
| HTR3A (gene) | triangular part of the inferior frontal gyrus |  | 0.00 | 0.03 | -0.07 | 0.06 |
| HTR3A (gene) | opercular part of the inferior frontal gyrus |  | -0.01 | 0.03 | -0.08 | 0.05 |
| HTR3A (gene) | orbital part |  | 0.02 | 0.03 | -0.04 | 0.08 |
| HTR3A (gene) | middle frontal gyrus (or F2) |  | -0.03 | 0.03 | -0.08 | 0.03 |
| HTR3A (gene) | superior frontal gyrus |  | 0.00 | 0.03 | -0.06 | 0.06 |
| HTR3A (gene) | gyrus rectus |  | 0.00 | 0.03 | -0.06 | 0.06 |
| HTR3A (gene) | transverse frontopolar gyrus or gyri |  | 0.00 | 0.03 | -0.05 | 0.06 |
| HTR3A (gene) | medial orbital sulcus |  | 0.00 | 0.03 | -0.06 | 0.06 |
| HTR3A (gene) | 4 orbital gyri |  | -0.01 | 0.03 | -0.07 | 0.05 |
| HTR3A (gene) | superior (circular sulcus of the insula) |  | 0.04 | 0.03 | -0.03 | 0.10 |
| HTR3A (gene) | anterior (circular sulcus of the insula) |  | -0.02 | 0.04 | -0.10 | 0.04 |
| HTR3A (gene) | inferior (circular sulcus of the insula) |  | -0.05 | 0.03 | -0.10 | 0.01 |
| HTR3A (gene) | vertical ramus of anterior segment of lateral sulcus |  | 0.02 | 0.04 | -0.05 | 0.09 |
| HTR3A (gene) | horizontal ramus of anterior segment of lateral sulcus |  | 0.01 | 0.04 | -0.07 | 0.08 |
| HTR3A (gene) | posterior segment of the lateral sulcus |  | -0.03 | 0.03 | -0.09 | 0.02 |
| HTR3A (gene) | the short insular gyri |  | 0.03 | 0.04 | -0.05 | 0.10 |
| HTR3A (gene) | long insular gyrus |  | 0.02 | 0.03 | -0.04 | 0.08 |
| HTR3A (gene) | transverse temporal gyrus (or Heschl's gyrus) |  | -0.04 | 0.04 | -0.11 | 0.03 |
| HTR3A (gene) | planum temporale or temporal plane of the superior temporal gyrus |  | -0.03 | 0.03 | -0.09 | 0.04 |
| HTR3A (gene) | planum polare of the superior temporal gyrus |  | -0.05 | 0.04 | -0.12 | 0.03 |
| HTR3A (gene) | lateral aspect of the superior temporal gyrus |  | -0.04 | 0.04 | -0.11 | 0.04 |
| HTR3A (gene) | middle temporal gyrus |  | 0.03 | 0.04 | -0.05 | 0.11 |
| HTR3A (gene) | superior occipital gyrus (O1) |  | -0.02 | 0.04 | -0.10 | 0.07 |
| HTR3A (gene) | middle occipital gyrus (O2, lateral occipital gyrus) |  | 0.01 | 0.03 | -0.06 | 0.08 |
| HTR3A (gene) | inferior temporal gyrus (T3) |  | 0.03 | 0.04 | -0.04 | 0.10 |
| HTR3A (gene) | lateral occipito-temporal gyrus (fusiform gyrus, O4-T4) |  | 0.02 | 0.04 | -0.06 | 0.10 |
| HTR3A (gene) | lingual gyrus (O5) |  | -0.02 | 0.04 | -0.09 | 0.06 |
| HTR3A (gene) | parahippocampal gyrus (or T5) |  | 0.01 | 0.03 | -0.06 | 0.08 |
| HTR3A (gene) | cuneus (O6) |  | 0.02 | 0.04 | -0.05 | 0.11 |
| HTR3A (gene) | occipital pole |  | 0.01 | 0.04 | -0.07 | 0.10 |
| HTR3A (gene) | temporal pole | * | 0.07 | 0.03 | 0.01 | 0.12 |
| HTR3A (gene) | postcentral gyrus |  | -0.03 | 0.03 | -0.09 | 0.04 |
| HTR3A (gene) | supramarginal gyrus |  | -0.01 | 0.03 | -0.08 | 0.06 |
| HTR3A (gene) | angular gyrus |  | -0.01 | 0.04 | -0.08 | 0.07 |
| HTR3A (gene) | superior parietal lobule (or P1) |  | -0.03 | 0.03 | -0.10 | 0.03 |
| HTR3A (gene) | precuneus |  | -0.04 | 0.03 | -0.10 | 0.03 |
| HTR3A (gene) | paracentral lobule and sulcus |  | 0.02 | 0.03 | -0.04 | 0.08 |
| HTR3A (gene) | subcentral gyrus and sulci |  | -0.04 | 0.03 | -0.11 | 0.03 |
| HTR3A (gene) | marginal branch (or part) of the cingulate sulcus |  | -0.06 | 0.04 | -0.13 | 0.02 |
| HTR3A (gene) | subparietal sulcus |  | -0.04 | 0.04 | -0.11 | 0.03 |
| HTR3A (gene) | calcarine sulcus |  | -0.02 | 0.03 | -0.07 | 0.03 |
| HTR3A (gene) | medial occipitotemporal sulcus (or collateral sulcus) |  | -0.05 | 0.03 | -0.11 | 0.01 |
| HTR3A (gene) | lateral occipito-temporal (or fusiform) sulcus |  | -0.07 | 0.04 | -0.15 | 0.02 |
| HTR3A (gene) | subcallosal area or gyrus |  | 0.01 | 0.04 | -0.07 | 0.09 |
| HTR3A (gene) | pericallosal sulcus or sulcus of the corpus callosum |  | 0.03 | 0.04 | -0.04 | 0.11 |
| HTR3A (gene) | anterior (ACC) |  | -0.01 | 0.03 | -0.08 | 0.05 |
| HTR3A (gene) | middle-anterior (aMCC) |  | -0.03 | 0.03 | -0.10 | 0.02 |
| HTR3A (gene) | middle-posterior (pMCC) |  | -0.04 | 0.03 | -0.11 | 0.02 |
| HTR3A (gene) | posterior-dorsal (dPCC) |  | -0.02 | 0.03 | -0.09 | 0.05 |
| HTR3A (gene) | posterior-ventral (vPCC or isthmus) |  | -0.02 | 0.03 | -0.09 | 0.04 |
| DRD2 (gene) | precentral gyrus |  | 0.06 | 0.03 | -0.01 | 0.12 |
| DRD2 (gene) | subcentral gyrus |  | 0.07 | 0.04 | -0.01 | 0.14 |
| DRD2 (gene) | inferior frontal gyrus (or F3) |  | 0.01 | 0.04 | -0.06 | 0.08 |
| DRD2 (gene) | triangular part of the inferior frontal gyrus | * | 0.11 | 0.03 | 0.04 | 0.17 |
| DRD2 (gene) | opercular part of the inferior frontal gyrus |  | 0.05 | 0.03 | -0.01 | 0.12 |
| DRD2 (gene) | orbital part |  | 0.06 | 0.04 | -0.01 | 0.13 |
| DRD2 (gene) | middle frontal gyrus (or F2) |  | 0.05 | 0.04 | -0.02 | 0.12 |
| DRD2 (gene) | superior frontal gyrus | * | 0.08 | 0.04 | 0.00 | 0.15 |
| DRD2 (gene) | gyrus rectus |  | 0.03 | 0.03 | -0.03 | 0.10 |
| DRD2 (gene) | transverse frontopolar gyrus or gyri |  | 0.05 | 0.04 | -0.02 | 0.12 |
| DRD2 (gene) | medial orbital sulcus |  | 0.05 | 0.04 | -0.02 | 0.13 |
| DRD2 (gene) | 4 orbital gyri | * | 0.09 | 0.03 | 0.02 | 0.15 |
| DRD2 (gene) | superior (circular sulcus of the insula) |  | 0.06 | 0.04 | 0.00 | 0.14 |
| DRD2 (gene) | anterior (circular sulcus of the insula) |  | 0.03 | 0.04 | -0.05 | 0.10 |
| DRD2 (gene) | inferior (circular sulcus of the insula) |  | 0.03 | 0.03 | -0.04 | 0.09 |
| DRD2 (gene) | vertical ramus of anterior segment of lateral sulcus |  | 0.01 | 0.04 | -0.06 | 0.08 |
| DRD2 (gene) | horizontal ramus of anterior segment of lateral sulcus |  | 0.04 | 0.04 | -0.04 | 0.11 |
| DRD2 (gene) | posterior segment of the lateral sulcus | * | 0.08 | 0.04 | 0.01 | 0.15 |
| DRD2 (gene) | the short insular gyri |  | 0.04 | 0.03 | -0.02 | 0.11 |
| DRD2 (gene) | long insular gyrus |  | 0.06 | 0.04 | -0.01 | 0.13 |
| DRD2 (gene) | transverse temporal gyrus (or Heschl's gyrus) |  | 0.07 | 0.04 | 0.00 | 0.15 |
| DRD2 (gene) | planum temporale or temporal plane of the superior temporal gyrus | * | 0.11 | 0.04 | 0.04 | 0.18 |
| DRD2 (gene) | planum polare of the superior temporal gyrus | * | 0.09 | 0.04 | 0.02 | 0.16 |
| DRD2 (gene) | lateral aspect of the superior temporal gyrus |  | 0.07 | 0.04 | 0.00 | 0.15 |
| DRD2 (gene) | middle temporal gyrus | * | 0.10 | 0.04 | 0.02 | 0.17 |
| DRD2 (gene) | superior occipital gyrus (O1) |  | 0.06 | 0.04 | -0.01 | 0.14 |
| DRD2 (gene) | middle occipital gyrus (O2, lateral occipital gyrus) | * | 0.08 | 0.04 | 0.01 | 0.16 |
| DRD2 (gene) | inferior temporal gyrus (T3) | * | 0.10 | 0.04 | 0.02 | 0.17 |
| DRD2 (gene) | lateral occipito-temporal gyrus (fusiform gyrus, O4-T4) | * | 0.14 | 0.04 | 0.07 | 0.22 |
| DRD2 (gene) | lingual gyrus (O5) | * | 0.09 | 0.04 | 0.02 | 0.17 |
| DRD2 (gene) | parahippocampal gyrus (or T5) | * | 0.09 | 0.04 | 0.01 | 0.17 |
| DRD2 (gene) | cuneus (O6) |  | 0.06 | 0.04 | -0.02 | 0.15 |
| DRD2 (gene) | occipital pole |  | 0.00 | 0.04 | -0.09 | 0.08 |
| DRD2 (gene) | temporal pole |  | 0.07 | 0.04 | 0.00 | 0.14 |
| DRD2 (gene) | postcentral gyrus | * | 0.10 | 0.04 | 0.03 | 0.17 |
| DRD2 (gene) | supramarginal gyrus | * | 0.13 | 0.04 | 0.06 | 0.20 |
| DRD2 (gene) | angular gyrus | * | 0.09 | 0.03 | 0.02 | 0.16 |
| DRD2 (gene) | superior parietal lobule (or P1) |  | 0.06 | 0.04 | -0.01 | 0.13 |
| DRD2 (gene) | precuneus |  | 0.07 | 0.04 | 0.00 | 0.15 |
| DRD2 (gene) | paracentral lobule and sulcus | * | 0.09 | 0.03 | 0.02 | 0.15 |
| DRD2 (gene) | subcentral gyrus and sulci |  | 0.07 | 0.04 | -0.01 | 0.14 |
| DRD2 (gene) | marginal branch (or part) of the cingulate sulcus | * | 0.08 | 0.04 | 0.01 | 0.15 |
| DRD2 (gene) | subparietal sulcus |  | 0.04 | 0.04 | -0.04 | 0.12 |
| DRD2 (gene) | calcarine sulcus |  | 0.01 | 0.04 | -0.05 | 0.09 |
| DRD2 (gene) | medial occipitotemporal sulcus (or collateral sulcus) | * | 0.08 | 0.04 | 0.01 | 0.15 |
| DRD2 (gene) | lateral occipito-temporal (or fusiform) sulcus | * | 0.08 | 0.04 | 0.01 | 0.16 |
| DRD2 (gene) | subcallosal area or gyrus |  | 0.00 | 0.04 | -0.08 | 0.07 |
| DRD2 (gene) | pericallosal sulcus or sulcus of the corpus callosum |  | 0.03 | 0.03 | -0.04 | 0.09 |
| DRD2 (gene) | anterior (ACC) |  | 0.07 | 0.04 | 0.00 | 0.14 |
| DRD2 (gene) | middle-anterior (aMCC) |  | 0.06 | 0.03 | -0.01 | 0.13 |
| DRD2 (gene) | middle-posterior (pMCC) | * | 0.07 | 0.03 | 0.01 | 0.14 |
| DRD2 (gene) | posterior-dorsal (dPCC) | * | 0.11 | 0.03 | 0.05 | 0.18 |
| DRD2 (gene) | posterior-ventral (vPCC or isthmus) |  | 0.03 | 0.04 | -0.05 | 0.10 |
| NR3C1 (gene) | precentral gyrus | * | 0.07 | 0.04 | 0.00 | 0.14 |
| NR3C1 (gene) | subcentral gyrus | * | 0.08 | 0.04 | 0.01 | 0.15 |
| NR3C1 (gene) | inferior frontal gyrus (or F3) |  | 0.04 | 0.03 | -0.02 | 0.11 |
| NR3C1 (gene) | triangular part of the inferior frontal gyrus |  | 0.01 | 0.03 | -0.06 | 0.07 |
| NR3C1 (gene) | opercular part of the inferior frontal gyrus |  | 0.05 | 0.03 | -0.01 | 0.11 |
| NR3C1 (gene) | orbital part |  | -0.02 | 0.03 | -0.08 | 0.05 |
| NR3C1 (gene) | middle frontal gyrus (or F2) |  | 0.04 | 0.03 | -0.02 | 0.10 |
| NR3C1 (gene) | superior frontal gyrus |  | 0.02 | 0.03 | -0.04 | 0.08 |
| NR3C1 (gene) | gyrus rectus |  | 0.01 | 0.04 | -0.06 | 0.08 |
| NR3C1 (gene) | transverse frontopolar gyrus or gyri |  | 0.02 | 0.04 | -0.05 | 0.09 |
| NR3C1 (gene) | medial orbital sulcus |  | 0.05 | 0.04 | -0.02 | 0.13 |
| NR3C1 (gene) | 4 orbital gyri |  | 0.03 | 0.03 | -0.04 | 0.09 |
| NR3C1 (gene) | superior (circular sulcus of the insula) |  | 0.02 | 0.04 | -0.06 | 0.09 |
| NR3C1 (gene) | anterior (circular sulcus of the insula) |  | 0.00 | 0.03 | -0.07 | 0.06 |
| NR3C1 (gene) | inferior (circular sulcus of the insula) |  | 0.01 | 0.04 | -0.06 | 0.08 |
| NR3C1 (gene) | vertical ramus of anterior segment of lateral sulcus |  | 0.04 | 0.03 | -0.02 | 0.11 |
| NR3C1 (gene) | horizontal ramus of anterior segment of lateral sulcus |  | -0.05 | 0.03 | -0.11 | 0.02 |
| NR3C1 (gene) | posterior segment of the lateral sulcus | * | 0.07 | 0.03 | 0.00 | 0.13 |
| NR3C1 (gene) | the short insular gyri |  | 0.00 | 0.04 | -0.07 | 0.08 |
| NR3C1 (gene) | long insular gyrus |  | -0.01 | 0.04 | -0.08 | 0.06 |
| NR3C1 (gene) | transverse temporal gyrus (or Heschl's gyrus) |  | 0.03 | 0.04 | -0.04 | 0.10 |
| NR3C1 (gene) | planum temporale or temporal plane of the superior temporal gyrus |  | 0.04 | 0.03 | -0.02 | 0.11 |
| NR3C1 (gene) | planum polare of the superior temporal gyrus | * | 0.09 | 0.03 | 0.02 | 0.15 |
| NR3C1 (gene) | lateral aspect of the superior temporal gyrus |  | 0.06 | 0.04 | -0.02 | 0.13 |
| NR3C1 (gene) | middle temporal gyrus |  | 0.05 | 0.04 | -0.02 | 0.12 |
| NR3C1 (gene) | superior occipital gyrus (O1) | * | 0.09 | 0.04 | 0.01 | 0.16 |
| NR3C1 (gene) | middle occipital gyrus (O2, lateral occipital gyrus) |  | 0.07 | 0.04 | -0.01 | 0.14 |
| NR3C1 (gene) | inferior temporal gyrus (T3) | * | 0.10 | 0.04 | 0.02 | 0.17 |
| NR3C1 (gene) | lateral occipito-temporal gyrus (fusiform gyrus, O4-T4) | * | 0.08 | 0.04 | 0.00 | 0.15 |
| NR3C1 (gene) | lingual gyrus (O5) |  | -0.06 | 0.03 | -0.13 | 0.00 |
| NR3C1 (gene) | parahippocampal gyrus (or T5) | * | 0.08 | 0.04 | 0.00 | 0.15 |
| NR3C1 (gene) | cuneus (O6) |  | -0.03 | 0.03 | -0.10 | 0.04 |
| NR3C1 (gene) | occipital pole |  | 0.00 | 0.04 | -0.07 | 0.08 |
| NR3C1 (gene) | temporal pole | * | 0.07 | 0.03 | 0.00 | 0.14 |
| NR3C1 (gene) | postcentral gyrus |  | 0.03 | 0.03 | -0.04 | 0.09 |
| NR3C1 (gene) | supramarginal gyrus |  | 0.05 | 0.03 | -0.01 | 0.12 |
| NR3C1 (gene) | angular gyrus |  | 0.01 | 0.04 | -0.06 | 0.08 |
| NR3C1 (gene) | superior parietal lobule (or P1) |  | 0.05 | 0.04 | -0.03 | 0.12 |
| NR3C1 (gene) | precuneus |  | 0.03 | 0.04 | -0.05 | 0.11 |
| NR3C1 (gene) | paracentral lobule and sulcus | * | 0.08 | 0.03 | 0.01 | 0.14 |
| NR3C1 (gene) | subcentral gyrus and sulci | * | 0.08 | 0.04 | 0.01 | 0.15 |
| NR3C1 (gene) | marginal branch (or part) of the cingulate sulcus |  | -0.01 | 0.04 | -0.08 | 0.07 |
| NR3C1 (gene) | subparietal sulcus |  | -0.01 | 0.04 | -0.09 | 0.07 |
| NR3C1 (gene) | calcarine sulcus |  | -0.01 | 0.03 | -0.07 | 0.05 |
| NR3C1 (gene) | medial occipitotemporal sulcus (or collateral sulcus) | * | 0.08 | 0.03 | 0.01 | 0.14 |
| NR3C1 (gene) | lateral occipito-temporal (or fusiform) sulcus |  | 0.03 | 0.04 | -0.04 | 0.11 |
| NR3C1 (gene) | subcallosal area or gyrus |  | 0.04 | 0.04 | -0.04 | 0.12 |
| NR3C1 (gene) | pericallosal sulcus or sulcus of the corpus callosum |  | 0.00 | 0.04 | -0.07 | 0.07 |
| NR3C1 (gene) | anterior (ACC) |  | 0.00 | 0.04 | -0.08 | 0.07 |
| NR3C1 (gene) | middle-anterior (aMCC) |  | -0.02 | 0.03 | -0.08 | 0.05 |
| NR3C1 (gene) | middle-posterior (pMCC) |  | 0.01 | 0.04 | -0.06 | 0.08 |
| NR3C1 (gene) | posterior-dorsal (dPCC) |  | 0.04 | 0.03 | -0.04 | 0.10 |
| NR3C1 (gene) | posterior-ventral (vPCC or isthmus) |  | 0.01 | 0.04 | -0.07 | 0.08 |
| OXTR (gene) | precentral gyrus |  | -0.04 | 0.04 | -0.11 | 0.03 |
| OXTR (gene) | subcentral gyrus |  | -0.02 | 0.03 | -0.09 | 0.04 |
| OXTR (gene) | inferior frontal gyrus (or F3) |  | -0.02 | 0.04 | -0.09 | 0.06 |
| OXTR (gene) | triangular part of the inferior frontal gyrus |  | 0.04 | 0.04 | -0.03 | 0.11 |
| OXTR (gene) | opercular part of the inferior frontal gyrus |  | 0.03 | 0.03 | -0.04 | 0.09 |
| OXTR (gene) | orbital part | * | 0.09 | 0.03 | 0.02 | 0.16 |
| OXTR (gene) | middle frontal gyrus (or F2) |  | -0.02 | 0.03 | -0.08 | 0.05 |
| OXTR (gene) | superior frontal gyrus |  | -0.02 | 0.03 | -0.09 | 0.04 |
| OXTR (gene) | gyrus rectus |  | -0.01 | 0.04 | -0.07 | 0.07 |
| OXTR (gene) | transverse frontopolar gyrus or gyri |  | 0.04 | 0.03 | -0.03 | 0.10 |
| OXTR (gene) | medial orbital sulcus |  | 0.03 | 0.04 | -0.05 | 0.11 |
| OXTR (gene) | 4 orbital gyri |  | 0.00 | 0.04 | -0.07 | 0.07 |
| OXTR (gene) | superior (circular sulcus of the insula) |  | 0.01 | 0.03 | -0.06 | 0.08 |
| OXTR (gene) | anterior (circular sulcus of the insula) |  | 0.03 | 0.04 | -0.04 | 0.10 |
| OXTR (gene) | inferior (circular sulcus of the insula) |  | -0.03 | 0.03 | -0.10 | 0.04 |
| OXTR (gene) | vertical ramus of anterior segment of lateral sulcus |  | -0.02 | 0.04 | -0.09 | 0.06 |
| OXTR (gene) | horizontal ramus of anterior segment of lateral sulcus |  | 0.05 | 0.04 | -0.03 | 0.12 |
| OXTR (gene) | posterior segment of the lateral sulcus |  | 0.00 | 0.04 | -0.08 | 0.08 |
| OXTR (gene) | the short insular gyri |  | -0.01 | 0.04 | -0.08 | 0.06 |
| OXTR (gene) | long insular gyrus |  | 0.00 | 0.04 | -0.08 | 0.07 |
| OXTR (gene) | transverse temporal gyrus (or Heschl's gyrus) |  | 0.00 | 0.04 | -0.08 | 0.07 |
| OXTR (gene) | planum temporale or temporal plane of the superior temporal gyrus |  | -0.01 | 0.04 | -0.08 | 0.06 |
| OXTR (gene) | planum polare of the superior temporal gyrus |  | 0.03 | 0.04 | -0.05 | 0.10 |
| OXTR (gene) | lateral aspect of the superior temporal gyrus |  | 0.02 | 0.04 | -0.06 | 0.10 |
| OXTR (gene) | middle temporal gyrus |  | -0.01 | 0.04 | -0.08 | 0.06 |
| OXTR (gene) | superior occipital gyrus (O1) |  | 0.05 | 0.04 | -0.03 | 0.12 |
| OXTR (gene) | middle occipital gyrus (O2, lateral occipital gyrus) |  | 0.00 | 0.04 | -0.08 | 0.07 |
| OXTR (gene) | inferior temporal gyrus (T3) |  | -0.02 | 0.04 | -0.10 | 0.06 |
| OXTR (gene) | lateral occipito-temporal gyrus (fusiform gyrus, O4-T4) |  | -0.04 | 0.04 | -0.12 | 0.03 |
| OXTR (gene) | lingual gyrus (O5) |  | -0.02 | 0.04 | -0.09 | 0.04 |
| OXTR (gene) | parahippocampal gyrus (or T5) |  | 0.07 | 0.04 | -0.02 | 0.14 |
| OXTR (gene) | cuneus (O6) |  | -0.06 | 0.04 | -0.14 | 0.01 |
| OXTR (gene) | occipital pole |  | -0.01 | 0.04 | -0.09 | 0.07 |
| OXTR (gene) | temporal pole |  | -0.01 | 0.04 | -0.08 | 0.07 |
| OXTR (gene) | postcentral gyrus |  | 0.00 | 0.04 | -0.07 | 0.07 |
| OXTR (gene) | supramarginal gyrus |  | 0.02 | 0.03 | -0.05 | 0.08 |
| OXTR (gene) | angular gyrus |  | 0.03 | 0.03 | -0.03 | 0.08 |
| OXTR (gene) | superior parietal lobule (or P1) |  | 0.01 | 0.03 | -0.06 | 0.07 |
| OXTR (gene) | precuneus |  | 0.04 | 0.04 | -0.03 | 0.11 |
| OXTR (gene) | paracentral lobule and sulcus |  | -0.02 | 0.04 | -0.09 | 0.05 |
| OXTR (gene) | subcentral gyrus and sulci |  | -0.02 | 0.03 | -0.09 | 0.04 |
| OXTR (gene) | marginal branch (or part) of the cingulate sulcus |  | -0.02 | 0.04 | -0.10 | 0.05 |
| OXTR (gene) | subparietal sulcus |  | 0.00 | 0.04 | -0.07 | 0.08 |
| OXTR (gene) | calcarine sulcus | * | -0.08 | 0.03 | -0.14 | -0.01 |
| OXTR (gene) | medial occipitotemporal sulcus (or collateral sulcus) |  | 0.05 | 0.03 | -0.03 | 0.11 |
| OXTR (gene) | lateral occipito-temporal (or fusiform) sulcus |  | 0.02 | 0.04 | -0.06 | 0.09 |
| OXTR (gene) | subcallosal area or gyrus |  | 0.05 | 0.04 | -0.03 | 0.13 |
| OXTR (gene) | pericallosal sulcus or sulcus of the corpus callosum |  | 0.02 | 0.04 | -0.06 | 0.10 |
| OXTR (gene) | anterior (ACC) |  | -0.04 | 0.04 | -0.11 | 0.03 |
| OXTR (gene) | middle-anterior (aMCC) |  | -0.02 | 0.04 | -0.08 | 0.07 |
| OXTR (gene) | middle-posterior (pMCC) |  | -0.01 | 0.04 | -0.08 | 0.06 |
| OXTR (gene) | posterior-dorsal (dPCC) |  | -0.03 | 0.04 | -0.10 | 0.04 |
| OXTR (gene) | posterior-ventral (vPCC or isthmus) |  | -0.06 | 0.04 | -0.13 | 0.02 |
| PTE | precentral gyrus |  | -0.03 | 0.04 | -0.11 | 0.04 |
| PTE | subcentral gyrus | * | -0.08 | 0.04 | -0.15 | -0.01 |
| PTE | inferior frontal gyrus (or F3) | * | -0.13 | 0.03 | -0.20 | -0.06 |
| PTE | triangular part of the inferior frontal gyrus | * | -0.08 | 0.03 | -0.14 | -0.01 |
| PTE | opercular part of the inferior frontal gyrus | * | -0.09 | 0.03 | -0.16 | -0.03 |
| PTE | orbital part |  | -0.03 | 0.03 | -0.09 | 0.03 |
| PTE | middle frontal gyrus (or F2) |  | -0.05 | 0.03 | -0.12 | 0.01 |
| PTE | superior frontal gyrus |  | -0.04 | 0.03 | -0.10 | 0.02 |
| PTE | gyrus rectus |  | -0.07 | 0.04 | -0.14 | 0.01 |
| PTE | transverse frontopolar gyrus or gyri |  | -0.07 | 0.03 | -0.13 | 0.00 |
| PTE | medial orbital sulcus |  | -0.05 | 0.03 | -0.12 | 0.02 |
| PTE | 4 orbital gyri | * | -0.08 | 0.03 | -0.14 | -0.02 |
| PTE | superior (circular sulcus of the insula) | * | -0.08 | 0.03 | -0.15 | -0.02 |
| PTE | anterior (circular sulcus of the insula) |  | -0.05 | 0.04 | -0.12 | 0.03 |
| PTE | inferior (circular sulcus of the insula) |  | -0.04 | 0.03 | -0.10 | 0.02 |
| PTE | vertical ramus of anterior segment of lateral sulcus | * | -0.13 | 0.03 | -0.20 | -0.06 |
| PTE | horizontal ramus of anterior segment of lateral sulcus |  | -0.01 | 0.04 | -0.08 | 0.07 |
| PTE | posterior segment of the lateral sulcus |  | -0.02 | 0.03 | -0.08 | 0.05 |
| PTE | the short insular gyri |  | 0.03 | 0.04 | -0.04 | 0.10 |
| PTE | long insular gyrus |  | 0.01 | 0.04 | -0.07 | 0.08 |
| PTE | transverse temporal gyrus (or Heschl's gyrus) |  | -0.06 | 0.03 | -0.12 | 0.01 |
| PTE | planum temporale or temporal plane of the superior temporal gyrus | * | -0.12 | 0.03 | -0.18 | -0.05 |
| PTE | planum polare of the superior temporal gyrus | * | -0.12 | 0.03 | -0.19 | -0.05 |
| PTE | lateral aspect of the superior temporal gyrus | * | -0.12 | 0.04 | -0.19 | -0.05 |
| PTE | middle temporal gyrus |  | -0.04 | 0.03 | -0.10 | 0.03 |
| PTE | superior occipital gyrus (O1) |  | -0.04 | 0.04 | -0.12 | 0.03 |
| PTE | middle occipital gyrus (O2, lateral occipital gyrus) |  | 0.01 | 0.04 | -0.07 | 0.08 |
| PTE | inferior temporal gyrus (T3) | * | -0.08 | 0.04 | -0.15 | -0.01 |
| PTE | lateral occipito-temporal gyrus (fusiform gyrus, O4-T4) |  | -0.04 | 0.04 | -0.11 | 0.03 |
| PTE | lingual gyrus (O5) | * | -0.07 | 0.03 | -0.13 | 0.00 |
| PTE | parahippocampal gyrus (or T5) |  | -0.02 | 0.04 | -0.09 | 0.05 |
| PTE | cuneus (O6) |  | -0.03 | 0.04 | -0.10 | 0.05 |
| PTE | occipital pole |  | -0.04 | 0.04 | -0.11 | 0.04 |
| PTE | temporal pole | * | -0.06 | 0.03 | -0.13 | 0.00 |
| PTE | postcentral gyrus | * | -0.07 | 0.04 | -0.14 | 0.00 |
| PTE | supramarginal gyrus | * | -0.09 | 0.03 | -0.16 | -0.02 |
| PTE | angular gyrus |  | -0.03 | 0.04 | -0.10 | 0.04 |
| PTE | superior parietal lobule (or P1) |  | -0.05 | 0.04 | -0.11 | 0.03 |
| PTE | precuneus |  | -0.03 | 0.03 | -0.10 | 0.04 |
| PTE | paracentral lobule and sulcus |  | -0.02 | 0.04 | -0.09 | 0.05 |
| PTE | subcentral gyrus and sulci | * | -0.08 | 0.04 | -0.15 | -0.01 |
| PTE | marginal branch (or part) of the cingulate sulcus | * | -0.08 | 0.03 | -0.14 | -0.01 |
| PTE | subparietal sulcus |  | -0.01 | 0.04 | -0.08 | 0.07 |
| PTE | calcarine sulcus |  | -0.06 | 0.03 | -0.11 | 0.00 |
| PTE | medial occipitotemporal sulcus (or collateral sulcus) |  | -0.07 | 0.03 | -0.13 | 0.00 |
| PTE | lateral occipito-temporal (or fusiform) sulcus | * | -0.09 | 0.04 | -0.16 | -0.01 |
| PTE | subcallosal area or gyrus | * | -0.09 | 0.04 | -0.16 | -0.01 |
| PTE | pericallosal sulcus or sulcus of the corpus callosum |  | 0.04 | 0.04 | -0.06 | 0.12 |
| PTE | anterior (ACC) |  | -0.01 | 0.04 | -0.08 | 0.06 |
| PTE | middle-anterior (aMCC) |  | -0.02 | 0.04 | -0.09 | 0.06 |
| PTE | middle-posterior (pMCC) |  | -0.02 | 0.04 | -0.09 | 0.05 |
| PTE | posterior-dorsal (dPCC) |  | -0.04 | 0.04 | -0.11 | 0.03 |
| PTE | posterior-ventral (vPCC or isthmus) |  | 0.04 | 0.04 | -0.04 | 0.11 |
| PTE | depression | * | 0.14 | 0.03 | 0.07 | 0.20 |
| precentral gyrus | depression |  | 0.00 | 0.03 | -0.06 | 0.06 |
| subcentral gyrus | depression |  | 0.00 | 0.03 | -0.05 | 0.05 |
| inferior frontal gyrus (or F3) | depression |  | 0.00 | 0.02 | -0.05 | 0.04 |
| triangular part of the inferior frontal gyrus | depression | * | -0.07 | 0.03 | -0.12 | 0.00 |
| opercular part of the inferior frontal gyrus | depression |  | -0.02 | 0.03 | -0.08 | 0.04 |
| orbital part | depression |  | 0.05 | 0.03 | -0.02 | 0.10 |
| middle frontal gyrus (or F2) | depression |  | -0.01 | 0.03 | -0.07 | 0.04 |
| superior frontal gyrus | depression |  | -0.02 | 0.03 | -0.07 | 0.03 |
| gyrus rectus | depression |  | -0.02 | 0.03 | -0.08 | 0.03 |
| transverse frontopolar gyrus or gyri | depression |  | -0.06 | 0.03 | -0.12 | 0.01 |
| medial orbital sulcus | depression |  | -0.05 | 0.03 | -0.12 | 0.02 |
| 4 orbital gyri | depression |  | 0.02 | 0.04 | -0.06 | 0.09 |
| superior (circular sulcus of the insula) | depression |  | -0.06 | 0.04 | -0.12 | 0.02 |
| anterior (circular sulcus of the insula) | depression | * | -0.11 | 0.03 | -0.17 | -0.05 |
| inferior (circular sulcus of the insula) | depression |  | 0.03 | 0.03 | -0.03 | 0.09 |
| vertical ramus of anterior segment of lateral sulcus | depression |  | 0.00 | 0.02 | -0.05 | 0.04 |
| horizontal ramus of anterior segment of lateral sulcus | depression |  | 0.04 | 0.04 | -0.03 | 0.11 |
| posterior segment of the lateral sulcus | depression |  | -0.01 | 0.03 | -0.07 | 0.04 |
| the short insular gyri | depression |  | 0.05 | 0.04 | -0.02 | 0.13 |
| long insular gyrus | depression |  | -0.06 | 0.03 | -0.12 | 0.01 |
| transverse temporal gyrus (or Heschl's gyrus) | depression |  | -0.01 | 0.03 | -0.07 | 0.05 |
| planum temporale or temporal plane of the superior temporal gyrus | depression |  | 0.03 | 0.03 | -0.03 | 0.09 |
| planum polare of the superior temporal gyrus | depression |  | -0.06 | 0.04 | -0.13 | 0.02 |
| lateral aspect of the superior temporal gyrus | depression |  | 0.00 | 0.03 | -0.05 | 0.06 |
| middle temporal gyrus | depression |  | 0.05 | 0.03 | -0.01 | 0.10 |
| superior occipital gyrus (O1) | depression |  | 0.03 | 0.04 | -0.04 | 0.10 |
| middle occipital gyrus (O2, lateral occipital gyrus) | depression |  | 0.00 | 0.03 | -0.07 | 0.07 |
| inferior temporal gyrus (T3) | depression |  | -0.01 | 0.03 | -0.08 | 0.06 |
| lateral occipito-temporal gyrus (fusiform gyrus, O4-T4) | depression |  | 0.02 | 0.03 | -0.05 | 0.08 |
| lingual gyrus (O5) | depression |  | -0.01 | 0.03 | -0.06 | 0.05 |
| parahippocampal gyrus (or T5) | depression |  | 0.06 | 0.03 | -0.01 | 0.12 |
| cuneus (O6) | depression |  | 0.04 | 0.03 | -0.02 | 0.11 |
| occipital pole | depression |  | 0.00 | 0.03 | -0.06 | 0.06 |
| temporal pole | depression |  | -0.04 | 0.03 | -0.11 | 0.02 |
| postcentral gyrus | depression |  | -0.04 | 0.03 | -0.11 | 0.03 |
| supramarginal gyrus | depression |  | 0.01 | 0.03 | -0.05 | 0.08 |
| angular gyrus | depression |  | -0.05 | 0.03 | -0.11 | 0.02 |
| superior parietal lobule (or P1) | depression |  | 0.04 | 0.03 | -0.02 | 0.09 |
| precuneus | depression |  | 0.05 | 0.03 | 0.00 | 0.11 |
| paracentral lobule and sulcus | depression |  | 0.00 | 0.03 | -0.05 | 0.06 |
| subcentral gyrus and sulci | depression |  | 0.00 | 0.03 | -0.05 | 0.05 |
| marginal branch (or part) of the cingulate sulcus | depression |  | 0.01 | 0.03 | -0.05 | 0.07 |
| subparietal sulcus | depression |  | 0.03 | 0.04 | -0.04 | 0.10 |
| calcarine sulcus | depression |  | 0.03 | 0.03 | -0.04 | 0.10 |
| medial occipitotemporal sulcus (or collateral sulcus) | depression |  | 0.00 | 0.04 | -0.06 | 0.08 |
| lateral occipito-temporal (or fusiform) sulcus | depression |  | 0.02 | 0.04 | -0.06 | 0.10 |
| subcallosal area or gyrus | depression |  | 0.03 | 0.04 | -0.04 | 0.10 |
| pericallosal sulcus or sulcus of the corpus callosum | depression |  | -0.05 | 0.03 | -0.11 | 0.02 |
| anterior (ACC) | depression |  | -0.02 | 0.03 | -0.09 | 0.04 |
| middle-anterior (aMCC) | depression |  | -0.05 | 0.03 | -0.10 | 0.01 |
| middle-posterior (pMCC) | depression | * | -0.08 | 0.03 | -0.14 | -0.01 |
| posterior-dorsal (dPCC) | depression |  | 0.00 | 0.04 | -0.07 | 0.07 |
| posterior-ventral (vPCC or isthmus) | depression |  | 0.07 | 0.03 | 0.00 | 0.13 |
| gender | precentral gyrus | * | 0.10 | 0.04 | 0.02 | 0.17 |
| gender | subcentral gyrus |  | 0.06 | 0.04 | -0.01 | 0.14 |
| gender | inferior frontal gyrus (or F3) |  | 0.03 | 0.03 | -0.04 | 0.10 |
| gender | triangular part of the inferior frontal gyrus | * | 0.10 | 0.04 | 0.02 | 0.17 |
| gender | opercular part of the inferior frontal gyrus | * | 0.09 | 0.03 | 0.02 | 0.15 |
| gender | orbital part | * | 0.10 | 0.04 | 0.03 | 0.17 |
| gender | middle frontal gyrus (or F2) | * | 0.10 | 0.04 | 0.03 | 0.17 |
| gender | superior frontal gyrus | * | 0.11 | 0.04 | 0.04 | 0.18 |
| gender | gyrus rectus |  | -0.01 | 0.04 | -0.09 | 0.06 |
| gender | transverse frontopolar gyrus or gyri | * | 0.10 | 0.04 | 0.01 | 0.18 |
| gender | medial orbital sulcus | * | -0.13 | 0.04 | -0.20 | -0.06 |
| gender | 4 orbital gyri |  | 0.04 | 0.04 | -0.04 | 0.11 |
| gender | superior (circular sulcus of the insula) |  | -0.08 | 0.04 | -0.15 | 0.00 |
| gender | anterior (circular sulcus of the insula) |  | 0.02 | 0.04 | -0.06 | 0.10 |
| gender | inferior (circular sulcus of the insula) | * | -0.09 | 0.03 | -0.15 | -0.02 |
| gender | vertical ramus of anterior segment of lateral sulcus |  | 0.03 | 0.03 | -0.04 | 0.10 |
| gender | horizontal ramus of anterior segment of lateral sulcus |  | 0.06 | 0.04 | -0.02 | 0.13 |
| gender | posterior segment of the lateral sulcus |  | -0.03 | 0.04 | -0.09 | 0.05 |
| gender | the short insular gyri |  | -0.02 | 0.04 | -0.10 | 0.06 |
| gender | long insular gyrus |  | 0.04 | 0.04 | -0.04 | 0.11 |
| gender | transverse temporal gyrus (or Heschl's gyrus) |  | 0.06 | 0.04 | -0.02 | 0.14 |
| gender | planum temporale or temporal plane of the superior temporal gyrus | * | 0.09 | 0.03 | 0.02 | 0.16 |
| gender | planum polare of the superior temporal gyrus |  | -0.04 | 0.04 | -0.12 | 0.03 |
| gender | lateral aspect of the superior temporal gyrus |  | 0.07 | 0.04 | -0.01 | 0.14 |
| gender | middle temporal gyrus |  | 0.00 | 0.04 | -0.07 | 0.08 |
| gender | superior occipital gyrus (O1) |  | 0.03 | 0.04 | -0.06 | 0.10 |
| gender | middle occipital gyrus (O2, lateral occipital gyrus) |  | 0.01 | 0.04 | -0.07 | 0.09 |
| gender | inferior temporal gyrus (T3) |  | 0.04 | 0.04 | -0.04 | 0.11 |
| gender | lateral occipito-temporal gyrus (fusiform gyrus, O4-T4) |  | -0.01 | 0.04 | -0.09 | 0.07 |
| gender | lingual gyrus (O5) |  | -0.04 | 0.04 | -0.11 | 0.03 |
| gender | parahippocampal gyrus (or T5) | * | 0.10 | 0.04 | 0.03 | 0.17 |
| gender | cuneus (O6) |  | -0.02 | 0.04 | -0.09 | 0.06 |
| gender | occipital pole | * | -0.08 | 0.04 | -0.16 | -0.01 |
| gender | temporal pole |  | 0.08 | 0.04 | 0.00 | 0.15 |
| gender | postcentral gyrus | * | 0.17 | 0.03 | 0.10 | 0.24 |
| gender | supramarginal gyrus | * | 0.09 | 0.04 | 0.02 | 0.16 |
| gender | angular gyrus | * | 0.17 | 0.03 | 0.10 | 0.23 |
| gender | superior parietal lobule (or P1) | * | 0.15 | 0.03 | 0.07 | 0.21 |
| gender | precuneus | * | 0.11 | 0.04 | 0.04 | 0.18 |
| gender | paracentral lobule and sulcus | * | 0.12 | 0.04 | 0.05 | 0.19 |
| gender | subcentral gyrus and sulci |  | 0.06 | 0.04 | -0.01 | 0.14 |
| gender | marginal branch (or part) of the cingulate sulcus |  | 0.06 | 0.04 | -0.02 | 0.13 |
| gender | subparietal sulcus |  | 0.03 | 0.04 | -0.04 | 0.12 |
| gender | calcarine sulcus | * | -0.08 | 0.03 | -0.14 | -0.01 |
| gender | medial occipitotemporal sulcus (or collateral sulcus) | * | 0.09 | 0.04 | 0.01 | 0.17 |
| gender | lateral occipito-temporal (or fusiform) sulcus |  | 0.06 | 0.04 | -0.01 | 0.13 |
| gender | subcallosal area or gyrus |  | 0.00 | 0.04 | -0.08 | 0.08 |
| gender | pericallosal sulcus or sulcus of the corpus callosum | * | 0.08 | 0.04 | 0.01 | 0.16 |
| gender | anterior (ACC) |  | 0.02 | 0.05 | -0.07 | 0.11 |
| gender | middle-anterior (aMCC) |  | 0.02 | 0.04 | -0.05 | 0.11 |
| gender | middle-posterior (pMCC) |  | 0.02 | 0.04 | -0.05 | 0.09 |
| gender | posterior-dorsal (dPCC) |  | -0.06 | 0.04 | -0.13 | 0.02 |
| gender | posterior-ventral (vPCC or isthmus) |  | -0.07 | 0.04 | -0.14 | 0.01 |
| gender | depression | * | -0.09 | 0.03 | -0.16 | -0.02 |
| age | precentral gyrus | * | -0.18 | 0.04 | -0.25 | -0.10 |
| age | subcentral gyrus | * | -0.21 | 0.03 | -0.27 | -0.14 |
| age | inferior frontal gyrus (or F3) | * | -0.18 | 0.04 | -0.25 | -0.10 |
| age | triangular part of the inferior frontal gyrus | * | -0.29 | 0.03 | -0.35 | -0.23 |
| age | opercular part of the inferior frontal gyrus | * | -0.30 | 0.03 | -0.35 | -0.23 |
| age | orbital part | * | -0.28 | 0.03 | -0.34 | -0.22 |
| age | middle frontal gyrus (or F2) | * | -0.29 | 0.03 | -0.35 | -0.23 |
| age | superior frontal gyrus | * | -0.32 | 0.03 | -0.37 | -0.26 |
| age | gyrus rectus | * | -0.26 | 0.03 | -0.32 | -0.20 |
| age | transverse frontopolar gyrus or gyri | * | -0.23 | 0.03 | -0.29 | -0.15 |
| age | medial orbital sulcus | * | -0.16 | 0.03 | -0.23 | -0.09 |
| age | 4 orbital gyri | * | -0.25 | 0.03 | -0.32 | -0.19 |
| age | superior (circular sulcus of the insula) | * | -0.22 | 0.04 | -0.29 | -0.14 |
| age | anterior (circular sulcus of the insula) | * | -0.19 | 0.04 | -0.26 | -0.11 |
| age | inferior (circular sulcus of the insula) | * | -0.32 | 0.03 | -0.38 | -0.26 |
| age | vertical ramus of anterior segment of lateral sulcus | * | -0.18 | 0.04 | -0.25 | -0.10 |
| age | horizontal ramus of anterior segment of lateral sulcus | * | -0.21 | 0.04 | -0.28 | -0.14 |
| age | posterior segment of the lateral sulcus | * | -0.29 | 0.03 | -0.35 | -0.23 |
| age | the short insular gyri | * | -0.19 | 0.04 | -0.26 | -0.11 |
| age | long insular gyrus | * | -0.23 | 0.03 | -0.29 | -0.17 |
| age | transverse temporal gyrus (or Heschl's gyrus) | * | -0.18 | 0.03 | -0.24 | -0.11 |
| age | planum temporale or temporal plane of the superior temporal gyrus | * | -0.22 | 0.03 | -0.27 | -0.16 |
| age | planum polare of the superior temporal gyrus | * | -0.15 | 0.03 | -0.22 | -0.08 |
| age | lateral aspect of the superior temporal gyrus | * | -0.15 | 0.04 | -0.22 | -0.08 |
| age | middle temporal gyrus | * | -0.18 | 0.03 | -0.25 | -0.12 |
| age | superior occipital gyrus (O1) |  | -0.07 | 0.04 | -0.15 | 0.01 |
| age | middle occipital gyrus (O2, lateral occipital gyrus) | * | -0.08 | 0.04 | -0.15 | 0.00 |
| age | inferior temporal gyrus (T3) |  | -0.02 | 0.04 | -0.10 | 0.07 |
| age | lateral occipito-temporal gyrus (fusiform gyrus, O4-T4) |  | 0.00 | 0.04 | -0.08 | 0.08 |
| age | lingual gyrus (O5) | * | -0.17 | 0.04 | -0.23 | -0.10 |
| age | parahippocampal gyrus (or T5) |  | 0.02 | 0.04 | -0.06 | 0.10 |
| age | cuneus (O6) | * | -0.15 | 0.04 | -0.22 | -0.08 |
| age | occipital pole | * | 0.09 | 0.04 | 0.01 | 0.16 |
| age | temporal pole |  | 0.02 | 0.05 | -0.08 | 0.11 |
| age | postcentral gyrus | * | -0.19 | 0.03 | -0.25 | -0.12 |
| age | supramarginal gyrus | * | -0.23 | 0.03 | -0.30 | -0.16 |
| age | angular gyrus | * | -0.25 | 0.03 | -0.31 | -0.19 |
| age | superior parietal lobule (or P1) | * | -0.23 | 0.03 | -0.29 | -0.16 |
| age | precuneus | * | -0.21 | 0.04 | -0.28 | -0.14 |
| age | paracentral lobule and sulcus | * | -0.20 | 0.04 | -0.27 | -0.12 |
| age | subcentral gyrus and sulci | * | -0.21 | 0.03 | -0.27 | -0.14 |
| age | marginal branch (or part) of the cingulate sulcus | * | -0.23 | 0.03 | -0.29 | -0.16 |
| age | subparietal sulcus | * | -0.15 | 0.04 | -0.23 | -0.07 |
| age | calcarine sulcus | * | -0.28 | 0.03 | -0.33 | -0.22 |
| age | medial occipitotemporal sulcus (or collateral sulcus) | * | -0.22 | 0.03 | -0.28 | -0.16 |
| age | lateral occipito-temporal (or fusiform) sulcus |  | -0.08 | 0.04 | -0.15 | 0.00 |
| age | subcallosal area or gyrus |  | -0.05 | 0.04 | -0.12 | 0.03 |
| age | pericallosal sulcus or sulcus of the corpus callosum |  | -0.06 | 0.04 | -0.13 | 0.02 |
| age | anterior (ACC) | * | -0.23 | 0.04 | -0.30 | -0.16 |
| age | middle-anterior (aMCC) | * | -0.27 | 0.03 | -0.33 | -0.20 |
| age | middle-posterior (pMCC) | * | -0.27 | 0.03 | -0.33 | -0.21 |
| age | posterior-dorsal (dPCC) | * | -0.18 | 0.04 | -0.25 | -0.11 |
| age | posterior-ventral (vPCC or isthmus) | * | -0.23 | 0.03 | -0.29 | -0.16 |
| age | depression |  | -0.07 | 0.03 | -0.13 | 0.00 |
| Auditpre | precentral gyrus |  | 0.05 | 0.03 | -0.02 | 0.12 |
| Auditpre | subcentral gyrus |  | -0.02 | 0.03 | -0.08 | 0.05 |
| Auditpre | inferior frontal gyrus (or F3) |  | 0.03 | 0.04 | -0.04 | 0.11 |
| Auditpre | triangular part of the inferior frontal gyrus |  | -0.05 | 0.03 | -0.12 | 0.02 |
| Auditpre | opercular part of the inferior frontal gyrus |  | -0.02 | 0.04 | -0.09 | 0.06 |
| Auditpre | orbital part |  | -0.02 | 0.04 | -0.09 | 0.05 |
| Auditpre | middle frontal gyrus (or F2) |  | -0.01 | 0.03 | -0.08 | 0.05 |
| Auditpre | superior frontal gyrus |  | 0.02 | 0.04 | -0.05 | 0.09 |
| Auditpre | gyrus rectus |  | 0.01 | 0.04 | -0.06 | 0.08 |
| Auditpre | transverse frontopolar gyrus or gyri |  | 0.02 | 0.04 | -0.05 | 0.09 |
| Auditpre | medial orbital sulcus |  | 0.00 | 0.04 | -0.07 | 0.07 |
| Auditpre | 4 orbital gyri |  | 0.03 | 0.04 | -0.04 | 0.10 |
| Auditpre | superior (circular sulcus of the insula) |  | -0.03 | 0.04 | -0.10 | 0.05 |
| Auditpre | anterior (circular sulcus of the insula) |  | -0.03 | 0.04 | -0.10 | 0.05 |
| Auditpre | inferior (circular sulcus of the insula) |  | 0.02 | 0.03 | -0.04 | 0.08 |
| Auditpre | vertical ramus of anterior segment of lateral sulcus |  | 0.03 | 0.04 | -0.04 | 0.11 |
| Auditpre | horizontal ramus of anterior segment of lateral sulcus |  | -0.03 | 0.04 | -0.11 | 0.05 |
| Auditpre | posterior segment of the lateral sulcus |  | -0.03 | 0.04 | -0.10 | 0.04 |
| Auditpre | the short insular gyri |  | 0.01 | 0.04 | -0.06 | 0.08 |
| Auditpre | long insular gyrus |  | 0.02 | 0.04 | -0.06 | 0.09 |
| Auditpre | transverse temporal gyrus (or Heschl's gyrus) |  | 0.05 | 0.04 | -0.04 | 0.13 |
| Auditpre | planum temporale or temporal plane of the superior temporal gyrus |  | 0.00 | 0.04 | -0.07 | 0.07 |
| Auditpre | planum polare of the superior temporal gyrus |  | -0.04 | 0.04 | -0.11 | 0.04 |
| Auditpre | lateral aspect of the superior temporal gyrus |  | -0.04 | 0.04 | -0.12 | 0.04 |
| Auditpre | middle temporal gyrus |  | -0.01 | 0.04 | -0.08 | 0.08 |
| Auditpre | superior occipital gyrus (O1) |  | 0.05 | 0.04 | -0.04 | 0.13 |
| Auditpre | middle occipital gyrus (O2, lateral occipital gyrus) |  | 0.05 | 0.04 | -0.03 | 0.13 |
| Auditpre | inferior temporal gyrus (T3) |  | -0.03 | 0.04 | -0.10 | 0.06 |
| Auditpre | lateral occipito-temporal gyrus (fusiform gyrus, O4-T4) |  | -0.01 | 0.04 | -0.09 | 0.08 |
| Auditpre | lingual gyrus (O5) |  | 0.03 | 0.04 | -0.05 | 0.10 |
| Auditpre | parahippocampal gyrus (or T5) |  | -0.05 | 0.04 | -0.13 | 0.04 |
| Auditpre | cuneus (O6) |  | 0.06 | 0.04 | -0.02 | 0.13 |
| Auditpre | occipital pole |  | 0.07 | 0.04 | -0.01 | 0.15 |
| Auditpre | temporal pole |  | -0.03 | 0.05 | -0.12 | 0.06 |
| Auditpre | postcentral gyrus |  | 0.04 | 0.04 | -0.03 | 0.11 |
| Auditpre | supramarginal gyrus |  | -0.03 | 0.04 | -0.10 | 0.05 |
| Auditpre | angular gyrus |  | 0.00 | 0.04 | -0.07 | 0.07 |
| Auditpre | superior parietal lobule (or P1) |  | 0.00 | 0.04 | -0.07 | 0.08 |
| Auditpre | precuneus |  | 0.03 | 0.04 | -0.04 | 0.10 |
| Auditpre | paracentral lobule and sulcus |  | 0.07 | 0.04 | -0.01 | 0.14 |
| Auditpre | subcentral gyrus and sulci |  | -0.02 | 0.03 | -0.08 | 0.05 |
| Auditpre | marginal branch (or part) of the cingulate sulcus |  | 0.03 | 0.04 | -0.05 | 0.11 |
| Auditpre | subparietal sulcus |  | 0.04 | 0.04 | -0.04 | 0.11 |
| Auditpre | calcarine sulcus |  | 0.05 | 0.04 | -0.02 | 0.13 |
| Auditpre | medial occipitotemporal sulcus (or collateral sulcus) |  | 0.00 | 0.04 | -0.09 | 0.08 |
| Auditpre | lateral occipito-temporal (or fusiform) sulcus |  | 0.04 | 0.03 | -0.02 | 0.10 |
| Auditpre | subcallosal area or gyrus | * | -0.11 | 0.04 | -0.18 | -0.03 |
| Auditpre | pericallosal sulcus or sulcus of the corpus callosum |  | 0.00 | 0.04 | -0.09 | 0.08 |
| Auditpre | anterior (ACC) |  | -0.02 | 0.04 | -0.10 | 0.07 |
| Auditpre | middle-anterior (aMCC) |  | 0.02 | 0.03 | -0.05 | 0.08 |
| Auditpre | middle-posterior (pMCC) |  | -0.01 | 0.04 | -0.08 | 0.06 |
| Auditpre | posterior-dorsal (dPCC) |  | 0.04 | 0.04 | -0.03 | 0.12 |
| Auditpre | posterior-ventral (vPCC or isthmus) |  | 0.06 | 0.04 | -0.02 | 0.14 |
| Auditpre | depression |  | 0.05 | 0.04 | -0.03 | 0.12 |
| PTE x SLC6A4 (gene) | precentral gyrus |  | -0.03 | 0.03 | -0.10 | 0.03 |
| PTE x SLC6A4 (gene) | subcentral gyrus |  | -0.02 | 0.03 | -0.09 | 0.04 |
| PTE x SLC6A4 (gene) | inferior frontal gyrus (or F3) |  | 0.00 | 0.03 | -0.07 | 0.06 |
| PTE x SLC6A4 (gene) | triangular part of the inferior frontal gyrus |  | 0.06 | 0.03 | 0.00 | 0.12 |
| PTE x SLC6A4 (gene) | opercular part of the inferior frontal gyrus |  | 0.02 | 0.03 | -0.04 | 0.07 |
| PTE x SLC6A4 (gene) | orbital part |  | -0.01 | 0.03 | -0.07 | 0.05 |
| PTE x SLC6A4 (gene) | middle frontal gyrus (or F2) |  | 0.05 | 0.03 | -0.02 | 0.11 |
| PTE x SLC6A4 (gene) | superior frontal gyrus |  | 0.04 | 0.03 | -0.03 | 0.10 |
| PTE x SLC6A4 (gene) | gyrus rectus | * | 0.07 | 0.03 | 0.01 | 0.13 |
| PTE x SLC6A4 (gene) | transverse frontopolar gyrus or gyri | * | 0.13 | 0.03 | 0.07 | 0.18 |
| PTE x SLC6A4 (gene) | medial orbital sulcus | * | 0.10 | 0.03 | 0.04 | 0.16 |
| PTE x SLC6A4 (gene) | 4 orbital gyri |  | 0.05 | 0.03 | 0.00 | 0.10 |
| PTE x SLC6A4 (gene) | superior (circular sulcus of the insula) |  | 0.02 | 0.03 | -0.04 | 0.08 |
| PTE x SLC6A4 (gene) | anterior (circular sulcus of the insula) |  | 0.03 | 0.04 | -0.05 | 0.09 |
| PTE x SLC6A4 (gene) | inferior (circular sulcus of the insula) |  | 0.00 | 0.03 | -0.05 | 0.05 |
| PTE x SLC6A4 (gene) | vertical ramus of anterior segment of lateral sulcus |  | 0.00 | 0.03 | -0.07 | 0.06 |
| PTE x SLC6A4 (gene) | horizontal ramus of anterior segment of lateral sulcus |  | 0.00 | 0.04 | -0.06 | 0.07 |
| PTE x SLC6A4 (gene) | posterior segment of the lateral sulcus |  | 0.00 | 0.03 | -0.06 | 0.05 |
| PTE x SLC6A4 (gene) | the short insular gyri |  | 0.05 | 0.03 | -0.01 | 0.11 |
| PTE x SLC6A4 (gene) | long insular gyrus |  | 0.03 | 0.03 | -0.04 | 0.09 |
| PTE x SLC6A4 (gene) | transverse temporal gyrus (or Heschl's gyrus) |  | 0.00 | 0.04 | -0.07 | 0.07 |
| PTE x SLC6A4 (gene) | planum temporale or temporal plane of the superior temporal gyrus |  | -0.02 | 0.03 | -0.08 | 0.04 |
| PTE x SLC6A4 (gene) | planum polare of the superior temporal gyrus |  | 0.02 | 0.03 | -0.05 | 0.08 |
| PTE x SLC6A4 (gene) | lateral aspect of the superior temporal gyrus |  | 0.04 | 0.03 | -0.02 | 0.11 |
| PTE x SLC6A4 (gene) | middle temporal gyrus | * | 0.07 | 0.03 | 0.01 | 0.13 |
| PTE x SLC6A4 (gene) | superior occipital gyrus (O1) |  | -0.02 | 0.04 | -0.09 | 0.06 |
| PTE x SLC6A4 (gene) | middle occipital gyrus (O2, lateral occipital gyrus) |  | 0.06 | 0.04 | -0.02 | 0.13 |
| PTE x SLC6A4 (gene) | inferior temporal gyrus (T3) |  | -0.03 | 0.03 | -0.10 | 0.04 |
| PTE x SLC6A4 (gene) | lateral occipito-temporal gyrus (fusiform gyrus, O4-T4) | * | 0.08 | 0.03 | 0.01 | 0.14 |
| PTE x SLC6A4 (gene) | lingual gyrus (O5) |  | 0.05 | 0.03 | -0.02 | 0.11 |
| PTE x SLC6A4 (gene) | parahippocampal gyrus (or T5) |  | 0.01 | 0.03 | -0.05 | 0.07 |
| PTE x SLC6A4 (gene) | cuneus (O6) |  | -0.01 | 0.03 | -0.07 | 0.06 |
| PTE x SLC6A4 (gene) | occipital pole |  | 0.07 | 0.04 | -0.01 | 0.13 |
| PTE x SLC6A4 (gene) | temporal pole |  | 0.03 | 0.03 | -0.03 | 0.09 |
| PTE x SLC6A4 (gene) | postcentral gyrus |  | -0.01 | 0.03 | -0.07 | 0.06 |
| PTE x SLC6A4 (gene) | supramarginal gyrus |  | -0.02 | 0.03 | -0.08 | 0.05 |
| PTE x SLC6A4 (gene) | angular gyrus |  | 0.00 | 0.03 | -0.07 | 0.07 |
| PTE x SLC6A4 (gene) | superior parietal lobule (or P1) |  | -0.01 | 0.04 | -0.08 | 0.06 |
| PTE x SLC6A4 (gene) | precuneus |  | 0.02 | 0.03 | -0.05 | 0.08 |
| PTE x SLC6A4 (gene) | paracentral lobule and sulcus |  | -0.05 | 0.03 | -0.11 | 0.02 |
| PTE x SLC6A4 (gene) | subcentral gyrus and sulci |  | -0.02 | 0.03 | -0.09 | 0.04 |
| PTE x SLC6A4 (gene) | marginal branch (or part) of the cingulate sulcus |  | -0.04 | 0.03 | -0.11 | 0.03 |
| PTE x SLC6A4 (gene) | subparietal sulcus | * | 0.09 | 0.03 | 0.02 | 0.15 |
| PTE x SLC6A4 (gene) | calcarine sulcus |  | 0.04 | 0.03 | -0.02 | 0.10 |
| PTE x SLC6A4 (gene) | medial occipitotemporal sulcus (or collateral sulcus) |  | 0.03 | 0.04 | -0.04 | 0.10 |
| PTE x SLC6A4 (gene) | lateral occipito-temporal (or fusiform) sulcus |  | 0.03 | 0.03 | -0.03 | 0.10 |
| PTE x SLC6A4 (gene) | subcallosal area or gyrus |  | 0.00 | 0.04 | -0.08 | 0.07 |
| PTE x SLC6A4 (gene) | pericallosal sulcus or sulcus of the corpus callosum |  | 0.06 | 0.04 | -0.02 | 0.14 |
| PTE x SLC6A4 (gene) | anterior (ACC) |  | 0.06 | 0.03 | 0.00 | 0.12 |
| PTE x SLC6A4 (gene) | middle-anterior (aMCC) |  | 0.03 | 0.03 | -0.04 | 0.09 |
| PTE x SLC6A4 (gene) | middle-posterior (pMCC) |  | 0.04 | 0.03 | -0.02 | 0.11 |
| PTE x SLC6A4 (gene) | posterior-dorsal (dPCC) | * | 0.09 | 0.04 | 0.02 | 0.16 |
| PTE x SLC6A4 (gene) | posterior-ventral (vPCC or isthmus) |  | 0.06 | 0.04 | -0.02 | 0.15 |
| PTE x FKBP5 (gene) | precentral gyrus |  | -0.02 | 0.03 | -0.08 | 0.06 |
| PTE x FKBP5 (gene) | subcentral gyrus |  | 0.07 | 0.04 | 0.00 | 0.14 |
| PTE x FKBP5 (gene) | inferior frontal gyrus (or F3) |  | -0.03 | 0.03 | -0.09 | 0.03 |
| PTE x FKBP5 (gene) | triangular part of the inferior frontal gyrus |  | -0.02 | 0.03 | -0.07 | 0.04 |
| PTE x FKBP5 (gene) | opercular part of the inferior frontal gyrus |  | 0.01 | 0.03 | -0.05 | 0.08 |
| PTE x FKBP5 (gene) | orbital part |  | 0.02 | 0.03 | -0.05 | 0.09 |
| PTE x FKBP5 (gene) | middle frontal gyrus (or F2) |  | -0.01 | 0.04 | -0.07 | 0.07 |
| PTE x FKBP5 (gene) | superior frontal gyrus |  | -0.02 | 0.03 | -0.08 | 0.04 |
| PTE x FKBP5 (gene) | gyrus rectus |  | 0.00 | 0.04 | -0.07 | 0.08 |
| PTE x FKBP5 (gene) | transverse frontopolar gyrus or gyri |  | 0.00 | 0.03 | -0.06 | 0.06 |
| PTE x FKBP5 (gene) | medial orbital sulcus | * | -0.08 | 0.03 | -0.14 | 0.00 |
| PTE x FKBP5 (gene) | 4 orbital gyri |  | -0.01 | 0.03 | -0.07 | 0.06 |
| PTE x FKBP5 (gene) | superior (circular sulcus of the insula) |  | 0.00 | 0.03 | -0.07 | 0.07 |
| PTE x FKBP5 (gene) | anterior (circular sulcus of the insula) |  | 0.02 | 0.04 | -0.05 | 0.09 |
| PTE x FKBP5 (gene) | inferior (circular sulcus of the insula) |  | 0.03 | 0.03 | -0.03 | 0.09 |
| PTE x FKBP5 (gene) | vertical ramus of anterior segment of lateral sulcus |  | -0.03 | 0.03 | -0.09 | 0.03 |
| PTE x FKBP5 (gene) | horizontal ramus of anterior segment of lateral sulcus |  | 0.02 | 0.03 | -0.05 | 0.08 |
| PTE x FKBP5 (gene) | posterior segment of the lateral sulcus |  | 0.02 | 0.04 | -0.05 | 0.09 |
| PTE x FKBP5 (gene) | the short insular gyri |  | 0.02 | 0.04 | -0.05 | 0.10 |
| PTE x FKBP5 (gene) | long insular gyrus |  | 0.05 | 0.03 | -0.02 | 0.11 |
| PTE x FKBP5 (gene) | transverse temporal gyrus (or Heschl's gyrus) |  | 0.05 | 0.03 | -0.01 | 0.12 |
| PTE x FKBP5 (gene) | planum temporale or temporal plane of the superior temporal gyrus |  | 0.01 | 0.03 | -0.05 | 0.07 |
| PTE x FKBP5 (gene) | planum polare of the superior temporal gyrus |  | 0.06 | 0.04 | -0.01 | 0.13 |
| PTE x FKBP5 (gene) | lateral aspect of the superior temporal gyrus |  | 0.06 | 0.04 | 0.00 | 0.14 |
| PTE x FKBP5 (gene) | middle temporal gyrus |  | 0.01 | 0.03 | -0.05 | 0.09 |
| PTE x FKBP5 (gene) | superior occipital gyrus (O1) |  | 0.01 | 0.03 | -0.05 | 0.08 |
| PTE x FKBP5 (gene) | middle occipital gyrus (O2, lateral occipital gyrus) |  | -0.02 | 0.04 | -0.09 | 0.06 |
| PTE x FKBP5 (gene) | inferior temporal gyrus (T3) |  | 0.04 | 0.03 | -0.02 | 0.11 |
| PTE x FKBP5 (gene) | lateral occipito-temporal gyrus (fusiform gyrus, O4-T4) |  | 0.03 | 0.03 | -0.03 | 0.10 |
| PTE x FKBP5 (gene) | lingual gyrus (O5) |  | -0.01 | 0.04 | -0.08 | 0.06 |
| PTE x FKBP5 (gene) | parahippocampal gyrus (or T5) |  | 0.03 | 0.03 | -0.04 | 0.10 |
| PTE x FKBP5 (gene) | cuneus (O6) |  | -0.03 | 0.04 | -0.10 | 0.05 |
| PTE x FKBP5 (gene) | occipital pole |  | 0.03 | 0.04 | -0.04 | 0.10 |
| PTE x FKBP5 (gene) | temporal pole |  | 0.01 | 0.03 | -0.05 | 0.08 |
| PTE x FKBP5 (gene) | postcentral gyrus |  | 0.05 | 0.03 | -0.01 | 0.12 |
| PTE x FKBP5 (gene) | supramarginal gyrus |  | 0.02 | 0.04 | -0.05 | 0.09 |
| PTE x FKBP5 (gene) | angular gyrus |  | 0.00 | 0.04 | -0.07 | 0.07 |
| PTE x FKBP5 (gene) | superior parietal lobule (or P1) |  | -0.02 | 0.03 | -0.08 | 0.05 |
| PTE x FKBP5 (gene) | precuneus |  | -0.04 | 0.03 | -0.09 | 0.03 |
| PTE x FKBP5 (gene) | paracentral lobule and sulcus |  | -0.02 | 0.03 | -0.08 | 0.05 |
| PTE x FKBP5 (gene) | subcentral gyrus and sulci |  | 0.07 | 0.04 | 0.00 | 0.14 |
| PTE x FKBP5 (gene) | marginal branch (or part) of the cingulate sulcus |  | -0.02 | 0.03 | -0.09 | 0.05 |
| PTE x FKBP5 (gene) | subparietal sulcus |  | -0.03 | 0.03 | -0.08 | 0.04 |
| PTE x FKBP5 (gene) | calcarine sulcus |  | 0.02 | 0.03 | -0.04 | 0.08 |
| PTE x FKBP5 (gene) | medial occipitotemporal sulcus (or collateral sulcus) |  | 0.01 | 0.03 | -0.05 | 0.07 |
| PTE x FKBP5 (gene) | lateral occipito-temporal (or fusiform) sulcus |  | 0.02 | 0.03 | -0.05 | 0.08 |
| PTE x FKBP5 (gene) | subcallosal area or gyrus |  | -0.07 | 0.04 | -0.14 | 0.01 |
| PTE x FKBP5 (gene) | pericallosal sulcus or sulcus of the corpus callosum |  | 0.02 | 0.04 | -0.06 | 0.10 |
| PTE x FKBP5 (gene) | anterior (ACC) |  | 0.00 | 0.04 | -0.07 | 0.07 |
| PTE x FKBP5 (gene) | middle-anterior (aMCC) |  | 0.02 | 0.04 | -0.05 | 0.09 |
| PTE x FKBP5 (gene) | middle-posterior (pMCC) |  | 0.03 | 0.03 | -0.03 | 0.11 |
| PTE x FKBP5 (gene) | posterior-dorsal (dPCC) |  | -0.03 | 0.03 | -0.09 | 0.04 |
| PTE x FKBP5 (gene) | posterior-ventral (vPCC or isthmus) |  | 0.03 | 0.03 | -0.04 | 0.09 |
| PTE x ADCYAP1R1 (gene) | precentral gyrus |  | 0.00 | 0.04 | -0.08 | 0.07 |
| PTE x ADCYAP1R1 (gene) | subcentral gyrus |  | 0.02 | 0.03 | -0.05 | 0.08 |
| PTE x ADCYAP1R1 (gene) | inferior frontal gyrus (or F3) |  | 0.00 | 0.03 | -0.07 | 0.07 |
| PTE x ADCYAP1R1 (gene) | triangular part of the inferior frontal gyrus |  | -0.04 | 0.04 | -0.11 | 0.03 |
| PTE x ADCYAP1R1 (gene) | opercular part of the inferior frontal gyrus |  | 0.00 | 0.04 | -0.07 | 0.07 |
| PTE x ADCYAP1R1 (gene) | orbital part |  | 0.03 | 0.03 | -0.04 | 0.10 |
| PTE x ADCYAP1R1 (gene) | middle frontal gyrus (or F2) |  | -0.01 | 0.03 | -0.07 | 0.06 |
| PTE x ADCYAP1R1 (gene) | superior frontal gyrus |  | 0.05 | 0.03 | -0.01 | 0.11 |
| PTE x ADCYAP1R1 (gene) | gyrus rectus |  | -0.02 | 0.04 | -0.09 | 0.06 |
| PTE x ADCYAP1R1 (gene) | transverse frontopolar gyrus or gyri |  | 0.05 | 0.04 | -0.03 | 0.13 |
| PTE x ADCYAP1R1 (gene) | medial orbital sulcus |  | 0.01 | 0.04 | -0.06 | 0.08 |
| PTE x ADCYAP1R1 (gene) | 4 orbital gyri |  | -0.02 | 0.03 | -0.07 | 0.04 |
| PTE x ADCYAP1R1 (gene) | superior (circular sulcus of the insula) |  | 0.01 | 0.04 | -0.06 | 0.08 |
| PTE x ADCYAP1R1 (gene) | anterior (circular sulcus of the insula) |  | -0.02 | 0.04 | -0.09 | 0.05 |
| PTE x ADCYAP1R1 (gene) | inferior (circular sulcus of the insula) |  | 0.02 | 0.04 | -0.05 | 0.10 |
| PTE x ADCYAP1R1 (gene) | vertical ramus of anterior segment of lateral sulcus |  | 0.00 | 0.03 | -0.07 | 0.07 |
| PTE x ADCYAP1R1 (gene) | horizontal ramus of anterior segment of lateral sulcus |  | -0.03 | 0.03 | -0.10 | 0.04 |
| PTE x ADCYAP1R1 (gene) | posterior segment of the lateral sulcus |  | 0.02 | 0.04 | -0.05 | 0.09 |
| PTE x ADCYAP1R1 (gene) | the short insular gyri |  | -0.03 | 0.04 | -0.11 | 0.04 |
| PTE x ADCYAP1R1 (gene) | long insular gyrus |  | -0.01 | 0.04 | -0.08 | 0.06 |
| PTE x ADCYAP1R1 (gene) | transverse temporal gyrus (or Heschl's gyrus) |  | -0.01 | 0.03 | -0.07 | 0.06 |
| PTE x ADCYAP1R1 (gene) | planum temporale or temporal plane of the superior temporal gyrus |  | 0.04 | 0.03 | -0.02 | 0.10 |
| PTE x ADCYAP1R1 (gene) | planum polare of the superior temporal gyrus |  | 0.03 | 0.03 | -0.05 | 0.09 |
| PTE x ADCYAP1R1 (gene) | lateral aspect of the superior temporal gyrus |  | 0.03 | 0.03 | -0.04 | 0.09 |
| PTE x ADCYAP1R1 (gene) | middle temporal gyrus |  | -0.04 | 0.04 | -0.11 | 0.03 |
| PTE x ADCYAP1R1 (gene) | superior occipital gyrus (O1) |  | 0.04 | 0.04 | -0.04 | 0.13 |
| PTE x ADCYAP1R1 (gene) | middle occipital gyrus (O2, lateral occipital gyrus) |  | 0.03 | 0.03 | -0.04 | 0.10 |
| PTE x ADCYAP1R1 (gene) | inferior temporal gyrus (T3) |  | 0.00 | 0.04 | -0.07 | 0.07 |
| PTE x ADCYAP1R1 (gene) | lateral occipito-temporal gyrus (fusiform gyrus, O4-T4) |  | -0.02 | 0.03 | -0.08 | 0.05 |
| PTE x ADCYAP1R1 (gene) | lingual gyrus (O5) |  | -0.06 | 0.04 | -0.13 | 0.02 |
| PTE x ADCYAP1R1 (gene) | parahippocampal gyrus (or T5) |  | -0.03 | 0.04 | -0.11 | 0.05 |
| PTE x ADCYAP1R1 (gene) | cuneus (O6) |  | -0.04 | 0.04 | -0.11 | 0.04 |
| PTE x ADCYAP1R1 (gene) | occipital pole |  | 0.03 | 0.04 | -0.06 | 0.11 |
| PTE x ADCYAP1R1 (gene) | temporal pole |  | -0.03 | 0.03 | -0.10 | 0.03 |
| PTE x ADCYAP1R1 (gene) | postcentral gyrus |  | 0.02 | 0.03 | -0.04 | 0.08 |
| PTE x ADCYAP1R1 (gene) | supramarginal gyrus |  | -0.04 | 0.03 | -0.10 | 0.03 |
| PTE x ADCYAP1R1 (gene) | angular gyrus |  | -0.06 | 0.04 | -0.13 | 0.01 |
| PTE x ADCYAP1R1 (gene) | superior parietal lobule (or P1) |  | -0.01 | 0.03 | -0.07 | 0.06 |
| PTE x ADCYAP1R1 (gene) | precuneus |  | -0.01 | 0.03 | -0.07 | 0.06 |
| PTE x ADCYAP1R1 (gene) | paracentral lobule and sulcus |  | -0.04 | 0.03 | -0.10 | 0.03 |
| PTE x ADCYAP1R1 (gene) | subcentral gyrus and sulci |  | 0.02 | 0.03 | -0.05 | 0.08 |
| PTE x ADCYAP1R1 (gene) | marginal branch (or part) of the cingulate sulcus |  | -0.04 | 0.04 | -0.11 | 0.04 |
| PTE x ADCYAP1R1 (gene) | subparietal sulcus |  | -0.02 | 0.04 | -0.11 | 0.07 |
| PTE x ADCYAP1R1 (gene) | calcarine sulcus |  | 0.00 | 0.04 | -0.06 | 0.07 |
| PTE x ADCYAP1R1 (gene) | medial occipitotemporal sulcus (or collateral sulcus) |  | 0.00 | 0.04 | -0.07 | 0.08 |
| PTE x ADCYAP1R1 (gene) | lateral occipito-temporal (or fusiform) sulcus |  | -0.05 | 0.03 | -0.12 | 0.01 |
| PTE x ADCYAP1R1 (gene) | subcallosal area or gyrus |  | -0.04 | 0.04 | -0.11 | 0.04 |
| PTE x ADCYAP1R1 (gene) | pericallosal sulcus or sulcus of the corpus callosum |  | 0.04 | 0.04 | -0.05 | 0.12 |
| PTE x ADCYAP1R1 (gene) | anterior (ACC) |  | 0.02 | 0.04 | -0.06 | 0.10 |
| PTE x ADCYAP1R1 (gene) | middle-anterior (aMCC) |  | 0.01 | 0.05 | -0.07 | 0.10 |
| PTE x ADCYAP1R1 (gene) | middle-posterior (pMCC) |  | 0.01 | 0.03 | -0.05 | 0.07 |
| PTE x ADCYAP1R1 (gene) | posterior-dorsal (dPCC) |  | 0.00 | 0.04 | -0.06 | 0.07 |
| PTE x ADCYAP1R1 (gene) | posterior-ventral (vPCC or isthmus) |  | -0.01 | 0.04 | -0.09 | 0.06 |
| PTE x BDNF (gene) | precentral gyrus |  | -0.03 | 0.04 | -0.10 | 0.04 |
| PTE x BDNF (gene) | subcentral gyrus |  | 0.04 | 0.03 | -0.02 | 0.11 |
| PTE x BDNF (gene) | inferior frontal gyrus (or F3) |  | -0.01 | 0.03 | -0.07 | 0.06 |
| PTE x BDNF (gene) | triangular part of the inferior frontal gyrus |  | 0.02 | 0.03 | -0.04 | 0.08 |
| PTE x BDNF (gene) | opercular part of the inferior frontal gyrus |  | 0.02 | 0.03 | -0.04 | 0.08 |
| PTE x BDNF (gene) | orbital part |  | -0.01 | 0.03 | -0.08 | 0.06 |
| PTE x BDNF (gene) | middle frontal gyrus (or F2) |  | 0.03 | 0.03 | -0.03 | 0.09 |
| PTE x BDNF (gene) | superior frontal gyrus | * | 0.06 | 0.03 | 0.00 | 0.12 |
| PTE x BDNF (gene) | gyrus rectus |  | 0.05 | 0.04 | -0.03 | 0.12 |
| PTE x BDNF (gene) | transverse frontopolar gyrus or gyri | * | 0.08 | 0.03 | 0.01 | 0.14 |
| PTE x BDNF (gene) | medial orbital sulcus |  | 0.06 | 0.03 | 0.00 | 0.13 |
| PTE x BDNF (gene) | 4 orbital gyri |  | 0.03 | 0.03 | -0.03 | 0.08 |
| PTE x BDNF (gene) | superior (circular sulcus of the insula) |  | -0.02 | 0.04 | -0.09 | 0.06 |
| PTE x BDNF (gene) | anterior (circular sulcus of the insula) |  | 0.03 | 0.04 | -0.05 | 0.11 |
| PTE x BDNF (gene) | inferior (circular sulcus of the insula) |  | -0.05 | 0.03 | -0.12 | 0.02 |
| PTE x BDNF (gene) | vertical ramus of anterior segment of lateral sulcus |  | -0.01 | 0.03 | -0.07 | 0.06 |
| PTE x BDNF (gene) | horizontal ramus of anterior segment of lateral sulcus |  | 0.02 | 0.04 | -0.05 | 0.09 |
| PTE x BDNF (gene) | posterior segment of the lateral sulcus |  | 0.07 | 0.03 | 0.00 | 0.13 |
| PTE x BDNF (gene) | the short insular gyri |  | 0.01 | 0.04 | -0.06 | 0.08 |
| PTE x BDNF (gene) | long insular gyrus |  | 0.01 | 0.04 | -0.06 | 0.08 |
| PTE x BDNF (gene) | transverse temporal gyrus (or Heschl's gyrus) |  | 0.02 | 0.04 | -0.05 | 0.09 |
| PTE x BDNF (gene) | planum temporale or temporal plane of the superior temporal gyrus |  | 0.02 | 0.03 | -0.05 | 0.09 |
| PTE x BDNF (gene) | planum polare of the superior temporal gyrus |  | -0.03 | 0.04 | -0.10 | 0.05 |
| PTE x BDNF (gene) | lateral aspect of the superior temporal gyrus |  | -0.05 | 0.04 | -0.12 | 0.03 |
| PTE x BDNF (gene) | middle temporal gyrus |  | -0.03 | 0.03 | -0.09 | 0.04 |
| PTE x BDNF (gene) | superior occipital gyrus (O1) |  | 0.03 | 0.04 | -0.05 | 0.10 |
| PTE x BDNF (gene) | middle occipital gyrus (O2, lateral occipital gyrus) |  | -0.01 | 0.04 | -0.08 | 0.06 |
| PTE x BDNF (gene) | inferior temporal gyrus (T3) |  | 0.00 | 0.04 | -0.08 | 0.07 |
| PTE x BDNF (gene) | lateral occipito-temporal gyrus (fusiform gyrus, O4-T4) |  | 0.00 | 0.03 | -0.07 | 0.07 |
| PTE x BDNF (gene) | lingual gyrus (O5) |  | -0.01 | 0.04 | -0.09 | 0.07 |
| PTE x BDNF (gene) | parahippocampal gyrus (or T5) |  | 0.01 | 0.04 | -0.07 | 0.09 |
| PTE x BDNF (gene) | cuneus (O6) |  | 0.03 | 0.04 | -0.06 | 0.11 |
| PTE x BDNF (gene) | occipital pole |  | 0.02 | 0.04 | -0.05 | 0.09 |
| PTE x BDNF (gene) | temporal pole |  | 0.00 | 0.03 | -0.07 | 0.07 |
| PTE x BDNF (gene) | postcentral gyrus |  | 0.02 | 0.03 | -0.05 | 0.08 |
| PTE x BDNF (gene) | supramarginal gyrus |  | 0.04 | 0.04 | -0.03 | 0.11 |
| PTE x BDNF (gene) | angular gyrus |  | 0.01 | 0.04 | -0.05 | 0.08 |
| PTE x BDNF (gene) | superior parietal lobule (or P1) |  | 0.00 | 0.04 | -0.08 | 0.07 |
| PTE x BDNF (gene) | precuneus |  | -0.01 | 0.04 | -0.08 | 0.07 |
| PTE x BDNF (gene) | paracentral lobule and sulcus |  | 0.07 | 0.03 | 0.00 | 0.13 |
| PTE x BDNF (gene) | subcentral gyrus and sulci |  | 0.04 | 0.03 | -0.02 | 0.11 |
| PTE x BDNF (gene) | marginal branch (or part) of the cingulate sulcus |  | 0.01 | 0.04 | -0.07 | 0.08 |
| PTE x BDNF (gene) | subparietal sulcus |  | 0.02 | 0.04 | -0.06 | 0.10 |
| PTE x BDNF (gene) | calcarine sulcus |  | 0.02 | 0.04 | -0.06 | 0.09 |
| PTE x BDNF (gene) | medial occipitotemporal sulcus (or collateral sulcus) |  | -0.04 | 0.04 | -0.12 | 0.03 |
| PTE x BDNF (gene) | lateral occipito-temporal (or fusiform) sulcus | * | 0.07 | 0.04 | 0.00 | 0.15 |
| PTE x BDNF (gene) | subcallosal area or gyrus |  | -0.04 | 0.04 | -0.12 | 0.04 |
| PTE x BDNF (gene) | pericallosal sulcus or sulcus of the corpus callosum |  | -0.01 | 0.05 | -0.10 | 0.08 |
| PTE x BDNF (gene) | anterior (ACC) |  | 0.06 | 0.04 | -0.02 | 0.13 |
| PTE x BDNF (gene) | middle-anterior (aMCC) |  | 0.03 | 0.04 | -0.06 | 0.11 |
| PTE x BDNF (gene) | middle-posterior (pMCC) |  | 0.03 | 0.04 | -0.04 | 0.10 |
| PTE x BDNF (gene) | posterior-dorsal (dPCC) |  | 0.04 | 0.04 | -0.03 | 0.11 |
| PTE x BDNF (gene) | posterior-ventral (vPCC or isthmus) |  | -0.02 | 0.04 | -0.10 | 0.06 |
| PTE x COMT (gene) | precentral gyrus |  | 0.05 | 0.04 | -0.02 | 0.13 |
| PTE x COMT (gene) | subcentral gyrus | * | 0.12 | 0.03 | 0.05 | 0.18 |
| PTE x COMT (gene) | inferior frontal gyrus (or F3) | * | 0.08 | 0.04 | 0.01 | 0.15 |
| PTE x COMT (gene) | triangular part of the inferior frontal gyrus |  | 0.02 | 0.03 | -0.03 | 0.08 |
| PTE x COMT (gene) | opercular part of the inferior frontal gyrus |  | 0.05 | 0.03 | -0.01 | 0.12 |
| PTE x COMT (gene) | orbital part | * | 0.09 | 0.03 | 0.03 | 0.15 |
| PTE x COMT (gene) | middle frontal gyrus (or F2) |  | 0.04 | 0.03 | -0.03 | 0.10 |
| PTE x COMT (gene) | superior frontal gyrus |  | 0.05 | 0.03 | -0.02 | 0.11 |
| PTE x COMT (gene) | gyrus rectus |  | 0.03 | 0.04 | -0.04 | 0.11 |
| PTE x COMT (gene) | transverse frontopolar gyrus or gyri |  | 0.04 | 0.03 | -0.02 | 0.10 |
| PTE x COMT (gene) | medial orbital sulcus |  | 0.01 | 0.03 | -0.05 | 0.08 |
| PTE x COMT (gene) | 4 orbital gyri | * | 0.11 | 0.03 | 0.05 | 0.17 |
| PTE x COMT (gene) | superior (circular sulcus of the insula) |  | 0.04 | 0.03 | -0.03 | 0.10 |
| PTE x COMT (gene) | anterior (circular sulcus of the insula) |  | 0.04 | 0.04 | -0.03 | 0.12 |
| PTE x COMT (gene) | inferior (circular sulcus of the insula) |  | 0.04 | 0.03 | -0.02 | 0.10 |
| PTE x COMT (gene) | vertical ramus of anterior segment of lateral sulcus | * | 0.08 | 0.04 | 0.01 | 0.15 |
| PTE x COMT (gene) | horizontal ramus of anterior segment of lateral sulcus |  | 0.05 | 0.04 | -0.02 | 0.12 |
| PTE x COMT (gene) | posterior segment of the lateral sulcus |  | 0.02 | 0.03 | -0.04 | 0.08 |
| PTE x COMT (gene) | the short insular gyri | * | 0.09 | 0.04 | 0.02 | 0.16 |
| PTE x COMT (gene) | long insular gyrus |  | 0.07 | 0.04 | -0.01 | 0.13 |
| PTE x COMT (gene) | transverse temporal gyrus (or Heschl's gyrus) |  | 0.04 | 0.03 | -0.03 | 0.11 |
| PTE x COMT (gene) | planum temporale or temporal plane of the superior temporal gyrus |  | 0.05 | 0.03 | -0.01 | 0.12 |
| PTE x COMT (gene) | planum polare of the superior temporal gyrus |  | 0.07 | 0.04 | 0.00 | 0.14 |
| PTE x COMT (gene) | lateral aspect of the superior temporal gyrus |  | 0.06 | 0.04 | -0.01 | 0.13 |
| PTE x COMT (gene) | middle temporal gyrus | * | 0.09 | 0.03 | 0.02 | 0.16 |
| PTE x COMT (gene) | superior occipital gyrus (O1) |  | 0.02 | 0.04 | -0.06 | 0.10 |
| PTE x COMT (gene) | middle occipital gyrus (O2, lateral occipital gyrus) |  | 0.03 | 0.04 | -0.05 | 0.11 |
| PTE x COMT (gene) | inferior temporal gyrus (T3) | * | 0.07 | 0.04 | 0.01 | 0.15 |
| PTE x COMT (gene) | lateral occipito-temporal gyrus (fusiform gyrus, O4-T4) |  | 0.06 | 0.03 | 0.00 | 0.12 |
| PTE x COMT (gene) | lingual gyrus (O5) |  | 0.06 | 0.04 | -0.01 | 0.13 |
| PTE x COMT (gene) | parahippocampal gyrus (or T5) |  | 0.03 | 0.04 | -0.04 | 0.11 |
| PTE x COMT (gene) | cuneus (O6) |  | 0.04 | 0.04 | -0.03 | 0.12 |
| PTE x COMT (gene) | occipital pole |  | 0.04 | 0.04 | -0.03 | 0.11 |
| PTE x COMT (gene) | temporal pole | * | 0.10 | 0.03 | 0.03 | 0.17 |
| PTE x COMT (gene) | postcentral gyrus |  | 0.06 | 0.03 | -0.01 | 0.13 |
| PTE x COMT (gene) | supramarginal gyrus |  | 0.03 | 0.04 | -0.04 | 0.09 |
| PTE x COMT (gene) | angular gyrus |  | 0.05 | 0.04 | -0.02 | 0.12 |
| PTE x COMT (gene) | superior parietal lobule (or P1) |  | 0.00 | 0.04 | -0.07 | 0.07 |
| PTE x COMT (gene) | precuneus |  | 0.01 | 0.04 | -0.06 | 0.09 |
| PTE x COMT (gene) | paracentral lobule and sulcus |  | 0.05 | 0.03 | -0.02 | 0.11 |
| PTE x COMT (gene) | subcentral gyrus and sulci | * | 0.12 | 0.03 | 0.05 | 0.18 |
| PTE x COMT (gene) | marginal branch (or part) of the cingulate sulcus |  | -0.07 | 0.03 | -0.13 | 0.00 |
| PTE x COMT (gene) | subparietal sulcus |  | -0.02 | 0.04 | -0.09 | 0.06 |
| PTE x COMT (gene) | calcarine sulcus |  | 0.01 | 0.03 | -0.05 | 0.08 |
| PTE x COMT (gene) | medial occipitotemporal sulcus (or collateral sulcus) |  | 0.05 | 0.04 | -0.03 | 0.12 |
| PTE x COMT (gene) | lateral occipito-temporal (or fusiform) sulcus |  | 0.00 | 0.04 | -0.07 | 0.07 |
| PTE x COMT (gene) | subcallosal area or gyrus |  | -0.02 | 0.04 | -0.10 | 0.06 |
| PTE x COMT (gene) | pericallosal sulcus or sulcus of the corpus callosum |  | 0.00 | 0.04 | -0.09 | 0.08 |
| PTE x COMT (gene) | anterior (ACC) |  | 0.03 | 0.03 | -0.04 | 0.10 |
| PTE x COMT (gene) | middle-anterior (aMCC) |  | 0.04 | 0.04 | -0.03 | 0.12 |
| PTE x COMT (gene) | middle-posterior (pMCC) |  | 0.01 | 0.04 | -0.06 | 0.08 |
| PTE x COMT (gene) | posterior-dorsal (dPCC) |  | -0.03 | 0.04 | -0.10 | 0.04 |
| PTE x COMT (gene) | posterior-ventral (vPCC or isthmus) |  | -0.01 | 0.04 | -0.09 | 0.07 |
| PTE x HTR3A (gene) | precentral gyrus |  | 0.03 | 0.03 | -0.03 | 0.09 |
| PTE x HTR3A (gene) | subcentral gyrus |  | 0.03 | 0.03 | -0.02 | 0.08 |
| PTE x HTR3A (gene) | inferior frontal gyrus (or F3) |  | 0.00 | 0.04 | -0.08 | 0.07 |
| PTE x HTR3A (gene) | triangular part of the inferior frontal gyrus |  | 0.01 | 0.03 | -0.04 | 0.07 |
| PTE x HTR3A (gene) | opercular part of the inferior frontal gyrus |  | 0.01 | 0.03 | -0.04 | 0.07 |
| PTE x HTR3A (gene) | orbital part |  | 0.03 | 0.03 | -0.03 | 0.09 |
| PTE x HTR3A (gene) | middle frontal gyrus (or F2) |  | 0.00 | 0.02 | -0.04 | 0.05 |
| PTE x HTR3A (gene) | superior frontal gyrus |  | 0.00 | 0.02 | -0.05 | 0.05 |
| PTE x HTR3A (gene) | gyrus rectus |  | -0.02 | 0.03 | -0.08 | 0.05 |
| PTE x HTR3A (gene) | transverse frontopolar gyrus or gyri |  | -0.01 | 0.03 | -0.06 | 0.04 |
| PTE x HTR3A (gene) | medial orbital sulcus |  | -0.01 | 0.03 | -0.07 | 0.05 |
| PTE x HTR3A (gene) | 4 orbital gyri |  | -0.04 | 0.02 | -0.08 | 0.01 |
| PTE x HTR3A (gene) | superior (circular sulcus of the insula) |  | 0.05 | 0.03 | -0.01 | 0.11 |
| PTE x HTR3A (gene) | anterior (circular sulcus of the insula) |  | -0.03 | 0.04 | -0.10 | 0.05 |
| PTE x HTR3A (gene) | inferior (circular sulcus of the insula) |  | -0.01 | 0.03 | -0.06 | 0.05 |
| PTE x HTR3A (gene) | vertical ramus of anterior segment of lateral sulcus |  | 0.00 | 0.04 | -0.08 | 0.07 |
| PTE x HTR3A (gene) | horizontal ramus of anterior segment of lateral sulcus |  | 0.00 | 0.04 | -0.07 | 0.08 |
| PTE x HTR3A (gene) | posterior segment of the lateral sulcus |  | -0.03 | 0.02 | -0.07 | 0.02 |
| PTE x HTR3A (gene) | the short insular gyri |  | 0.04 | 0.04 | -0.04 | 0.12 |
| PTE x HTR3A (gene) | long insular gyrus |  | 0.02 | 0.03 | -0.04 | 0.09 |
| PTE x HTR3A (gene) | transverse temporal gyrus (or Heschl's gyrus) |  | -0.07 | 0.03 | -0.12 | 0.00 |
| PTE x HTR3A (gene) | planum temporale or temporal plane of the superior temporal gyrus |  | -0.01 | 0.03 | -0.07 | 0.06 |
| PTE x HTR3A (gene) | planum polare of the superior temporal gyrus |  | -0.02 | 0.04 | -0.09 | 0.05 |
| PTE x HTR3A (gene) | lateral aspect of the superior temporal gyrus |  | -0.05 | 0.03 | -0.11 | 0.01 |
| PTE x HTR3A (gene) | middle temporal gyrus |  | 0.03 | 0.03 | -0.04 | 0.09 |
| PTE x HTR3A (gene) | superior occipital gyrus (O1) |  | -0.06 | 0.04 | -0.13 | 0.03 |
| PTE x HTR3A (gene) | middle occipital gyrus (O2, lateral occipital gyrus) | * | -0.08 | 0.04 | -0.14 | 0.00 |
| PTE x HTR3A (gene) | inferior temporal gyrus (T3) |  | -0.03 | 0.03 | -0.09 | 0.03 |
| PTE x HTR3A (gene) | lateral occipito-temporal gyrus (fusiform gyrus, O4-T4) |  | -0.01 | 0.03 | -0.07 | 0.06 |
| PTE x HTR3A (gene) | lingual gyrus (O5) |  | -0.04 | 0.04 | -0.11 | 0.04 |
| PTE x HTR3A (gene) | parahippocampal gyrus (or T5) |  | -0.02 | 0.03 | -0.09 | 0.04 |
| PTE x HTR3A (gene) | cuneus (O6) |  | 0.00 | 0.04 | -0.08 | 0.08 |
| PTE x HTR3A (gene) | occipital pole |  | 0.05 | 0.04 | -0.03 | 0.13 |
| PTE x HTR3A (gene) | temporal pole |  | 0.03 | 0.03 | -0.02 | 0.08 |
| PTE x HTR3A (gene) | postcentral gyrus |  | 0.02 | 0.03 | -0.04 | 0.08 |
| PTE x HTR3A (gene) | supramarginal gyrus |  | -0.02 | 0.03 | -0.08 | 0.04 |
| PTE x HTR3A (gene) | angular gyrus |  | -0.04 | 0.04 | -0.11 | 0.04 |
| PTE x HTR3A (gene) | superior parietal lobule (or P1) |  | -0.01 | 0.03 | -0.07 | 0.06 |
| PTE x HTR3A (gene) | precuneus |  | 0.01 | 0.03 | -0.05 | 0.07 |
| PTE x HTR3A (gene) | paracentral lobule and sulcus |  | -0.03 | 0.03 | -0.09 | 0.03 |
| PTE x HTR3A (gene) | subcentral gyrus and sulci |  | 0.03 | 0.03 | -0.02 | 0.08 |
| PTE x HTR3A (gene) | marginal branch (or part) of the cingulate sulcus |  | 0.04 | 0.03 | -0.02 | 0.11 |
| PTE x HTR3A (gene) | subparietal sulcus |  | 0.03 | 0.03 | -0.03 | 0.10 |
| PTE x HTR3A (gene) | calcarine sulcus |  | 0.04 | 0.03 | -0.01 | 0.09 |
| PTE x HTR3A (gene) | medial occipitotemporal sulcus (or collateral sulcus) | * | -0.07 | 0.03 | -0.12 | -0.01 |
| PTE x HTR3A (gene) | lateral occipito-temporal (or fusiform) sulcus |  | -0.03 | 0.04 | -0.10 | 0.06 |
| PTE x HTR3A (gene) | subcallosal area or gyrus |  | 0.02 | 0.04 | -0.06 | 0.09 |
| PTE x HTR3A (gene) | pericallosal sulcus or sulcus of the corpus callosum |  | -0.03 | 0.04 | -0.11 | 0.05 |
| PTE x HTR3A (gene) | anterior (ACC) |  | -0.02 | 0.03 | -0.08 | 0.04 |
| PTE x HTR3A (gene) | middle-anterior (aMCC) |  | 0.00 | 0.03 | -0.05 | 0.05 |
| PTE x HTR3A (gene) | middle-posterior (pMCC) |  | 0.01 | 0.03 | -0.05 | 0.06 |
| PTE x HTR3A (gene) | posterior-dorsal (dPCC) |  | -0.03 | 0.03 | -0.08 | 0.04 |
| PTE x HTR3A (gene) | posterior-ventral (vPCC or isthmus) |  | -0.02 | 0.04 | -0.09 | 0.05 |
| PTE x DRD2 (gene) | precentral gyrus |  | -0.01 | 0.03 | -0.08 | 0.05 |
| PTE x DRD2 (gene) | subcentral gyrus |  | 0.00 | 0.04 | -0.08 | 0.07 |
| PTE x DRD2 (gene) | inferior frontal gyrus (or F3) |  | -0.01 | 0.03 | -0.07 | 0.06 |
| PTE x DRD2 (gene) | triangular part of the inferior frontal gyrus |  | 0.01 | 0.03 | -0.05 | 0.07 |
| PTE x DRD2 (gene) | opercular part of the inferior frontal gyrus |  | -0.04 | 0.03 | -0.10 | 0.02 |
| PTE x DRD2 (gene) | orbital part |  | -0.02 | 0.03 | -0.09 | 0.04 |
| PTE x DRD2 (gene) | middle frontal gyrus (or F2) |  | -0.05 | 0.04 | -0.12 | 0.02 |
| PTE x DRD2 (gene) | superior frontal gyrus |  | -0.02 | 0.03 | -0.09 | 0.05 |
| PTE x DRD2 (gene) | gyrus rectus |  | 0.02 | 0.03 | -0.05 | 0.08 |
| PTE x DRD2 (gene) | transverse frontopolar gyrus or gyri |  | -0.01 | 0.03 | -0.08 | 0.06 |
| PTE x DRD2 (gene) | medial orbital sulcus |  | 0.04 | 0.04 | -0.04 | 0.10 |
| PTE x DRD2 (gene) | 4 orbital gyri |  | 0.01 | 0.03 | -0.05 | 0.07 |
| PTE x DRD2 (gene) | superior (circular sulcus of the insula) |  | 0.00 | 0.03 | -0.07 | 0.06 |
| PTE x DRD2 (gene) | anterior (circular sulcus of the insula) |  | 0.03 | 0.04 | -0.05 | 0.11 |
| PTE x DRD2 (gene) | inferior (circular sulcus of the insula) |  | 0.00 | 0.03 | -0.06 | 0.05 |
| PTE x DRD2 (gene) | vertical ramus of anterior segment of lateral sulcus |  | -0.01 | 0.03 | -0.07 | 0.06 |
| PTE x DRD2 (gene) | horizontal ramus of anterior segment of lateral sulcus |  | -0.04 | 0.04 | -0.12 | 0.03 |
| PTE x DRD2 (gene) | posterior segment of the lateral sulcus |  | 0.05 | 0.03 | -0.01 | 0.12 |
| PTE x DRD2 (gene) | the short insular gyri |  | 0.00 | 0.03 | -0.07 | 0.06 |
| PTE x DRD2 (gene) | long insular gyrus |  | 0.05 | 0.03 | -0.01 | 0.12 |
| PTE x DRD2 (gene) | transverse temporal gyrus (or Heschl's gyrus) |  | -0.02 | 0.04 | -0.09 | 0.06 |
| PTE x DRD2 (gene) | planum temporale or temporal plane of the superior temporal gyrus |  | -0.04 | 0.03 | -0.10 | 0.03 |
| PTE x DRD2 (gene) | planum polare of the superior temporal gyrus |  | -0.04 | 0.04 | -0.11 | 0.03 |
| PTE x DRD2 (gene) | lateral aspect of the superior temporal gyrus |  | -0.01 | 0.04 | -0.08 | 0.06 |
| PTE x DRD2 (gene) | middle temporal gyrus |  | -0.01 | 0.03 | -0.07 | 0.06 |
| PTE x DRD2 (gene) | superior occipital gyrus (O1) |  | 0.01 | 0.04 | -0.06 | 0.08 |
| PTE x DRD2 (gene) | middle occipital gyrus (O2, lateral occipital gyrus) |  | 0.04 | 0.04 | -0.04 | 0.12 |
| PTE x DRD2 (gene) | inferior temporal gyrus (T3) |  | 0.01 | 0.04 | -0.06 | 0.08 |
| PTE x DRD2 (gene) | lateral occipito-temporal gyrus (fusiform gyrus, O4-T4) |  | 0.00 | 0.04 | -0.07 | 0.07 |
| PTE x DRD2 (gene) | lingual gyrus (O5) |  | 0.03 | 0.03 | -0.03 | 0.10 |
| PTE x DRD2 (gene) | parahippocampal gyrus (or T5) | * | 0.08 | 0.04 | 0.00 | 0.15 |
| PTE x DRD2 (gene) | cuneus (O6) |  | 0.04 | 0.04 | -0.04 | 0.12 |
| PTE x DRD2 (gene) | occipital pole |  | 0.05 | 0.05 | -0.04 | 0.13 |
| PTE x DRD2 (gene) | temporal pole |  | -0.05 | 0.03 | -0.11 | 0.02 |
| PTE x DRD2 (gene) | postcentral gyrus |  | -0.03 | 0.04 | -0.10 | 0.04 |
| PTE x DRD2 (gene) | supramarginal gyrus |  | 0.02 | 0.04 | -0.05 | 0.09 |
| PTE x DRD2 (gene) | angular gyrus |  | 0.02 | 0.04 | -0.05 | 0.09 |
| PTE x DRD2 (gene) | superior parietal lobule (or P1) |  | 0.01 | 0.04 | -0.06 | 0.09 |
| PTE x DRD2 (gene) | precuneus |  | 0.07 | 0.04 | 0.00 | 0.13 |
| PTE x DRD2 (gene) | paracentral lobule and sulcus |  | 0.04 | 0.04 | -0.04 | 0.10 |
| PTE x DRD2 (gene) | subcentral gyrus and sulci |  | 0.00 | 0.04 | -0.08 | 0.07 |
| PTE x DRD2 (gene) | marginal branch (or part) of the cingulate sulcus |  | 0.00 | 0.03 | -0.07 | 0.06 |
| PTE x DRD2 (gene) | subparietal sulcus |  | 0.02 | 0.04 | -0.06 | 0.09 |
| PTE x DRD2 (gene) | calcarine sulcus |  | 0.06 | 0.03 | 0.00 | 0.12 |
| PTE x DRD2 (gene) | medial occipitotemporal sulcus (or collateral sulcus) |  | -0.01 | 0.03 | -0.08 | 0.06 |
| PTE x DRD2 (gene) | lateral occipito-temporal (or fusiform) sulcus |  | 0.03 | 0.04 | -0.04 | 0.11 |
| PTE x DRD2 (gene) | subcallosal area or gyrus |  | 0.00 | 0.04 | -0.08 | 0.07 |
| PTE x DRD2 (gene) | pericallosal sulcus or sulcus of the corpus callosum |  | 0.08 | 0.04 | -0.01 | 0.15 |
| PTE x DRD2 (gene) | anterior (ACC) |  | 0.00 | 0.03 | -0.07 | 0.07 |
| PTE x DRD2 (gene) | middle-anterior (aMCC) |  | -0.02 | 0.04 | -0.10 | 0.05 |
| PTE x DRD2 (gene) | middle-posterior (pMCC) |  | 0.02 | 0.04 | -0.05 | 0.09 |
| PTE x DRD2 (gene) | posterior-dorsal (dPCC) |  | 0.06 | 0.04 | -0.02 | 0.13 |
| PTE x DRD2 (gene) | posterior-ventral (vPCC or isthmus) |  | 0.04 | 0.04 | -0.04 | 0.12 |
| PTE x NR3C1 (gene) | precentral gyrus | * | 0.12 | 0.04 | 0.03 | 0.19 |
| PTE x NR3C1 (gene) | subcentral gyrus |  | 0.03 | 0.03 | -0.04 | 0.09 |
| PTE x NR3C1 (gene) | inferior frontal gyrus (or F3) |  | 0.02 | 0.03 | -0.05 | 0.08 |
| PTE x NR3C1 (gene) | triangular part of the inferior frontal gyrus |  | 0.05 | 0.03 | -0.01 | 0.10 |
| PTE x NR3C1 (gene) | opercular part of the inferior frontal gyrus |  | 0.06 | 0.03 | -0.01 | 0.12 |
| PTE x NR3C1 (gene) | orbital part |  | 0.05 | 0.03 | 0.00 | 0.11 |
| PTE x NR3C1 (gene) | middle frontal gyrus (or F2) |  | 0.06 | 0.03 | -0.01 | 0.11 |
| PTE x NR3C1 (gene) | superior frontal gyrus | * | 0.07 | 0.03 | 0.01 | 0.12 |
| PTE x NR3C1 (gene) | gyrus rectus |  | 0.03 | 0.04 | -0.04 | 0.10 |
| PTE x NR3C1 (gene) | transverse frontopolar gyrus or gyri |  | 0.03 | 0.03 | -0.03 | 0.10 |
| PTE x NR3C1 (gene) | medial orbital sulcus |  | 0.02 | 0.03 | -0.04 | 0.08 |
| PTE x NR3C1 (gene) | 4 orbital gyri |  | 0.02 | 0.03 | -0.04 | 0.07 |
| PTE x NR3C1 (gene) | superior (circular sulcus of the insula) |  | 0.02 | 0.04 | -0.05 | 0.09 |
| PTE x NR3C1 (gene) | anterior (circular sulcus of the insula) |  | 0.04 | 0.04 | -0.04 | 0.11 |
| PTE x NR3C1 (gene) | inferior (circular sulcus of the insula) |  | 0.05 | 0.03 | -0.01 | 0.11 |
| PTE x NR3C1 (gene) | vertical ramus of anterior segment of lateral sulcus |  | 0.02 | 0.03 | -0.05 | 0.08 |
| PTE x NR3C1 (gene) | horizontal ramus of anterior segment of lateral sulcus |  | 0.03 | 0.03 | -0.04 | 0.09 |
| PTE x NR3C1 (gene) | posterior segment of the lateral sulcus |  | 0.01 | 0.03 | -0.05 | 0.08 |
| PTE x NR3C1 (gene) | the short insular gyri |  | 0.04 | 0.03 | -0.02 | 0.10 |
| PTE x NR3C1 (gene) | long insular gyrus |  | 0.03 | 0.03 | -0.04 | 0.09 |
| PTE x NR3C1 (gene) | transverse temporal gyrus (or Heschl's gyrus) |  | 0.05 | 0.03 | -0.01 | 0.10 |
| PTE x NR3C1 (gene) | planum temporale or temporal plane of the superior temporal gyrus |  | 0.05 | 0.03 | -0.01 | 0.11 |
| PTE x NR3C1 (gene) | planum polare of the superior temporal gyrus |  | 0.02 | 0.04 | -0.05 | 0.09 |
| PTE x NR3C1 (gene) | lateral aspect of the superior temporal gyrus |  | 0.05 | 0.04 | -0.03 | 0.11 |
| PTE x NR3C1 (gene) | middle temporal gyrus |  | 0.04 | 0.03 | -0.03 | 0.10 |
| PTE x NR3C1 (gene) | superior occipital gyrus (O1) |  | 0.01 | 0.04 | -0.07 | 0.08 |
| PTE x NR3C1 (gene) | middle occipital gyrus (O2, lateral occipital gyrus) |  | 0.04 | 0.04 | -0.05 | 0.10 |
| PTE x NR3C1 (gene) | inferior temporal gyrus (T3) |  | 0.07 | 0.04 | -0.01 | 0.14 |
| PTE x NR3C1 (gene) | lateral occipito-temporal gyrus (fusiform gyrus, O4-T4) |  | 0.00 | 0.03 | -0.07 | 0.06 |
| PTE x NR3C1 (gene) | lingual gyrus (O5) |  | -0.03 | 0.03 | -0.10 | 0.03 |
| PTE x NR3C1 (gene) | parahippocampal gyrus (or T5) |  | 0.06 | 0.04 | -0.03 | 0.13 |
| PTE x NR3C1 (gene) | cuneus (O6) |  | 0.01 | 0.03 | -0.06 | 0.07 |
| PTE x NR3C1 (gene) | occipital pole |  | -0.01 | 0.04 | -0.09 | 0.07 |
| PTE x NR3C1 (gene) | temporal pole |  | 0.03 | 0.03 | -0.03 | 0.09 |
| PTE x NR3C1 (gene) | postcentral gyrus | * | 0.07 | 0.03 | 0.00 | 0.13 |
| PTE x NR3C1 (gene) | supramarginal gyrus |  | 0.06 | 0.03 | -0.01 | 0.12 |
| PTE x NR3C1 (gene) | angular gyrus | * | 0.08 | 0.04 | 0.00 | 0.14 |
| PTE x NR3C1 (gene) | superior parietal lobule (or P1) | * | 0.08 | 0.04 | 0.01 | 0.15 |
| PTE x NR3C1 (gene) | precuneus |  | 0.05 | 0.04 | -0.04 | 0.12 |
| PTE x NR3C1 (gene) | paracentral lobule and sulcus | * | 0.08 | 0.03 | 0.01 | 0.14 |
| PTE x NR3C1 (gene) | subcentral gyrus and sulci |  | 0.03 | 0.03 | -0.04 | 0.09 |
| PTE x NR3C1 (gene) | marginal branch (or part) of the cingulate sulcus | * | 0.08 | 0.04 | 0.00 | 0.15 |
| PTE x NR3C1 (gene) | subparietal sulcus |  | -0.01 | 0.04 | -0.10 | 0.07 |
| PTE x NR3C1 (gene) | calcarine sulcus |  | -0.02 | 0.03 | -0.08 | 0.04 |
| PTE x NR3C1 (gene) | medial occipitotemporal sulcus (or collateral sulcus) |  | 0.01 | 0.04 | -0.06 | 0.08 |
| PTE x NR3C1 (gene) | lateral occipito-temporal (or fusiform) sulcus |  | 0.01 | 0.04 | -0.06 | 0.09 |
| PTE x NR3C1 (gene) | subcallosal area or gyrus |  | 0.05 | 0.04 | -0.03 | 0.12 |
| PTE x NR3C1 (gene) | pericallosal sulcus or sulcus of the corpus callosum |  | -0.04 | 0.04 | -0.11 | 0.04 |
| PTE x NR3C1 (gene) | anterior (ACC) |  | 0.04 | 0.03 | -0.03 | 0.10 |
| PTE x NR3C1 (gene) | middle-anterior (aMCC) |  | 0.03 | 0.04 | -0.04 | 0.10 |
| PTE x NR3C1 (gene) | middle-posterior (pMCC) |  | 0.04 | 0.03 | -0.04 | 0.10 |
| PTE x NR3C1 (gene) | posterior-dorsal (dPCC) |  | 0.00 | 0.03 | -0.07 | 0.07 |
| PTE x NR3C1 (gene) | posterior-ventral (vPCC or isthmus) |  | 0.00 | 0.04 | -0.08 | 0.07 |
| PTE x OXTR (gene) | precentral gyrus |  | 0.00 | 0.03 | -0.07 | 0.07 |
| PTE x OXTR (gene) | subcentral gyrus |  | 0.02 | 0.03 | -0.05 | 0.09 |
| PTE x OXTR (gene) | inferior frontal gyrus (or F3) | * | 0.07 | 0.03 | 0.00 | 0.13 |
| PTE x OXTR (gene) | triangular part of the inferior frontal gyrus |  | 0.05 | 0.03 | -0.02 | 0.11 |
| PTE x OXTR (gene) | opercular part of the inferior frontal gyrus |  | 0.02 | 0.03 | -0.04 | 0.08 |
| PTE x OXTR (gene) | orbital part |  | -0.01 | 0.03 | -0.07 | 0.05 |
| PTE x OXTR (gene) | middle frontal gyrus (or F2) |  | 0.04 | 0.03 | -0.02 | 0.10 |
| PTE x OXTR (gene) | superior frontal gyrus |  | 0.02 | 0.03 | -0.04 | 0.09 |
| PTE x OXTR (gene) | gyrus rectus |  | 0.03 | 0.03 | -0.04 | 0.10 |
| PTE x OXTR (gene) | transverse frontopolar gyrus or gyri |  | 0.01 | 0.03 | -0.05 | 0.08 |
| PTE x OXTR (gene) | medial orbital sulcus |  | 0.02 | 0.04 | -0.05 | 0.09 |
| PTE x OXTR (gene) | 4 orbital gyri | * | -0.08 | 0.03 | -0.14 | -0.01 |
| PTE x OXTR (gene) | superior (circular sulcus of the insula) |  | 0.01 | 0.03 | -0.05 | 0.08 |
| PTE x OXTR (gene) | anterior (circular sulcus of the insula) |  | 0.03 | 0.04 | -0.04 | 0.10 |
| PTE x OXTR (gene) | inferior (circular sulcus of the insula) |  | 0.02 | 0.03 | -0.04 | 0.08 |
| PTE x OXTR (gene) | vertical ramus of anterior segment of lateral sulcus | * | 0.07 | 0.03 | 0.00 | 0.13 |
| PTE x OXTR (gene) | horizontal ramus of anterior segment of lateral sulcus |  | 0.01 | 0.04 | -0.07 | 0.08 |
| PTE x OXTR (gene) | posterior segment of the lateral sulcus |  | 0.02 | 0.03 | -0.05 | 0.08 |
| PTE x OXTR (gene) | the short insular gyri |  | 0.03 | 0.03 | -0.04 | 0.09 |
| PTE x OXTR (gene) | long insular gyrus | * | 0.10 | 0.03 | 0.03 | 0.16 |
| PTE x OXTR (gene) | transverse temporal gyrus (or Heschl's gyrus) |  | -0.01 | 0.03 | -0.07 | 0.06 |
| PTE x OXTR (gene) | planum temporale or temporal plane of the superior temporal gyrus |  | 0.02 | 0.03 | -0.05 | 0.08 |
| PTE x OXTR (gene) | planum polare of the superior temporal gyrus |  | 0.04 | 0.03 | -0.02 | 0.11 |
| PTE x OXTR (gene) | lateral aspect of the superior temporal gyrus |  | 0.04 | 0.03 | -0.03 | 0.11 |
| PTE x OXTR (gene) | middle temporal gyrus |  | -0.04 | 0.03 | -0.10 | 0.02 |
| PTE x OXTR (gene) | superior occipital gyrus (O1) |  | 0.00 | 0.04 | -0.08 | 0.07 |
| PTE x OXTR (gene) | middle occipital gyrus (O2, lateral occipital gyrus) |  | 0.00 | 0.04 | -0.07 | 0.07 |
| PTE x OXTR (gene) | inferior temporal gyrus (T3) |  | 0.04 | 0.03 | -0.03 | 0.11 |
| PTE x OXTR (gene) | lateral occipito-temporal gyrus (fusiform gyrus, O4-T4) |  | 0.05 | 0.03 | -0.02 | 0.11 |
| PTE x OXTR (gene) | lingual gyrus (O5) |  | 0.04 | 0.03 | -0.02 | 0.10 |
| PTE x OXTR (gene) | parahippocampal gyrus (or T5) |  | 0.02 | 0.04 | -0.05 | 0.10 |
| PTE x OXTR (gene) | cuneus (O6) |  | 0.05 | 0.03 | -0.02 | 0.11 |
| PTE x OXTR (gene) | occipital pole |  | -0.01 | 0.04 | -0.09 | 0.06 |
| PTE x OXTR (gene) | temporal pole |  | 0.03 | 0.03 | -0.03 | 0.09 |
| PTE x OXTR (gene) | postcentral gyrus |  | 0.03 | 0.03 | -0.04 | 0.10 |
| PTE x OXTR (gene) | supramarginal gyrus |  | 0.05 | 0.03 | -0.02 | 0.11 |
| PTE x OXTR (gene) | angular gyrus |  | 0.01 | 0.03 | -0.05 | 0.07 |
| PTE x OXTR (gene) | superior parietal lobule (or P1) |  | -0.01 | 0.03 | -0.07 | 0.06 |
| PTE x OXTR (gene) | precuneus |  | -0.01 | 0.03 | -0.07 | 0.05 |
| PTE x OXTR (gene) | paracentral lobule and sulcus |  | 0.03 | 0.04 | -0.04 | 0.10 |
| PTE x OXTR (gene) | subcentral gyrus and sulci |  | 0.02 | 0.03 | -0.05 | 0.09 |
| PTE x OXTR (gene) | marginal branch (or part) of the cingulate sulcus |  | -0.01 | 0.03 | -0.08 | 0.05 |
| PTE x OXTR (gene) | subparietal sulcus |  | 0.03 | 0.04 | -0.05 | 0.09 |
| PTE x OXTR (gene) | calcarine sulcus | * | 0.07 | 0.03 | 0.02 | 0.13 |
| PTE x OXTR (gene) | medial occipitotemporal sulcus (or collateral sulcus) | * | 0.07 | 0.03 | 0.00 | 0.13 |
| PTE x OXTR (gene) | lateral occipito-temporal (or fusiform) sulcus |  | 0.01 | 0.04 | -0.06 | 0.08 |
| PTE x OXTR (gene) | subcallosal area or gyrus |  | -0.02 | 0.04 | -0.09 | 0.05 |
| PTE x OXTR (gene) | pericallosal sulcus or sulcus of the corpus callosum |  | 0.02 | 0.04 | -0.06 | 0.11 |
| PTE x OXTR (gene) | anterior (ACC) |  | 0.01 | 0.03 | -0.05 | 0.08 |
| PTE x OXTR (gene) | middle-anterior (aMCC) |  | 0.02 | 0.03 | -0.06 | 0.08 |
| PTE x OXTR (gene) | middle-posterior (pMCC) |  | 0.04 | 0.03 | -0.03 | 0.10 |
| PTE x OXTR (gene) | posterior-dorsal (dPCC) |  | 0.01 | 0.03 | -0.05 | 0.08 |
| PTE x OXTR (gene) | posterior-ventral (vPCC or isthmus) |  | 0.01 | 0.04 | -0.06 | 0.08 |
