# Supplementary material for: A knowledge-based multivariate statistical method for examining gene-brain-behavioral/cognitive relationships: Imaging genetics generalized structured component analysis
Source: PLoS One. 2021 Mar 10;16(3):e0247592. doi: 10.1371/journal.pone.0247592 (PMC7946325; doi:10.1371/journal.pone.0247592)
Supplement: S2 Table — (DOCX) [file pone.0247592.s002.docx]

S2 Table 1. Biases, standard deviations (SD), and root mean square errors (RMSE) of loadings estimated from IG-GSCA over different sample sizes in the simulation study.

S2 Table 2. Biases, standard deviations (SD), and root mean square errors (RMSE) of path coefficients estimated from IG-GSCA over different sample sizes in the simulation study.

S2 Table 1. Biases, standard deviations (SD), and root mean square errors (RMSE) of loadings estimated from IG-GSCA over different sample sizes in the simulation study.

|  |  |  | Bias | | | | SD | | | | RMSE | | | |
| --- | --- | --- | --- | --- | --- | --- | --- | --- | --- | --- | --- | --- | --- | --- |
|  |  | True value | N=250 | N=500 | N=1000 | N=2000 | N=250 | N=500 | N=1000 | N=2000 | N=250 | N=500 | N=1000 | N=2000 |
| γ*_g_*_1_ | z*_g_*_1_ | 1.00 | 0.00 | 0.00 | 0.00 | 0.00 | 0.00 | 0.00 | 0.00 | 0.00 | 0.00 | 0.00 | 0.00 | 0.00 |
| γ*_g_*_2_ | z*_g_*_2,1_ | 0.87 | 0.00 | 0.00 | 0.00 | 0.00 | 0.02 | 0.01 | 0.01 | 0.01 | 0.02 | 0.01 | 0.01 | 0.01 |
| γ*_g_*_2_ | z*_g_*_2,2_ | 0.87 | 0.00 | 0.00 | 0.00 | 0.00 | 0.02 | 0.01 | 0.01 | 0.01 | 0.02 | 0.01 | 0.01 | 0.01 |
| γ*_g_*_3_ | z*_g_*_3,1_ | 0.84 | 0.00 | 0.00 | 0.00 | 0.00 | 0.02 | 0.02 | 0.01 | 0.01 | 0.02 | 0.02 | 0.01 | 0.01 |
| γ*_g_*_3_ | z*_g_*_3,2_ | 0.87 | 0.00 | 0.00 | 0.00 | 0.00 | 0.02 | 0.01 | 0.01 | 0.01 | 0.02 | 0.01 | 0.01 | 0.01 |
| γ*_g_*_3_ | z*_g_*_3,3_ | 0.81 | 0.00 | 0.00 | 0.00 | 0.00 | 0.03 | 0.02 | 0.01 | 0.01 | 0.03 | 0.02 | 0.01 | 0.01 |
| γ*_g_*_3_ | z*_g_*_3,4_ | 0.77 | 0.00 | 0.00 | 0.00 | 0.00 | 0.03 | 0.02 | 0.02 | 0.01 | 0.03 | 0.02 | 0.02 | 0.01 |
| γ*_g_*_4_ | z*_g_*_4_ | 1.00 | 0.00 | 0.00 | 0.00 | 0.00 | 0.00 | 0.00 | 0.00 | 0.00 | 0.00 | 0.00 | 0.00 | 0.00 |
| γ*_g_*_5_ | z*_g_*_5,1_ | 0.86 | 0.00 | 0.00 | 0.00 | 0.00 | 0.02 | 0.01 | 0.01 | 0.01 | 0.02 | 0.01 | 0.01 | 0.01 |
| γ*_g_*_5_ | z*_g_*_5,2_ | 0.86 | 0.00 | 0.00 | 0.00 | 0.00 | 0.02 | 0.01 | 0.01 | 0.01 | 0.02 | 0.01 | 0.01 | 0.01 |
| γ*_g_*_6_ | z*_g_*_6,1_ | 0.84 | 0.00 | 0.00 | 0.00 | 0.00 | 0.02 | 0.02 | 0.01 | 0.01 | 0.02 | 0.02 | 0.01 | 0.01 |
| γ*_g_*_6_ | z*_g_*_6,2_ | 0.87 | 0.00 | 0.00 | 0.00 | 0.00 | 0.02 | 0.01 | 0.01 | 0.01 | 0.02 | 0.01 | 0.01 | 0.01 |
| γ*_g_*_6_ | z*_g_*_6,3_ | 0.80 | 0.00 | 0.00 | 0.00 | 0.00 | 0.03 | 0.02 | 0.01 | 0.01 | 0.03 | 0.02 | 0.01 | 0.01 |
| γ*_g_*_6_ | z*_g_*_6,4_ | 0.77 | 0.00 | 0.00 | 0.00 | 0.00 | 0.03 | 0.02 | 0.02 | 0.01 | 0.03 | 0.02 | 0.02 | 0.01 |
| γ*_g_*_7_ | z*_g_*_7_ | 1.00 | 0.00 | 0.00 | 0.00 | 0.00 | 0.00 | 0.00 | 0.00 | 0.00 | 0.00 | 0.00 | 0.00 | 0.00 |
| γ*_g_*_8_ | z*_g_*_8,1_ | 0.86 | 0.00 | 0.00 | 0.00 | 0.00 | 0.02 | 0.01 | 0.01 | 0.01 | 0.02 | 0.01 | 0.01 | 0.01 |
| γ*_g_*_8_ | z*_g_*_8,2_ | 0.86 | 0.00 | 0.00 | 0.00 | 0.00 | 0.02 | 0.01 | 0.01 | 0.01 | 0.02 | 0.01 | 0.01 | 0.01 |
| γ*_g_*_9_ | z*_g_*_9,1_ | 0.84 | 0.00 | 0.00 | 0.00 | 0.00 | 0.02 | 0.02 | 0.01 | 0.01 | 0.02 | 0.02 | 0.01 | 0.01 |
| γ*_g_*_9_ | z*_g_*_9,2_ | 0.87 | 0.00 | 0.00 | 0.00 | 0.00 | 0.02 | 0.01 | 0.01 | 0.01 | 0.02 | 0.01 | 0.01 | 0.01 |
| γ*_g_*_9_ | z*_g_*_9,3_ | 0.81 | 0.00 | 0.00 | 0.00 | 0.00 | 0.03 | 0.02 | 0.01 | 0.01 | 0.03 | 0.02 | 0.01 | 0.01 |
| γ*_g_*_9_ | z*_g_*_9,4_ | 0.77 | 0.00 | 0.00 | 0.00 | 0.00 | 0.03 | 0.02 | 0.02 | 0.01 | 0.03 | 0.02 | 0.02 | 0.01 |
| z_E_ | z_E_ | 1.00 | 0.00 | 0.00 | 0.00 | 0.00 | 0.00 | 0.00 | 0.00 | 0.00 | 0.00 | 0.00 | 0.00 | 0.00 |
| γ*_b_*_1_ | z*_b_*_1,1_ | 0.89 | 0.00 | 0.00 | 0.00 | 0.00 | 0.01 | 0.01 | 0.01 | 0.01 | 0.01 | 0.01 | 0.01 | 0.01 |
| γ*_b_*_1_ | z*_b_*_1,2_ | 0.89 | 0.00 | 0.00 | 0.00 | 0.00 | 0.01 | 0.01 | 0.01 | 0.01 | 0.01 | 0.01 | 0.01 | 0.01 |
| γ*_b_*_2_ | z*_b_*_2,1_ | 0.92 | 0.00 | 0.00 | 0.00 | 0.00 | 0.01 | 0.01 | 0.01 | 0.00 | 0.01 | 0.01 | 0.01 | 0.00 |
| γ*_b_*_2_ | z*_b_*_2,2_ | 0.92 | 0.00 | 0.00 | 0.00 | 0.00 | 0.01 | 0.01 | 0.01 | 0.00 | 0.01 | 0.01 | 0.01 | 0.00 |
| γ*_b_*_3_ | z*_b_*_3,1_ | 0.95 | 0.00 | 0.00 | 0.00 | 0.00 | 0.01 | 0.01 | 0.00 | 0.00 | 0.01 | 0.01 | 0.00 | 0.00 |
| γ*_b_*_3_ | z*_b_*_3,2_ | 0.95 | 0.00 | 0.00 | 0.00 | 0.00 | 0.01 | 0.01 | 0.00 | 0.00 | 0.01 | 0.01 | 0.00 | 0.00 |
| γ*_b_*_4_ | z*_b_*_4,1_ | 0.87 | 0.00 | 0.00 | 0.00 | 0.00 | 0.02 | 0.01 | 0.01 | 0.01 | 0.02 | 0.01 | 0.01 | 0.01 |
| γ*_b_*_4_ | z*_b_*_4,2_ | 0.87 | 0.00 | 0.00 | 0.00 | 0.00 | 0.02 | 0.01 | 0.01 | 0.01 | 0.02 | 0.01 | 0.01 | 0.01 |
| γ*_b_*_5_ | z*_b_*_5,1_ | 0.89 | 0.00 | 0.00 | 0.00 | 0.00 | 0.01 | 0.01 | 0.01 | 0.01 | 0.01 | 0.01 | 0.01 | 0.01 |
| γ*_b_*_5_ | z*_b_*_5,2_ | 0.89 | 0.00 | 0.00 | 0.00 | 0.00 | 0.01 | 0.01 | 0.01 | 0.01 | 0.01 | 0.01 | 0.01 | 0.01 |
| γ*_b_*_6_ | z*_b_*_6,1_ | 0.92 | 0.00 | 0.00 | 0.00 | 0.00 | 0.01 | 0.01 | 0.01 | 0.00 | 0.01 | 0.01 | 0.01 | 0.00 |
| γ*_b_*_6_ | z*_b_*_6,2_ | 0.92 | 0.00 | 0.00 | 0.00 | 0.00 | 0.01 | 0.01 | 0.01 | 0.00 | 0.01 | 0.01 | 0.01 | 0.00 |
| γ*_b_*_7_ | z*_b_*_7,1_ | 0.95 | 0.00 | 0.00 | 0.00 | 0.00 | 0.01 | 0.01 | 0.00 | 0.00 | 0.01 | 0.01 | 0.00 | 0.00 |
| γ*_b_*_7_ | z*_b_*_7,2_ | 0.95 | 0.00 | 0.00 | 0.00 | 0.00 | 0.01 | 0.01 | 0.00 | 0.00 | 0.01 | 0.01 | 0.00 | 0.00 |
| γ*_b_*_8_ | z*_b_*_8,1_ | 0.87 | 0.00 | 0.00 | 0.00 | 0.00 | 0.02 | 0.01 | 0.01 | 0.01 | 0.02 | 0.01 | 0.01 | 0.01 |
| γ*_b_*_8_ | z*_b_*_8,2_ | 0.87 | 0.00 | 0.00 | 0.00 | 0.00 | 0.02 | 0.01 | 0.01 | 0.01 | 0.02 | 0.01 | 0.01 | 0.01 |
| γ*_b_*_9_ | z*_b_*_9,1_ | 0.89 | 0.00 | 0.00 | 0.00 | 0.00 | 0.01 | 0.01 | 0.01 | 0.01 | 0.01 | 0.01 | 0.01 | 0.01 |
| γ*_b_*_9_ | z*_b_*_9,2_ | 0.89 | 0.00 | 0.00 | 0.00 | 0.00 | 0.01 | 0.01 | 0.01 | 0.01 | 0.01 | 0.01 | 0.01 | 0.01 |
| γ*_b_*_10_ | z*_b_*_10,1_ | 0.92 | 0.00 | 0.00 | 0.00 | 0.00 | 0.01 | 0.01 | 0.01 | 0.00 | 0.01 | 0.01 | 0.01 | 0.00 |
| γ*_b_*_10_ | z*_b_*_10,2_ | 0.92 | 0.00 | 0.00 | 0.00 | 0.00 | 0.01 | 0.01 | 0.01 | 0.00 | 0.01 | 0.01 | 0.01 | 0.00 |
| γ*_b_*_11_ | z*_b_*_11,1_ | 0.95 | 0.00 | 0.00 | 0.00 | 0.00 | 0.01 | 0.01 | 0.00 | 0.00 | 0.01 | 0.01 | 0.00 | 0.00 |
| γ*_b_*_11_ | z*_b_*_11,2_ | 0.95 | 0.00 | 0.00 | 0.00 | 0.00 | 0.01 | 0.01 | 0.00 | 0.00 | 0.01 | 0.01 | 0.00 | 0.00 |
| γ*_b_*_12_ | z*_b_*_12,1_ | 0.87 | 0.00 | 0.00 | 0.00 | 0.00 | 0.02 | 0.01 | 0.01 | 0.01 | 0.02 | 0.01 | 0.01 | 0.01 |
| γ*_b_*_12_ | z*_b_*_12,2_ | 0.87 | 0.00 | 0.00 | 0.00 | 0.00 | 0.02 | 0.01 | 0.01 | 0.01 | 0.02 | 0.01 | 0.01 | 0.01 |
| γ*_b_*_13_ | z*_b_*_13,1_ | 0.89 | 0.00 | 0.00 | 0.00 | 0.00 | 0.02 | 0.01 | 0.01 | 0.01 | 0.02 | 0.01 | 0.01 | 0.01 |
| γ*_b_*_13_ | z*_b_*_13,2_ | 0.89 | 0.00 | 0.00 | 0.00 | 0.00 | 0.02 | 0.01 | 0.01 | 0.01 | 0.02 | 0.01 | 0.01 | 0.01 |
| γ*_b_*_14_ | z*_b_*_14,1_ | 0.92 | 0.00 | 0.00 | 0.00 | 0.00 | 0.01 | 0.01 | 0.01 | 0.00 | 0.01 | 0.01 | 0.01 | 0.00 |
| γ*_b_*_14_ | z*_b_*_14,2_ | 0.92 | 0.00 | 0.00 | 0.00 | 0.00 | 0.01 | 0.01 | 0.01 | 0.00 | 0.01 | 0.01 | 0.01 | 0.00 |
| γ*_b_*_15_ | z*_b_*_15,1_ | 0.95 | 0.00 | 0.00 | 0.00 | 0.00 | 0.01 | 0.01 | 0.00 | 0.00 | 0.01 | 0.01 | 0.00 | 0.00 |
| γ*_b_*_15_ | z*_b_*_15,2_ | 0.95 | 0.00 | 0.00 | 0.00 | 0.00 | 0.01 | 0.01 | 0.00 | 0.00 | 0.01 | 0.01 | 0.00 | 0.00 |
| γ*_b_*_16_ | z*_b_*_16,1_ | 0.87 | 0.00 | 0.00 | 0.00 | 0.00 | 0.02 | 0.01 | 0.01 | 0.01 | 0.02 | 0.01 | 0.01 | 0.01 |
| γ*_b_*_16_ | z*_b_*_16,2_ | 0.87 | 0.00 | 0.00 | 0.00 | 0.00 | 0.02 | 0.01 | 0.01 | 0.01 | 0.02 | 0.01 | 0.01 | 0.01 |
| γ*_b_*_17_ | z*_b_*_17,1_ | 0.89 | 0.00 | 0.00 | 0.00 | 0.00 | 0.01 | 0.01 | 0.01 | 0.01 | 0.01 | 0.01 | 0.01 | 0.01 |
| γ*_b_*_17_ | z*_b_*_17,2_ | 0.89 | 0.00 | 0.00 | 0.00 | 0.00 | 0.01 | 0.01 | 0.01 | 0.01 | 0.01 | 0.01 | 0.01 | 0.01 |
| γ*_b_*_18_ | z*_b_*_18,1_ | 0.92 | 0.00 | 0.00 | 0.00 | 0.00 | 0.01 | 0.01 | 0.01 | 0.00 | 0.01 | 0.01 | 0.01 | 0.00 |
| γ*_b_*_18_ | z*_b_*_18,2_ | 0.92 | 0.00 | 0.00 | 0.00 | 0.00 | 0.01 | 0.01 | 0.01 | 0.00 | 0.01 | 0.01 | 0.01 | 0.00 |
| γ*_b_*_19_ | z*_b_*_19,1_ | 0.95 | 0.00 | 0.00 | 0.00 | 0.00 | 0.01 | 0.01 | 0.00 | 0.00 | 0.01 | 0.01 | 0.00 | 0.00 |
| γ*_b_*_19_ | z*_b_*_19,2_ | 0.95 | 0.00 | 0.00 | 0.00 | 0.00 | 0.01 | 0.01 | 0.00 | 0.00 | 0.01 | 0.01 | 0.00 | 0.00 |
| γ*_b_*_20_ | z*_b_*_20,1_ | 0.87 | 0.00 | 0.00 | 0.00 | 0.00 | 0.02 | 0.01 | 0.01 | 0.01 | 0.02 | 0.01 | 0.01 | 0.01 |
| γ*_b_*_20_ | z*_b_*_20,2_ | 0.87 | 0.00 | 0.00 | 0.00 | 0.00 | 0.02 | 0.01 | 0.01 | 0.01 | 0.02 | 0.01 | 0.01 | 0.01 |
| γ*_b_*_21_ | z*_b_*_21,1_ | 0.89 | 0.00 | 0.00 | 0.00 | 0.00 | 0.01 | 0.01 | 0.01 | 0.01 | 0.01 | 0.01 | 0.01 | 0.01 |
| γ*_b_*_21_ | z*_b_*_21,2_ | 0.89 | 0.00 | 0.00 | 0.00 | 0.00 | 0.01 | 0.01 | 0.01 | 0.01 | 0.01 | 0.01 | 0.01 | 0.01 |
| γ*_b_*_22_ | z*_b_*_22,1_ | 0.92 | 0.00 | 0.00 | 0.00 | 0.00 | 0.01 | 0.01 | 0.01 | 0.00 | 0.01 | 0.01 | 0.01 | 0.00 |
| γ*_b_*_22_ | z*_b_*_22,2_ | 0.92 | 0.00 | 0.00 | 0.00 | 0.00 | 0.01 | 0.01 | 0.01 | 0.00 | 0.01 | 0.01 | 0.01 | 0.00 |
| γ*_b_*_23_ | z*_b_*_23,1_ | 0.95 | 0.00 | 0.00 | 0.00 | 0.00 | 0.01 | 0.01 | 0.00 | 0.00 | 0.01 | 0.01 | 0.00 | 0.00 |
| γ*_b_*_23_ | z*_b_*_23,2_ | 0.95 | 0.00 | 0.00 | 0.00 | 0.00 | 0.01 | 0.01 | 0.00 | 0.00 | 0.01 | 0.01 | 0.00 | 0.00 |
| γ*_b_*_24_ | z*_b_*_24,1_ | 0.87 | 0.00 | 0.00 | 0.00 | 0.00 | 0.02 | 0.01 | 0.01 | 0.01 | 0.02 | 0.01 | 0.01 | 0.01 |
| γ*_b_*_24_ | z*_b_*_24,2_ | 0.87 | 0.00 | 0.00 | 0.00 | 0.00 | 0.02 | 0.01 | 0.01 | 0.01 | 0.02 | 0.01 | 0.01 | 0.01 |
| γ*_b_*_25_ | z*_b_*_25,1_ | 0.89 | 0.00 | 0.00 | 0.00 | 0.00 | 0.01 | 0.01 | 0.01 | 0.01 | 0.01 | 0.01 | 0.01 | 0.01 |
| γ*_b_*_25_ | z*_b_*_25,2_ | 0.89 | 0.00 | 0.00 | 0.00 | 0.00 | 0.01 | 0.01 | 0.01 | 0.01 | 0.01 | 0.01 | 0.01 | 0.01 |
| γ*_b_*_26_ | z*_b_*_26,1_ | 0.92 | 0.00 | 0.00 | 0.00 | 0.00 | 0.01 | 0.01 | 0.01 | 0.00 | 0.01 | 0.01 | 0.01 | 0.00 |
| γ*_b_*_26_ | z*_b_*_26,2_ | 0.92 | 0.00 | 0.00 | 0.00 | 0.00 | 0.01 | 0.01 | 0.01 | 0.00 | 0.01 | 0.01 | 0.01 | 0.00 |
| γ*_b_*_27_ | z*_b_*_27,1_ | 0.95 | 0.00 | 0.00 | 0.00 | 0.00 | 0.01 | 0.01 | 0.00 | 0.00 | 0.01 | 0.01 | 0.00 | 0.00 |
| γ*_b_*_27_ | z*_b_*_27,2_ | 0.95 | 0.00 | 0.00 | 0.00 | 0.00 | 0.01 | 0.01 | 0.00 | 0.00 | 0.01 | 0.01 | 0.00 | 0.00 |
| γ*_b_*_28_ | z*_b_*_28,1_ | 0.87 | 0.00 | 0.00 | 0.00 | 0.00 | 0.02 | 0.01 | 0.01 | 0.01 | 0.02 | 0.01 | 0.01 | 0.01 |
| γ*_b_*_28_ | z*_b_*_28,2_ | 0.87 | 0.00 | 0.00 | 0.00 | 0.00 | 0.02 | 0.01 | 0.01 | 0.01 | 0.02 | 0.01 | 0.01 | 0.01 |
| γ*_b_*_29_ | z*_b_*_29,1_ | 0.89 | 0.00 | 0.00 | 0.00 | 0.00 | 0.01 | 0.01 | 0.01 | 0.01 | 0.01 | 0.01 | 0.01 | 0.01 |
| γ*_b_*_29_ | z*_b_*_29,2_ | 0.89 | 0.00 | 0.00 | 0.00 | 0.00 | 0.01 | 0.01 | 0.01 | 0.01 | 0.01 | 0.01 | 0.01 | 0.01 |
| γ*_b_*_30_ | z*_b_*_30,1_ | 0.92 | 0.00 | 0.00 | 0.00 | 0.00 | 0.01 | 0.01 | 0.01 | 0.00 | 0.01 | 0.01 | 0.01 | 0.00 |
| γ*_b_*_30_ | z*_b_*_30,2_ | 0.92 | 0.00 | 0.00 | 0.00 | 0.00 | 0.01 | 0.01 | 0.01 | 0.00 | 0.01 | 0.01 | 0.01 | 0.00 |
| γ*_b_*_31_ | z*_b_*_31,1_ | 0.95 | 0.00 | 0.00 | 0.00 | 0.00 | 0.01 | 0.01 | 0.00 | 0.00 | 0.01 | 0.01 | 0.00 | 0.00 |
| γ*_b_*_31_ | z*_b_*_31,2_ | 0.95 | 0.00 | 0.00 | 0.00 | 0.00 | 0.01 | 0.01 | 0.00 | 0.00 | 0.01 | 0.01 | 0.00 | 0.00 |
| γ*_b_*_32_ | z*_b_*_32,1_ | 0.87 | 0.00 | 0.00 | 0.00 | 0.00 | 0.02 | 0.01 | 0.01 | 0.01 | 0.02 | 0.01 | 0.01 | 0.01 |
| γ*_b_*_32_ | z*_b_*_32,2_ | 0.87 | 0.00 | 0.00 | 0.00 | 0.00 | 0.02 | 0.01 | 0.01 | 0.01 | 0.02 | 0.01 | 0.01 | 0.01 |
| γ*_b_*_33_ | z*_b_*_33,1_ | 0.89 | 0.00 | 0.00 | 0.00 | 0.00 | 0.01 | 0.01 | 0.01 | 0.01 | 0.01 | 0.01 | 0.01 | 0.01 |
| γ*_b_*_33_ | z*_b_*_33,2_ | 0.89 | 0.00 | 0.00 | 0.00 | 0.00 | 0.01 | 0.01 | 0.01 | 0.01 | 0.01 | 0.01 | 0.01 | 0.01 |
| γ*_b_*_34_ | z*_b_*_34,1_ | 0.92 | 0.00 | 0.00 | 0.00 | 0.00 | 0.01 | 0.01 | 0.01 | 0.00 | 0.01 | 0.01 | 0.01 | 0.00 |
| γ*_b_*_34_ | z*_b_*_34,2_ | 0.92 | 0.00 | 0.00 | 0.00 | 0.00 | 0.01 | 0.01 | 0.01 | 0.00 | 0.01 | 0.01 | 0.01 | 0.00 |
| γ*_b_*_35_ | z*_b_*_35,1_ | 0.95 | 0.00 | 0.00 | 0.00 | 0.00 | 0.01 | 0.01 | 0.00 | 0.00 | 0.01 | 0.01 | 0.00 | 0.00 |
| γ*_b_*_35_ | z*_b_*_35,2_ | 0.95 | 0.00 | 0.00 | 0.00 | 0.00 | 0.01 | 0.01 | 0.00 | 0.00 | 0.01 | 0.01 | 0.00 | 0.00 |
| γ*_b_*_36_ | z*_b_*_36,1_ | 0.87 | 0.00 | 0.00 | 0.00 | 0.00 | 0.02 | 0.01 | 0.01 | 0.01 | 0.02 | 0.01 | 0.01 | 0.01 |
| γ*_b_*_36_ | z*_b_*_36,2_ | 0.87 | 0.00 | 0.00 | 0.00 | 0.00 | 0.02 | 0.01 | 0.01 | 0.01 | 0.02 | 0.01 | 0.01 | 0.01 |
| γ*_b_*_37_ | z*_b_*_37,1_ | 0.89 | 0.00 | 0.00 | 0.00 | 0.00 | 0.01 | 0.01 | 0.01 | 0.01 | 0.01 | 0.01 | 0.01 | 0.01 |
| γ*_b_*_37_ | z*_b_*_37,2_ | 0.89 | 0.00 | 0.00 | 0.00 | 0.00 | 0.01 | 0.01 | 0.01 | 0.01 | 0.01 | 0.01 | 0.01 | 0.01 |
| γ*_b_*_38_ | z*_b_*_38,1_ | 0.92 | 0.00 | 0.00 | 0.00 | 0.00 | 0.01 | 0.01 | 0.01 | 0.00 | 0.01 | 0.01 | 0.01 | 0.00 |
| γ*_b_*_38_ | z*_b_*_38,2_ | 0.92 | 0.00 | 0.00 | 0.00 | 0.00 | 0.01 | 0.01 | 0.01 | 0.00 | 0.01 | 0.01 | 0.01 | 0.00 |
| γ*_b_*_39_ | z*_b_*_39,1_ | 0.95 | 0.00 | 0.00 | 0.00 | 0.00 | 0.01 | 0.01 | 0.00 | 0.00 | 0.01 | 0.01 | 0.00 | 0.00 |
| γ*_b_*_39_ | z*_b_*_39,2_ | 0.95 | 0.00 | 0.00 | 0.00 | 0.00 | 0.01 | 0.01 | 0.00 | 0.00 | 0.01 | 0.01 | 0.00 | 0.00 |
| γ*_b_*_40_ | z*_b_*_40,1_ | 0.87 | 0.00 | 0.00 | 0.00 | 0.00 | 0.02 | 0.01 | 0.01 | 0.01 | 0.02 | 0.01 | 0.01 | 0.01 |
| γ*_b_*_40_ | z*_b_*_40,2_ | 0.87 | 0.00 | 0.00 | 0.00 | 0.00 | 0.02 | 0.01 | 0.01 | 0.01 | 0.02 | 0.01 | 0.01 | 0.01 |
| γ*_b_*_41_ | z*_b_*_41,1_ | 0.89 | 0.00 | 0.00 | 0.00 | 0.00 | 0.01 | 0.01 | 0.01 | 0.01 | 0.01 | 0.01 | 0.01 | 0.01 |
| γ*_b_*_41_ | z*_b_*_41,2_ | 0.89 | 0.00 | 0.00 | 0.00 | 0.00 | 0.01 | 0.01 | 0.01 | 0.01 | 0.01 | 0.01 | 0.01 | 0.01 |
| γ*_b_*_42_ | z*_b_*_42,1_ | 0.92 | 0.00 | 0.00 | 0.00 | 0.00 | 0.01 | 0.01 | 0.01 | 0.00 | 0.01 | 0.01 | 0.01 | 0.00 |
| γ*_b_*_42_ | z*_b_*_42,2_ | 0.92 | 0.00 | 0.00 | 0.00 | 0.00 | 0.01 | 0.01 | 0.01 | 0.00 | 0.01 | 0.01 | 0.01 | 0.00 |
| γ*_b_*_43_ | z*_b_*_43,1_ | 0.95 | 0.00 | 0.00 | 0.00 | 0.00 | 0.01 | 0.01 | 0.00 | 0.00 | 0.01 | 0.01 | 0.00 | 0.00 |
| γ*_b_*_43_ | z*_b_*_43,2_ | 0.95 | 0.00 | 0.00 | 0.00 | 0.00 | 0.01 | 0.01 | 0.00 | 0.00 | 0.01 | 0.01 | 0.00 | 0.00 |
| γ*_b_*_44_ | z*_b_*_44,1_ | 0.87 | 0.00 | 0.00 | 0.00 | 0.00 | 0.02 | 0.01 | 0.01 | 0.01 | 0.02 | 0.01 | 0.01 | 0.01 |
| γ*_b_*_44_ | z*_b_*_44,2_ | 0.87 | 0.00 | 0.00 | 0.00 | 0.00 | 0.02 | 0.01 | 0.01 | 0.01 | 0.02 | 0.01 | 0.01 | 0.01 |
| γ*_b_*_45_ | z*_b_*_45,1_ | 0.89 | 0.00 | 0.00 | 0.00 | 0.00 | 0.01 | 0.01 | 0.01 | 0.01 | 0.01 | 0.01 | 0.01 | 0.01 |
| γ*_b_*_45_ | z*_b_*_45,2_ | 0.89 | 0.00 | 0.00 | 0.00 | 0.00 | 0.01 | 0.01 | 0.01 | 0.01 | 0.01 | 0.01 | 0.01 | 0.01 |
| γ*_b_*_46_ | z*_b_*_46,1_ | 0.92 | 0.00 | 0.00 | 0.00 | 0.00 | 0.01 | 0.01 | 0.01 | 0.00 | 0.01 | 0.01 | 0.01 | 0.00 |
| γ*_b_*_46_ | z*_b_*_46,2_ | 0.92 | 0.00 | 0.00 | 0.00 | 0.00 | 0.01 | 0.01 | 0.01 | 0.00 | 0.01 | 0.01 | 0.01 | 0.00 |
| γ*_b_*_47_ | z*_b_*_47,1_ | 0.95 | 0.00 | 0.00 | 0.00 | 0.00 | 0.01 | 0.01 | 0.00 | 0.00 | 0.01 | 0.01 | 0.00 | 0.00 |
| γ*_b_*_47_ | z*_b_*_47,2_ | 0.95 | 0.00 | 0.00 | 0.00 | 0.00 | 0.01 | 0.01 | 0.00 | 0.00 | 0.01 | 0.01 | 0.00 | 0.00 |
| γ*_b_*_48_ | z*_b_*_48,1_ | 0.87 | 0.00 | 0.00 | 0.00 | 0.00 | 0.02 | 0.01 | 0.01 | 0.01 | 0.02 | 0.01 | 0.01 | 0.01 |
| γ*_b_*_48_ | z*_b_*_48,2_ | 0.87 | 0.00 | 0.00 | 0.00 | 0.00 | 0.02 | 0.01 | 0.01 | 0.01 | 0.02 | 0.01 | 0.01 | 0.01 |
| γ*_b_*_49_ | z*_b_*_49,1_ | 0.89 | 0.00 | 0.00 | 0.00 | 0.00 | 0.01 | 0.01 | 0.01 | 0.01 | 0.01 | 0.01 | 0.01 | 0.01 |
| γ*_b_*_49_ | z*_b_*_49,2_ | 0.89 | 0.00 | 0.00 | 0.00 | 0.00 | 0.01 | 0.01 | 0.01 | 0.01 | 0.01 | 0.01 | 0.01 | 0.01 |
| γ*_b_*_50_ | z*_b_*_50,1_ | 0.92 | 0.00 | 0.00 | 0.00 | 0.00 | 0.01 | 0.01 | 0.01 | 0.00 | 0.01 | 0.01 | 0.01 | 0.00 |
| γ*_b_*_50_ | z*_b_*_50,2_ | 0.92 | 0.00 | 0.00 | 0.00 | 0.00 | 0.01 | 0.01 | 0.01 | 0.00 | 0.01 | 0.01 | 0.01 | 0.00 |
| γ*_b_*_51_ | z*_b_*_51,1_ | 0.95 | 0.00 | 0.00 | 0.00 | 0.00 | 0.01 | 0.01 | 0.00 | 0.00 | 0.01 | 0.01 | 0.00 | 0.00 |
| γ*_b_*_51_ | z*_b_*_51,2_ | 0.95 | 0.00 | 0.00 | 0.00 | 0.00 | 0.01 | 0.01 | 0.00 | 0.00 | 0.01 | 0.01 | 0.00 | 0.00 |
| γ*_b_*_52_ | z*_b_*_52,1_ | 0.87 | 0.00 | 0.00 | 0.00 | 0.00 | 0.02 | 0.01 | 0.01 | 0.01 | 0.02 | 0.01 | 0.01 | 0.01 |
| γ*_b_*_52_ | z*_b_*_52,2_ | 0.87 | 0.00 | 0.00 | 0.00 | 0.00 | 0.02 | 0.01 | 0.01 | 0.01 | 0.02 | 0.01 | 0.01 | 0.01 |
| γ*_b_*_53_ | z*_b_*_53,1_ | 0.89 | 0.00 | 0.00 | 0.00 | 0.00 | 0.01 | 0.01 | 0.01 | 0.01 | 0.02 | 0.01 | 0.01 | 0.01 |
| γ*_b_*_53_ | z*_b_*_53,2_ | 0.89 | 0.00 | 0.00 | 0.00 | 0.00 | 0.01 | 0.01 | 0.01 | 0.01 | 0.01 | 0.01 | 0.01 | 0.01 |
| γ*_b_*_54_ | z*_b_*_54,1_ | 0.92 | 0.00 | 0.00 | 0.00 | 0.00 | 0.01 | 0.01 | 0.01 | 0.00 | 0.01 | 0.01 | 0.01 | 0.00 |
| γ*_b_*_54_ | z*_b_*_54,2_ | 0.92 | 0.00 | 0.00 | 0.00 | 0.00 | 0.01 | 0.01 | 0.01 | 0.00 | 0.01 | 0.01 | 0.01 | 0.00 |
| γ*_b_*_55_ | z*_b_*_55,1_ | 0.95 | 0.00 | 0.00 | 0.00 | 0.00 | 0.01 | 0.01 | 0.00 | 0.00 | 0.01 | 0.01 | 0.00 | 0.00 |
| γ*_b_*_55_ | z*_b_*_55,2_ | 0.95 | 0.00 | 0.00 | 0.00 | 0.00 | 0.01 | 0.01 | 0.00 | 0.00 | 0.01 | 0.01 | 0.00 | 0.00 |
| γ*_b_*_56_ | z*_b_*_56,1_ | 0.87 | 0.00 | 0.00 | 0.00 | 0.00 | 0.02 | 0.01 | 0.01 | 0.01 | 0.02 | 0.01 | 0.01 | 0.01 |
| γ*_b_*_56_ | z*_b_*_56,2_ | 0.87 | 0.00 | 0.00 | 0.00 | 0.00 | 0.02 | 0.01 | 0.01 | 0.01 | 0.02 | 0.01 | 0.01 | 0.01 |
| γ*_b_*_57_ | z*_b_*_57,1_ | 0.89 | 0.00 | 0.00 | 0.00 | 0.00 | 0.01 | 0.01 | 0.01 | 0.01 | 0.01 | 0.01 | 0.01 | 0.01 |
| γ*_b_*_57_ | z*_b_*_57,2_ | 0.89 | 0.00 | 0.00 | 0.00 | 0.00 | 0.01 | 0.01 | 0.01 | 0.01 | 0.01 | 0.01 | 0.01 | 0.01 |
| γ*_b_*_58_ | z*_b_*_58,1_ | 0.92 | 0.00 | 0.00 | 0.00 | 0.00 | 0.01 | 0.01 | 0.01 | 0.00 | 0.01 | 0.01 | 0.01 | 0.00 |
| γ*_b_*_58_ | z*_b_*_58,2_ | 0.92 | 0.00 | 0.00 | 0.00 | 0.00 | 0.01 | 0.01 | 0.01 | 0.00 | 0.01 | 0.01 | 0.01 | 0.00 |
| γ*_b_*_59_ | z*_b_*_59,1_ | 0.95 | 0.00 | 0.00 | 0.00 | 0.00 | 0.01 | 0.01 | 0.00 | 0.00 | 0.01 | 0.01 | 0.00 | 0.00 |
| γ*_b_*_59_ | z*_b_*_59,2_ | 0.95 | 0.00 | 0.00 | 0.00 | 0.00 | 0.01 | 0.01 | 0.00 | 0.00 | 0.01 | 0.01 | 0.00 | 0.00 |
| γ*_b_*_60_ | z*_b_*_60,1_ | 0.87 | 0.00 | 0.00 | 0.00 | 0.00 | 0.02 | 0.01 | 0.01 | 0.01 | 0.02 | 0.01 | 0.01 | 0.01 |
| γ*_b_*_60_ | z*_b_*_60,2_ | 0.87 | 0.00 | 0.00 | 0.00 | 0.00 | 0.02 | 0.01 | 0.01 | 0.01 | 0.02 | 0.01 | 0.01 | 0.01 |
| z_O_ | z_O_ | 1.00 | 0.00 | 0.00 | 0.00 | 0.00 | 0.00 | 0.00 | 0.00 | 0.00 | 0.00 | 0.00 | 0.00 | 0.00 |

S2 Table 2. Biases, standard deviations (SD), and root mean square errors (RMSE) of path coefficients estimated from IG-GSCA over different sample sizes in the simulation study.

|  |  |  | Bias | | | | SD | | | | RMSE | | | |
| --- | --- | --- | --- | --- | --- | --- | --- | --- | --- | --- | --- | --- | --- | --- |
|  |  | True value | N=250 | N=500 | N=1000 | N=2000 | N=250 | N=500 | N=1000 | N=2000 | N=250 | N=500 | N=1000 | N=2000 |
| γ*_g_*_1_ | γ*_b_*_1_ | 0.6 | -0.04 | -0.02 | -0.01 | -0.01 | 0.03 | 0.02 | 0.04 | 0.01 | 0.05 | 0.03 | 0.04 | 0.01 |
| γ*_g_*_2_ | γ*_b_*_1_ | 0.4 | -0.02 | -0.01 | 0.00 | 0.00 | 0.03 | 0.02 | 0.03 | 0.01 | 0.04 | 0.02 | 0.03 | 0.01 |
| γ*_g_*_3_ | γ*_b_*_1_ | 0 | 0.01 | 0.01 | 0.00 | 0.00 | 0.03 | 0.02 | 0.01 | 0.01 | 0.03 | 0.02 | 0.01 | 0.01 |
| γ*_g_*_4_ | γ*_b_*_1_ | 0 | 0.00 | 0.00 | 0.00 | 0.00 | 0.03 | 0.02 | 0.01 | 0.01 | 0.03 | 0.02 | 0.01 | 0.01 |
| γ*_g_*_5_ | γ*_b_*_1_ | 0 | 0.00 | 0.00 | 0.00 | 0.00 | 0.03 | 0.02 | 0.01 | 0.01 | 0.03 | 0.02 | 0.01 | 0.01 |
| γ*_g_*_6_ | γ*_b_*_1_ | 0 | 0.00 | 0.00 | 0.00 | 0.00 | 0.03 | 0.02 | 0.01 | 0.01 | 0.03 | 0.02 | 0.01 | 0.01 |
| γ*_g_*_7_ | γ*_b_*_1_ | 0 | 0.00 | 0.00 | 0.00 | 0.00 | 0.03 | 0.02 | 0.01 | 0.01 | 0.03 | 0.02 | 0.01 | 0.01 |
| γ*_g_*_8_ | γ*_b_*_1_ | 0 | 0.00 | 0.00 | 0.00 | 0.00 | 0.03 | 0.02 | 0.01 | 0.01 | 0.03 | 0.02 | 0.01 | 0.01 |
| γ*_g_*_9_ | γ*_b_*_1_ | 0 |  | 0.00 | 0.00 | 0.00 | 0.03 | 0.02 | 0.01 | 0.01 | 0.03 | 0.02 | 0.01 | 0.01 |
| z_E_ | γ*_b_*_1_ | 0.2 | -0.02 | -0.01 | -0.01 | 0.00 | 0.03 | 0.02 | 0.02 | 0.01 | 0.04 | 0.03 | 0.02 | 0.01 |
| γ*_g_*_1×E_ | γ*_b_*_1_ | -0.4 | 0.03 | 0.01 | 0.01 | 0.00 | 0.03 | 0.02 | 0.03 | 0.01 | 0.04 | 0.03 | 0.03 | 0.01 |
| γ*_g_*_2×E_ | γ*_b_*_1_ | 0 | -0.01 | 0.00 | 0.00 | 0.00 | 0.03 | 0.02 | 0.01 | 0.01 | 0.03 | 0.02 | 0.01 | 0.01 |
| γ*_g_*_3×E_ | γ*_b_*_1_ | 0 | -0.01 | 0.00 | 0.00 | 0.00 | 0.03 | 0.02 | 0.01 | 0.01 | 0.03 | 0.02 | 0.01 | 0.01 |
| γ*_g_*_4×E_ | γ*_b_*_1_ | 0 | 0.00 | 0.00 | 0.00 | 0.00 | 0.03 | 0.02 | 0.01 | 0.01 | 0.03 | 0.02 | 0.01 | 0.01 |
| γ*_g_*_5×E_ | γ*_b_*_1_ | 0 | 0.00 | 0.00 | 0.00 | 0.00 | 0.03 | 0.02 | 0.01 | 0.01 | 0.03 | 0.02 | 0.01 | 0.01 |
| γ*_g_*_6×E_ | γ*_b_*_1_ | 0 | 0.00 | 0.00 | 0.00 | 0.00 | 0.03 | 0.02 | 0.01 | 0.01 | 0.03 | 0.02 | 0.01 | 0.01 |
| γ*_g_*_7×E_ | γ*_b_*_1_ | 0 | 0.00 | 0.00 | 0.00 | 0.00 | 0.03 | 0.02 | 0.01 | 0.01 | 0.03 | 0.02 | 0.01 | 0.01 |
| γ*_g_*_8×E_ | γ*_b_*_1_ | 0 | 0.00 | 0.00 | 0.00 | 0.00 | 0.03 | 0.02 | 0.01 | 0.01 | 0.03 | 0.02 | 0.01 | 0.01 |
| γ*_g_*_9×E_ | γ*_b_*_1_ | 0 | 0.00 | 0.00 | 0.00 | 0.00 | 0.03 | 0.02 | 0.01 | 0.01 | 0.03 | 0.02 | 0.01 | 0.01 |
| γ*_g_*_1_ | γ*_b_*_2_ | 0 | -0.01 | 0.00 | 0.00 | 0.00 | 0.05 | 0.03 | 0.02 | 0.02 | 0.05 | 0.03 | 0.02 | 0.02 |
| γ*_g_*_2_ | γ*_b_*_2_ | -0.6 | 0.05 | 0.02 | 0.01 | 0.01 | 0.04 | 0.05 | 0.04 | 0.04 | 0.06 | 0.05 | 0.05 | 0.04 |
| γ*_g_*_3_ | γ*_b_*_2_ | 0.4 | -0.04 | -0.02 | -0.01 | -0.01 | 0.05 | 0.04 | 0.04 | 0.03 | 0.06 | 0.05 | 0.04 | 0.03 |
| γ*_g_*_4_ | γ*_b_*_2_ | 0 | 0.01 | 0.00 | 0.00 | 0.00 | 0.04 | 0.03 | 0.02 | 0.02 | 0.04 | 0.03 | 0.02 | 0.02 |
| γ*_g_*_5_ | γ*_b_*_2_ | 0 | 0.00 | 0.00 | 0.00 | 0.00 | 0.04 | 0.03 | 0.02 | 0.02 | 0.04 | 0.03 | 0.02 | 0.02 |
| γ*_g_*_6_ | γ*_b_*_2_ | 0 | 0.00 | 0.00 | 0.00 | 0.00 | 0.05 | 0.03 | 0.02 | 0.02 | 0.05 | 0.03 | 0.02 | 0.02 |
| γ*_g_*_7_ | γ*_b_*_2_ | 0 | 0.00 | 0.00 | 0.00 | 0.00 | 0.04 | 0.03 | 0.02 | 0.02 | 0.04 | 0.03 | 0.02 | 0.02 |
| γ*_g_*_8_ | γ*_b_*_2_ | 0 | 0.00 | 0.00 | 0.00 | 0.00 | 0.05 | 0.03 | 0.02 | 0.02 | 0.05 | 0.03 | 0.02 | 0.02 |
| γ*_g_*_9_ | γ*_b_*_2_ | 0 | 0.00 | 0.00 | 0.00 | 0.00 | 0.05 | 0.03 | 0.02 | 0.02 | 0.05 | 0.03 | 0.02 | 0.02 |
| z_E_ | γ*_b_*_2_ | 0 | 0.00 | 0.00 | 0.00 | 0.00 | 0.05 | 0.04 | 0.02 | 0.02 | 0.05 | 0.04 | 0.02 | 0.02 |
| γ*_g_*_1×E_ | γ*_b_*_2_ | 0 | 0.00 | 0.00 | 0.00 | 0.00 | 0.05 | 0.03 | 0.02 | 0.02 | 0.05 | 0.03 | 0.02 | 0.02 |
| γ*_g_*_2×E_ | γ*_b_*_2_ | 0.2 | -0.02 | -0.01 | -0.01 | 0.00 | 0.05 | 0.04 | 0.03 | 0.02 | 0.05 | 0.04 | 0.03 | 0.02 |
| γ*_g_*_3×E_ | γ*_b_*_2_ | -0.4 | 0.03 | 0.02 | 0.01 | 0.00 | 0.05 | 0.04 | 0.04 | 0.03 | 0.06 | 0.05 | 0.04 | 0.03 |
| γ*_g_*_4×E_ | γ*_b_*_2_ | 0 | -0.01 | 0.00 | -0.01 | 0.00 | 0.05 | 0.03 | 0.02 | 0.02 | 0.05 | 0.03 | 0.03 | 0.02 |
| γ*_g_*_5×E_ | γ*_b_*_2_ | 0 | 0.00 | 0.00 | 0.00 | 0.00 | 0.05 | 0.03 | 0.02 | 0.02 | 0.05 | 0.03 | 0.02 | 0.02 |
| γ*_g_*_6×E_ | γ*_b_*_2_ | 0 | 0.00 | 0.00 | 0.00 | 0.00 | 0.05 | 0.03 | 0.02 | 0.02 | 0.05 | 0.03 | 0.02 | 0.02 |
| γ*_g_*_7×E_ | γ*_b_*_2_ | 0 | 0.00 | 0.00 | 0.00 | 0.00 | 0.05 | 0.03 | 0.02 | 0.02 | 0.05 | 0.03 | 0.02 | 0.02 |
| γ*_g_*_8×E_ | γ*_b_*_2_ | 0 | 0.00 | 0.00 | 0.00 | 0.00 | 0.05 | 0.04 | 0.02 | 0.02 | 0.05 | 0.04 | 0.02 | 0.02 |
| γ*_g_*_9×E_ | γ*_b_*_2_ | 0 | 0.00 | 0.00 | 0.00 | 0.00 | 0.05 | 0.03 | 0.02 | 0.02 | 0.05 | 0.03 | 0.02 | 0.02 |
| γ*_g_*_1_ | γ*_b_*_3_ | 0.4 | -0.04 | -0.02 | -0.01 | -0.01 | 0.05 | 0.04 | 0.03 | 0.03 | 0.06 | 0.04 | 0.03 | 0.03 |
| γ*_g_*_2_ | γ*_b_*_3_ | 0 | 0.00 | 0.00 | 0.00 | 0.00 | 0.05 | 0.04 | 0.03 | 0.02 | 0.05 | 0.04 | 0.03 | 0.02 |
| γ*_g_*_3_ | γ*_b_*_3_ | -0.6 | 0.05 | 0.02 | 0.01 | 0.01 | 0.04 | 0.03 | 0.02 | 0.04 | 0.06 | 0.04 | 0.02 | 0.04 |
| γ*_g_*_4_ | γ*_b_*_3_ | 0 | 0.00 | 0.00 | 0.00 | 0.00 | 0.05 | 0.04 | 0.03 | 0.02 | 0.05 | 0.04 | 0.03 | 0.02 |
| γ*_g_*_5_ | γ*_b_*_3_ | 0 | 0.00 | 0.00 | 0.00 | 0.00 | 0.05 | 0.04 | 0.03 | 0.02 | 0.05 | 0.04 | 0.03 | 0.02 |
| γ*_g_*_6_ | γ*_b_*_3_ | 0 | 0.01 | 0.01 | 0.01 | 0.00 | 0.05 | 0.04 | 0.03 | 0.02 | 0.05 | 0.04 | 0.03 | 0.02 |
| γ*_g_*_7_ | γ*_b_*_3_ | 0 | 0.00 | 0.00 | 0.00 | 0.00 | 0.05 | 0.04 | 0.03 | 0.02 | 0.05 | 0.04 | 0.03 | 0.02 |
| γ*_g_*_8_ | γ*_b_*_3_ | 0 | 0.00 | 0.00 | 0.00 | 0.00 | 0.05 | 0.04 | 0.03 | 0.02 | 0.05 | 0.04 | 0.03 | 0.02 |
| γ*_g_*_9_ | γ*_b_*_3_ | 0 | 0.00 | 0.00 | 0.00 | 0.00 | 0.05 | 0.04 | 0.03 | 0.02 | 0.05 | 0.04 | 0.03 | 0.02 |
| z_E_ | γ*_b_*_3_ | 0 | 0.00 | 0.00 | 0.00 | 0.00 | 0.05 | 0.03 | 0.03 | 0.02 | 0.05 | 0.03 | 0.03 | 0.02 |
| γ*_g_*_1×E_ | γ*_b_*_3_ | 0.2 | -0.02 | -0.01 | -0.01 | -0.01 | 0.05 | 0.04 | 0.03 | 0.02 | 0.05 | 0.04 | 0.03 | 0.02 |
| γ*_g_*_2×E_ | γ*_b_*_3_ | 0 | 0.01 | 0.00 | 0.00 | 0.00 | 0.05 | 0.04 | 0.03 | 0.02 | 0.05 | 0.04 | 0.03 | 0.02 |
| γ*_g_*_3×E_ | γ*_b_*_3_ | 0 | 0.00 | 0.00 | 0.00 | 0.00 | 0.05 | 0.04 | 0.03 | 0.02 | 0.05 | 0.04 | 0.03 | 0.02 |
| γ*_g_*_4×E_ | γ*_b_*_3_ | 0 | 0.01 | 0.01 | 0.01 | 0.01 | 0.05 | 0.04 | 0.03 | 0.02 | 0.05 | 0.04 | 0.03 | 0.02 |
| γ*_g_*_5×E_ | γ*_b_*_3_ | 0 | 0.00 | 0.00 | 0.00 | 0.00 | 0.05 | 0.04 | 0.03 | 0.02 | 0.05 | 0.04 | 0.03 | 0.02 |
| γ*_g_*_6×E_ | γ*_b_*_3_ | 0 | 0.00 | 0.00 | 0.00 | 0.00 | 0.05 | 0.04 | 0.03 | 0.02 | 0.05 | 0.04 | 0.03 | 0.02 |
| γ*_g_*_7×E_ | γ*_b_*_3_ | 0 | 0.00 | 0.00 | 0.00 | 0.00 | 0.05 | 0.04 | 0.03 | 0.02 | 0.05 | 0.04 | 0.03 | 0.02 |
| γ*_g_*_8×E_ | γ*_b_*_3_ | 0 | 0.00 | 0.01 | 0.00 | 0.00 | 0.05 | 0.04 | 0.03 | 0.02 | 0.05 | 0.04 | 0.03 | 0.02 |
| γ*_g_*_9×E_ | γ*_b_*_3_ | 0 | 0.00 | 0.00 | 0.00 | 0.00 | 0.05 | 0.04 | 0.03 | 0.02 | 0.05 | 0.04 | 0.03 | 0.02 |
| γ*_g_*_1_ | γ*_b_*_4_ | -0.6 | 0.04 | 0.02 | 0.01 | 0.00 | 0.04 | 0.05 | 0.04 | 0.04 | 0.06 | 0.05 | 0.04 | 0.04 |
| γ*_g_*_2_ | γ*_b_*_4_ | 0 | -0.01 | -0.01 | 0.00 | 0.00 | 0.04 | 0.03 | 0.02 | 0.02 | 0.04 | 0.03 | 0.02 | 0.02 |
| γ*_g_*_3_ | γ*_b_*_4_ | 0 | -0.01 | 0.00 | 0.00 | 0.00 | 0.04 | 0.03 | 0.02 | 0.02 | 0.05 | 0.03 | 0.02 | 0.02 |
| γ*_g_*_4_ | γ*_b_*_4_ | 0 | 0.00 | 0.00 | 0.00 | 0.00 | 0.04 | 0.03 | 0.02 | 0.02 | 0.04 | 0.03 | 0.02 | 0.02 |
| γ*_g_*_5_ | γ*_b_*_4_ | 0 | 0.00 | 0.00 | 0.00 | 0.00 | 0.04 | 0.03 | 0.02 | 0.02 | 0.04 | 0.03 | 0.02 | 0.02 |
| γ*_g_*_6_ | γ*_b_*_4_ | 0 | 0.00 | 0.00 | 0.00 | 0.00 | 0.04 | 0.03 | 0.02 | 0.02 | 0.04 | 0.03 | 0.02 | 0.02 |
| γ*_g_*_7_ | γ*_b_*_4_ | 0 | 0.00 | 0.00 | 0.00 | 0.00 | 0.04 | 0.03 | 0.02 | 0.02 | 0.04 | 0.03 | 0.02 | 0.02 |
| γ*_g_*_8_ | γ*_b_*_4_ | 0 | 0.00 | 0.00 | 0.00 | 0.00 | 0.04 | 0.03 | 0.02 | 0.02 | 0.04 | 0.03 | 0.02 | 0.02 |
| γ*_g_*_9_ | γ*_b_*_4_ | 0 | -0.01 | -0.01 | -0.01 | -0.01 | 0.04 | 0.03 | 0.02 | 0.02 | 0.04 | 0.03 | 0.02 | 0.02 |
| z_E_ | γ*_b_*_4_ | 0.2 | -0.02 | -0.01 | -0.01 | -0.01 | 0.05 | 0.04 | 0.03 | 0.02 | 0.05 | 0.04 | 0.03 | 0.02 |
| γ*_g_*_1×E_ | γ*_b_*_4_ | 0.4 | -0.03 | -0.02 | -0.01 | -0.01 | 0.05 | 0.04 | 0.03 | 0.03 | 0.05 | 0.04 | 0.04 | 0.03 |
| γ*_g_*_2×E_ | γ*_b_*_4_ | 0 | 0.01 | 0.00 | 0.00 | 0.00 | 0.05 | 0.03 | 0.02 | 0.02 | 0.05 | 0.03 | 0.02 | 0.02 |
| γ*_g_*_3×E_ | γ*_b_*_4_ | 0 | 0.00 | 0.00 | 0.00 | 0.00 | 0.05 | 0.03 | 0.02 | 0.02 | 0.05 | 0.03 | 0.02 | 0.02 |
| γ*_g_*_4×E_ | γ*_b_*_4_ | 0 | 0.00 | 0.00 | 0.00 | 0.00 | 0.04 | 0.03 | 0.02 | 0.02 | 0.04 | 0.03 | 0.02 | 0.02 |
| γ*_g_*_5×E_ | γ*_b_*_4_ | 0 | 0.01 | 0.01 | 0.01 | 0.01 | 0.05 | 0.03 | 0.02 | 0.02 | 0.05 | 0.03 | 0.02 | 0.02 |
| γ*_g_*_6×E_ | γ*_b_*_4_ | 0 | 0.00 | 0.00 | 0.00 | 0.00 | 0.05 | 0.03 | 0.02 | 0.02 | 0.05 | 0.03 | 0.02 | 0.02 |
| γ*_g_*_7×E_ | γ*_b_*_4_ | 0 | 0.00 | 0.00 | 0.00 | 0.00 | 0.05 | 0.03 | 0.02 | 0.02 | 0.05 | 0.03 | 0.02 | 0.02 |
| γ*_g_*_8×E_ | γ*_b_*_4_ | 0 | 0.00 | 0.00 | 0.00 | 0.00 | 0.04 | 0.03 | 0.02 | 0.02 | 0.04 | 0.03 | 0.02 | 0.02 |
| γ*_g_*_9×E_ | γ*_b_*_4_ | 0 | 0.00 | 0.00 | 0.00 | 0.00 | 0.05 | 0.03 | 0.02 | 0.02 | 0.05 | 0.03 | 0.02 | 0.02 |
| γ*_g_*_1_ | γ*_b_*_5_ | 0.6 | -0.04 | -0.02 | -0.01 | 0.00 | 0.03 | 0.02 | 0.02 | 0.01 | 0.05 | 0.03 | 0.02 | 0.01 |
| γ*_g_*_2_ | γ*_b_*_5_ | 0.4 | -0.02 | -0.01 | 0.00 | 0.00 | 0.03 | 0.02 | 0.02 | 0.01 | 0.03 | 0.02 | 0.02 | 0.01 |
| γ*_g_*_3_ | γ*_b_*_5_ | 0 | 0.02 | 0.01 | 0.00 | 0.00 | 0.03 | 0.02 | 0.01 | 0.01 | 0.03 | 0.02 | 0.01 | 0.01 |
| γ*_g_*_4_ | γ*_b_*_5_ | 0 | 0.00 | 0.00 | 0.00 | 0.00 | 0.03 | 0.02 | 0.01 | 0.01 | 0.03 | 0.02 | 0.01 | 0.01 |
| γ*_g_*_5_ | γ*_b_*_5_ | 0 | 0.00 | 0.00 | 0.00 | 0.00 | 0.03 | 0.02 | 0.01 | 0.01 | 0.03 | 0.02 | 0.01 | 0.01 |
| γ*_g_*_6_ | γ*_b_*_5_ | 0 | 0.00 | 0.00 | 0.00 | 0.00 | 0.03 | 0.02 | 0.01 | 0.01 | 0.03 | 0.02 | 0.01 | 0.01 |
| γ*_g_*_7_ | γ*_b_*_5_ | 0 | 0.00 | 0.00 | 0.00 | 0.00 | 0.03 | 0.02 | 0.01 | 0.01 | 0.03 | 0.02 | 0.01 | 0.01 |
| γ*_g_*_8_ | γ*_b_*_5_ | 0 | 0.00 | 0.00 | 0.00 | 0.00 | 0.03 | 0.02 | 0.01 | 0.01 | 0.03 | 0.02 | 0.01 | 0.01 |
| γ*_g_*_9_ | γ*_b_*_5_ | 0 | 0.00 | 0.00 | 0.00 | 0.00 | 0.03 | 0.02 | 0.01 | 0.01 | 0.03 | 0.02 | 0.01 | 0.01 |
| z_E_ | γ*_b_*_5_ | 0.2 | -0.02 | -0.01 | -0.01 | 0.00 | 0.03 | 0.02 | 0.02 | 0.01 | 0.04 | 0.03 | 0.02 | 0.01 |
| γ*_g_*_1×E_ | γ*_b_*_5_ | -0.4 | 0.03 | 0.02 | 0.01 | 0.01 | 0.03 | 0.02 | 0.02 | 0.01 | 0.04 | 0.03 | 0.02 | 0.01 |
| γ*_g_*_2×E_ | γ*_b_*_5_ | 0 | -0.01 | 0.00 | 0.00 | 0.00 | 0.03 | 0.02 | 0.01 | 0.01 | 0.03 | 0.02 | 0.01 | 0.01 |
| γ*_g_*_3×E_ | γ*_b_*_5_ | 0 | -0.01 | 0.00 | 0.00 | 0.00 | 0.03 | 0.02 | 0.01 | 0.01 | 0.03 | 0.02 | 0.01 | 0.01 |
| γ*_g_*_4×E_ | γ*_b_*_5_ | 0 | 0.00 | 0.00 | 0.00 | 0.00 | 0.03 | 0.02 | 0.01 | 0.01 | 0.03 | 0.02 | 0.01 | 0.01 |
| γ*_g_*_5×E_ | γ*_b_*_5_ | 0 | 0.00 | 0.00 | 0.00 | 0.00 | 0.03 | 0.02 | 0.01 | 0.01 | 0.03 | 0.02 | 0.01 | 0.01 |
| γ*_g_*_6×E_ | γ*_b_*_5_ | 0 | 0.00 | 0.00 | 0.00 | 0.00 | 0.03 | 0.02 | 0.01 | 0.01 | 0.03 | 0.02 | 0.01 | 0.01 |
| γ*_g_*_7×E_ | γ*_b_*_5_ | 0 | 0.00 | 0.00 | 0.00 | 0.00 | 0.03 | 0.02 | 0.01 | 0.01 | 0.03 | 0.02 | 0.01 | 0.01 |
| γ*_g_*_8×E_ | γ*_b_*_5_ | 0 | 0.00 | 0.00 | 0.00 | 0.00 | 0.03 | 0.02 | 0.01 | 0.01 | 0.03 | 0.02 | 0.01 | 0.01 |
| γ*_g_*_9×E_ | γ*_b_*_5_ | 0 | 0.00 | 0.00 | 0.00 | 0.00 | 0.03 | 0.02 | 0.01 | 0.01 | 0.03 | 0.02 | 0.01 | 0.01 |
| γ*_g_*_1_ | γ*_b_*_6_ | 0 | 0.00 | 0.00 | 0.00 | 0.00 | 0.05 | 0.03 | 0.02 | 0.02 | 0.05 | 0.03 | 0.02 | 0.02 |
| γ*_g_*_2_ | γ*_b_*_6_ | -0.6 | 0.05 | 0.02 | 0.01 | 0.00 | 0.07 | 0.05 | 0.02 | 0.02 | 0.09 | 0.05 | 0.02 | 0.02 |
| γ*_g_*_3_ | γ*_b_*_6_ | 0.4 | -0.04 | -0.02 | -0.01 | -0.01 | 0.06 | 0.04 | 0.03 | 0.02 | 0.07 | 0.05 | 0.03 | 0.02 |
| γ*_g_*_4_ | γ*_b_*_6_ | 0 | 0.00 | 0.00 | -0.01 | 0.00 | 0.05 | 0.03 | 0.02 | 0.02 | 0.05 | 0.03 | 0.02 | 0.02 |
| γ*_g_*_5_ | γ*_b_*_6_ | 0 | 0.00 | 0.00 | 0.00 | 0.00 | 0.05 | 0.03 | 0.03 | 0.02 | 0.05 | 0.03 | 0.03 | 0.02 |
| γ*_g_*_6_ | γ*_b_*_6_ | 0 | 0.00 | 0.00 | 0.00 | 0.00 | 0.05 | 0.03 | 0.02 | 0.02 | 0.05 | 0.03 | 0.02 | 0.02 |
| γ*_g_*_7_ | γ*_b_*_6_ | 0 | 0.00 | 0.00 | 0.00 | 0.00 | 0.04 | 0.03 | 0.02 | 0.02 | 0.04 | 0.03 | 0.02 | 0.02 |
| γ*_g_*_8_ | γ*_b_*_6_ | 0 | 0.00 | 0.00 | 0.00 | 0.00 | 0.05 | 0.03 | 0.02 | 0.02 | 0.05 | 0.03 | 0.02 | 0.02 |
| γ*_g_*_9_ | γ*_b_*_6_ | 0 | 0.00 | 0.00 | 0.00 | 0.00 | 0.05 | 0.04 | 0.02 | 0.02 | 0.05 | 0.04 | 0.02 | 0.02 |
| z_E_ | γ*_b_*_6_ | 0 | 0.00 | 0.00 | 0.00 | 0.00 | 0.05 | 0.04 | 0.02 | 0.02 | 0.05 | 0.04 | 0.03 | 0.02 |
| γ*_g_*_1×E_ | γ*_b_*_6_ | 0 | 0.00 | 0.00 | 0.00 | 0.01 | 0.05 | 0.04 | 0.02 | 0.02 | 0.05 | 0.04 | 0.02 | 0.02 |
| γ*_g_*_2×E_ | γ*_b_*_6_ | 0.2 | -0.03 | -0.01 | -0.01 | 0.00 | 0.05 | 0.04 | 0.02 | 0.02 | 0.06 | 0.04 | 0.02 | 0.02 |
| γ*_g_*_3×E_ | γ*_b_*_6_ | -0.4 | 0.04 | 0.02 | 0.01 | 0.01 | 0.06 | 0.04 | 0.03 | 0.02 | 0.07 | 0.05 | 0.03 | 0.02 |
| γ*_g_*_4×E_ | γ*_b_*_6_ | 0 | 0.00 | 0.00 | 0.00 | 0.00 | 0.05 | 0.03 | 0.02 | 0.02 | 0.05 | 0.03 | 0.02 | 0.02 |
| γ*_g_*_5×E_ | γ*_b_*_6_ | 0 | 0.00 | 0.00 | 0.00 | 0.00 | 0.05 | 0.03 | 0.02 | 0.02 | 0.05 | 0.03 | 0.02 | 0.02 |
| γ*_g_*_6×E_ | γ*_b_*_6_ | 0 | 0.00 | 0.00 | 0.00 | 0.00 | 0.05 | 0.03 | 0.02 | 0.02 | 0.05 | 0.03 | 0.02 | 0.02 |
| γ*_g_*_7×E_ | γ*_b_*_6_ | 0 | 0.00 | 0.00 | 0.00 | 0.00 | 0.05 | 0.03 | 0.02 | 0.02 | 0.05 | 0.03 | 0.02 | 0.02 |
| γ*_g_*_8×E_ | γ*_b_*_6_ | 0 | 0.00 | 0.00 | 0.00 | 0.00 | 0.05 | 0.03 | 0.02 | 0.02 | 0.05 | 0.03 | 0.02 | 0.02 |
| γ*_g_*_9×E_ | γ*_b_*_6_ | 0 | 0.00 | 0.00 | 0.00 | 0.00 | 0.05 | 0.03 | 0.02 | 0.02 | 0.05 | 0.03 | 0.02 | 0.02 |
| γ*_g_*_1_ | γ*_b_*_7_ | 0.4 | -0.04 | -0.02 | -0.01 | -0.01 | 0.05 | 0.04 | 0.02 | 0.02 | 0.06 | 0.04 | 0.03 | 0.02 |
| γ*_g_*_2_ | γ*_b_*_7_ | 0 | -0.01 | 0.00 | 0.00 | 0.00 | 0.05 | 0.04 | 0.03 | 0.02 | 0.05 | 0.04 | 0.03 | 0.02 |
| γ*_g_*_3_ | γ*_b_*_7_ | -0.6 | 0.05 | 0.03 | 0.02 | 0.01 | 0.04 | 0.03 | 0.02 | 0.02 | 0.07 | 0.04 | 0.03 | 0.02 |
| γ*_g_*_4_ | γ*_b_*_7_ | 0 | 0.00 | 0.00 | 0.00 | 0.00 | 0.05 | 0.04 | 0.03 | 0.02 | 0.05 | 0.04 | 0.03 | 0.02 |
| γ*_g_*_5_ | γ*_b_*_7_ | 0 | -0.01 | 0.00 | 0.00 | 0.00 | 0.05 | 0.04 | 0.03 | 0.02 | 0.05 | 0.04 | 0.03 | 0.02 |
| γ*_g_*_6_ | γ*_b_*_7_ | 0 | -0.01 | 0.00 | 0.00 | 0.00 | 0.05 | 0.04 | 0.03 | 0.02 | 0.05 | 0.04 | 0.03 | 0.02 |
| γ*_g_*_7_ | γ*_b_*_7_ | 0 | 0.00 | 0.00 | 0.00 | 0.01 | 0.05 | 0.04 | 0.03 | 0.02 | 0.05 | 0.04 | 0.03 | 0.02 |
| γ*_g_*_8_ | γ*_b_*_7_ | 0 | 0.00 | 0.00 | 0.00 | 0.00 | 0.05 | 0.04 | 0.03 | 0.02 | 0.05 | 0.04 | 0.03 | 0.02 |
| γ*_g_*_9_ | γ*_b_*_7_ | 0 | 0.00 | 0.00 | 0.00 | 0.00 | 0.05 | 0.04 | 0.03 | 0.02 | 0.05 | 0.04 | 0.03 | 0.02 |
| z_E_ | γ*_b_*_7_ | 0 | 0.00 | 0.00 | 0.00 | 0.00 | 0.05 | 0.04 | 0.03 | 0.02 | 0.05 | 0.04 | 0.03 | 0.02 |
| γ*_g_*_1×E_ | γ*_b_*_7_ | 0.2 | -0.02 | -0.01 | 0.00 | 0.00 | 0.05 | 0.04 | 0.03 | 0.02 | 0.05 | 0.04 | 0.03 | 0.02 |
| γ*_g_*_2×E_ | γ*_b_*_7_ | 0 | 0.01 | 0.00 | 0.00 | 0.00 | 0.05 | 0.04 | 0.03 | 0.02 | 0.05 | 0.04 | 0.03 | 0.02 |
| γ*_g_*_3×E_ | γ*_b_*_7_ | 0 | 0.00 | 0.00 | 0.00 | 0.00 | 0.05 | 0.04 | 0.03 | 0.02 | 0.05 | 0.04 | 0.03 | 0.02 |
| γ*_g_*_4×E_ | γ*_b_*_7_ | 0 | 0.01 | 0.00 | 0.01 | 0.00 | 0.05 | 0.04 | 0.03 | 0.02 | 0.05 | 0.04 | 0.03 | 0.02 |
| γ*_g_*_5×E_ | γ*_b_*_7_ | 0 | 0.00 | 0.00 | 0.00 | 0.00 | 0.05 | 0.04 | 0.03 | 0.02 | 0.05 | 0.04 | 0.03 | 0.02 |
| γ*_g_*_6×E_ | γ*_b_*_7_ | 0 | 0.00 | 0.00 | 0.00 | 0.00 | 0.05 | 0.04 | 0.03 | 0.02 | 0.05 | 0.04 | 0.03 | 0.02 |
| γ*_g_*_7×E_ | γ*_b_*_7_ | 0 | 0.00 | 0.00 | 0.00 | 0.00 | 0.05 | 0.04 | 0.03 | 0.02 | 0.05 | 0.04 | 0.03 | 0.02 |
| γ*_g_*_8×E_ | γ*_b_*_7_ | 0 | 0.00 | 0.00 | 0.00 | 0.00 | 0.05 | 0.04 | 0.03 | 0.02 | 0.05 | 0.04 | 0.03 | 0.02 |
| γ*_g_*_9×E_ | γ*_b_*_7_ | 0 | 0.00 | 0.00 | 0.00 | 0.00 | 0.05 | 0.04 | 0.03 | 0.02 | 0.05 | 0.04 | 0.03 | 0.02 |
| γ*_g_*_1_ | γ*_b_*_8_ | -0.6 | 0.04 | 0.02 | 0.01 | 0.01 | 0.04 | 0.03 | 0.02 | 0.04 | 0.06 | 0.04 | 0.02 | 0.04 |
| γ*_g_*_2_ | γ*_b_*_8_ | 0 | -0.01 | 0.00 | 0.00 | 0.00 | 0.04 | 0.03 | 0.02 | 0.02 | 0.05 | 0.03 | 0.02 | 0.02 |
| γ*_g_*_3_ | γ*_b_*_8_ | 0 | -0.01 | 0.00 | 0.00 | 0.00 | 0.05 | 0.03 | 0.02 | 0.02 | 0.05 | 0.03 | 0.02 | 0.02 |
| γ*_g_*_4_ | γ*_b_*_8_ | 0 | 0.00 | 0.00 | 0.00 | 0.00 | 0.04 | 0.03 | 0.02 | 0.02 | 0.04 | 0.03 | 0.02 | 0.02 |
| γ*_g_*_5_ | γ*_b_*_8_ | 0 | 0.00 | 0.00 | 0.00 | 0.00 | 0.04 | 0.03 | 0.02 | 0.02 | 0.04 | 0.03 | 0.02 | 0.02 |
| γ*_g_*_6_ | γ*_b_*_8_ | 0 | 0.00 | 0.00 | 0.00 | 0.00 | 0.04 | 0.03 | 0.02 | 0.02 | 0.05 | 0.03 | 0.02 | 0.02 |
| γ*_g_*_7_ | γ*_b_*_8_ | 0 | 0.00 | 0.00 | 0.00 | 0.00 | 0.04 | 0.03 | 0.02 | 0.02 | 0.04 | 0.03 | 0.02 | 0.02 |
| γ*_g_*_8_ | γ*_b_*_8_ | 0 | 0.00 | 0.00 | 0.00 | 0.00 | 0.04 | 0.03 | 0.02 | 0.02 | 0.04 | 0.03 | 0.02 | 0.02 |
| γ*_g_*_9_ | γ*_b_*_8_ | 0 | 0.00 | 0.00 | 0.00 | 0.00 | 0.04 | 0.03 | 0.02 | 0.02 | 0.04 | 0.03 | 0.02 | 0.02 |
| z_E_ | γ*_b_*_8_ | 0.2 | -0.01 | -0.01 | 0.00 | 0.00 | 0.05 | 0.03 | 0.03 | 0.02 | 0.05 | 0.03 | 0.03 | 0.02 |
| γ*_g_*_1×E_ | γ*_b_*_8_ | 0.4 | -0.04 | -0.02 | -0.01 | -0.01 | 0.03 | 0.02 | 0.04 | 0.01 | 0.05 | 0.03 | 0.04 | 0.01 |
| γ*_g_*_2×E_ | γ*_b_*_8_ | 0 | -0.02 | -0.01 | 0.00 | 0.00 | 0.03 | 0.02 | 0.03 | 0.01 | 0.04 | 0.02 | 0.03 | 0.01 |
| γ*_g_*_3×E_ | γ*_b_*_8_ | 0 | 0.01 | 0.01 | 0.00 | 0.00 | 0.03 | 0.02 | 0.01 | 0.01 | 0.03 | 0.02 | 0.01 | 0.01 |
| γ*_g_*_4×E_ | γ*_b_*_8_ | 0 | 0.00 | 0.00 | 0.00 | 0.00 | 0.03 | 0.02 | 0.01 | 0.01 | 0.03 | 0.02 | 0.01 | 0.01 |
| γ*_g_*_5×E_ | γ*_b_*_8_ | 0 | 0.00 | 0.00 | 0.00 | 0.00 | 0.03 | 0.02 | 0.01 | 0.01 | 0.03 | 0.02 | 0.01 | 0.01 |
| γ*_g_*_6×E_ | γ*_b_*_8_ | 0 | 0.00 | 0.00 | 0.00 | 0.00 | 0.03 | 0.02 | 0.01 | 0.01 | 0.03 | 0.02 | 0.01 | 0.01 |
| γ*_g_*_7×E_ | γ*_b_*_8_ | 0 | 0.00 | 0.00 | 0.00 | 0.00 | 0.03 | 0.02 | 0.01 | 0.01 | 0.03 | 0.02 | 0.01 | 0.01 |
| γ*_g_*_8×E_ | γ*_b_*_8_ | 0 | 0.00 | 0.00 | 0.00 | 0.00 | 0.03 | 0.02 | 0.01 | 0.01 | 0.03 | 0.02 | 0.01 | 0.01 |
| γ*_g_*_9×E_ | γ*_b_*_8_ | 0 |  | 0.00 | 0.00 | 0.00 | 0.03 | 0.02 | 0.01 | 0.01 | 0.03 | 0.02 | 0.01 | 0.01 |
| γ*_g_*_1_ | γ*_b_*_9_ | 0.6 | -0.02 | -0.01 | -0.01 | 0.00 | 0.03 | 0.02 | 0.02 | 0.01 | 0.04 | 0.03 | 0.02 | 0.01 |
| γ*_g_*_2_ | γ*_b_*_9_ | 0.4 | 0.03 | 0.01 | 0.01 | 0.00 | 0.03 | 0.02 | 0.03 | 0.01 | 0.04 | 0.03 | 0.03 | 0.01 |
| γ*_g_*_3_ | γ*_b_*_9_ | 0 | -0.01 | 0.00 | 0.00 | 0.00 | 0.03 | 0.02 | 0.01 | 0.01 | 0.03 | 0.02 | 0.01 | 0.01 |
| γ*_g_*_4_ | γ*_b_*_9_ | 0 | -0.01 | 0.00 | 0.00 | 0.00 | 0.03 | 0.02 | 0.01 | 0.01 | 0.03 | 0.02 | 0.01 | 0.01 |
| γ*_g_*_5_ | γ*_b_*_9_ | 0 | 0.00 | 0.00 | 0.00 | 0.00 | 0.03 | 0.02 | 0.01 | 0.01 | 0.03 | 0.02 | 0.01 | 0.01 |
| γ*_g_*_6_ | γ*_b_*_9_ | 0 | 0.00 | 0.00 | 0.00 | 0.00 | 0.03 | 0.02 | 0.01 | 0.01 | 0.03 | 0.02 | 0.01 | 0.01 |
| γ*_g_*_7_ | γ*_b_*_9_ | 0 | 0.00 | 0.00 | 0.00 | 0.00 | 0.03 | 0.02 | 0.01 | 0.01 | 0.03 | 0.02 | 0.01 | 0.01 |
| γ*_g_*_8_ | γ*_b_*_9_ | 0 | 0.00 | 0.00 | 0.00 | 0.00 | 0.03 | 0.02 | 0.01 | 0.01 | 0.03 | 0.02 | 0.01 | 0.01 |
| γ*_g_*_9_ | γ*_b_*_9_ | 0 | 0.00 | 0.00 | 0.00 | 0.00 | 0.03 | 0.02 | 0.01 | 0.01 | 0.03 | 0.02 | 0.01 | 0.01 |
| z_E_ | γ*_b_*_9_ | 0.2 | 0.00 | 0.00 | 0.00 | 0.00 | 0.03 | 0.02 | 0.01 | 0.01 | 0.03 | 0.02 | 0.01 | 0.01 |
| γ*_g_*_1×E_ | γ*_b_*_9_ | -0.4 | -0.01 | 0.00 | 0.00 | 0.00 | 0.05 | 0.03 | 0.02 | 0.02 | 0.05 | 0.03 | 0.02 | 0.02 |
| γ*_g_*_2×E_ | γ*_b_*_9_ | 0 | 0.05 | 0.02 | 0.01 | 0.01 | 0.04 | 0.05 | 0.04 | 0.04 | 0.06 | 0.05 | 0.05 | 0.04 |
| γ*_g_*_3×E_ | γ*_b_*_9_ | 0 | -0.04 | -0.02 | -0.01 | -0.01 | 0.05 | 0.04 | 0.04 | 0.03 | 0.06 | 0.05 | 0.04 | 0.03 |
| γ*_g_*_4×E_ | γ*_b_*_9_ | 0 | 0.01 | 0.00 | 0.00 | 0.00 | 0.04 | 0.03 | 0.02 | 0.02 | 0.04 | 0.03 | 0.02 | 0.02 |
| γ*_g_*_5×E_ | γ*_b_*_9_ | 0 | 0.00 | 0.00 | 0.00 | 0.00 | 0.04 | 0.03 | 0.02 | 0.02 | 0.04 | 0.03 | 0.02 | 0.02 |
| γ*_g_*_6×E_ | γ*_b_*_9_ | 0 | 0.00 | 0.00 | 0.00 | 0.00 | 0.05 | 0.03 | 0.02 | 0.02 | 0.05 | 0.03 | 0.02 | 0.02 |
| γ*_g_*_7×E_ | γ*_b_*_9_ | 0 | 0.00 | 0.00 | 0.00 | 0.00 | 0.04 | 0.03 | 0.02 | 0.02 | 0.04 | 0.03 | 0.02 | 0.02 |
| γ*_g_*_8×E_ | γ*_b_*_9_ | 0 | 0.00 | 0.00 | 0.00 | 0.00 | 0.05 | 0.03 | 0.02 | 0.02 | 0.05 | 0.03 | 0.02 | 0.02 |
| γ*_g_*_9×E_ | γ*_b_*_9_ | 0 | 0.00 | 0.00 | 0.00 | 0.00 | 0.05 | 0.03 | 0.02 | 0.02 | 0.05 | 0.03 | 0.02 | 0.02 |
| γ*_g_*_1_ | γ*_b_*_10_ | 0 | 0.00 | 0.00 | 0.00 | 0.00 | 0.05 | 0.04 | 0.02 | 0.02 | 0.05 | 0.04 | 0.02 | 0.02 |
| γ*_g_*_2_ | γ*_b_*_10_ | -0.6 | 0.00 | 0.00 | 0.00 | 0.00 | 0.05 | 0.03 | 0.02 | 0.02 | 0.05 | 0.03 | 0.02 | 0.02 |
| γ*_g_*_3_ | γ*_b_*_10_ | 0.4 | -0.02 | -0.01 | -0.01 | 0.00 | 0.05 | 0.04 | 0.03 | 0.02 | 0.05 | 0.04 | 0.03 | 0.02 |
| γ*_g_*_4_ | γ*_b_*_10_ | 0 | 0.03 | 0.02 | 0.01 | 0.00 | 0.05 | 0.04 | 0.04 | 0.03 | 0.06 | 0.05 | 0.04 | 0.03 |
| γ*_g_*_5_ | γ*_b_*_10_ | 0 | -0.01 | 0.00 | -0.01 | 0.00 | 0.05 | 0.03 | 0.02 | 0.02 | 0.05 | 0.03 | 0.03 | 0.02 |
| γ*_g_*_6_ | γ*_b_*_10_ | 0 | 0.00 | 0.00 | 0.00 | 0.00 | 0.05 | 0.03 | 0.02 | 0.02 | 0.05 | 0.03 | 0.02 | 0.02 |
| γ*_g_*_7_ | γ*_b_*_10_ | 0 | 0.00 | 0.00 | 0.00 | 0.00 | 0.05 | 0.03 | 0.02 | 0.02 | 0.05 | 0.03 | 0.02 | 0.02 |
| γ*_g_*_8_ | γ*_b_*_10_ | 0 | 0.00 | 0.00 | 0.00 | 0.00 | 0.05 | 0.03 | 0.02 | 0.02 | 0.05 | 0.03 | 0.02 | 0.02 |
| γ*_g_*_9_ | γ*_b_*_10_ | 0 | 0.00 | 0.00 | 0.00 | 0.00 | 0.05 | 0.04 | 0.02 | 0.02 | 0.05 | 0.04 | 0.02 | 0.02 |
| z_E_ | γ*_b_*_10_ | 0 | 0.00 | 0.00 | 0.00 | 0.00 | 0.05 | 0.03 | 0.02 | 0.02 | 0.05 | 0.03 | 0.02 | 0.02 |
| γ*_g_*_1×E_ | γ*_b_*_10_ | 0 | -0.04 | -0.02 | -0.01 | -0.01 | 0.05 | 0.04 | 0.03 | 0.03 | 0.06 | 0.04 | 0.03 | 0.03 |
| γ*_g_*_2×E_ | γ*_b_*_10_ | 0.2 | 0.00 | 0.00 | 0.00 | 0.00 | 0.05 | 0.04 | 0.03 | 0.02 | 0.05 | 0.04 | 0.03 | 0.02 |
| γ*_g_*_3×E_ | γ*_b_*_10_ | -0.4 | 0.05 | 0.02 | 0.01 | 0.01 | 0.04 | 0.03 | 0.02 | 0.04 | 0.06 | 0.04 | 0.02 | 0.04 |
| γ*_g_*_4×E_ | γ*_b_*_10_ | 0 | 0.00 | 0.00 | 0.00 | 0.00 | 0.05 | 0.04 | 0.03 | 0.02 | 0.05 | 0.04 | 0.03 | 0.02 |
| γ*_g_*_5×E_ | γ*_b_*_10_ | 0 | 0.00 | 0.00 | 0.00 | 0.00 | 0.05 | 0.04 | 0.03 | 0.02 | 0.05 | 0.04 | 0.03 | 0.02 |
| γ*_g_*_6×E_ | γ*_b_*_10_ | 0 | 0.01 | 0.01 | 0.01 | 0.00 | 0.05 | 0.04 | 0.03 | 0.02 | 0.05 | 0.04 | 0.03 | 0.02 |
| γ*_g_*_7×E_ | γ*_b_*_10_ | 0 | 0.00 | 0.00 | 0.00 | 0.00 | 0.05 | 0.04 | 0.03 | 0.02 | 0.05 | 0.04 | 0.03 | 0.02 |
| γ*_g_*_8×E_ | γ*_b_*_10_ | 0 | 0.00 | 0.00 | 0.00 | 0.00 | 0.05 | 0.04 | 0.03 | 0.02 | 0.05 | 0.04 | 0.03 | 0.02 |
| γ*_g_*_9×E_ | γ*_b_*_10_ | 0 | 0.00 | 0.00 | 0.00 | 0.00 | 0.05 | 0.04 | 0.03 | 0.02 | 0.05 | 0.04 | 0.03 | 0.02 |
| γ*_g_*_1_ | γ*_b_*_11_ | 0.4 | 0.00 | 0.00 | 0.00 | 0.00 | 0.05 | 0.03 | 0.03 | 0.02 | 0.05 | 0.03 | 0.03 | 0.02 |
| γ*_g_*_2_ | γ*_b_*_11_ | 0 | -0.02 | -0.01 | -0.01 | -0.01 | 0.05 | 0.04 | 0.03 | 0.02 | 0.05 | 0.04 | 0.03 | 0.02 |
| γ*_g_*_3_ | γ*_b_*_11_ | -0.6 | 0.01 | 0.00 | 0.00 | 0.00 | 0.05 | 0.04 | 0.03 | 0.02 | 0.05 | 0.04 | 0.03 | 0.02 |
| γ*_g_*_4_ | γ*_b_*_11_ | 0 | 0.00 | 0.00 | 0.00 | 0.00 | 0.05 | 0.04 | 0.03 | 0.02 | 0.05 | 0.04 | 0.03 | 0.02 |
| γ*_g_*_5_ | γ*_b_*_11_ | 0 | 0.01 | 0.01 | 0.01 | 0.01 | 0.05 | 0.04 | 0.03 | 0.02 | 0.05 | 0.04 | 0.03 | 0.02 |
| γ*_g_*_6_ | γ*_b_*_11_ | 0 | 0.00 | 0.00 | 0.00 | 0.00 | 0.05 | 0.04 | 0.03 | 0.02 | 0.05 | 0.04 | 0.03 | 0.02 |
| γ*_g_*_7_ | γ*_b_*_11_ | 0 | 0.00 | 0.00 | 0.00 | 0.00 | 0.05 | 0.04 | 0.03 | 0.02 | 0.05 | 0.04 | 0.03 | 0.02 |
| γ*_g_*_8_ | γ*_b_*_11_ | 0 | 0.00 | 0.00 | 0.00 | 0.00 | 0.05 | 0.04 | 0.03 | 0.02 | 0.05 | 0.04 | 0.03 | 0.02 |
| γ*_g_*_9_ | γ*_b_*_11_ | 0 | 0.00 | 0.01 | 0.00 | 0.00 | 0.05 | 0.04 | 0.03 | 0.02 | 0.05 | 0.04 | 0.03 | 0.02 |
| z_E_ | γ*_b_*_11_ | 0 | 0.00 | 0.00 | 0.00 | 0.00 | 0.05 | 0.04 | 0.03 | 0.02 | 0.05 | 0.04 | 0.03 | 0.02 |
| γ*_g_*_1×E_ | γ*_b_*_11_ | 0.2 | 0.04 | 0.02 | 0.01 | 0.00 | 0.04 | 0.05 | 0.04 | 0.04 | 0.06 | 0.05 | 0.04 | 0.04 |
| γ*_g_*_2×E_ | γ*_b_*_11_ | 0 | -0.01 | -0.01 | 0.00 | 0.00 | 0.04 | 0.03 | 0.02 | 0.02 | 0.04 | 0.03 | 0.02 | 0.02 |
| γ*_g_*_3×E_ | γ*_b_*_11_ | 0 | -0.01 | 0.00 | 0.00 | 0.00 | 0.04 | 0.03 | 0.02 | 0.02 | 0.05 | 0.03 | 0.02 | 0.02 |
| γ*_g_*_4×E_ | γ*_b_*_11_ | 0 | 0.00 | 0.00 | 0.00 | 0.00 | 0.04 | 0.03 | 0.02 | 0.02 | 0.04 | 0.03 | 0.02 | 0.02 |
| γ*_g_*_5×E_ | γ*_b_*_11_ | 0 | 0.00 | 0.00 | 0.00 | 0.00 | 0.04 | 0.03 | 0.02 | 0.02 | 0.04 | 0.03 | 0.02 | 0.02 |
| γ*_g_*_6×E_ | γ*_b_*_11_ | 0 | 0.00 | 0.00 | 0.00 | 0.00 | 0.04 | 0.03 | 0.02 | 0.02 | 0.04 | 0.03 | 0.02 | 0.02 |
| γ*_g_*_7×E_ | γ*_b_*_11_ | 0 | 0.00 | 0.00 | 0.00 | 0.00 | 0.04 | 0.03 | 0.02 | 0.02 | 0.04 | 0.03 | 0.02 | 0.02 |
| γ*_g_*_8×E_ | γ*_b_*_11_ | 0 | 0.00 | 0.00 | 0.00 | 0.00 | 0.04 | 0.03 | 0.02 | 0.02 | 0.04 | 0.03 | 0.02 | 0.02 |
| γ*_g_*_9×E_ | γ*_b_*_11_ | 0 | -0.01 | -0.01 | -0.01 | -0.01 | 0.04 | 0.03 | 0.02 | 0.02 | 0.04 | 0.03 | 0.02 | 0.02 |
| γ*_g_*_1_ | γ*_b_*_12_ | -0.6 | -0.02 | -0.01 | -0.01 | -0.01 | 0.05 | 0.04 | 0.03 | 0.02 | 0.05 | 0.04 | 0.03 | 0.02 |
| γ*_g_*_2_ | γ*_b_*_12_ | 0 | -0.03 | -0.02 | -0.01 | -0.01 | 0.05 | 0.04 | 0.03 | 0.03 | 0.05 | 0.04 | 0.04 | 0.03 |
| γ*_g_*_3_ | γ*_b_*_12_ | 0 | 0.01 | 0.00 | 0.00 | 0.00 | 0.05 | 0.03 | 0.02 | 0.02 | 0.05 | 0.03 | 0.02 | 0.02 |
| γ*_g_*_4_ | γ*_b_*_12_ | 0 | 0.00 | 0.00 | 0.00 | 0.00 | 0.05 | 0.03 | 0.02 | 0.02 | 0.05 | 0.03 | 0.02 | 0.02 |
| γ*_g_*_5_ | γ*_b_*_12_ | 0 | 0.00 | 0.00 | 0.00 | 0.00 | 0.04 | 0.03 | 0.02 | 0.02 | 0.04 | 0.03 | 0.02 | 0.02 |
| γ*_g_*_6_ | γ*_b_*_12_ | 0 | 0.01 | 0.01 | 0.01 | 0.01 | 0.05 | 0.03 | 0.02 | 0.02 | 0.05 | 0.03 | 0.02 | 0.02 |
| γ*_g_*_7_ | γ*_b_*_12_ | 0 | 0.00 | 0.00 | 0.00 | 0.00 | 0.05 | 0.03 | 0.02 | 0.02 | 0.05 | 0.03 | 0.02 | 0.02 |
| γ*_g_*_8_ | γ*_b_*_12_ | 0 | 0.00 | 0.00 | 0.00 | 0.00 | 0.05 | 0.03 | 0.02 | 0.02 | 0.05 | 0.03 | 0.02 | 0.02 |
| γ*_g_*_9_ | γ*_b_*_12_ | 0 | 0.00 | 0.00 | 0.00 | 0.00 | 0.04 | 0.03 | 0.02 | 0.02 | 0.04 | 0.03 | 0.02 | 0.02 |
| z_E_ | γ*_b_*_12_ | 0.2 | 0.00 | 0.00 | 0.00 | 0.00 | 0.05 | 0.03 | 0.02 | 0.02 | 0.05 | 0.03 | 0.02 | 0.02 |
| γ*_g_*_1×E_ | γ*_b_*_12_ | 0.4 | -0.04 | -0.02 | -0.01 | 0.00 | 0.03 | 0.02 | 0.02 | 0.01 | 0.05 | 0.03 | 0.02 | 0.01 |
| γ*_g_*_2×E_ | γ*_b_*_12_ | 0 | -0.02 | -0.01 | 0.00 | 0.00 | 0.03 | 0.02 | 0.02 | 0.01 | 0.03 | 0.02 | 0.02 | 0.01 |
| γ*_g_*_3×E_ | γ*_b_*_12_ | 0 | 0.02 | 0.01 | 0.00 | 0.00 | 0.03 | 0.02 | 0.01 | 0.01 | 0.03 | 0.02 | 0.01 | 0.01 |
| γ*_g_*_4×E_ | γ*_b_*_12_ | 0 | 0.00 | 0.00 | 0.00 | 0.00 | 0.03 | 0.02 | 0.01 | 0.01 | 0.03 | 0.02 | 0.01 | 0.01 |
| γ*_g_*_5×E_ | γ*_b_*_12_ | 0 | 0.00 | 0.00 | 0.00 | 0.00 | 0.03 | 0.02 | 0.01 | 0.01 | 0.03 | 0.02 | 0.01 | 0.01 |
| γ*_g_*_6×E_ | γ*_b_*_12_ | 0 | 0.00 | 0.00 | 0.00 | 0.00 | 0.03 | 0.02 | 0.01 | 0.01 | 0.03 | 0.02 | 0.01 | 0.01 |
| γ*_g_*_7×E_ | γ*_b_*_12_ | 0 | 0.00 | 0.00 | 0.00 | 0.00 | 0.03 | 0.02 | 0.01 | 0.01 | 0.03 | 0.02 | 0.01 | 0.01 |
| γ*_g_*_8×E_ | γ*_b_*_12_ | 0 | 0.00 | 0.00 | 0.00 | 0.00 | 0.03 | 0.02 | 0.01 | 0.01 | 0.03 | 0.02 | 0.01 | 0.01 |
| γ*_g_*_9×E_ | γ*_b_*_12_ | 0 | 0.00 | 0.00 | 0.00 | 0.00 | 0.03 | 0.02 | 0.01 | 0.01 | 0.03 | 0.02 | 0.01 | 0.01 |
| γ*_g_*_1_ | γ*_b_*_13_ | 0.6 | -0.02 | -0.01 | -0.01 | 0.00 | 0.03 | 0.02 | 0.02 | 0.01 | 0.04 | 0.03 | 0.02 | 0.01 |
| γ*_g_*_2_ | γ*_b_*_13_ | 0.4 | 0.03 | 0.02 | 0.01 | 0.01 | 0.03 | 0.02 | 0.02 | 0.01 | 0.04 | 0.03 | 0.02 | 0.01 |
| γ*_g_*_3_ | γ*_b_*_13_ | 0 | -0.01 | 0.00 | 0.00 | 0.00 | 0.03 | 0.02 | 0.01 | 0.01 | 0.03 | 0.02 | 0.01 | 0.01 |
| γ*_g_*_4_ | γ*_b_*_13_ | 0 | -0.01 | 0.00 | 0.00 | 0.00 | 0.03 | 0.02 | 0.01 | 0.01 | 0.03 | 0.02 | 0.01 | 0.01 |
| γ*_g_*_5_ | γ*_b_*_13_ | 0 | 0.00 | 0.00 | 0.00 | 0.00 | 0.03 | 0.02 | 0.01 | 0.01 | 0.03 | 0.02 | 0.01 | 0.01 |
| γ*_g_*_6_ | γ*_b_*_13_ | 0 | 0.00 | 0.00 | 0.00 | 0.00 | 0.03 | 0.02 | 0.01 | 0.01 | 0.03 | 0.02 | 0.01 | 0.01 |
| γ*_g_*_7_ | γ*_b_*_13_ | 0 | 0.00 | 0.00 | 0.00 | 0.00 | 0.03 | 0.02 | 0.01 | 0.01 | 0.03 | 0.02 | 0.01 | 0.01 |
| γ*_g_*_8_ | γ*_b_*_13_ | 0 | 0.00 | 0.00 | 0.00 | 0.00 | 0.03 | 0.02 | 0.01 | 0.01 | 0.03 | 0.02 | 0.01 | 0.01 |
| γ*_g_*_9_ | γ*_b_*_13_ | 0 | 0.00 | 0.00 | 0.00 | 0.00 | 0.03 | 0.02 | 0.01 | 0.01 | 0.03 | 0.02 | 0.01 | 0.01 |
| z_E_ | γ*_b_*_13_ | 0.2 | 0.00 | 0.00 | 0.00 | 0.00 | 0.03 | 0.02 | 0.01 | 0.01 | 0.03 | 0.02 | 0.01 | 0.01 |
| γ*_g_*_1×E_ | γ*_b_*_13_ | -0.4 | 0.00 | 0.00 | 0.00 | 0.00 | 0.05 | 0.03 | 0.02 | 0.02 | 0.05 | 0.03 | 0.02 | 0.02 |
| γ*_g_*_2×E_ | γ*_b_*_13_ | 0 | 0.05 | 0.02 | 0.01 | 0.00 | 0.07 | 0.05 | 0.02 | 0.02 | 0.09 | 0.05 | 0.02 | 0.02 |
| γ*_g_*_3×E_ | γ*_b_*_13_ | 0 | -0.04 | -0.02 | -0.01 | -0.01 | 0.06 | 0.04 | 0.03 | 0.02 | 0.07 | 0.05 | 0.03 | 0.02 |
| γ*_g_*_4×E_ | γ*_b_*_13_ | 0 | 0.00 | 0.00 | -0.01 | 0.00 | 0.05 | 0.03 | 0.02 | 0.02 | 0.05 | 0.03 | 0.02 | 0.02 |
| γ*_g_*_5×E_ | γ*_b_*_13_ | 0 | 0.00 | 0.00 | 0.00 | 0.00 | 0.05 | 0.03 | 0.03 | 0.02 | 0.05 | 0.03 | 0.03 | 0.02 |
| γ*_g_*_6×E_ | γ*_b_*_13_ | 0 | 0.00 | 0.00 | 0.00 | 0.00 | 0.05 | 0.03 | 0.02 | 0.02 | 0.05 | 0.03 | 0.02 | 0.02 |
| γ*_g_*_7×E_ | γ*_b_*_13_ | 0 | 0.00 | 0.00 | 0.00 | 0.00 | 0.04 | 0.03 | 0.02 | 0.02 | 0.04 | 0.03 | 0.02 | 0.02 |
| γ*_g_*_8×E_ | γ*_b_*_13_ | 0 | 0.00 | 0.00 | 0.00 | 0.00 | 0.05 | 0.03 | 0.02 | 0.02 | 0.05 | 0.03 | 0.02 | 0.02 |
| γ*_g_*_9×E_ | γ*_b_*_13_ | 0 | 0.00 | 0.00 | 0.00 | 0.00 | 0.05 | 0.04 | 0.02 | 0.02 | 0.05 | 0.04 | 0.02 | 0.02 |
| γ*_g_*_1_ | γ*_b_*_14_ | 0 | 0.00 | 0.00 | 0.00 | 0.00 | 0.05 | 0.04 | 0.02 | 0.02 | 0.05 | 0.04 | 0.03 | 0.02 |
| γ*_g_*_2_ | γ*_b_*_14_ | -0.6 | 0.00 | 0.00 | 0.00 | 0.01 | 0.05 | 0.04 | 0.02 | 0.02 | 0.05 | 0.04 | 0.02 | 0.02 |
| γ*_g_*_3_ | γ*_b_*_14_ | 0.4 | -0.03 | -0.01 | -0.01 | 0.00 | 0.05 | 0.04 | 0.02 | 0.02 | 0.06 | 0.04 | 0.02 | 0.02 |
| γ*_g_*_4_ | γ*_b_*_14_ | 0 | 0.04 | 0.02 | 0.01 | 0.01 | 0.06 | 0.04 | 0.03 | 0.02 | 0.07 | 0.05 | 0.03 | 0.02 |
| γ*_g_*_5_ | γ*_b_*_14_ | 0 | 0.00 | 0.00 | 0.00 | 0.00 | 0.05 | 0.03 | 0.02 | 0.02 | 0.05 | 0.03 | 0.02 | 0.02 |
| γ*_g_*_6_ | γ*_b_*_14_ | 0 | 0.00 | 0.00 | 0.00 | 0.00 | 0.05 | 0.03 | 0.02 | 0.02 | 0.05 | 0.03 | 0.02 | 0.02 |
| γ*_g_*_7_ | γ*_b_*_14_ | 0 | 0.00 | 0.00 | 0.00 | 0.00 | 0.05 | 0.03 | 0.02 | 0.02 | 0.05 | 0.03 | 0.02 | 0.02 |
| γ*_g_*_8_ | γ*_b_*_14_ | 0 | 0.00 | 0.00 | 0.00 | 0.00 | 0.05 | 0.03 | 0.02 | 0.02 | 0.05 | 0.03 | 0.02 | 0.02 |
| γ*_g_*_9_ | γ*_b_*_14_ | 0 | 0.00 | 0.00 | 0.00 | 0.00 | 0.05 | 0.03 | 0.02 | 0.02 | 0.05 | 0.03 | 0.02 | 0.02 |
| z_E_ | γ*_b_*_14_ | 0 | 0.00 | 0.00 | 0.00 | 0.00 | 0.05 | 0.03 | 0.02 | 0.02 | 0.05 | 0.03 | 0.02 | 0.02 |
| γ*_g_*_1×E_ | γ*_b_*_14_ | 0 | -0.04 | -0.02 | -0.01 | -0.01 | 0.05 | 0.04 | 0.02 | 0.02 | 0.06 | 0.04 | 0.03 | 0.02 |
| γ*_g_*_2×E_ | γ*_b_*_14_ | 0.2 | -0.01 | 0.00 | 0.00 | 0.00 | 0.05 | 0.04 | 0.03 | 0.02 | 0.05 | 0.04 | 0.03 | 0.02 |
| γ*_g_*_3×E_ | γ*_b_*_14_ | -0.4 | 0.05 | 0.03 | 0.02 | 0.01 | 0.04 | 0.03 | 0.02 | 0.02 | 0.07 | 0.04 | 0.03 | 0.02 |
| γ*_g_*_4×E_ | γ*_b_*_14_ | 0 | 0.00 | 0.00 | 0.00 | 0.00 | 0.05 | 0.04 | 0.03 | 0.02 | 0.05 | 0.04 | 0.03 | 0.02 |
| γ*_g_*_5×E_ | γ*_b_*_14_ | 0 | -0.01 | 0.00 | 0.00 | 0.00 | 0.05 | 0.04 | 0.03 | 0.02 | 0.05 | 0.04 | 0.03 | 0.02 |
| γ*_g_*_6×E_ | γ*_b_*_14_ | 0 | -0.01 | 0.00 | 0.00 | 0.00 | 0.05 | 0.04 | 0.03 | 0.02 | 0.05 | 0.04 | 0.03 | 0.02 |
| γ*_g_*_7×E_ | γ*_b_*_14_ | 0 | 0.00 | 0.00 | 0.00 | 0.01 | 0.05 | 0.04 | 0.03 | 0.02 | 0.05 | 0.04 | 0.03 | 0.02 |
| γ*_g_*_8×E_ | γ*_b_*_14_ | 0 | 0.00 | 0.00 | 0.00 | 0.00 | 0.05 | 0.04 | 0.03 | 0.02 | 0.05 | 0.04 | 0.03 | 0.02 |
| γ*_g_*_9×E_ | γ*_b_*_14_ | 0 | 0.00 | 0.00 | 0.00 | 0.00 | 0.05 | 0.04 | 0.03 | 0.02 | 0.05 | 0.04 | 0.03 | 0.02 |
| γ*_g_*_1_ | γ*_b_*_15_ | 0.4 | 0.00 | 0.00 | 0.00 | 0.00 | 0.05 | 0.04 | 0.03 | 0.02 | 0.05 | 0.04 | 0.03 | 0.02 |
| γ*_g_*_2_ | γ*_b_*_15_ | 0 | -0.02 | -0.01 | 0.00 | 0.00 | 0.05 | 0.04 | 0.03 | 0.02 | 0.05 | 0.04 | 0.03 | 0.02 |
| γ*_g_*_3_ | γ*_b_*_15_ | -0.6 | 0.01 | 0.00 | 0.00 | 0.00 | 0.05 | 0.04 | 0.03 | 0.02 | 0.05 | 0.04 | 0.03 | 0.02 |
| γ*_g_*_4_ | γ*_b_*_15_ | 0 | 0.00 | 0.00 | 0.00 | 0.00 | 0.05 | 0.04 | 0.03 | 0.02 | 0.05 | 0.04 | 0.03 | 0.02 |
| γ*_g_*_5_ | γ*_b_*_15_ | 0 | 0.01 | 0.00 | 0.01 | 0.00 | 0.05 | 0.04 | 0.03 | 0.02 | 0.05 | 0.04 | 0.03 | 0.02 |
| γ*_g_*_6_ | γ*_b_*_15_ | 0 | 0.00 | 0.00 | 0.00 | 0.00 | 0.05 | 0.04 | 0.03 | 0.02 | 0.05 | 0.04 | 0.03 | 0.02 |
| γ*_g_*_7_ | γ*_b_*_15_ | 0 | 0.00 | 0.00 | 0.00 | 0.00 | 0.05 | 0.04 | 0.03 | 0.02 | 0.05 | 0.04 | 0.03 | 0.02 |
| γ*_g_*_8_ | γ*_b_*_15_ | 0 | 0.00 | 0.00 | 0.00 | 0.00 | 0.05 | 0.04 | 0.03 | 0.02 | 0.05 | 0.04 | 0.03 | 0.02 |
| γ*_g_*_9_ | γ*_b_*_15_ | 0 | 0.00 | 0.00 | 0.00 | 0.00 | 0.05 | 0.04 | 0.03 | 0.02 | 0.05 | 0.04 | 0.03 | 0.02 |
| z_E_ | γ*_b_*_15_ | 0 | 0.00 | 0.00 | 0.00 | 0.00 | 0.05 | 0.04 | 0.03 | 0.02 | 0.05 | 0.04 | 0.03 | 0.02 |
| γ*_g_*_1×E_ | γ*_b_*_15_ | 0.2 | 0.04 | 0.02 | 0.01 | 0.01 | 0.04 | 0.03 | 0.02 | 0.04 | 0.06 | 0.04 | 0.02 | 0.04 |
| γ*_g_*_2×E_ | γ*_b_*_15_ | 0 | -0.01 | 0.00 | 0.00 | 0.00 | 0.04 | 0.03 | 0.02 | 0.02 | 0.05 | 0.03 | 0.02 | 0.02 |
| γ*_g_*_3×E_ | γ*_b_*_15_ | 0 | -0.01 | 0.00 | 0.00 | 0.00 | 0.05 | 0.03 | 0.02 | 0.02 | 0.05 | 0.03 | 0.02 | 0.02 |
| γ*_g_*_4×E_ | γ*_b_*_15_ | 0 | 0.00 | 0.00 | 0.00 | 0.00 | 0.04 | 0.03 | 0.02 | 0.02 | 0.04 | 0.03 | 0.02 | 0.02 |
| γ*_g_*_5×E_ | γ*_b_*_15_ | 0 | 0.00 | 0.00 | 0.00 | 0.00 | 0.04 | 0.03 | 0.02 | 0.02 | 0.04 | 0.03 | 0.02 | 0.02 |
| γ*_g_*_6×E_ | γ*_b_*_15_ | 0 | 0.00 | 0.00 | 0.00 | 0.00 | 0.04 | 0.03 | 0.02 | 0.02 | 0.05 | 0.03 | 0.02 | 0.02 |
| γ*_g_*_7×E_ | γ*_b_*_15_ | 0 | 0.00 | 0.00 | 0.00 | 0.00 | 0.04 | 0.03 | 0.02 | 0.02 | 0.04 | 0.03 | 0.02 | 0.02 |
| γ*_g_*_8×E_ | γ*_b_*_15_ | 0 | 0.00 | 0.00 | 0.00 | 0.00 | 0.04 | 0.03 | 0.02 | 0.02 | 0.04 | 0.03 | 0.02 | 0.02 |
| γ*_g_*_9×E_ | γ*_b_*_15_ | 0 | 0.00 | 0.00 | 0.00 | 0.00 | 0.04 | 0.03 | 0.02 | 0.02 | 0.04 | 0.03 | 0.02 | 0.02 |
| γ*_g_*_1_ | γ*_b_*_16_ | -0.6 | -0.01 | -0.01 | 0.00 | 0.00 | 0.05 | 0.03 | 0.03 | 0.02 | 0.05 | 0.03 | 0.03 | 0.02 |
| γ*_g_*_2_ | γ*_b_*_16_ | 0 | -0.04 | -0.02 | -0.01 | -0.01 | 0.03 | 0.02 | 0.04 | 0.01 | 0.05 | 0.03 | 0.04 | 0.01 |
| γ*_g_*_3_ | γ*_b_*_16_ | 0 | -0.02 | -0.01 | 0.00 | 0.00 | 0.03 | 0.02 | 0.03 | 0.01 | 0.04 | 0.02 | 0.03 | 0.01 |
| γ*_g_*_4_ | γ*_b_*_16_ | 0 | 0.01 | 0.01 | 0.00 | 0.00 | 0.03 | 0.02 | 0.01 | 0.01 | 0.03 | 0.02 | 0.01 | 0.01 |
| γ*_g_*_5_ | γ*_b_*_16_ | 0 | 0.00 | 0.00 | 0.00 | 0.00 | 0.03 | 0.02 | 0.01 | 0.01 | 0.03 | 0.02 | 0.01 | 0.01 |
| γ*_g_*_6_ | γ*_b_*_16_ | 0 | 0.00 | 0.00 | 0.00 | 0.00 | 0.03 | 0.02 | 0.01 | 0.01 | 0.03 | 0.02 | 0.01 | 0.01 |
| γ*_g_*_7_ | γ*_b_*_16_ | 0 | 0.00 | 0.00 | 0.00 | 0.00 | 0.03 | 0.02 | 0.01 | 0.01 | 0.03 | 0.02 | 0.01 | 0.01 |
| γ*_g_*_8_ | γ*_b_*_16_ | 0 | 0.00 | 0.00 | 0.00 | 0.00 | 0.03 | 0.02 | 0.01 | 0.01 | 0.03 | 0.02 | 0.01 | 0.01 |
| γ*_g_*_9_ | γ*_b_*_16_ | 0 | 0.00 | 0.00 | 0.00 | 0.00 | 0.03 | 0.02 | 0.01 | 0.01 | 0.03 | 0.02 | 0.01 | 0.01 |
| z_E_ | γ*_b_*_16_ | 0.2 |  | 0.00 | 0.00 | 0.00 | 0.03 | 0.02 | 0.01 | 0.01 | 0.03 | 0.02 | 0.01 | 0.01 |
| γ*_g_*_1×E_ | γ*_b_*_16_ | 0.4 | -0.02 | -0.01 | -0.01 | 0.00 | 0.03 | 0.02 | 0.02 | 0.01 | 0.04 | 0.03 | 0.02 | 0.01 |
| γ*_g_*_2×E_ | γ*_b_*_16_ | 0 | 0.03 | 0.01 | 0.01 | 0.00 | 0.03 | 0.02 | 0.03 | 0.01 | 0.04 | 0.03 | 0.03 | 0.01 |
| γ*_g_*_3×E_ | γ*_b_*_16_ | 0 | -0.01 | 0.00 | 0.00 | 0.00 | 0.03 | 0.02 | 0.01 | 0.01 | 0.03 | 0.02 | 0.01 | 0.01 |
| γ*_g_*_4×E_ | γ*_b_*_16_ | 0 | -0.01 | 0.00 | 0.00 | 0.00 | 0.03 | 0.02 | 0.01 | 0.01 | 0.03 | 0.02 | 0.01 | 0.01 |
| γ*_g_*_5×E_ | γ*_b_*_16_ | 0 | 0.00 | 0.00 | 0.00 | 0.00 | 0.03 | 0.02 | 0.01 | 0.01 | 0.03 | 0.02 | 0.01 | 0.01 |
| γ*_g_*_6×E_ | γ*_b_*_16_ | 0 | 0.00 | 0.00 | 0.00 | 0.00 | 0.03 | 0.02 | 0.01 | 0.01 | 0.03 | 0.02 | 0.01 | 0.01 |
| γ*_g_*_7×E_ | γ*_b_*_16_ | 0 | 0.00 | 0.00 | 0.00 | 0.00 | 0.03 | 0.02 | 0.01 | 0.01 | 0.03 | 0.02 | 0.01 | 0.01 |
| γ*_g_*_8×E_ | γ*_b_*_16_ | 0 | 0.00 | 0.00 | 0.00 | 0.00 | 0.03 | 0.02 | 0.01 | 0.01 | 0.03 | 0.02 | 0.01 | 0.01 |
| γ*_g_*_9×E_ | γ*_b_*_16_ | 0 | 0.00 | 0.00 | 0.00 | 0.00 | 0.03 | 0.02 | 0.01 | 0.01 | 0.03 | 0.02 | 0.01 | 0.01 |
| γ*_g_*_1_ | γ*_b_*_17_ | 0.6 | 0.00 | 0.00 | 0.00 | 0.00 | 0.03 | 0.02 | 0.01 | 0.01 | 0.03 | 0.02 | 0.01 | 0.01 |
| γ*_g_*_2_ | γ*_b_*_17_ | 0.4 | -0.01 | 0.00 | 0.00 | 0.00 | 0.05 | 0.03 | 0.02 | 0.02 | 0.05 | 0.03 | 0.02 | 0.02 |
| γ*_g_*_3_ | γ*_b_*_17_ | 0 | 0.05 | 0.02 | 0.01 | 0.01 | 0.04 | 0.05 | 0.04 | 0.04 | 0.06 | 0.05 | 0.05 | 0.04 |
| γ*_g_*_4_ | γ*_b_*_17_ | 0 | -0.04 | -0.02 | -0.01 | -0.01 | 0.05 | 0.04 | 0.04 | 0.03 | 0.06 | 0.05 | 0.04 | 0.03 |
| γ*_g_*_5_ | γ*_b_*_17_ | 0 | 0.01 | 0.00 | 0.00 | 0.00 | 0.04 | 0.03 | 0.02 | 0.02 | 0.04 | 0.03 | 0.02 | 0.02 |
| γ*_g_*_6_ | γ*_b_*_17_ | 0 | 0.00 | 0.00 | 0.00 | 0.00 | 0.04 | 0.03 | 0.02 | 0.02 | 0.04 | 0.03 | 0.02 | 0.02 |
| γ*_g_*_7_ | γ*_b_*_17_ | 0 | 0.00 | 0.00 | 0.00 | 0.00 | 0.05 | 0.03 | 0.02 | 0.02 | 0.05 | 0.03 | 0.02 | 0.02 |
| γ*_g_*_8_ | γ*_b_*_17_ | 0 | 0.00 | 0.00 | 0.00 | 0.00 | 0.04 | 0.03 | 0.02 | 0.02 | 0.04 | 0.03 | 0.02 | 0.02 |
| γ*_g_*_9_ | γ*_b_*_17_ | 0 | 0.00 | 0.00 | 0.00 | 0.00 | 0.05 | 0.03 | 0.02 | 0.02 | 0.05 | 0.03 | 0.02 | 0.02 |
| z_E_ | γ*_b_*_17_ | 0.2 | 0.00 | 0.00 | 0.00 | 0.00 | 0.05 | 0.03 | 0.02 | 0.02 | 0.05 | 0.03 | 0.02 | 0.02 |
| γ*_g_*_1×E_ | γ*_b_*_17_ | -0.4 | 0.00 | 0.00 | 0.00 | 0.00 | 0.05 | 0.04 | 0.02 | 0.02 | 0.05 | 0.04 | 0.02 | 0.02 |
| γ*_g_*_2×E_ | γ*_b_*_17_ | 0 | 0.00 | 0.00 | 0.00 | 0.00 | 0.05 | 0.03 | 0.02 | 0.02 | 0.05 | 0.03 | 0.02 | 0.02 |
| γ*_g_*_3×E_ | γ*_b_*_17_ | 0 | -0.02 | -0.01 | -0.01 | 0.00 | 0.05 | 0.04 | 0.03 | 0.02 | 0.05 | 0.04 | 0.03 | 0.02 |
| γ*_g_*_4×E_ | γ*_b_*_17_ | 0 | 0.03 | 0.02 | 0.01 | 0.00 | 0.05 | 0.04 | 0.04 | 0.03 | 0.06 | 0.05 | 0.04 | 0.03 |
| γ*_g_*_5×E_ | γ*_b_*_17_ | 0 | -0.01 | 0.00 | -0.01 | 0.00 | 0.05 | 0.03 | 0.02 | 0.02 | 0.05 | 0.03 | 0.03 | 0.02 |
| γ*_g_*_6×E_ | γ*_b_*_17_ | 0 | 0.00 | 0.00 | 0.00 | 0.00 | 0.05 | 0.03 | 0.02 | 0.02 | 0.05 | 0.03 | 0.02 | 0.02 |
| γ*_g_*_7×E_ | γ*_b_*_17_ | 0 | 0.00 | 0.00 | 0.00 | 0.00 | 0.05 | 0.03 | 0.02 | 0.02 | 0.05 | 0.03 | 0.02 | 0.02 |
| γ*_g_*_8×E_ | γ*_b_*_17_ | 0 | 0.00 | 0.00 | 0.00 | 0.00 | 0.05 | 0.03 | 0.02 | 0.02 | 0.05 | 0.03 | 0.02 | 0.02 |
| γ*_g_*_9×E_ | γ*_b_*_17_ | 0 | 0.00 | 0.00 | 0.00 | 0.00 | 0.05 | 0.04 | 0.02 | 0.02 | 0.05 | 0.04 | 0.02 | 0.02 |
| γ*_g_*_1_ | γ*_b_*_18_ | 0 | 0.00 | 0.00 | 0.00 | 0.00 | 0.05 | 0.03 | 0.02 | 0.02 | 0.05 | 0.03 | 0.02 | 0.02 |
| γ*_g_*_2_ | γ*_b_*_18_ | -0.6 | -0.04 | -0.02 | -0.01 | -0.01 | 0.05 | 0.04 | 0.03 | 0.03 | 0.06 | 0.04 | 0.03 | 0.03 |
| γ*_g_*_3_ | γ*_b_*_18_ | 0.4 | 0.00 | 0.00 | 0.00 | 0.00 | 0.05 | 0.04 | 0.03 | 0.02 | 0.05 | 0.04 | 0.03 | 0.02 |
| γ*_g_*_4_ | γ*_b_*_18_ | 0 | 0.05 | 0.02 | 0.01 | 0.01 | 0.04 | 0.03 | 0.02 | 0.04 | 0.06 | 0.04 | 0.02 | 0.04 |
| γ*_g_*_5_ | γ*_b_*_18_ | 0 | 0.00 | 0.00 | 0.00 | 0.00 | 0.05 | 0.04 | 0.03 | 0.02 | 0.05 | 0.04 | 0.03 | 0.02 |
| γ*_g_*_6_ | γ*_b_*_18_ | 0 | 0.00 | 0.00 | 0.00 | 0.00 | 0.05 | 0.04 | 0.03 | 0.02 | 0.05 | 0.04 | 0.03 | 0.02 |
| γ*_g_*_7_ | γ*_b_*_18_ | 0 | 0.01 | 0.01 | 0.01 | 0.00 | 0.05 | 0.04 | 0.03 | 0.02 | 0.05 | 0.04 | 0.03 | 0.02 |
| γ*_g_*_8_ | γ*_b_*_18_ | 0 | 0.00 | 0.00 | 0.00 | 0.00 | 0.05 | 0.04 | 0.03 | 0.02 | 0.05 | 0.04 | 0.03 | 0.02 |
| γ*_g_*_9_ | γ*_b_*_18_ | 0 | 0.00 | 0.00 | 0.00 | 0.00 | 0.05 | 0.04 | 0.03 | 0.02 | 0.05 | 0.04 | 0.03 | 0.02 |
| z_E_ | γ*_b_*_18_ | 0 | 0.00 | 0.00 | 0.00 | 0.00 | 0.05 | 0.04 | 0.03 | 0.02 | 0.05 | 0.04 | 0.03 | 0.02 |
| γ*_g_*_1×E_ | γ*_b_*_18_ | 0 | 0.00 | 0.00 | 0.00 | 0.00 | 0.05 | 0.03 | 0.03 | 0.02 | 0.05 | 0.03 | 0.03 | 0.02 |
| γ*_g_*_2×E_ | γ*_b_*_18_ | 0.2 | -0.02 | -0.01 | -0.01 | -0.01 | 0.05 | 0.04 | 0.03 | 0.02 | 0.05 | 0.04 | 0.03 | 0.02 |
| γ*_g_*_3×E_ | γ*_b_*_18_ | -0.4 | 0.01 | 0.00 | 0.00 | 0.00 | 0.05 | 0.04 | 0.03 | 0.02 | 0.05 | 0.04 | 0.03 | 0.02 |
| γ*_g_*_4×E_ | γ*_b_*_18_ | 0 | 0.00 | 0.00 | 0.00 | 0.00 | 0.05 | 0.04 | 0.03 | 0.02 | 0.05 | 0.04 | 0.03 | 0.02 |
| γ*_g_*_5×E_ | γ*_b_*_18_ | 0 | 0.01 | 0.01 | 0.01 | 0.01 | 0.05 | 0.04 | 0.03 | 0.02 | 0.05 | 0.04 | 0.03 | 0.02 |
| γ*_g_*_6×E_ | γ*_b_*_18_ | 0 | 0.00 | 0.00 | 0.00 | 0.00 | 0.05 | 0.04 | 0.03 | 0.02 | 0.05 | 0.04 | 0.03 | 0.02 |
| γ*_g_*_7×E_ | γ*_b_*_18_ | 0 | 0.00 | 0.00 | 0.00 | 0.00 | 0.05 | 0.04 | 0.03 | 0.02 | 0.05 | 0.04 | 0.03 | 0.02 |
| γ*_g_*_8×E_ | γ*_b_*_18_ | 0 | 0.00 | 0.00 | 0.00 | 0.00 | 0.05 | 0.04 | 0.03 | 0.02 | 0.05 | 0.04 | 0.03 | 0.02 |
| γ*_g_*_9×E_ | γ*_b_*_18_ | 0 | 0.00 | 0.01 | 0.00 | 0.00 | 0.05 | 0.04 | 0.03 | 0.02 | 0.05 | 0.04 | 0.03 | 0.02 |
| γ*_g_*_1_ | γ*_b_*_19_ | 0.4 | 0.00 | 0.00 | 0.00 | 0.00 | 0.05 | 0.04 | 0.03 | 0.02 | 0.05 | 0.04 | 0.03 | 0.02 |
| γ*_g_*_2_ | γ*_b_*_19_ | 0 | 0.04 | 0.02 | 0.01 | 0.00 | 0.04 | 0.05 | 0.04 | 0.04 | 0.06 | 0.05 | 0.04 | 0.04 |
| γ*_g_*_3_ | γ*_b_*_19_ | -0.6 | -0.01 | -0.01 | 0.00 | 0.00 | 0.04 | 0.03 | 0.02 | 0.02 | 0.04 | 0.03 | 0.02 | 0.02 |
| γ*_g_*_4_ | γ*_b_*_19_ | 0 | -0.01 | 0.00 | 0.00 | 0.00 | 0.04 | 0.03 | 0.02 | 0.02 | 0.05 | 0.03 | 0.02 | 0.02 |
| γ*_g_*_5_ | γ*_b_*_19_ | 0 | 0.00 | 0.00 | 0.00 | 0.00 | 0.04 | 0.03 | 0.02 | 0.02 | 0.04 | 0.03 | 0.02 | 0.02 |
| γ*_g_*_6_ | γ*_b_*_19_ | 0 | 0.00 | 0.00 | 0.00 | 0.00 | 0.04 | 0.03 | 0.02 | 0.02 | 0.04 | 0.03 | 0.02 | 0.02 |
| γ*_g_*_7_ | γ*_b_*_19_ | 0 | 0.00 | 0.00 | 0.00 | 0.00 | 0.04 | 0.03 | 0.02 | 0.02 | 0.04 | 0.03 | 0.02 | 0.02 |
| γ*_g_*_8_ | γ*_b_*_19_ | 0 | 0.00 | 0.00 | 0.00 | 0.00 | 0.04 | 0.03 | 0.02 | 0.02 | 0.04 | 0.03 | 0.02 | 0.02 |
| γ*_g_*_9_ | γ*_b_*_19_ | 0 | 0.00 | 0.00 | 0.00 | 0.00 | 0.04 | 0.03 | 0.02 | 0.02 | 0.04 | 0.03 | 0.02 | 0.02 |
| z_E_ | γ*_b_*_19_ | 0 | -0.01 | -0.01 | -0.01 | -0.01 | 0.04 | 0.03 | 0.02 | 0.02 | 0.04 | 0.03 | 0.02 | 0.02 |
| γ*_g_*_1×E_ | γ*_b_*_19_ | 0.2 | -0.02 | -0.01 | -0.01 | -0.01 | 0.05 | 0.04 | 0.03 | 0.02 | 0.05 | 0.04 | 0.03 | 0.02 |
| γ*_g_*_2×E_ | γ*_b_*_19_ | 0 | -0.03 | -0.02 | -0.01 | -0.01 | 0.05 | 0.04 | 0.03 | 0.03 | 0.05 | 0.04 | 0.04 | 0.03 |
| γ*_g_*_3×E_ | γ*_b_*_19_ | 0 | 0.01 | 0.00 | 0.00 | 0.00 | 0.05 | 0.03 | 0.02 | 0.02 | 0.05 | 0.03 | 0.02 | 0.02 |
| γ*_g_*_4×E_ | γ*_b_*_19_ | 0 | 0.00 | 0.00 | 0.00 | 0.00 | 0.05 | 0.03 | 0.02 | 0.02 | 0.05 | 0.03 | 0.02 | 0.02 |
| γ*_g_*_5×E_ | γ*_b_*_19_ | 0 | 0.00 | 0.00 | 0.00 | 0.00 | 0.04 | 0.03 | 0.02 | 0.02 | 0.04 | 0.03 | 0.02 | 0.02 |
| γ*_g_*_6×E_ | γ*_b_*_19_ | 0 | 0.01 | 0.01 | 0.01 | 0.01 | 0.05 | 0.03 | 0.02 | 0.02 | 0.05 | 0.03 | 0.02 | 0.02 |
| γ*_g_*_7×E_ | γ*_b_*_19_ | 0 | 0.00 | 0.00 | 0.00 | 0.00 | 0.05 | 0.03 | 0.02 | 0.02 | 0.05 | 0.03 | 0.02 | 0.02 |
| γ*_g_*_8×E_ | γ*_b_*_19_ | 0 | 0.00 | 0.00 | 0.00 | 0.00 | 0.05 | 0.03 | 0.02 | 0.02 | 0.05 | 0.03 | 0.02 | 0.02 |
| γ*_g_*_9×E_ | γ*_b_*_19_ | 0 | 0.00 | 0.00 | 0.00 | 0.00 | 0.04 | 0.03 | 0.02 | 0.02 | 0.04 | 0.03 | 0.02 | 0.02 |
| γ*_g_*_1_ | γ*_b_*_20_ | -0.6 | 0.00 | 0.00 | 0.00 | 0.00 | 0.05 | 0.03 | 0.02 | 0.02 | 0.05 | 0.03 | 0.02 | 0.02 |
| γ*_g_*_2_ | γ*_b_*_20_ | 0 | -0.04 | -0.02 | -0.01 | 0.00 | 0.03 | 0.02 | 0.02 | 0.01 | 0.05 | 0.03 | 0.02 | 0.01 |
| γ*_g_*_3_ | γ*_b_*_20_ | 0 | -0.02 | -0.01 | 0.00 | 0.00 | 0.03 | 0.02 | 0.02 | 0.01 | 0.03 | 0.02 | 0.02 | 0.01 |
| γ*_g_*_4_ | γ*_b_*_20_ | 0 | 0.02 | 0.01 | 0.00 | 0.00 | 0.03 | 0.02 | 0.01 | 0.01 | 0.03 | 0.02 | 0.01 | 0.01 |
| γ*_g_*_5_ | γ*_b_*_20_ | 0 | 0.00 | 0.00 | 0.00 | 0.00 | 0.03 | 0.02 | 0.01 | 0.01 | 0.03 | 0.02 | 0.01 | 0.01 |
| γ*_g_*_6_ | γ*_b_*_20_ | 0 | 0.00 | 0.00 | 0.00 | 0.00 | 0.03 | 0.02 | 0.01 | 0.01 | 0.03 | 0.02 | 0.01 | 0.01 |
| γ*_g_*_7_ | γ*_b_*_20_ | 0 | 0.00 | 0.00 | 0.00 | 0.00 | 0.03 | 0.02 | 0.01 | 0.01 | 0.03 | 0.02 | 0.01 | 0.01 |
| γ*_g_*_8_ | γ*_b_*_20_ | 0 | 0.00 | 0.00 | 0.00 | 0.00 | 0.03 | 0.02 | 0.01 | 0.01 | 0.03 | 0.02 | 0.01 | 0.01 |
| γ*_g_*_9_ | γ*_b_*_20_ | 0 | 0.00 | 0.00 | 0.00 | 0.00 | 0.03 | 0.02 | 0.01 | 0.01 | 0.03 | 0.02 | 0.01 | 0.01 |
| z_E_ | γ*_b_*_20_ | 0.2 | 0.00 | 0.00 | 0.00 | 0.00 | 0.03 | 0.02 | 0.01 | 0.01 | 0.03 | 0.02 | 0.01 | 0.01 |
| γ*_g_*_1×E_ | γ*_b_*_20_ | 0.4 | -0.02 | -0.01 | -0.01 | 0.00 | 0.03 | 0.02 | 0.02 | 0.01 | 0.04 | 0.03 | 0.02 | 0.01 |
| γ*_g_*_2×E_ | γ*_b_*_20_ | 0 | 0.03 | 0.02 | 0.01 | 0.01 | 0.03 | 0.02 | 0.02 | 0.01 | 0.04 | 0.03 | 0.02 | 0.01 |
| γ*_g_*_3×E_ | γ*_b_*_20_ | 0 | -0.01 | 0.00 | 0.00 | 0.00 | 0.03 | 0.02 | 0.01 | 0.01 | 0.03 | 0.02 | 0.01 | 0.01 |
| γ*_g_*_4×E_ | γ*_b_*_20_ | 0 | -0.01 | 0.00 | 0.00 | 0.00 | 0.03 | 0.02 | 0.01 | 0.01 | 0.03 | 0.02 | 0.01 | 0.01 |
| γ*_g_*_5×E_ | γ*_b_*_20_ | 0 | 0.00 | 0.00 | 0.00 | 0.00 | 0.03 | 0.02 | 0.01 | 0.01 | 0.03 | 0.02 | 0.01 | 0.01 |
| γ*_g_*_6×E_ | γ*_b_*_20_ | 0 | 0.00 | 0.00 | 0.00 | 0.00 | 0.03 | 0.02 | 0.01 | 0.01 | 0.03 | 0.02 | 0.01 | 0.01 |
| γ*_g_*_7×E_ | γ*_b_*_20_ | 0 | 0.00 | 0.00 | 0.00 | 0.00 | 0.03 | 0.02 | 0.01 | 0.01 | 0.03 | 0.02 | 0.01 | 0.01 |
| γ*_g_*_8×E_ | γ*_b_*_20_ | 0 | 0.00 | 0.00 | 0.00 | 0.00 | 0.03 | 0.02 | 0.01 | 0.01 | 0.03 | 0.02 | 0.01 | 0.01 |
| γ*_g_*_9×E_ | γ*_b_*_20_ | 0 | 0.00 | 0.00 | 0.00 | 0.00 | 0.03 | 0.02 | 0.01 | 0.01 | 0.03 | 0.02 | 0.01 | 0.01 |
| γ*_g_*_1_ | γ*_b_*_21_ | 0 | 0.00 | 0.00 | 0.00 | 0.00 | 0.03 | 0.02 | 0.01 | 0.01 | 0.03 | 0.02 | 0.01 | 0.01 |
| γ*_g_*_2_ | γ*_b_*_21_ | 0 | 0.00 | 0.00 | 0.00 | 0.00 | 0.05 | 0.03 | 0.02 | 0.02 | 0.05 | 0.03 | 0.02 | 0.02 |
| γ*_g_*_3_ | γ*_b_*_21_ | 0 | 0.05 | 0.02 | 0.01 | 0.00 | 0.07 | 0.05 | 0.02 | 0.02 | 0.09 | 0.05 | 0.02 | 0.02 |
| γ*_g_*_4_ | γ*_b_*_21_ | 0.6 | -0.04 | -0.02 | -0.01 | -0.01 | 0.06 | 0.04 | 0.03 | 0.02 | 0.07 | 0.05 | 0.03 | 0.02 |
| γ*_g_*_5_ | γ*_b_*_21_ | 0.4 | 0.00 | 0.00 | -0.01 | 0.00 | 0.05 | 0.03 | 0.02 | 0.02 | 0.05 | 0.03 | 0.02 | 0.02 |
| γ*_g_*_6_ | γ*_b_*_21_ | 0 | 0.00 | 0.00 | 0.00 | 0.00 | 0.05 | 0.03 | 0.03 | 0.02 | 0.05 | 0.03 | 0.03 | 0.02 |
| γ*_g_*_7_ | γ*_b_*_21_ | 0 | 0.00 | 0.00 | 0.00 | 0.00 | 0.05 | 0.03 | 0.02 | 0.02 | 0.05 | 0.03 | 0.02 | 0.02 |
| γ*_g_*_8_ | γ*_b_*_21_ | 0 | 0.00 | 0.00 | 0.00 | 0.00 | 0.04 | 0.03 | 0.02 | 0.02 | 0.04 | 0.03 | 0.02 | 0.02 |
| γ*_g_*_9_ | γ*_b_*_21_ | 0 | 0.00 | 0.00 | 0.00 | 0.00 | 0.05 | 0.03 | 0.02 | 0.02 | 0.05 | 0.03 | 0.02 | 0.02 |
| z_E_ | γ*_b_*_21_ | 0.2 | 0.00 | 0.00 | 0.00 | 0.00 | 0.05 | 0.04 | 0.02 | 0.02 | 0.05 | 0.04 | 0.02 | 0.02 |
| γ*_g_*_1×E_ | γ*_b_*_21_ | 0 | 0.00 | 0.00 | 0.00 | 0.00 | 0.05 | 0.04 | 0.02 | 0.02 | 0.05 | 0.04 | 0.03 | 0.02 |
| γ*_g_*_2×E_ | γ*_b_*_21_ | 0 | 0.00 | 0.00 | 0.00 | 0.01 | 0.05 | 0.04 | 0.02 | 0.02 | 0.05 | 0.04 | 0.02 | 0.02 |
| γ*_g_*_3×E_ | γ*_b_*_21_ | 0 | -0.03 | -0.01 | -0.01 | 0.00 | 0.05 | 0.04 | 0.02 | 0.02 | 0.06 | 0.04 | 0.02 | 0.02 |
| γ*_g_*_4×E_ | γ*_b_*_21_ | -0.4 | 0.04 | 0.02 | 0.01 | 0.01 | 0.06 | 0.04 | 0.03 | 0.02 | 0.07 | 0.05 | 0.03 | 0.02 |
| γ*_g_*_5×E_ | γ*_b_*_21_ | 0 | 0.00 | 0.00 | 0.00 | 0.00 | 0.05 | 0.03 | 0.02 | 0.02 | 0.05 | 0.03 | 0.02 | 0.02 |
| γ*_g_*_6×E_ | γ*_b_*_21_ | 0 | 0.00 | 0.00 | 0.00 | 0.00 | 0.05 | 0.03 | 0.02 | 0.02 | 0.05 | 0.03 | 0.02 | 0.02 |
| γ*_g_*_7×E_ | γ*_b_*_21_ | 0 | 0.00 | 0.00 | 0.00 | 0.00 | 0.05 | 0.03 | 0.02 | 0.02 | 0.05 | 0.03 | 0.02 | 0.02 |
| γ*_g_*_8×E_ | γ*_b_*_21_ | 0 | 0.00 | 0.00 | 0.00 | 0.00 | 0.05 | 0.03 | 0.02 | 0.02 | 0.05 | 0.03 | 0.02 | 0.02 |
| γ*_g_*_9×E_ | γ*_b_*_21_ | 0 | 0.00 | 0.00 | 0.00 | 0.00 | 0.05 | 0.03 | 0.02 | 0.02 | 0.05 | 0.03 | 0.02 | 0.02 |
| γ*_g_*_1_ | γ*_b_*_22_ | 0 | 0.00 | 0.00 | 0.00 | 0.00 | 0.05 | 0.03 | 0.02 | 0.02 | 0.05 | 0.03 | 0.02 | 0.02 |
| γ*_g_*_2_ | γ*_b_*_22_ | 0 | -0.04 | -0.02 | -0.01 | -0.01 | 0.05 | 0.04 | 0.02 | 0.02 | 0.06 | 0.04 | 0.03 | 0.02 |
| γ*_g_*_3_ | γ*_b_*_22_ | 0 | -0.01 | 0.00 | 0.00 | 0.00 | 0.05 | 0.04 | 0.03 | 0.02 | 0.05 | 0.04 | 0.03 | 0.02 |
| γ*_g_*_4_ | γ*_b_*_22_ | 0 | 0.05 | 0.03 | 0.02 | 0.01 | 0.04 | 0.03 | 0.02 | 0.02 | 0.07 | 0.04 | 0.03 | 0.02 |
| γ*_g_*_5_ | γ*_b_*_22_ | -0.6 | 0.00 | 0.00 | 0.00 | 0.00 | 0.05 | 0.04 | 0.03 | 0.02 | 0.05 | 0.04 | 0.03 | 0.02 |
| γ*_g_*_6_ | γ*_b_*_22_ | 0.4 | -0.01 | 0.00 | 0.00 | 0.00 | 0.05 | 0.04 | 0.03 | 0.02 | 0.05 | 0.04 | 0.03 | 0.02 |
| γ*_g_*_7_ | γ*_b_*_22_ | 0 | -0.01 | 0.00 | 0.00 | 0.00 | 0.05 | 0.04 | 0.03 | 0.02 | 0.05 | 0.04 | 0.03 | 0.02 |
| γ*_g_*_8_ | γ*_b_*_22_ | 0 | 0.00 | 0.00 | 0.00 | 0.01 | 0.05 | 0.04 | 0.03 | 0.02 | 0.05 | 0.04 | 0.03 | 0.02 |
| γ*_g_*_9_ | γ*_b_*_22_ | 0 | 0.00 | 0.00 | 0.00 | 0.00 | 0.05 | 0.04 | 0.03 | 0.02 | 0.05 | 0.04 | 0.03 | 0.02 |
| z_E_ | γ*_b_*_22_ | 0 | 0.00 | 0.00 | 0.00 | 0.00 | 0.05 | 0.04 | 0.03 | 0.02 | 0.05 | 0.04 | 0.03 | 0.02 |
| γ*_g_*_1×E_ | γ*_b_*_22_ | 0 | 0.00 | 0.00 | 0.00 | 0.00 | 0.05 | 0.04 | 0.03 | 0.02 | 0.05 | 0.04 | 0.03 | 0.02 |
| γ*_g_*_2×E_ | γ*_b_*_22_ | 0 | -0.02 | -0.01 | 0.00 | 0.00 | 0.05 | 0.04 | 0.03 | 0.02 | 0.05 | 0.04 | 0.03 | 0.02 |
| γ*_g_*_3×E_ | γ*_b_*_22_ | 0 | 0.01 | 0.00 | 0.00 | 0.00 | 0.05 | 0.04 | 0.03 | 0.02 | 0.05 | 0.04 | 0.03 | 0.02 |
| γ*_g_*_4×E_ | γ*_b_*_22_ | 0 | 0.00 | 0.00 | 0.00 | 0.00 | 0.05 | 0.04 | 0.03 | 0.02 | 0.05 | 0.04 | 0.03 | 0.02 |
| γ*_g_*_5×E_ | γ*_b_*_22_ | 0.2 | 0.01 | 0.00 | 0.01 | 0.00 | 0.05 | 0.04 | 0.03 | 0.02 | 0.05 | 0.04 | 0.03 | 0.02 |
| γ*_g_*_6×E_ | γ*_b_*_22_ | -0.4 | 0.00 | 0.00 | 0.00 | 0.00 | 0.05 | 0.04 | 0.03 | 0.02 | 0.05 | 0.04 | 0.03 | 0.02 |
| γ*_g_*_7×E_ | γ*_b_*_22_ | 0 | 0.00 | 0.00 | 0.00 | 0.00 | 0.05 | 0.04 | 0.03 | 0.02 | 0.05 | 0.04 | 0.03 | 0.02 |
| γ*_g_*_8×E_ | γ*_b_*_22_ | 0 | 0.00 | 0.00 | 0.00 | 0.00 | 0.05 | 0.04 | 0.03 | 0.02 | 0.05 | 0.04 | 0.03 | 0.02 |
| γ*_g_*_9×E_ | γ*_b_*_22_ | 0 | 0.00 | 0.00 | 0.00 | 0.00 | 0.05 | 0.04 | 0.03 | 0.02 | 0.05 | 0.04 | 0.03 | 0.02 |
| γ*_g_*_1_ | γ*_b_*_23_ | 0 | 0.00 | 0.00 | 0.00 | 0.00 | 0.05 | 0.04 | 0.03 | 0.02 | 0.05 | 0.04 | 0.03 | 0.02 |
| γ*_g_*_2_ | γ*_b_*_23_ | 0 | 0.04 | 0.02 | 0.01 | 0.01 | 0.04 | 0.03 | 0.02 | 0.04 | 0.06 | 0.04 | 0.02 | 0.04 |
| γ*_g_*_3_ | γ*_b_*_23_ | 0 | -0.01 | 0.00 | 0.00 | 0.00 | 0.04 | 0.03 | 0.02 | 0.02 | 0.05 | 0.03 | 0.02 | 0.02 |
| γ*_g_*_4_ | γ*_b_*_23_ | 0.4 | -0.01 | 0.00 | 0.00 | 0.00 | 0.05 | 0.03 | 0.02 | 0.02 | 0.05 | 0.03 | 0.02 | 0.02 |
| γ*_g_*_5_ | γ*_b_*_23_ | 0 | 0.00 | 0.00 | 0.00 | 0.00 | 0.04 | 0.03 | 0.02 | 0.02 | 0.04 | 0.03 | 0.02 | 0.02 |
| γ*_g_*_6_ | γ*_b_*_23_ | -0.6 | 0.00 | 0.00 | 0.00 | 0.00 | 0.04 | 0.03 | 0.02 | 0.02 | 0.04 | 0.03 | 0.02 | 0.02 |
| γ*_g_*_7_ | γ*_b_*_23_ | 0 | 0.00 | 0.00 | 0.00 | 0.00 | 0.04 | 0.03 | 0.02 | 0.02 | 0.05 | 0.03 | 0.02 | 0.02 |
| γ*_g_*_8_ | γ*_b_*_23_ | 0 | 0.00 | 0.00 | 0.00 | 0.00 | 0.04 | 0.03 | 0.02 | 0.02 | 0.04 | 0.03 | 0.02 | 0.02 |
| γ*_g_*_9_ | γ*_b_*_23_ | 0 | 0.00 | 0.00 | 0.00 | 0.00 | 0.04 | 0.03 | 0.02 | 0.02 | 0.04 | 0.03 | 0.02 | 0.02 |
| z_E_ | γ*_b_*_23_ | 0 | 0.00 | 0.00 | 0.00 | 0.00 | 0.04 | 0.03 | 0.02 | 0.02 | 0.04 | 0.03 | 0.02 | 0.02 |
| γ*_g_*_1×E_ | γ*_b_*_23_ | 0 | -0.01 | -0.01 | 0.00 | 0.00 | 0.05 | 0.03 | 0.03 | 0.02 | 0.05 | 0.03 | 0.03 | 0.02 |
| γ*_g_*_2×E_ | γ*_b_*_23_ | 0 | -0.04 | -0.02 | -0.01 | -0.01 | 0.03 | 0.02 | 0.04 | 0.01 | 0.05 | 0.03 | 0.04 | 0.01 |
| γ*_g_*_3×E_ | γ*_b_*_23_ | 0 | -0.02 | -0.01 | 0.00 | 0.00 | 0.03 | 0.02 | 0.03 | 0.01 | 0.04 | 0.02 | 0.03 | 0.01 |
| γ*_g_*_4×E_ | γ*_b_*_23_ | 0.2 | 0.01 | 0.01 | 0.00 | 0.00 | 0.03 | 0.02 | 0.01 | 0.01 | 0.03 | 0.02 | 0.01 | 0.01 |
| γ*_g_*_5×E_ | γ*_b_*_23_ | 0 | 0.00 | 0.00 | 0.00 | 0.00 | 0.03 | 0.02 | 0.01 | 0.01 | 0.03 | 0.02 | 0.01 | 0.01 |
| γ*_g_*_6×E_ | γ*_b_*_23_ | 0 | 0.00 | 0.00 | 0.00 | 0.00 | 0.03 | 0.02 | 0.01 | 0.01 | 0.03 | 0.02 | 0.01 | 0.01 |
| γ*_g_*_7×E_ | γ*_b_*_23_ | 0 | 0.00 | 0.00 | 0.00 | 0.00 | 0.03 | 0.02 | 0.01 | 0.01 | 0.03 | 0.02 | 0.01 | 0.01 |
| γ*_g_*_8×E_ | γ*_b_*_23_ | 0 | 0.00 | 0.00 | 0.00 | 0.00 | 0.03 | 0.02 | 0.01 | 0.01 | 0.03 | 0.02 | 0.01 | 0.01 |
| γ*_g_*_9×E_ | γ*_b_*_23_ | 0 | 0.00 | 0.00 | 0.00 | 0.00 | 0.03 | 0.02 | 0.01 | 0.01 | 0.03 | 0.02 | 0.01 | 0.01 |
| γ*_g_*_1_ | γ*_b_*_24_ | 0 |  | 0.00 | 0.00 | 0.00 | 0.03 | 0.02 | 0.01 | 0.01 | 0.03 | 0.02 | 0.01 | 0.01 |
| γ*_g_*_2_ | γ*_b_*_24_ | 0 | -0.02 | -0.01 | -0.01 | 0.00 | 0.03 | 0.02 | 0.02 | 0.01 | 0.04 | 0.03 | 0.02 | 0.01 |
| γ*_g_*_3_ | γ*_b_*_24_ | 0 | 0.03 | 0.01 | 0.01 | 0.00 | 0.03 | 0.02 | 0.03 | 0.01 | 0.04 | 0.03 | 0.03 | 0.01 |
| γ*_g_*_4_ | γ*_b_*_24_ | -0.6 | -0.01 | 0.00 | 0.00 | 0.00 | 0.03 | 0.02 | 0.01 | 0.01 | 0.03 | 0.02 | 0.01 | 0.01 |
| γ*_g_*_5_ | γ*_b_*_24_ | 0 | -0.01 | 0.00 | 0.00 | 0.00 | 0.03 | 0.02 | 0.01 | 0.01 | 0.03 | 0.02 | 0.01 | 0.01 |
| γ*_g_*_6_ | γ*_b_*_24_ | 0 | 0.00 | 0.00 | 0.00 | 0.00 | 0.03 | 0.02 | 0.01 | 0.01 | 0.03 | 0.02 | 0.01 | 0.01 |
| γ*_g_*_7_ | γ*_b_*_24_ | 0 | 0.00 | 0.00 | 0.00 | 0.00 | 0.03 | 0.02 | 0.01 | 0.01 | 0.03 | 0.02 | 0.01 | 0.01 |
| γ*_g_*_8_ | γ*_b_*_24_ | 0 | 0.00 | 0.00 | 0.00 | 0.00 | 0.03 | 0.02 | 0.01 | 0.01 | 0.03 | 0.02 | 0.01 | 0.01 |
| γ*_g_*_9_ | γ*_b_*_24_ | 0 | 0.00 | 0.00 | 0.00 | 0.00 | 0.03 | 0.02 | 0.01 | 0.01 | 0.03 | 0.02 | 0.01 | 0.01 |
| z_E_ | γ*_b_*_24_ | 0.2 | 0.00 | 0.00 | 0.00 | 0.00 | 0.03 | 0.02 | 0.01 | 0.01 | 0.03 | 0.02 | 0.01 | 0.01 |
| γ*_g_*_1×E_ | γ*_b_*_24_ | 0 | 0.00 | 0.00 | 0.00 | 0.00 | 0.03 | 0.02 | 0.01 | 0.01 | 0.03 | 0.02 | 0.01 | 0.01 |
| γ*_g_*_2×E_ | γ*_b_*_24_ | 0 | -0.01 | 0.00 | 0.00 | 0.00 | 0.05 | 0.03 | 0.02 | 0.02 | 0.05 | 0.03 | 0.02 | 0.02 |
| γ*_g_*_3×E_ | γ*_b_*_24_ | 0 | 0.05 | 0.02 | 0.01 | 0.01 | 0.04 | 0.05 | 0.04 | 0.04 | 0.06 | 0.05 | 0.05 | 0.04 |
| γ*_g_*_4×E_ | γ*_b_*_24_ | 0.4 | -0.04 | -0.02 | -0.01 | -0.01 | 0.05 | 0.04 | 0.04 | 0.03 | 0.06 | 0.05 | 0.04 | 0.03 |
| γ*_g_*_5×E_ | γ*_b_*_24_ | 0 | 0.01 | 0.00 | 0.00 | 0.00 | 0.04 | 0.03 | 0.02 | 0.02 | 0.04 | 0.03 | 0.02 | 0.02 |
| γ*_g_*_6×E_ | γ*_b_*_24_ | 0 | 0.00 | 0.00 | 0.00 | 0.00 | 0.04 | 0.03 | 0.02 | 0.02 | 0.04 | 0.03 | 0.02 | 0.02 |
| γ*_g_*_7×E_ | γ*_b_*_24_ | 0 | 0.00 | 0.00 | 0.00 | 0.00 | 0.05 | 0.03 | 0.02 | 0.02 | 0.05 | 0.03 | 0.02 | 0.02 |
| γ*_g_*_8×E_ | γ*_b_*_24_ | 0 | 0.00 | 0.00 | 0.00 | 0.00 | 0.04 | 0.03 | 0.02 | 0.02 | 0.04 | 0.03 | 0.02 | 0.02 |
| γ*_g_*_9×E_ | γ*_b_*_24_ | 0 | 0.00 | 0.00 | 0.00 | 0.00 | 0.05 | 0.03 | 0.02 | 0.02 | 0.05 | 0.03 | 0.02 | 0.02 |
| γ*_g_*_1_ | γ*_b_*_25_ | 0 | 0.00 | 0.00 | 0.00 | 0.00 | 0.05 | 0.03 | 0.02 | 0.02 | 0.05 | 0.03 | 0.02 | 0.02 |
| γ*_g_*_2_ | γ*_b_*_25_ | 0 | 0.00 | 0.00 | 0.00 | 0.00 | 0.05 | 0.04 | 0.02 | 0.02 | 0.05 | 0.04 | 0.02 | 0.02 |
| γ*_g_*_3_ | γ*_b_*_25_ | 0 | 0.00 | 0.00 | 0.00 | 0.00 | 0.05 | 0.03 | 0.02 | 0.02 | 0.05 | 0.03 | 0.02 | 0.02 |
| γ*_g_*_4_ | γ*_b_*_25_ | 0.6 | -0.02 | -0.01 | -0.01 | 0.00 | 0.05 | 0.04 | 0.03 | 0.02 | 0.05 | 0.04 | 0.03 | 0.02 |
| γ*_g_*_5_ | γ*_b_*_25_ | 0.4 | 0.03 | 0.02 | 0.01 | 0.00 | 0.05 | 0.04 | 0.04 | 0.03 | 0.06 | 0.05 | 0.04 | 0.03 |
| γ*_g_*_6_ | γ*_b_*_25_ | 0 | -0.01 | 0.00 | -0.01 | 0.00 | 0.05 | 0.03 | 0.02 | 0.02 | 0.05 | 0.03 | 0.03 | 0.02 |
| γ*_g_*_7_ | γ*_b_*_25_ | 0 | 0.00 | 0.00 | 0.00 | 0.00 | 0.05 | 0.03 | 0.02 | 0.02 | 0.05 | 0.03 | 0.02 | 0.02 |
| γ*_g_*_8_ | γ*_b_*_25_ | 0 | 0.00 | 0.00 | 0.00 | 0.00 | 0.05 | 0.03 | 0.02 | 0.02 | 0.05 | 0.03 | 0.02 | 0.02 |
| γ*_g_*_9_ | γ*_b_*_25_ | 0 | 0.00 | 0.00 | 0.00 | 0.00 | 0.05 | 0.03 | 0.02 | 0.02 | 0.05 | 0.03 | 0.02 | 0.02 |
| z_E_ | γ*_b_*_25_ | 0.2 | 0.00 | 0.00 | 0.00 | 0.00 | 0.05 | 0.04 | 0.02 | 0.02 | 0.05 | 0.04 | 0.02 | 0.02 |
| γ*_g_*_1×E_ | γ*_b_*_25_ | 0 | 0.00 | 0.00 | 0.00 | 0.00 | 0.05 | 0.03 | 0.02 | 0.02 | 0.05 | 0.03 | 0.02 | 0.02 |
| γ*_g_*_2×E_ | γ*_b_*_25_ | 0 | -0.04 | -0.02 | -0.01 | -0.01 | 0.05 | 0.04 | 0.03 | 0.03 | 0.06 | 0.04 | 0.03 | 0.03 |
| γ*_g_*_3×E_ | γ*_b_*_25_ | 0 | 0.00 | 0.00 | 0.00 | 0.00 | 0.05 | 0.04 | 0.03 | 0.02 | 0.05 | 0.04 | 0.03 | 0.02 |
| γ*_g_*_4×E_ | γ*_b_*_25_ | -0.4 | 0.05 | 0.02 | 0.01 | 0.01 | 0.04 | 0.03 | 0.02 | 0.04 | 0.06 | 0.04 | 0.02 | 0.04 |
| γ*_g_*_5×E_ | γ*_b_*_25_ | 0 | 0.00 | 0.00 | 0.00 | 0.00 | 0.05 | 0.04 | 0.03 | 0.02 | 0.05 | 0.04 | 0.03 | 0.02 |
| γ*_g_*_6×E_ | γ*_b_*_25_ | 0 | 0.00 | 0.00 | 0.00 | 0.00 | 0.05 | 0.04 | 0.03 | 0.02 | 0.05 | 0.04 | 0.03 | 0.02 |
| γ*_g_*_7×E_ | γ*_b_*_25_ | 0 | 0.01 | 0.01 | 0.01 | 0.00 | 0.05 | 0.04 | 0.03 | 0.02 | 0.05 | 0.04 | 0.03 | 0.02 |
| γ*_g_*_8×E_ | γ*_b_*_25_ | 0 | 0.00 | 0.00 | 0.00 | 0.00 | 0.05 | 0.04 | 0.03 | 0.02 | 0.05 | 0.04 | 0.03 | 0.02 |
| γ*_g_*_9×E_ | γ*_b_*_25_ | 0 | 0.00 | 0.00 | 0.00 | 0.00 | 0.05 | 0.04 | 0.03 | 0.02 | 0.05 | 0.04 | 0.03 | 0.02 |
| γ*_g_*_1_ | γ*_b_*_26_ | 0 | 0.00 | 0.00 | 0.00 | 0.00 | 0.05 | 0.04 | 0.03 | 0.02 | 0.05 | 0.04 | 0.03 | 0.02 |
| γ*_g_*_2_ | γ*_b_*_26_ | 0 | 0.00 | 0.00 | 0.00 | 0.00 | 0.05 | 0.03 | 0.03 | 0.02 | 0.05 | 0.03 | 0.03 | 0.02 |
| γ*_g_*_3_ | γ*_b_*_26_ | 0 | -0.02 | -0.01 | -0.01 | -0.01 | 0.05 | 0.04 | 0.03 | 0.02 | 0.05 | 0.04 | 0.03 | 0.02 |
| γ*_g_*_4_ | γ*_b_*_26_ | 0 | 0.01 | 0.00 | 0.00 | 0.00 | 0.05 | 0.04 | 0.03 | 0.02 | 0.05 | 0.04 | 0.03 | 0.02 |
| γ*_g_*_5_ | γ*_b_*_26_ | -0.6 | 0.00 | 0.00 | 0.00 | 0.00 | 0.05 | 0.04 | 0.03 | 0.02 | 0.05 | 0.04 | 0.03 | 0.02 |
| γ*_g_*_6_ | γ*_b_*_26_ | 0.4 | 0.01 | 0.01 | 0.01 | 0.01 | 0.05 | 0.04 | 0.03 | 0.02 | 0.05 | 0.04 | 0.03 | 0.02 |
| γ*_g_*_7_ | γ*_b_*_26_ | 0 | 0.00 | 0.00 | 0.00 | 0.00 | 0.05 | 0.04 | 0.03 | 0.02 | 0.05 | 0.04 | 0.03 | 0.02 |
| γ*_g_*_8_ | γ*_b_*_26_ | 0 | 0.00 | 0.00 | 0.00 | 0.00 | 0.05 | 0.04 | 0.03 | 0.02 | 0.05 | 0.04 | 0.03 | 0.02 |
| γ*_g_*_9_ | γ*_b_*_26_ | 0 | 0.00 | 0.00 | 0.00 | 0.00 | 0.05 | 0.04 | 0.03 | 0.02 | 0.05 | 0.04 | 0.03 | 0.02 |
| z_E_ | γ*_b_*_26_ | 0 | 0.00 | 0.01 | 0.00 | 0.00 | 0.05 | 0.04 | 0.03 | 0.02 | 0.05 | 0.04 | 0.03 | 0.02 |
| γ*_g_*_1×E_ | γ*_b_*_26_ | 0 | 0.00 | 0.00 | 0.00 | 0.00 | 0.05 | 0.04 | 0.03 | 0.02 | 0.05 | 0.04 | 0.03 | 0.02 |
| γ*_g_*_2×E_ | γ*_b_*_26_ | 0 | 0.04 | 0.02 | 0.01 | 0.00 | 0.04 | 0.05 | 0.04 | 0.04 | 0.06 | 0.05 | 0.04 | 0.04 |
| γ*_g_*_3×E_ | γ*_b_*_26_ | 0 | -0.01 | -0.01 | 0.00 | 0.00 | 0.04 | 0.03 | 0.02 | 0.02 | 0.04 | 0.03 | 0.02 | 0.02 |
| γ*_g_*_4×E_ | γ*_b_*_26_ | 0 | -0.01 | 0.00 | 0.00 | 0.00 | 0.04 | 0.03 | 0.02 | 0.02 | 0.05 | 0.03 | 0.02 | 0.02 |
| γ*_g_*_5×E_ | γ*_b_*_26_ | 0.2 | 0.00 | 0.00 | 0.00 | 0.00 | 0.04 | 0.03 | 0.02 | 0.02 | 0.04 | 0.03 | 0.02 | 0.02 |
| γ*_g_*_6×E_ | γ*_b_*_26_ | -0.4 | 0.00 | 0.00 | 0.00 | 0.00 | 0.04 | 0.03 | 0.02 | 0.02 | 0.04 | 0.03 | 0.02 | 0.02 |
| γ*_g_*_7×E_ | γ*_b_*_26_ | 0 | 0.00 | 0.00 | 0.00 | 0.00 | 0.04 | 0.03 | 0.02 | 0.02 | 0.04 | 0.03 | 0.02 | 0.02 |
| γ*_g_*_8×E_ | γ*_b_*_26_ | 0 | 0.00 | 0.00 | 0.00 | 0.00 | 0.04 | 0.03 | 0.02 | 0.02 | 0.04 | 0.03 | 0.02 | 0.02 |
| γ*_g_*_9×E_ | γ*_b_*_26_ | 0 | 0.00 | 0.00 | 0.00 | 0.00 | 0.04 | 0.03 | 0.02 | 0.02 | 0.04 | 0.03 | 0.02 | 0.02 |
| γ*_g_*_1_ | γ*_b_*_27_ | 0 | -0.01 | -0.01 | -0.01 | -0.01 | 0.04 | 0.03 | 0.02 | 0.02 | 0.04 | 0.03 | 0.02 | 0.02 |
| γ*_g_*_2_ | γ*_b_*_27_ | 0 | -0.02 | -0.01 | -0.01 | -0.01 | 0.05 | 0.04 | 0.03 | 0.02 | 0.05 | 0.04 | 0.03 | 0.02 |
| γ*_g_*_3_ | γ*_b_*_27_ | 0 | -0.03 | -0.02 | -0.01 | -0.01 | 0.05 | 0.04 | 0.03 | 0.03 | 0.05 | 0.04 | 0.04 | 0.03 |
| γ*_g_*_4_ | γ*_b_*_27_ | 0.4 | 0.01 | 0.00 | 0.00 | 0.00 | 0.05 | 0.03 | 0.02 | 0.02 | 0.05 | 0.03 | 0.02 | 0.02 |
| γ*_g_*_5_ | γ*_b_*_27_ | 0 | 0.00 | 0.00 | 0.00 | 0.00 | 0.05 | 0.03 | 0.02 | 0.02 | 0.05 | 0.03 | 0.02 | 0.02 |
| γ*_g_*_6_ | γ*_b_*_27_ | -0.6 | 0.00 | 0.00 | 0.00 | 0.00 | 0.04 | 0.03 | 0.02 | 0.02 | 0.04 | 0.03 | 0.02 | 0.02 |
| γ*_g_*_7_ | γ*_b_*_27_ | 0 | 0.01 | 0.01 | 0.01 | 0.01 | 0.05 | 0.03 | 0.02 | 0.02 | 0.05 | 0.03 | 0.02 | 0.02 |
| γ*_g_*_8_ | γ*_b_*_27_ | 0 | 0.00 | 0.00 | 0.00 | 0.00 | 0.05 | 0.03 | 0.02 | 0.02 | 0.05 | 0.03 | 0.02 | 0.02 |
| γ*_g_*_9_ | γ*_b_*_27_ | 0 | 0.00 | 0.00 | 0.00 | 0.00 | 0.05 | 0.03 | 0.02 | 0.02 | 0.05 | 0.03 | 0.02 | 0.02 |
| z_E_ | γ*_b_*_27_ | 0 | 0.00 | 0.00 | 0.00 | 0.00 | 0.04 | 0.03 | 0.02 | 0.02 | 0.04 | 0.03 | 0.02 | 0.02 |
| γ*_g_*_1×E_ | γ*_b_*_27_ | 0 | 0.00 | 0.00 | 0.00 | 0.00 | 0.05 | 0.03 | 0.02 | 0.02 | 0.05 | 0.03 | 0.02 | 0.02 |
| γ*_g_*_2×E_ | γ*_b_*_27_ | 0 | -0.04 | -0.02 | -0.01 | 0.00 | 0.03 | 0.02 | 0.02 | 0.01 | 0.05 | 0.03 | 0.02 | 0.01 |
| γ*_g_*_3×E_ | γ*_b_*_27_ | 0 | -0.02 | -0.01 | 0.00 | 0.00 | 0.03 | 0.02 | 0.02 | 0.01 | 0.03 | 0.02 | 0.02 | 0.01 |
| γ*_g_*_4×E_ | γ*_b_*_27_ | 0.2 | 0.02 | 0.01 | 0.00 | 0.00 | 0.03 | 0.02 | 0.01 | 0.01 | 0.03 | 0.02 | 0.01 | 0.01 |
| γ*_g_*_5×E_ | γ*_b_*_27_ | 0 | 0.00 | 0.00 | 0.00 | 0.00 | 0.03 | 0.02 | 0.01 | 0.01 | 0.03 | 0.02 | 0.01 | 0.01 |
| γ*_g_*_6×E_ | γ*_b_*_27_ | 0 | 0.00 | 0.00 | 0.00 | 0.00 | 0.03 | 0.02 | 0.01 | 0.01 | 0.03 | 0.02 | 0.01 | 0.01 |
| γ*_g_*_7×E_ | γ*_b_*_27_ | 0 | 0.00 | 0.00 | 0.00 | 0.00 | 0.03 | 0.02 | 0.01 | 0.01 | 0.03 | 0.02 | 0.01 | 0.01 |
| γ*_g_*_8×E_ | γ*_b_*_27_ | 0 | 0.00 | 0.00 | 0.00 | 0.00 | 0.03 | 0.02 | 0.01 | 0.01 | 0.03 | 0.02 | 0.01 | 0.01 |
| γ*_g_*_9×E_ | γ*_b_*_27_ | 0 | 0.00 | 0.00 | 0.00 | 0.00 | 0.03 | 0.02 | 0.01 | 0.01 | 0.03 | 0.02 | 0.01 | 0.01 |
| γ*_g_*_1_ | γ*_b_*_28_ | 0 | 0.00 | 0.00 | 0.00 | 0.00 | 0.03 | 0.02 | 0.01 | 0.01 | 0.03 | 0.02 | 0.01 | 0.01 |
| γ*_g_*_2_ | γ*_b_*_28_ | 0 | -0.02 | -0.01 | -0.01 | 0.00 | 0.03 | 0.02 | 0.02 | 0.01 | 0.04 | 0.03 | 0.02 | 0.01 |
| γ*_g_*_3_ | γ*_b_*_28_ | 0 | 0.03 | 0.02 | 0.01 | 0.01 | 0.03 | 0.02 | 0.02 | 0.01 | 0.04 | 0.03 | 0.02 | 0.01 |
| γ*_g_*_4_ | γ*_b_*_28_ | -0.6 | -0.01 | 0.00 | 0.00 | 0.00 | 0.03 | 0.02 | 0.01 | 0.01 | 0.03 | 0.02 | 0.01 | 0.01 |
| γ*_g_*_5_ | γ*_b_*_28_ | 0 | -0.01 | 0.00 | 0.00 | 0.00 | 0.03 | 0.02 | 0.01 | 0.01 | 0.03 | 0.02 | 0.01 | 0.01 |
| γ*_g_*_6_ | γ*_b_*_28_ | 0 | 0.00 | 0.00 | 0.00 | 0.00 | 0.03 | 0.02 | 0.01 | 0.01 | 0.03 | 0.02 | 0.01 | 0.01 |
| γ*_g_*_7_ | γ*_b_*_28_ | 0 | 0.00 | 0.00 | 0.00 | 0.00 | 0.03 | 0.02 | 0.01 | 0.01 | 0.03 | 0.02 | 0.01 | 0.01 |
| γ*_g_*_8_ | γ*_b_*_28_ | 0 | 0.00 | 0.00 | 0.00 | 0.00 | 0.03 | 0.02 | 0.01 | 0.01 | 0.03 | 0.02 | 0.01 | 0.01 |
| γ*_g_*_9_ | γ*_b_*_28_ | 0 | 0.00 | 0.00 | 0.00 | 0.00 | 0.03 | 0.02 | 0.01 | 0.01 | 0.03 | 0.02 | 0.01 | 0.01 |
| z_E_ | γ*_b_*_28_ | 0.2 | 0.00 | 0.00 | 0.00 | 0.00 | 0.03 | 0.02 | 0.01 | 0.01 | 0.03 | 0.02 | 0.01 | 0.01 |
| γ*_g_*_1×E_ | γ*_b_*_28_ | 0 | 0.00 | 0.00 | 0.00 | 0.00 | 0.03 | 0.02 | 0.01 | 0.01 | 0.03 | 0.02 | 0.01 | 0.01 |
| γ*_g_*_2×E_ | γ*_b_*_28_ | 0 | 0.00 | 0.00 | 0.00 | 0.00 | 0.05 | 0.03 | 0.02 | 0.02 | 0.05 | 0.03 | 0.02 | 0.02 |
| γ*_g_*_3×E_ | γ*_b_*_28_ | 0 | 0.05 | 0.02 | 0.01 | 0.00 | 0.07 | 0.05 | 0.02 | 0.02 | 0.09 | 0.05 | 0.02 | 0.02 |
| γ*_g_*_4×E_ | γ*_b_*_28_ | 0.4 | -0.04 | -0.02 | -0.01 | -0.01 | 0.06 | 0.04 | 0.03 | 0.02 | 0.07 | 0.05 | 0.03 | 0.02 |
| γ*_g_*_5×E_ | γ*_b_*_28_ | 0 | 0.00 | 0.00 | -0.01 | 0.00 | 0.05 | 0.03 | 0.02 | 0.02 | 0.05 | 0.03 | 0.02 | 0.02 |
| γ*_g_*_6×E_ | γ*_b_*_28_ | 0 | 0.00 | 0.00 | 0.00 | 0.00 | 0.05 | 0.03 | 0.03 | 0.02 | 0.05 | 0.03 | 0.03 | 0.02 |
| γ*_g_*_7×E_ | γ*_b_*_28_ | 0 | 0.00 | 0.00 | 0.00 | 0.00 | 0.05 | 0.03 | 0.02 | 0.02 | 0.05 | 0.03 | 0.02 | 0.02 |
| γ*_g_*_8×E_ | γ*_b_*_28_ | 0 | 0.00 | 0.00 | 0.00 | 0.00 | 0.04 | 0.03 | 0.02 | 0.02 | 0.04 | 0.03 | 0.02 | 0.02 |
| γ*_g_*_9×E_ | γ*_b_*_28_ | 0 | 0.00 | 0.00 | 0.00 | 0.00 | 0.05 | 0.03 | 0.02 | 0.02 | 0.05 | 0.03 | 0.02 | 0.02 |
| γ*_g_*_1_ | γ*_b_*_29_ | 0 | 0.00 | 0.00 | 0.00 | 0.00 | 0.05 | 0.04 | 0.02 | 0.02 | 0.05 | 0.04 | 0.02 | 0.02 |
| γ*_g_*_2_ | γ*_b_*_29_ | 0 | 0.00 | 0.00 | 0.00 | 0.00 | 0.05 | 0.04 | 0.02 | 0.02 | 0.05 | 0.04 | 0.03 | 0.02 |
| γ*_g_*_3_ | γ*_b_*_29_ | 0 | 0.00 | 0.00 | 0.00 | 0.01 | 0.05 | 0.04 | 0.02 | 0.02 | 0.05 | 0.04 | 0.02 | 0.02 |
| γ*_g_*_4_ | γ*_b_*_29_ | 0.6 | -0.03 | -0.01 | -0.01 | 0.00 | 0.05 | 0.04 | 0.02 | 0.02 | 0.06 | 0.04 | 0.02 | 0.02 |
| γ*_g_*_5_ | γ*_b_*_29_ | 0.4 | 0.04 | 0.02 | 0.01 | 0.01 | 0.06 | 0.04 | 0.03 | 0.02 | 0.07 | 0.05 | 0.03 | 0.02 |
| γ*_g_*_6_ | γ*_b_*_29_ | 0 | 0.00 | 0.00 | 0.00 | 0.00 | 0.05 | 0.03 | 0.02 | 0.02 | 0.05 | 0.03 | 0.02 | 0.02 |
| γ*_g_*_7_ | γ*_b_*_29_ | 0 | 0.00 | 0.00 | 0.00 | 0.00 | 0.05 | 0.03 | 0.02 | 0.02 | 0.05 | 0.03 | 0.02 | 0.02 |
| γ*_g_*_8_ | γ*_b_*_29_ | 0 | 0.00 | 0.00 | 0.00 | 0.00 | 0.05 | 0.03 | 0.02 | 0.02 | 0.05 | 0.03 | 0.02 | 0.02 |
| γ*_g_*_9_ | γ*_b_*_29_ | 0 | 0.00 | 0.00 | 0.00 | 0.00 | 0.05 | 0.03 | 0.02 | 0.02 | 0.05 | 0.03 | 0.02 | 0.02 |
| z_E_ | γ*_b_*_29_ | 0.2 | 0.00 | 0.00 | 0.00 | 0.00 | 0.05 | 0.03 | 0.02 | 0.02 | 0.05 | 0.03 | 0.02 | 0.02 |
| γ*_g_*_1×E_ | γ*_b_*_29_ | 0 | 0.00 | 0.00 | 0.00 | 0.00 | 0.05 | 0.03 | 0.02 | 0.02 | 0.05 | 0.03 | 0.02 | 0.02 |
| γ*_g_*_2×E_ | γ*_b_*_29_ | 0 | -0.04 | -0.02 | -0.01 | -0.01 | 0.05 | 0.04 | 0.02 | 0.02 | 0.06 | 0.04 | 0.03 | 0.02 |
| γ*_g_*_3×E_ | γ*_b_*_29_ | 0 | -0.01 | 0.00 | 0.00 | 0.00 | 0.05 | 0.04 | 0.03 | 0.02 | 0.05 | 0.04 | 0.03 | 0.02 |
| γ*_g_*_4×E_ | γ*_b_*_29_ | -0.4 | 0.05 | 0.03 | 0.02 | 0.01 | 0.04 | 0.03 | 0.02 | 0.02 | 0.07 | 0.04 | 0.03 | 0.02 |
| γ*_g_*_5×E_ | γ*_b_*_29_ | 0 | 0.00 | 0.00 | 0.00 | 0.00 | 0.05 | 0.04 | 0.03 | 0.02 | 0.05 | 0.04 | 0.03 | 0.02 |
| γ*_g_*_6×E_ | γ*_b_*_29_ | 0 | -0.01 | 0.00 | 0.00 | 0.00 | 0.05 | 0.04 | 0.03 | 0.02 | 0.05 | 0.04 | 0.03 | 0.02 |
| γ*_g_*_7×E_ | γ*_b_*_29_ | 0 | -0.01 | 0.00 | 0.00 | 0.00 | 0.05 | 0.04 | 0.03 | 0.02 | 0.05 | 0.04 | 0.03 | 0.02 |
| γ*_g_*_8×E_ | γ*_b_*_29_ | 0 | 0.00 | 0.00 | 0.00 | 0.01 | 0.05 | 0.04 | 0.03 | 0.02 | 0.05 | 0.04 | 0.03 | 0.02 |
| γ*_g_*_9×E_ | γ*_b_*_29_ | 0 | 0.00 | 0.00 | 0.00 | 0.00 | 0.05 | 0.04 | 0.03 | 0.02 | 0.05 | 0.04 | 0.03 | 0.02 |
| γ*_g_*_1_ | γ*_b_*_30_ | 0 | 0.00 | 0.00 | 0.00 | 0.00 | 0.05 | 0.04 | 0.03 | 0.02 | 0.05 | 0.04 | 0.03 | 0.02 |
| γ*_g_*_2_ | γ*_b_*_30_ | 0 | 0.00 | 0.00 | 0.00 | 0.00 | 0.05 | 0.04 | 0.03 | 0.02 | 0.05 | 0.04 | 0.03 | 0.02 |
| γ*_g_*_3_ | γ*_b_*_30_ | 0 | -0.02 | -0.01 | 0.00 | 0.00 | 0.05 | 0.04 | 0.03 | 0.02 | 0.05 | 0.04 | 0.03 | 0.02 |
| γ*_g_*_4_ | γ*_b_*_30_ | 0 | 0.01 | 0.00 | 0.00 | 0.00 | 0.05 | 0.04 | 0.03 | 0.02 | 0.05 | 0.04 | 0.03 | 0.02 |
| γ*_g_*_5_ | γ*_b_*_30_ | -0.6 | 0.00 | 0.00 | 0.00 | 0.00 | 0.05 | 0.04 | 0.03 | 0.02 | 0.05 | 0.04 | 0.03 | 0.02 |
| γ*_g_*_6_ | γ*_b_*_30_ | 0.4 | 0.01 | 0.00 | 0.01 | 0.00 | 0.05 | 0.04 | 0.03 | 0.02 | 0.05 | 0.04 | 0.03 | 0.02 |
| γ*_g_*_7_ | γ*_b_*_30_ | 0 | 0.00 | 0.00 | 0.00 | 0.00 | 0.05 | 0.04 | 0.03 | 0.02 | 0.05 | 0.04 | 0.03 | 0.02 |
| γ*_g_*_8_ | γ*_b_*_30_ | 0 | 0.00 | 0.00 | 0.00 | 0.00 | 0.05 | 0.04 | 0.03 | 0.02 | 0.05 | 0.04 | 0.03 | 0.02 |
| γ*_g_*_9_ | γ*_b_*_30_ | 0 | 0.00 | 0.00 | 0.00 | 0.00 | 0.05 | 0.04 | 0.03 | 0.02 | 0.05 | 0.04 | 0.03 | 0.02 |
| z_E_ | γ*_b_*_30_ | 0 | 0.00 | 0.00 | 0.00 | 0.00 | 0.05 | 0.04 | 0.03 | 0.02 | 0.05 | 0.04 | 0.03 | 0.02 |
| γ*_g_*_1×E_ | γ*_b_*_30_ | 0 | 0.00 | 0.00 | 0.00 | 0.00 | 0.05 | 0.04 | 0.03 | 0.02 | 0.05 | 0.04 | 0.03 | 0.02 |
| γ*_g_*_2×E_ | γ*_b_*_30_ | 0 | 0.04 | 0.02 | 0.01 | 0.01 | 0.04 | 0.03 | 0.02 | 0.04 | 0.06 | 0.04 | 0.02 | 0.04 |
| γ*_g_*_3×E_ | γ*_b_*_30_ | 0 | -0.01 | 0.00 | 0.00 | 0.00 | 0.04 | 0.03 | 0.02 | 0.02 | 0.05 | 0.03 | 0.02 | 0.02 |
| γ*_g_*_4×E_ | γ*_b_*_30_ | 0 | -0.01 | 0.00 | 0.00 | 0.00 | 0.05 | 0.03 | 0.02 | 0.02 | 0.05 | 0.03 | 0.02 | 0.02 |
| γ*_g_*_5×E_ | γ*_b_*_30_ | 0.2 | 0.00 | 0.00 | 0.00 | 0.00 | 0.04 | 0.03 | 0.02 | 0.02 | 0.04 | 0.03 | 0.02 | 0.02 |
| γ*_g_*_6×E_ | γ*_b_*_30_ | -0.4 | 0.00 | 0.00 | 0.00 | 0.00 | 0.04 | 0.03 | 0.02 | 0.02 | 0.04 | 0.03 | 0.02 | 0.02 |
| γ*_g_*_7×E_ | γ*_b_*_30_ | 0 | 0.00 | 0.00 | 0.00 | 0.00 | 0.04 | 0.03 | 0.02 | 0.02 | 0.05 | 0.03 | 0.02 | 0.02 |
| γ*_g_*_8×E_ | γ*_b_*_30_ | 0 | 0.00 | 0.00 | 0.00 | 0.00 | 0.04 | 0.03 | 0.02 | 0.02 | 0.04 | 0.03 | 0.02 | 0.02 |
| γ*_g_*_9×E_ | γ*_b_*_30_ | 0 | 0.00 | 0.00 | 0.00 | 0.00 | 0.04 | 0.03 | 0.02 | 0.02 | 0.04 | 0.03 | 0.02 | 0.02 |
| γ*_g_*_1_ | γ*_b_*_31_ | 0 | 0.00 | 0.00 | 0.00 | 0.00 | 0.04 | 0.03 | 0.02 | 0.02 | 0.04 | 0.03 | 0.02 | 0.02 |
| γ*_g_*_2_ | γ*_b_*_31_ | 0 | -0.01 | -0.01 | 0.00 | 0.00 | 0.05 | 0.03 | 0.03 | 0.02 | 0.05 | 0.03 | 0.03 | 0.02 |
| γ*_g_*_3_ | γ*_b_*_31_ | 0 | -0.04 | -0.02 | -0.01 | -0.01 | 0.03 | 0.02 | 0.04 | 0.01 | 0.05 | 0.03 | 0.04 | 0.01 |
| γ*_g_*_4_ | γ*_b_*_31_ | 0.4 | -0.02 | -0.01 | 0.00 | 0.00 | 0.03 | 0.02 | 0.03 | 0.01 | 0.04 | 0.02 | 0.03 | 0.01 |
| γ*_g_*_5_ | γ*_b_*_31_ | 0 | 0.01 | 0.01 | 0.00 | 0.00 | 0.03 | 0.02 | 0.01 | 0.01 | 0.03 | 0.02 | 0.01 | 0.01 |
| γ*_g_*_6_ | γ*_b_*_31_ | -0.6 | 0.00 | 0.00 | 0.00 | 0.00 | 0.03 | 0.02 | 0.01 | 0.01 | 0.03 | 0.02 | 0.01 | 0.01 |
| γ*_g_*_7_ | γ*_b_*_31_ | 0 | 0.00 | 0.00 | 0.00 | 0.00 | 0.03 | 0.02 | 0.01 | 0.01 | 0.03 | 0.02 | 0.01 | 0.01 |
| γ*_g_*_8_ | γ*_b_*_31_ | 0 | 0.00 | 0.00 | 0.00 | 0.00 | 0.03 | 0.02 | 0.01 | 0.01 | 0.03 | 0.02 | 0.01 | 0.01 |
| γ*_g_*_9_ | γ*_b_*_31_ | 0 | 0.00 | 0.00 | 0.00 | 0.00 | 0.03 | 0.02 | 0.01 | 0.01 | 0.03 | 0.02 | 0.01 | 0.01 |
| z_E_ | γ*_b_*_31_ | 0 | 0.00 | 0.00 | 0.00 | 0.00 | 0.03 | 0.02 | 0.01 | 0.01 | 0.03 | 0.02 | 0.01 | 0.01 |
| γ*_g_*_1×E_ | γ*_b_*_31_ | 0 |  | 0.00 | 0.00 | 0.00 | 0.03 | 0.02 | 0.01 | 0.01 | 0.03 | 0.02 | 0.01 | 0.01 |
| γ*_g_*_2×E_ | γ*_b_*_31_ | 0 | -0.02 | -0.01 | -0.01 | 0.00 | 0.03 | 0.02 | 0.02 | 0.01 | 0.04 | 0.03 | 0.02 | 0.01 |
| γ*_g_*_3×E_ | γ*_b_*_31_ | 0 | 0.03 | 0.01 | 0.01 | 0.00 | 0.03 | 0.02 | 0.03 | 0.01 | 0.04 | 0.03 | 0.03 | 0.01 |
| γ*_g_*_4×E_ | γ*_b_*_31_ | 0.2 | -0.01 | 0.00 | 0.00 | 0.00 | 0.03 | 0.02 | 0.01 | 0.01 | 0.03 | 0.02 | 0.01 | 0.01 |
| γ*_g_*_5×E_ | γ*_b_*_31_ | 0 | -0.01 | 0.00 | 0.00 | 0.00 | 0.03 | 0.02 | 0.01 | 0.01 | 0.03 | 0.02 | 0.01 | 0.01 |
| γ*_g_*_6×E_ | γ*_b_*_31_ | 0 | 0.00 | 0.00 | 0.00 | 0.00 | 0.03 | 0.02 | 0.01 | 0.01 | 0.03 | 0.02 | 0.01 | 0.01 |
| γ*_g_*_7×E_ | γ*_b_*_31_ | 0 | 0.00 | 0.00 | 0.00 | 0.00 | 0.03 | 0.02 | 0.01 | 0.01 | 0.03 | 0.02 | 0.01 | 0.01 |
| γ*_g_*_8×E_ | γ*_b_*_31_ | 0 | 0.00 | 0.00 | 0.00 | 0.00 | 0.03 | 0.02 | 0.01 | 0.01 | 0.03 | 0.02 | 0.01 | 0.01 |
| γ*_g_*_9×E_ | γ*_b_*_31_ | 0 | 0.00 | 0.00 | 0.00 | 0.00 | 0.03 | 0.02 | 0.01 | 0.01 | 0.03 | 0.02 | 0.01 | 0.01 |
| γ*_g_*_1_ | γ*_b_*_32_ | 0 | 0.00 | 0.00 | 0.00 | 0.00 | 0.03 | 0.02 | 0.01 | 0.01 | 0.03 | 0.02 | 0.01 | 0.01 |
| γ*_g_*_2_ | γ*_b_*_32_ | 0 | 0.00 | 0.00 | 0.00 | 0.00 | 0.03 | 0.02 | 0.01 | 0.01 | 0.03 | 0.02 | 0.01 | 0.01 |
| γ*_g_*_3_ | γ*_b_*_32_ | 0 | -0.01 | 0.00 | 0.00 | 0.00 | 0.05 | 0.03 | 0.02 | 0.02 | 0.05 | 0.03 | 0.02 | 0.02 |
| γ*_g_*_4_ | γ*_b_*_32_ | -0.6 | 0.05 | 0.02 | 0.01 | 0.01 | 0.04 | 0.05 | 0.04 | 0.04 | 0.06 | 0.05 | 0.05 | 0.04 |
| γ*_g_*_5_ | γ*_b_*_32_ | 0 | -0.04 | -0.02 | -0.01 | -0.01 | 0.05 | 0.04 | 0.04 | 0.03 | 0.06 | 0.05 | 0.04 | 0.03 |
| γ*_g_*_6_ | γ*_b_*_32_ | 0 | 0.01 | 0.00 | 0.00 | 0.00 | 0.04 | 0.03 | 0.02 | 0.02 | 0.04 | 0.03 | 0.02 | 0.02 |
| γ*_g_*_7_ | γ*_b_*_32_ | 0 | 0.00 | 0.00 | 0.00 | 0.00 | 0.04 | 0.03 | 0.02 | 0.02 | 0.04 | 0.03 | 0.02 | 0.02 |
| γ*_g_*_8_ | γ*_b_*_32_ | 0 | 0.00 | 0.00 | 0.00 | 0.00 | 0.05 | 0.03 | 0.02 | 0.02 | 0.05 | 0.03 | 0.02 | 0.02 |
| γ*_g_*_9_ | γ*_b_*_32_ | 0 | 0.00 | 0.00 | 0.00 | 0.00 | 0.04 | 0.03 | 0.02 | 0.02 | 0.04 | 0.03 | 0.02 | 0.02 |
| z_E_ | γ*_b_*_32_ | 0.2 | 0.00 | 0.00 | 0.00 | 0.00 | 0.05 | 0.03 | 0.02 | 0.02 | 0.05 | 0.03 | 0.02 | 0.02 |
| γ*_g_*_1×E_ | γ*_b_*_32_ | 0 | 0.00 | 0.00 | 0.00 | 0.00 | 0.05 | 0.03 | 0.02 | 0.02 | 0.05 | 0.03 | 0.02 | 0.02 |
| γ*_g_*_2×E_ | γ*_b_*_32_ | 0 | 0.00 | 0.00 | 0.00 | 0.00 | 0.05 | 0.04 | 0.02 | 0.02 | 0.05 | 0.04 | 0.02 | 0.02 |
| γ*_g_*_3×E_ | γ*_b_*_32_ | 0 | 0.00 | 0.00 | 0.00 | 0.00 | 0.05 | 0.03 | 0.02 | 0.02 | 0.05 | 0.03 | 0.02 | 0.02 |
| γ*_g_*_4×E_ | γ*_b_*_32_ | 0.4 | -0.02 | -0.01 | -0.01 | 0.00 | 0.05 | 0.04 | 0.03 | 0.02 | 0.05 | 0.04 | 0.03 | 0.02 |
| γ*_g_*_5×E_ | γ*_b_*_32_ | 0 | 0.03 | 0.02 | 0.01 | 0.00 | 0.05 | 0.04 | 0.04 | 0.03 | 0.06 | 0.05 | 0.04 | 0.03 |
| γ*_g_*_6×E_ | γ*_b_*_32_ | 0 | -0.01 | 0.00 | -0.01 | 0.00 | 0.05 | 0.03 | 0.02 | 0.02 | 0.05 | 0.03 | 0.03 | 0.02 |
| γ*_g_*_7×E_ | γ*_b_*_32_ | 0 | 0.00 | 0.00 | 0.00 | 0.00 | 0.05 | 0.03 | 0.02 | 0.02 | 0.05 | 0.03 | 0.02 | 0.02 |
| γ*_g_*_8×E_ | γ*_b_*_32_ | 0 | 0.00 | 0.00 | 0.00 | 0.00 | 0.05 | 0.03 | 0.02 | 0.02 | 0.05 | 0.03 | 0.02 | 0.02 |
| γ*_g_*_9×E_ | γ*_b_*_32_ | 0 | 0.00 | 0.00 | 0.00 | 0.00 | 0.05 | 0.03 | 0.02 | 0.02 | 0.05 | 0.03 | 0.02 | 0.02 |
| γ*_g_*_1_ | γ*_b_*_33_ | 0 | 0.00 | 0.00 | 0.00 | 0.00 | 0.05 | 0.04 | 0.02 | 0.02 | 0.05 | 0.04 | 0.02 | 0.02 |
| γ*_g_*_2_ | γ*_b_*_33_ | 0 | 0.00 | 0.00 | 0.00 | 0.00 | 0.05 | 0.03 | 0.02 | 0.02 | 0.05 | 0.03 | 0.02 | 0.02 |
| γ*_g_*_3_ | γ*_b_*_33_ | 0 | -0.04 | -0.02 | -0.01 | -0.01 | 0.05 | 0.04 | 0.03 | 0.03 | 0.06 | 0.04 | 0.03 | 0.03 |
| γ*_g_*_4_ | γ*_b_*_33_ | 0.6 | 0.00 | 0.00 | 0.00 | 0.00 | 0.05 | 0.04 | 0.03 | 0.02 | 0.05 | 0.04 | 0.03 | 0.02 |
| γ*_g_*_5_ | γ*_b_*_33_ | 0.4 | 0.05 | 0.02 | 0.01 | 0.01 | 0.04 | 0.03 | 0.02 | 0.04 | 0.06 | 0.04 | 0.02 | 0.04 |
| γ*_g_*_6_ | γ*_b_*_33_ | 0 | 0.00 | 0.00 | 0.00 | 0.00 | 0.05 | 0.04 | 0.03 | 0.02 | 0.05 | 0.04 | 0.03 | 0.02 |
| γ*_g_*_7_ | γ*_b_*_33_ | 0 | 0.00 | 0.00 | 0.00 | 0.00 | 0.05 | 0.04 | 0.03 | 0.02 | 0.05 | 0.04 | 0.03 | 0.02 |
| γ*_g_*_8_ | γ*_b_*_33_ | 0 | 0.01 | 0.01 | 0.01 | 0.00 | 0.05 | 0.04 | 0.03 | 0.02 | 0.05 | 0.04 | 0.03 | 0.02 |
| γ*_g_*_9_ | γ*_b_*_33_ | 0 | 0.00 | 0.00 | 0.00 | 0.00 | 0.05 | 0.04 | 0.03 | 0.02 | 0.05 | 0.04 | 0.03 | 0.02 |
| z_E_ | γ*_b_*_33_ | 0.2 | 0.00 | 0.00 | 0.00 | 0.00 | 0.05 | 0.04 | 0.03 | 0.02 | 0.05 | 0.04 | 0.03 | 0.02 |
| γ*_g_*_1×E_ | γ*_b_*_33_ | 0 | 0.00 | 0.00 | 0.00 | 0.00 | 0.05 | 0.04 | 0.03 | 0.02 | 0.05 | 0.04 | 0.03 | 0.02 |
| γ*_g_*_2×E_ | γ*_b_*_33_ | 0 | 0.00 | 0.00 | 0.00 | 0.00 | 0.05 | 0.03 | 0.03 | 0.02 | 0.05 | 0.03 | 0.03 | 0.02 |
| γ*_g_*_3×E_ | γ*_b_*_33_ | 0 | -0.02 | -0.01 | -0.01 | -0.01 | 0.05 | 0.04 | 0.03 | 0.02 | 0.05 | 0.04 | 0.03 | 0.02 |
| γ*_g_*_4×E_ | γ*_b_*_33_ | -0.4 | 0.01 | 0.00 | 0.00 | 0.00 | 0.05 | 0.04 | 0.03 | 0.02 | 0.05 | 0.04 | 0.03 | 0.02 |
| γ*_g_*_5×E_ | γ*_b_*_33_ | 0 | 0.00 | 0.00 | 0.00 | 0.00 | 0.05 | 0.04 | 0.03 | 0.02 | 0.05 | 0.04 | 0.03 | 0.02 |
| γ*_g_*_6×E_ | γ*_b_*_33_ | 0 | 0.01 | 0.01 | 0.01 | 0.01 | 0.05 | 0.04 | 0.03 | 0.02 | 0.05 | 0.04 | 0.03 | 0.02 |
| γ*_g_*_7×E_ | γ*_b_*_33_ | 0 | 0.00 | 0.00 | 0.00 | 0.00 | 0.05 | 0.04 | 0.03 | 0.02 | 0.05 | 0.04 | 0.03 | 0.02 |
| γ*_g_*_8×E_ | γ*_b_*_33_ | 0 | 0.00 | 0.00 | 0.00 | 0.00 | 0.05 | 0.04 | 0.03 | 0.02 | 0.05 | 0.04 | 0.03 | 0.02 |
| γ*_g_*_9×E_ | γ*_b_*_33_ | 0 | 0.00 | 0.00 | 0.00 | 0.00 | 0.05 | 0.04 | 0.03 | 0.02 | 0.05 | 0.04 | 0.03 | 0.02 |
| γ*_g_*_1_ | γ*_b_*_34_ | 0 | 0.00 | 0.01 | 0.00 | 0.00 | 0.05 | 0.04 | 0.03 | 0.02 | 0.05 | 0.04 | 0.03 | 0.02 |
| γ*_g_*_2_ | γ*_b_*_34_ | 0 | 0.00 | 0.00 | 0.00 | 0.00 | 0.05 | 0.04 | 0.03 | 0.02 | 0.05 | 0.04 | 0.03 | 0.02 |
| γ*_g_*_3_ | γ*_b_*_34_ | 0 | 0.04 | 0.02 | 0.01 | 0.00 | 0.04 | 0.05 | 0.04 | 0.04 | 0.06 | 0.05 | 0.04 | 0.04 |
| γ*_g_*_4_ | γ*_b_*_34_ | 0 | -0.01 | -0.01 | 0.00 | 0.00 | 0.04 | 0.03 | 0.02 | 0.02 | 0.04 | 0.03 | 0.02 | 0.02 |
| γ*_g_*_5_ | γ*_b_*_34_ | -0.6 | -0.01 | 0.00 | 0.00 | 0.00 | 0.04 | 0.03 | 0.02 | 0.02 | 0.05 | 0.03 | 0.02 | 0.02 |
| γ*_g_*_6_ | γ*_b_*_34_ | 0.4 | 0.00 | 0.00 | 0.00 | 0.00 | 0.04 | 0.03 | 0.02 | 0.02 | 0.04 | 0.03 | 0.02 | 0.02 |
| γ*_g_*_7_ | γ*_b_*_34_ | 0 | 0.00 | 0.00 | 0.00 | 0.00 | 0.04 | 0.03 | 0.02 | 0.02 | 0.04 | 0.03 | 0.02 | 0.02 |
| γ*_g_*_8_ | γ*_b_*_34_ | 0 | 0.00 | 0.00 | 0.00 | 0.00 | 0.04 | 0.03 | 0.02 | 0.02 | 0.04 | 0.03 | 0.02 | 0.02 |
| γ*_g_*_9_ | γ*_b_*_34_ | 0 | 0.00 | 0.00 | 0.00 | 0.00 | 0.04 | 0.03 | 0.02 | 0.02 | 0.04 | 0.03 | 0.02 | 0.02 |
| z_E_ | γ*_b_*_34_ | 0 | 0.00 | 0.00 | 0.00 | 0.00 | 0.04 | 0.03 | 0.02 | 0.02 | 0.04 | 0.03 | 0.02 | 0.02 |
| γ*_g_*_1×E_ | γ*_b_*_34_ | 0 | -0.01 | -0.01 | -0.01 | -0.01 | 0.04 | 0.03 | 0.02 | 0.02 | 0.04 | 0.03 | 0.02 | 0.02 |
| γ*_g_*_2×E_ | γ*_b_*_34_ | 0 | -0.02 | -0.01 | -0.01 | -0.01 | 0.05 | 0.04 | 0.03 | 0.02 | 0.05 | 0.04 | 0.03 | 0.02 |
| γ*_g_*_3×E_ | γ*_b_*_34_ | 0 | -0.03 | -0.02 | -0.01 | -0.01 | 0.05 | 0.04 | 0.03 | 0.03 | 0.05 | 0.04 | 0.04 | 0.03 |
| γ*_g_*_4×E_ | γ*_b_*_34_ | 0 | 0.01 | 0.00 | 0.00 | 0.00 | 0.05 | 0.03 | 0.02 | 0.02 | 0.05 | 0.03 | 0.02 | 0.02 |
| γ*_g_*_5×E_ | γ*_b_*_34_ | 0.2 | 0.00 | 0.00 | 0.00 | 0.00 | 0.05 | 0.03 | 0.02 | 0.02 | 0.05 | 0.03 | 0.02 | 0.02 |
| γ*_g_*_6×E_ | γ*_b_*_34_ | -0.4 | 0.00 | 0.00 | 0.00 | 0.00 | 0.04 | 0.03 | 0.02 | 0.02 | 0.04 | 0.03 | 0.02 | 0.02 |
| γ*_g_*_7×E_ | γ*_b_*_34_ | 0 | 0.01 | 0.01 | 0.01 | 0.01 | 0.05 | 0.03 | 0.02 | 0.02 | 0.05 | 0.03 | 0.02 | 0.02 |
| γ*_g_*_8×E_ | γ*_b_*_34_ | 0 | 0.00 | 0.00 | 0.00 | 0.00 | 0.05 | 0.03 | 0.02 | 0.02 | 0.05 | 0.03 | 0.02 | 0.02 |
| γ*_g_*_9×E_ | γ*_b_*_34_ | 0 | 0.00 | 0.00 | 0.00 | 0.00 | 0.05 | 0.03 | 0.02 | 0.02 | 0.05 | 0.03 | 0.02 | 0.02 |
| γ*_g_*_1_ | γ*_b_*_35_ | 0 | 0.00 | 0.00 | 0.00 | 0.00 | 0.04 | 0.03 | 0.02 | 0.02 | 0.04 | 0.03 | 0.02 | 0.02 |
| γ*_g_*_2_ | γ*_b_*_35_ | 0 | 0.00 | 0.00 | 0.00 | 0.00 | 0.05 | 0.03 | 0.02 | 0.02 | 0.05 | 0.03 | 0.02 | 0.02 |
| γ*_g_*_3_ | γ*_b_*_35_ | 0 | -0.04 | -0.02 | -0.01 | 0.00 | 0.03 | 0.02 | 0.02 | 0.01 | 0.05 | 0.03 | 0.02 | 0.01 |
| γ*_g_*_4_ | γ*_b_*_35_ | 0.4 | -0.02 | -0.01 | 0.00 | 0.00 | 0.03 | 0.02 | 0.02 | 0.01 | 0.03 | 0.02 | 0.02 | 0.01 |
| γ*_g_*_5_ | γ*_b_*_35_ | 0 | 0.02 | 0.01 | 0.00 | 0.00 | 0.03 | 0.02 | 0.01 | 0.01 | 0.03 | 0.02 | 0.01 | 0.01 |
| γ*_g_*_6_ | γ*_b_*_35_ | -0.6 | 0.00 | 0.00 | 0.00 | 0.00 | 0.03 | 0.02 | 0.01 | 0.01 | 0.03 | 0.02 | 0.01 | 0.01 |
| γ*_g_*_7_ | γ*_b_*_35_ | 0 | 0.00 | 0.00 | 0.00 | 0.00 | 0.03 | 0.02 | 0.01 | 0.01 | 0.03 | 0.02 | 0.01 | 0.01 |
| γ*_g_*_8_ | γ*_b_*_35_ | 0 | 0.00 | 0.00 | 0.00 | 0.00 | 0.03 | 0.02 | 0.01 | 0.01 | 0.03 | 0.02 | 0.01 | 0.01 |
| γ*_g_*_9_ | γ*_b_*_35_ | 0 | 0.00 | 0.00 | 0.00 | 0.00 | 0.03 | 0.02 | 0.01 | 0.01 | 0.03 | 0.02 | 0.01 | 0.01 |
| z_E_ | γ*_b_*_35_ | 0 | 0.00 | 0.00 | 0.00 | 0.00 | 0.03 | 0.02 | 0.01 | 0.01 | 0.03 | 0.02 | 0.01 | 0.01 |
| γ*_g_*_1×E_ | γ*_b_*_35_ | 0 | 0.00 | 0.00 | 0.00 | 0.00 | 0.03 | 0.02 | 0.01 | 0.01 | 0.03 | 0.02 | 0.01 | 0.01 |
| γ*_g_*_2×E_ | γ*_b_*_35_ | 0 | -0.02 | -0.01 | -0.01 | 0.00 | 0.03 | 0.02 | 0.02 | 0.01 | 0.04 | 0.03 | 0.02 | 0.01 |
| γ*_g_*_3×E_ | γ*_b_*_35_ | 0 | 0.03 | 0.02 | 0.01 | 0.01 | 0.03 | 0.02 | 0.02 | 0.01 | 0.04 | 0.03 | 0.02 | 0.01 |
| γ*_g_*_4×E_ | γ*_b_*_35_ | 0.2 | -0.01 | 0.00 | 0.00 | 0.00 | 0.03 | 0.02 | 0.01 | 0.01 | 0.03 | 0.02 | 0.01 | 0.01 |
| γ*_g_*_5×E_ | γ*_b_*_35_ | 0 | -0.01 | 0.00 | 0.00 | 0.00 | 0.03 | 0.02 | 0.01 | 0.01 | 0.03 | 0.02 | 0.01 | 0.01 |
| γ*_g_*_6×E_ | γ*_b_*_35_ | 0 | 0.00 | 0.00 | 0.00 | 0.00 | 0.03 | 0.02 | 0.01 | 0.01 | 0.03 | 0.02 | 0.01 | 0.01 |
| γ*_g_*_7×E_ | γ*_b_*_35_ | 0 | 0.00 | 0.00 | 0.00 | 0.00 | 0.03 | 0.02 | 0.01 | 0.01 | 0.03 | 0.02 | 0.01 | 0.01 |
| γ*_g_*_8×E_ | γ*_b_*_35_ | 0 | 0.00 | 0.00 | 0.00 | 0.00 | 0.03 | 0.02 | 0.01 | 0.01 | 0.03 | 0.02 | 0.01 | 0.01 |
| γ*_g_*_9×E_ | γ*_b_*_35_ | 0 | 0.00 | 0.00 | 0.00 | 0.00 | 0.03 | 0.02 | 0.01 | 0.01 | 0.03 | 0.02 | 0.01 | 0.01 |
| γ*_g_*_1_ | γ*_b_*_36_ | 0 | 0.00 | 0.00 | 0.00 | 0.00 | 0.03 | 0.02 | 0.01 | 0.01 | 0.03 | 0.02 | 0.01 | 0.01 |
| γ*_g_*_2_ | γ*_b_*_36_ | 0 | 0.00 | 0.00 | 0.00 | 0.00 | 0.03 | 0.02 | 0.01 | 0.01 | 0.03 | 0.02 | 0.01 | 0.01 |
| γ*_g_*_3_ | γ*_b_*_36_ | 0 | 0.00 | 0.00 | 0.00 | 0.00 | 0.05 | 0.03 | 0.02 | 0.02 | 0.05 | 0.03 | 0.02 | 0.02 |
| γ*_g_*_4_ | γ*_b_*_36_ | -0.6 | 0.05 | 0.02 | 0.01 | 0.00 | 0.07 | 0.05 | 0.02 | 0.02 | 0.09 | 0.05 | 0.02 | 0.02 |
| γ*_g_*_5_ | γ*_b_*_36_ | 0 | -0.04 | -0.02 | -0.01 | -0.01 | 0.06 | 0.04 | 0.03 | 0.02 | 0.07 | 0.05 | 0.03 | 0.02 |
| γ*_g_*_6_ | γ*_b_*_36_ | 0 | 0.00 | 0.00 | -0.01 | 0.00 | 0.05 | 0.03 | 0.02 | 0.02 | 0.05 | 0.03 | 0.02 | 0.02 |
| γ*_g_*_7_ | γ*_b_*_36_ | 0 | 0.00 | 0.00 | 0.00 | 0.00 | 0.05 | 0.03 | 0.03 | 0.02 | 0.05 | 0.03 | 0.03 | 0.02 |
| γ*_g_*_8_ | γ*_b_*_36_ | 0 | 0.00 | 0.00 | 0.00 | 0.00 | 0.05 | 0.03 | 0.02 | 0.02 | 0.05 | 0.03 | 0.02 | 0.02 |
| γ*_g_*_9_ | γ*_b_*_36_ | 0 | 0.00 | 0.00 | 0.00 | 0.00 | 0.04 | 0.03 | 0.02 | 0.02 | 0.04 | 0.03 | 0.02 | 0.02 |
| z_E_ | γ*_b_*_36_ | 0.2 | 0.00 | 0.00 | 0.00 | 0.00 | 0.05 | 0.03 | 0.02 | 0.02 | 0.05 | 0.03 | 0.02 | 0.02 |
| γ*_g_*_1×E_ | γ*_b_*_36_ | 0 | 0.00 | 0.00 | 0.00 | 0.00 | 0.05 | 0.04 | 0.02 | 0.02 | 0.05 | 0.04 | 0.02 | 0.02 |
| γ*_g_*_2×E_ | γ*_b_*_36_ | 0 | 0.00 | 0.00 | 0.00 | 0.00 | 0.05 | 0.04 | 0.02 | 0.02 | 0.05 | 0.04 | 0.03 | 0.02 |
| γ*_g_*_3×E_ | γ*_b_*_36_ | 0 | 0.00 | 0.00 | 0.00 | 0.01 | 0.05 | 0.04 | 0.02 | 0.02 | 0.05 | 0.04 | 0.02 | 0.02 |
| γ*_g_*_4×E_ | γ*_b_*_36_ | 0.4 | -0.03 | -0.01 | -0.01 | 0.00 | 0.05 | 0.04 | 0.02 | 0.02 | 0.06 | 0.04 | 0.02 | 0.02 |
| γ*_g_*_5×E_ | γ*_b_*_36_ | 0 | 0.04 | 0.02 | 0.01 | 0.01 | 0.06 | 0.04 | 0.03 | 0.02 | 0.07 | 0.05 | 0.03 | 0.02 |
| γ*_g_*_6×E_ | γ*_b_*_36_ | 0 | 0.00 | 0.00 | 0.00 | 0.00 | 0.05 | 0.03 | 0.02 | 0.02 | 0.05 | 0.03 | 0.02 | 0.02 |
| γ*_g_*_7×E_ | γ*_b_*_36_ | 0 | 0.00 | 0.00 | 0.00 | 0.00 | 0.05 | 0.03 | 0.02 | 0.02 | 0.05 | 0.03 | 0.02 | 0.02 |
| γ*_g_*_8×E_ | γ*_b_*_36_ | 0 | 0.00 | 0.00 | 0.00 | 0.00 | 0.05 | 0.03 | 0.02 | 0.02 | 0.05 | 0.03 | 0.02 | 0.02 |
| γ*_g_*_9×E_ | γ*_b_*_36_ | 0 | 0.00 | 0.00 | 0.00 | 0.00 | 0.05 | 0.03 | 0.02 | 0.02 | 0.05 | 0.03 | 0.02 | 0.02 |
| γ*_g_*_1_ | γ*_b_*_37_ | 0 | 0.00 | 0.00 | 0.00 | 0.00 | 0.05 | 0.03 | 0.02 | 0.02 | 0.05 | 0.03 | 0.02 | 0.02 |
| γ*_g_*_2_ | γ*_b_*_37_ | 0 | 0.00 | 0.00 | 0.00 | 0.00 | 0.05 | 0.03 | 0.02 | 0.02 | 0.05 | 0.03 | 0.02 | 0.02 |
| γ*_g_*_3_ | γ*_b_*_37_ | 0 | -0.04 | -0.02 | -0.01 | -0.01 | 0.05 | 0.04 | 0.02 | 0.02 | 0.06 | 0.04 | 0.03 | 0.02 |
| γ*_g_*_4_ | γ*_b_*_37_ | 0.6 | -0.01 | 0.00 | 0.00 | 0.00 | 0.05 | 0.04 | 0.03 | 0.02 | 0.05 | 0.04 | 0.03 | 0.02 |
| γ*_g_*_5_ | γ*_b_*_37_ | 0.4 | 0.05 | 0.03 | 0.02 | 0.01 | 0.04 | 0.03 | 0.02 | 0.02 | 0.07 | 0.04 | 0.03 | 0.02 |
| γ*_g_*_6_ | γ*_b_*_37_ | 0 | 0.00 | 0.00 | 0.00 | 0.00 | 0.05 | 0.04 | 0.03 | 0.02 | 0.05 | 0.04 | 0.03 | 0.02 |
| γ*_g_*_7_ | γ*_b_*_37_ | 0 | -0.01 | 0.00 | 0.00 | 0.00 | 0.05 | 0.04 | 0.03 | 0.02 | 0.05 | 0.04 | 0.03 | 0.02 |
| γ*_g_*_8_ | γ*_b_*_37_ | 0 | -0.01 | 0.00 | 0.00 | 0.00 | 0.05 | 0.04 | 0.03 | 0.02 | 0.05 | 0.04 | 0.03 | 0.02 |
| γ*_g_*_9_ | γ*_b_*_37_ | 0 | 0.00 | 0.00 | 0.00 | 0.01 | 0.05 | 0.04 | 0.03 | 0.02 | 0.05 | 0.04 | 0.03 | 0.02 |
| z_E_ | γ*_b_*_37_ | 0.2 | 0.00 | 0.00 | 0.00 | 0.00 | 0.05 | 0.04 | 0.03 | 0.02 | 0.05 | 0.04 | 0.03 | 0.02 |
| γ*_g_*_1×E_ | γ*_b_*_37_ | 0 | 0.00 | 0.00 | 0.00 | 0.00 | 0.05 | 0.04 | 0.03 | 0.02 | 0.05 | 0.04 | 0.03 | 0.02 |
| γ*_g_*_2×E_ | γ*_b_*_37_ | 0 | 0.00 | 0.00 | 0.00 | 0.00 | 0.05 | 0.04 | 0.03 | 0.02 | 0.05 | 0.04 | 0.03 | 0.02 |
| γ*_g_*_3×E_ | γ*_b_*_37_ | 0 | -0.02 | -0.01 | 0.00 | 0.00 | 0.05 | 0.04 | 0.03 | 0.02 | 0.05 | 0.04 | 0.03 | 0.02 |
| γ*_g_*_4×E_ | γ*_b_*_37_ | -0.4 | 0.01 | 0.00 | 0.00 | 0.00 | 0.05 | 0.04 | 0.03 | 0.02 | 0.05 | 0.04 | 0.03 | 0.02 |
| γ*_g_*_5×E_ | γ*_b_*_37_ | 0 | 0.00 | 0.00 | 0.00 | 0.00 | 0.05 | 0.04 | 0.03 | 0.02 | 0.05 | 0.04 | 0.03 | 0.02 |
| γ*_g_*_6×E_ | γ*_b_*_37_ | 0 | 0.01 | 0.00 | 0.01 | 0.00 | 0.05 | 0.04 | 0.03 | 0.02 | 0.05 | 0.04 | 0.03 | 0.02 |
| γ*_g_*_7×E_ | γ*_b_*_37_ | 0 | 0.00 | 0.00 | 0.00 | 0.00 | 0.05 | 0.04 | 0.03 | 0.02 | 0.05 | 0.04 | 0.03 | 0.02 |
| γ*_g_*_8×E_ | γ*_b_*_37_ | 0 | 0.00 | 0.00 | 0.00 | 0.00 | 0.05 | 0.04 | 0.03 | 0.02 | 0.05 | 0.04 | 0.03 | 0.02 |
| γ*_g_*_9×E_ | γ*_b_*_37_ | 0 | 0.00 | 0.00 | 0.00 | 0.00 | 0.05 | 0.04 | 0.03 | 0.02 | 0.05 | 0.04 | 0.03 | 0.02 |
| γ*_g_*_1_ | γ*_b_*_38_ | 0 | 0.00 | 0.00 | 0.00 | 0.00 | 0.05 | 0.04 | 0.03 | 0.02 | 0.05 | 0.04 | 0.03 | 0.02 |
| γ*_g_*_2_ | γ*_b_*_38_ | 0 | 0.00 | 0.00 | 0.00 | 0.00 | 0.05 | 0.04 | 0.03 | 0.02 | 0.05 | 0.04 | 0.03 | 0.02 |
| γ*_g_*_3_ | γ*_b_*_38_ | 0 | 0.04 | 0.02 | 0.01 | 0.01 | 0.04 | 0.03 | 0.02 | 0.04 | 0.06 | 0.04 | 0.02 | 0.04 |
| γ*_g_*_4_ | γ*_b_*_38_ | 0 | -0.01 | 0.00 | 0.00 | 0.00 | 0.04 | 0.03 | 0.02 | 0.02 | 0.05 | 0.03 | 0.02 | 0.02 |
| γ*_g_*_5_ | γ*_b_*_38_ | -0.6 | -0.01 | 0.00 | 0.00 | 0.00 | 0.05 | 0.03 | 0.02 | 0.02 | 0.05 | 0.03 | 0.02 | 0.02 |
| γ*_g_*_6_ | γ*_b_*_38_ | 0.4 | 0.00 | 0.00 | 0.00 | 0.00 | 0.04 | 0.03 | 0.02 | 0.02 | 0.04 | 0.03 | 0.02 | 0.02 |
| γ*_g_*_7_ | γ*_b_*_38_ | 0 | 0.00 | 0.00 | 0.00 | 0.00 | 0.04 | 0.03 | 0.02 | 0.02 | 0.04 | 0.03 | 0.02 | 0.02 |
| γ*_g_*_8_ | γ*_b_*_38_ | 0 | 0.00 | 0.00 | 0.00 | 0.00 | 0.04 | 0.03 | 0.02 | 0.02 | 0.05 | 0.03 | 0.02 | 0.02 |
| γ*_g_*_9_ | γ*_b_*_38_ | 0 | 0.00 | 0.00 | 0.00 | 0.00 | 0.04 | 0.03 | 0.02 | 0.02 | 0.04 | 0.03 | 0.02 | 0.02 |
| z_E_ | γ*_b_*_38_ | 0 | 0.00 | 0.00 | 0.00 | 0.00 | 0.04 | 0.03 | 0.02 | 0.02 | 0.04 | 0.03 | 0.02 | 0.02 |
| γ*_g_*_1×E_ | γ*_b_*_38_ | 0 | 0.00 | 0.00 | 0.00 | 0.00 | 0.04 | 0.03 | 0.02 | 0.02 | 0.04 | 0.03 | 0.02 | 0.02 |
| γ*_g_*_2×E_ | γ*_b_*_38_ | 0 | -0.01 | -0.01 | 0.00 | 0.00 | 0.05 | 0.03 | 0.03 | 0.02 | 0.05 | 0.03 | 0.03 | 0.02 |
| γ*_g_*_3×E_ | γ*_b_*_38_ | 0 | -0.04 | -0.02 | -0.01 | -0.01 | 0.03 | 0.02 | 0.04 | 0.01 | 0.05 | 0.03 | 0.04 | 0.01 |
| γ*_g_*_4×E_ | γ*_b_*_38_ | 0 | -0.02 | -0.01 | 0.00 | 0.00 | 0.03 | 0.02 | 0.03 | 0.01 | 0.04 | 0.02 | 0.03 | 0.01 |
| γ*_g_*_5×E_ | γ*_b_*_38_ | 0.2 | 0.01 | 0.01 | 0.00 | 0.00 | 0.03 | 0.02 | 0.01 | 0.01 | 0.03 | 0.02 | 0.01 | 0.01 |
| γ*_g_*_6×E_ | γ*_b_*_38_ | -0.4 | 0.00 | 0.00 | 0.00 | 0.00 | 0.03 | 0.02 | 0.01 | 0.01 | 0.03 | 0.02 | 0.01 | 0.01 |
| γ*_g_*_7×E_ | γ*_b_*_38_ | 0 | 0.00 | 0.00 | 0.00 | 0.00 | 0.03 | 0.02 | 0.01 | 0.01 | 0.03 | 0.02 | 0.01 | 0.01 |
| γ*_g_*_8×E_ | γ*_b_*_38_ | 0 | 0.00 | 0.00 | 0.00 | 0.00 | 0.03 | 0.02 | 0.01 | 0.01 | 0.03 | 0.02 | 0.01 | 0.01 |
| γ*_g_*_9×E_ | γ*_b_*_38_ | 0 | 0.00 | 0.00 | 0.00 | 0.00 | 0.03 | 0.02 | 0.01 | 0.01 | 0.03 | 0.02 | 0.01 | 0.01 |
| γ*_g_*_1_ | γ*_b_*_39_ | 0 | 0.00 | 0.00 | 0.00 | 0.00 | 0.03 | 0.02 | 0.01 | 0.01 | 0.03 | 0.02 | 0.01 | 0.01 |
| γ*_g_*_2_ | γ*_b_*_39_ | 0 |  | 0.00 | 0.00 | 0.00 | 0.03 | 0.02 | 0.01 | 0.01 | 0.03 | 0.02 | 0.01 | 0.01 |
| γ*_g_*_3_ | γ*_b_*_39_ | 0 | -0.02 | -0.01 | -0.01 | 0.00 | 0.03 | 0.02 | 0.02 | 0.01 | 0.04 | 0.03 | 0.02 | 0.01 |
| γ*_g_*_4_ | γ*_b_*_39_ | 0.4 | 0.03 | 0.01 | 0.01 | 0.00 | 0.03 | 0.02 | 0.03 | 0.01 | 0.04 | 0.03 | 0.03 | 0.01 |
| γ*_g_*_5_ | γ*_b_*_39_ | 0 | -0.01 | 0.00 | 0.00 | 0.00 | 0.03 | 0.02 | 0.01 | 0.01 | 0.03 | 0.02 | 0.01 | 0.01 |
| γ*_g_*_6_ | γ*_b_*_39_ | -0.6 | -0.01 | 0.00 | 0.00 | 0.00 | 0.03 | 0.02 | 0.01 | 0.01 | 0.03 | 0.02 | 0.01 | 0.01 |
| γ*_g_*_7_ | γ*_b_*_39_ | 0 | 0.00 | 0.00 | 0.00 | 0.00 | 0.03 | 0.02 | 0.01 | 0.01 | 0.03 | 0.02 | 0.01 | 0.01 |
| γ*_g_*_8_ | γ*_b_*_39_ | 0 | 0.00 | 0.00 | 0.00 | 0.00 | 0.03 | 0.02 | 0.01 | 0.01 | 0.03 | 0.02 | 0.01 | 0.01 |
| γ*_g_*_9_ | γ*_b_*_39_ | 0 | 0.00 | 0.00 | 0.00 | 0.00 | 0.03 | 0.02 | 0.01 | 0.01 | 0.03 | 0.02 | 0.01 | 0.01 |
| z_E_ | γ*_b_*_39_ | 0 | 0.00 | 0.00 | 0.00 | 0.00 | 0.03 | 0.02 | 0.01 | 0.01 | 0.03 | 0.02 | 0.01 | 0.01 |
| γ*_g_*_1×E_ | γ*_b_*_39_ | 0 | 0.00 | 0.00 | 0.00 | 0.00 | 0.03 | 0.02 | 0.01 | 0.01 | 0.03 | 0.02 | 0.01 | 0.01 |
| γ*_g_*_2×E_ | γ*_b_*_39_ | 0 | 0.00 | 0.00 | 0.00 | 0.00 | 0.03 | 0.02 | 0.01 | 0.01 | 0.03 | 0.02 | 0.01 | 0.01 |
| γ*_g_*_3×E_ | γ*_b_*_39_ | 0 | -0.01 | 0.00 | 0.00 | 0.00 | 0.05 | 0.03 | 0.02 | 0.02 | 0.05 | 0.03 | 0.02 | 0.02 |
| γ*_g_*_4×E_ | γ*_b_*_39_ | 0.2 | 0.05 | 0.02 | 0.01 | 0.01 | 0.04 | 0.05 | 0.04 | 0.04 | 0.06 | 0.05 | 0.05 | 0.04 |
| γ*_g_*_5×E_ | γ*_b_*_39_ | 0 | -0.04 | -0.02 | -0.01 | -0.01 | 0.05 | 0.04 | 0.04 | 0.03 | 0.06 | 0.05 | 0.04 | 0.03 |
| γ*_g_*_6×E_ | γ*_b_*_39_ | 0 | 0.01 | 0.00 | 0.00 | 0.00 | 0.04 | 0.03 | 0.02 | 0.02 | 0.04 | 0.03 | 0.02 | 0.02 |
| γ*_g_*_7×E_ | γ*_b_*_39_ | 0 | 0.00 | 0.00 | 0.00 | 0.00 | 0.04 | 0.03 | 0.02 | 0.02 | 0.04 | 0.03 | 0.02 | 0.02 |
| γ*_g_*_8×E_ | γ*_b_*_39_ | 0 | 0.00 | 0.00 | 0.00 | 0.00 | 0.05 | 0.03 | 0.02 | 0.02 | 0.05 | 0.03 | 0.02 | 0.02 |
| γ*_g_*_9×E_ | γ*_b_*_39_ | 0 | 0.00 | 0.00 | 0.00 | 0.00 | 0.04 | 0.03 | 0.02 | 0.02 | 0.04 | 0.03 | 0.02 | 0.02 |
| γ*_g_*_1_ | γ*_b_*_40_ | 0 | 0.00 | 0.00 | 0.00 | 0.00 | 0.05 | 0.03 | 0.02 | 0.02 | 0.05 | 0.03 | 0.02 | 0.02 |
| γ*_g_*_2_ | γ*_b_*_40_ | 0 | 0.00 | 0.00 | 0.00 | 0.00 | 0.05 | 0.03 | 0.02 | 0.02 | 0.05 | 0.03 | 0.02 | 0.02 |
| γ*_g_*_3_ | γ*_b_*_40_ | 0 | 0.00 | 0.00 | 0.00 | 0.00 | 0.05 | 0.04 | 0.02 | 0.02 | 0.05 | 0.04 | 0.02 | 0.02 |
| γ*_g_*_4_ | γ*_b_*_40_ | -0.6 | 0.00 | 0.00 | 0.00 | 0.00 | 0.05 | 0.03 | 0.02 | 0.02 | 0.05 | 0.03 | 0.02 | 0.02 |
| γ*_g_*_5_ | γ*_b_*_40_ | 0 | -0.02 | -0.01 | -0.01 | 0.00 | 0.05 | 0.04 | 0.03 | 0.02 | 0.05 | 0.04 | 0.03 | 0.02 |
| γ*_g_*_6_ | γ*_b_*_40_ | 0 | 0.03 | 0.02 | 0.01 | 0.00 | 0.05 | 0.04 | 0.04 | 0.03 | 0.06 | 0.05 | 0.04 | 0.03 |
| γ*_g_*_7_ | γ*_b_*_40_ | 0 | -0.01 | 0.00 | -0.01 | 0.00 | 0.05 | 0.03 | 0.02 | 0.02 | 0.05 | 0.03 | 0.03 | 0.02 |
| γ*_g_*_8_ | γ*_b_*_40_ | 0 | 0.00 | 0.00 | 0.00 | 0.00 | 0.05 | 0.03 | 0.02 | 0.02 | 0.05 | 0.03 | 0.02 | 0.02 |
| γ*_g_*_9_ | γ*_b_*_40_ | 0 | 0.00 | 0.00 | 0.00 | 0.00 | 0.05 | 0.03 | 0.02 | 0.02 | 0.05 | 0.03 | 0.02 | 0.02 |
| z_E_ | γ*_b_*_40_ | 0.2 | 0.00 | 0.00 | 0.00 | 0.00 | 0.05 | 0.03 | 0.02 | 0.02 | 0.05 | 0.03 | 0.02 | 0.02 |
| γ*_g_*_1×E_ | γ*_b_*_40_ | 0 | 0.00 | 0.00 | 0.00 | 0.00 | 0.05 | 0.04 | 0.02 | 0.02 | 0.05 | 0.04 | 0.02 | 0.02 |
| γ*_g_*_2×E_ | γ*_b_*_40_ | 0 | 0.00 | 0.00 | 0.00 | 0.00 | 0.05 | 0.03 | 0.02 | 0.02 | 0.05 | 0.03 | 0.02 | 0.02 |
| γ*_g_*_3×E_ | γ*_b_*_40_ | 0 | -0.04 | -0.02 | -0.01 | -0.01 | 0.05 | 0.04 | 0.03 | 0.03 | 0.06 | 0.04 | 0.03 | 0.03 |
| γ*_g_*_4×E_ | γ*_b_*_40_ | 0.4 | 0.00 | 0.00 | 0.00 | 0.00 | 0.05 | 0.04 | 0.03 | 0.02 | 0.05 | 0.04 | 0.03 | 0.02 |
| γ*_g_*_5×E_ | γ*_b_*_40_ | 0 | 0.05 | 0.02 | 0.01 | 0.01 | 0.04 | 0.03 | 0.02 | 0.04 | 0.06 | 0.04 | 0.02 | 0.04 |
| γ*_g_*_6×E_ | γ*_b_*_40_ | 0 | 0.00 | 0.00 | 0.00 | 0.00 | 0.05 | 0.04 | 0.03 | 0.02 | 0.05 | 0.04 | 0.03 | 0.02 |
| γ*_g_*_7×E_ | γ*_b_*_40_ | 0 | 0.00 | 0.00 | 0.00 | 0.00 | 0.05 | 0.04 | 0.03 | 0.02 | 0.05 | 0.04 | 0.03 | 0.02 |
| γ*_g_*_8×E_ | γ*_b_*_40_ | 0 | 0.01 | 0.01 | 0.01 | 0.00 | 0.05 | 0.04 | 0.03 | 0.02 | 0.05 | 0.04 | 0.03 | 0.02 |
| γ*_g_*_9×E_ | γ*_b_*_40_ | 0 | 0.00 | 0.00 | 0.00 | 0.00 | 0.05 | 0.04 | 0.03 | 0.02 | 0.05 | 0.04 | 0.03 | 0.02 |
| γ*_g_*_1_ | γ*_b_*_41_ | 0 | 0.00 | 0.00 | 0.00 | 0.00 | 0.05 | 0.04 | 0.03 | 0.02 | 0.05 | 0.04 | 0.03 | 0.02 |
| γ*_g_*_2_ | γ*_b_*_41_ | 0 | 0.00 | 0.00 | 0.00 | 0.00 | 0.05 | 0.04 | 0.03 | 0.02 | 0.05 | 0.04 | 0.03 | 0.02 |
| γ*_g_*_3_ | γ*_b_*_41_ | 0 | 0.00 | 0.00 | 0.00 | 0.00 | 0.05 | 0.03 | 0.03 | 0.02 | 0.05 | 0.03 | 0.03 | 0.02 |
| γ*_g_*_4_ | γ*_b_*_41_ | 0 | -0.02 | -0.01 | -0.01 | -0.01 | 0.05 | 0.04 | 0.03 | 0.02 | 0.05 | 0.04 | 0.03 | 0.02 |
| γ*_g_*_5_ | γ*_b_*_41_ | 0 | 0.01 | 0.00 | 0.00 | 0.00 | 0.05 | 0.04 | 0.03 | 0.02 | 0.05 | 0.04 | 0.03 | 0.02 |
| γ*_g_*_6_ | γ*_b_*_41_ | 0 | 0.00 | 0.00 | 0.00 | 0.00 | 0.05 | 0.04 | 0.03 | 0.02 | 0.05 | 0.04 | 0.03 | 0.02 |
| γ*_g_*_7_ | γ*_b_*_41_ | 0.6 | 0.01 | 0.01 | 0.01 | 0.01 | 0.05 | 0.04 | 0.03 | 0.02 | 0.05 | 0.04 | 0.03 | 0.02 |
| γ*_g_*_8_ | γ*_b_*_41_ | 0.4 | 0.00 | 0.00 | 0.00 | 0.00 | 0.05 | 0.04 | 0.03 | 0.02 | 0.05 | 0.04 | 0.03 | 0.02 |
| γ*_g_*_9_ | γ*_b_*_41_ | 0 | 0.00 | 0.00 | 0.00 | 0.00 | 0.05 | 0.04 | 0.03 | 0.02 | 0.05 | 0.04 | 0.03 | 0.02 |
| z_E_ | γ*_b_*_41_ | 0.2 | 0.00 | 0.00 | 0.00 | 0.00 | 0.05 | 0.04 | 0.03 | 0.02 | 0.05 | 0.04 | 0.03 | 0.02 |
| γ*_g_*_1×E_ | γ*_b_*_41_ | 0 | 0.00 | 0.01 | 0.00 | 0.00 | 0.05 | 0.04 | 0.03 | 0.02 | 0.05 | 0.04 | 0.03 | 0.02 |
| γ*_g_*_2×E_ | γ*_b_*_41_ | 0 | 0.00 | 0.00 | 0.00 | 0.00 | 0.05 | 0.04 | 0.03 | 0.02 | 0.05 | 0.04 | 0.03 | 0.02 |
| γ*_g_*_3×E_ | γ*_b_*_41_ | 0 | 0.04 | 0.02 | 0.01 | 0.00 | 0.04 | 0.05 | 0.04 | 0.04 | 0.06 | 0.05 | 0.04 | 0.04 |
| γ*_g_*_4×E_ | γ*_b_*_41_ | 0 | -0.01 | -0.01 | 0.00 | 0.00 | 0.04 | 0.03 | 0.02 | 0.02 | 0.04 | 0.03 | 0.02 | 0.02 |
| γ*_g_*_5×E_ | γ*_b_*_41_ | 0 | -0.01 | 0.00 | 0.00 | 0.00 | 0.04 | 0.03 | 0.02 | 0.02 | 0.05 | 0.03 | 0.02 | 0.02 |
| γ*_g_*_6×E_ | γ*_b_*_41_ | 0 | 0.00 | 0.00 | 0.00 | 0.00 | 0.04 | 0.03 | 0.02 | 0.02 | 0.04 | 0.03 | 0.02 | 0.02 |
| γ*_g_*_7×E_ | γ*_b_*_41_ | -0.4 | 0.00 | 0.00 | 0.00 | 0.00 | 0.04 | 0.03 | 0.02 | 0.02 | 0.04 | 0.03 | 0.02 | 0.02 |
| γ*_g_*_8×E_ | γ*_b_*_41_ | 0 | 0.00 | 0.00 | 0.00 | 0.00 | 0.04 | 0.03 | 0.02 | 0.02 | 0.04 | 0.03 | 0.02 | 0.02 |
| γ*_g_*_9×E_ | γ*_b_*_41_ | 0 | 0.00 | 0.00 | 0.00 | 0.00 | 0.04 | 0.03 | 0.02 | 0.02 | 0.04 | 0.03 | 0.02 | 0.02 |
| γ*_g_*_1_ | γ*_b_*_42_ | 0 | 0.00 | 0.00 | 0.00 | 0.00 | 0.04 | 0.03 | 0.02 | 0.02 | 0.04 | 0.03 | 0.02 | 0.02 |
| γ*_g_*_2_ | γ*_b_*_42_ | 0 | -0.01 | -0.01 | -0.01 | -0.01 | 0.04 | 0.03 | 0.02 | 0.02 | 0.04 | 0.03 | 0.02 | 0.02 |
| γ*_g_*_3_ | γ*_b_*_42_ | 0 | -0.02 | -0.01 | -0.01 | -0.01 | 0.05 | 0.04 | 0.03 | 0.02 | 0.05 | 0.04 | 0.03 | 0.02 |
| γ*_g_*_4_ | γ*_b_*_42_ | 0 | -0.03 | -0.02 | -0.01 | -0.01 | 0.05 | 0.04 | 0.03 | 0.03 | 0.05 | 0.04 | 0.04 | 0.03 |
| γ*_g_*_5_ | γ*_b_*_42_ | 0 | 0.01 | 0.00 | 0.00 | 0.00 | 0.05 | 0.03 | 0.02 | 0.02 | 0.05 | 0.03 | 0.02 | 0.02 |
| γ*_g_*_6_ | γ*_b_*_42_ | 0 | 0.00 | 0.00 | 0.00 | 0.00 | 0.05 | 0.03 | 0.02 | 0.02 | 0.05 | 0.03 | 0.02 | 0.02 |
| γ*_g_*_7_ | γ*_b_*_42_ | 0 | 0.00 | 0.00 | 0.00 | 0.00 | 0.04 | 0.03 | 0.02 | 0.02 | 0.04 | 0.03 | 0.02 | 0.02 |
| γ*_g_*_8_ | γ*_b_*_42_ | -0.6 | 0.01 | 0.01 | 0.01 | 0.01 | 0.05 | 0.03 | 0.02 | 0.02 | 0.05 | 0.03 | 0.02 | 0.02 |
| γ*_g_*_9_ | γ*_b_*_42_ | 0.4 | 0.00 | 0.00 | 0.00 | 0.00 | 0.05 | 0.03 | 0.02 | 0.02 | 0.05 | 0.03 | 0.02 | 0.02 |
| z_E_ | γ*_b_*_42_ | 0 | 0.00 | 0.00 | 0.00 | 0.00 | 0.05 | 0.03 | 0.02 | 0.02 | 0.05 | 0.03 | 0.02 | 0.02 |
| γ*_g_*_1×E_ | γ*_b_*_42_ | 0 | 0.00 | 0.00 | 0.00 | 0.00 | 0.04 | 0.03 | 0.02 | 0.02 | 0.04 | 0.03 | 0.02 | 0.02 |
| γ*_g_*_2×E_ | γ*_b_*_42_ | 0 | 0.00 | 0.00 | 0.00 | 0.00 | 0.05 | 0.03 | 0.02 | 0.02 | 0.05 | 0.03 | 0.02 | 0.02 |
| γ*_g_*_3×E_ | γ*_b_*_42_ | 0 | -0.04 | -0.02 | -0.01 | 0.00 | 0.03 | 0.02 | 0.02 | 0.01 | 0.05 | 0.03 | 0.02 | 0.01 |
| γ*_g_*_4×E_ | γ*_b_*_42_ | 0 | -0.02 | -0.01 | 0.00 | 0.00 | 0.03 | 0.02 | 0.02 | 0.01 | 0.03 | 0.02 | 0.02 | 0.01 |
| γ*_g_*_5×E_ | γ*_b_*_42_ | 0 | 0.02 | 0.01 | 0.00 | 0.00 | 0.03 | 0.02 | 0.01 | 0.01 | 0.03 | 0.02 | 0.01 | 0.01 |
| γ*_g_*_6×E_ | γ*_b_*_42_ | 0 | 0.00 | 0.00 | 0.00 | 0.00 | 0.03 | 0.02 | 0.01 | 0.01 | 0.03 | 0.02 | 0.01 | 0.01 |
| γ*_g_*_7×E_ | γ*_b_*_42_ | 0 | 0.00 | 0.00 | 0.00 | 0.00 | 0.03 | 0.02 | 0.01 | 0.01 | 0.03 | 0.02 | 0.01 | 0.01 |
| γ*_g_*_8×E_ | γ*_b_*_42_ | 0.2 | 0.00 | 0.00 | 0.00 | 0.00 | 0.03 | 0.02 | 0.01 | 0.01 | 0.03 | 0.02 | 0.01 | 0.01 |
| γ*_g_*_9×E_ | γ*_b_*_42_ | -0.4 | 0.00 | 0.00 | 0.00 | 0.00 | 0.03 | 0.02 | 0.01 | 0.01 | 0.03 | 0.02 | 0.01 | 0.01 |
| γ*_g_*_1_ | γ*_b_*_43_ | 0 | 0.00 | 0.00 | 0.00 | 0.00 | 0.03 | 0.02 | 0.01 | 0.01 | 0.03 | 0.02 | 0.01 | 0.01 |
| γ*_g_*_2_ | γ*_b_*_43_ | 0 | 0.00 | 0.00 | 0.00 | 0.00 | 0.03 | 0.02 | 0.01 | 0.01 | 0.03 | 0.02 | 0.01 | 0.01 |
| γ*_g_*_3_ | γ*_b_*_43_ | 0 | -0.02 | -0.01 | -0.01 | 0.00 | 0.03 | 0.02 | 0.02 | 0.01 | 0.04 | 0.03 | 0.02 | 0.01 |
| γ*_g_*_4_ | γ*_b_*_43_ | 0 | 0.03 | 0.02 | 0.01 | 0.01 | 0.03 | 0.02 | 0.02 | 0.01 | 0.04 | 0.03 | 0.02 | 0.01 |
| γ*_g_*_5_ | γ*_b_*_43_ | 0 | -0.01 | 0.00 | 0.00 | 0.00 | 0.03 | 0.02 | 0.01 | 0.01 | 0.03 | 0.02 | 0.01 | 0.01 |
| γ*_g_*_6_ | γ*_b_*_43_ | 0 | -0.01 | 0.00 | 0.00 | 0.00 | 0.03 | 0.02 | 0.01 | 0.01 | 0.03 | 0.02 | 0.01 | 0.01 |
| γ*_g_*_7_ | γ*_b_*_43_ | 0.4 | 0.00 | 0.00 | 0.00 | 0.00 | 0.03 | 0.02 | 0.01 | 0.01 | 0.03 | 0.02 | 0.01 | 0.01 |
| γ*_g_*_8_ | γ*_b_*_43_ | 0 | 0.00 | 0.00 | 0.00 | 0.00 | 0.03 | 0.02 | 0.01 | 0.01 | 0.03 | 0.02 | 0.01 | 0.01 |
| γ*_g_*_9_ | γ*_b_*_43_ | -0.6 | 0.00 | 0.00 | 0.00 | 0.00 | 0.03 | 0.02 | 0.01 | 0.01 | 0.03 | 0.02 | 0.01 | 0.01 |
| z_E_ | γ*_b_*_43_ | 0 | 0.00 | 0.00 | 0.00 | 0.00 | 0.03 | 0.02 | 0.01 | 0.01 | 0.03 | 0.02 | 0.01 | 0.01 |
| γ*_g_*_1×E_ | γ*_b_*_43_ | 0 | 0.00 | 0.00 | 0.00 | 0.00 | 0.03 | 0.02 | 0.01 | 0.01 | 0.03 | 0.02 | 0.01 | 0.01 |
| γ*_g_*_2×E_ | γ*_b_*_43_ | 0 | 0.00 | 0.00 | 0.00 | 0.00 | 0.03 | 0.02 | 0.01 | 0.01 | 0.03 | 0.02 | 0.01 | 0.01 |
| γ*_g_*_3×E_ | γ*_b_*_43_ | 0 | 0.00 | 0.00 | 0.00 | 0.00 | 0.05 | 0.03 | 0.02 | 0.02 | 0.05 | 0.03 | 0.02 | 0.02 |
| γ*_g_*_4×E_ | γ*_b_*_43_ | 0 | 0.05 | 0.02 | 0.01 | 0.00 | 0.07 | 0.05 | 0.02 | 0.02 | 0.09 | 0.05 | 0.02 | 0.02 |
| γ*_g_*_5×E_ | γ*_b_*_43_ | 0 | -0.04 | -0.02 | -0.01 | -0.01 | 0.06 | 0.04 | 0.03 | 0.02 | 0.07 | 0.05 | 0.03 | 0.02 |
| γ*_g_*_6×E_ | γ*_b_*_43_ | 0 | 0.00 | 0.00 | -0.01 | 0.00 | 0.05 | 0.03 | 0.02 | 0.02 | 0.05 | 0.03 | 0.02 | 0.02 |
| γ*_g_*_7×E_ | γ*_b_*_43_ | 0.2 | 0.00 | 0.00 | 0.00 | 0.00 | 0.05 | 0.03 | 0.03 | 0.02 | 0.05 | 0.03 | 0.03 | 0.02 |
| γ*_g_*_8×E_ | γ*_b_*_43_ | 0 | 0.00 | 0.00 | 0.00 | 0.00 | 0.05 | 0.03 | 0.02 | 0.02 | 0.05 | 0.03 | 0.02 | 0.02 |
| γ*_g_*_9×E_ | γ*_b_*_43_ | 0 | 0.00 | 0.00 | 0.00 | 0.00 | 0.04 | 0.03 | 0.02 | 0.02 | 0.04 | 0.03 | 0.02 | 0.02 |
| γ*_g_*_1_ | γ*_b_*_44_ | 0 | 0.00 | 0.00 | 0.00 | 0.00 | 0.05 | 0.03 | 0.02 | 0.02 | 0.05 | 0.03 | 0.02 | 0.02 |
| γ*_g_*_2_ | γ*_b_*_44_ | 0 | 0.00 | 0.00 | 0.00 | 0.00 | 0.05 | 0.04 | 0.02 | 0.02 | 0.05 | 0.04 | 0.02 | 0.02 |
| γ*_g_*_3_ | γ*_b_*_44_ | 0 | 0.00 | 0.00 | 0.00 | 0.00 | 0.05 | 0.04 | 0.02 | 0.02 | 0.05 | 0.04 | 0.03 | 0.02 |
| γ*_g_*_4_ | γ*_b_*_44_ | 0 | 0.00 | 0.00 | 0.00 | 0.01 | 0.05 | 0.04 | 0.02 | 0.02 | 0.05 | 0.04 | 0.02 | 0.02 |
| γ*_g_*_5_ | γ*_b_*_44_ | 0 | -0.03 | -0.01 | -0.01 | 0.00 | 0.05 | 0.04 | 0.02 | 0.02 | 0.06 | 0.04 | 0.02 | 0.02 |
| γ*_g_*_6_ | γ*_b_*_44_ | 0 | 0.04 | 0.02 | 0.01 | 0.01 | 0.06 | 0.04 | 0.03 | 0.02 | 0.07 | 0.05 | 0.03 | 0.02 |
| γ*_g_*_7_ | γ*_b_*_44_ | -0.6 | 0.00 | 0.00 | 0.00 | 0.00 | 0.05 | 0.03 | 0.02 | 0.02 | 0.05 | 0.03 | 0.02 | 0.02 |
| γ*_g_*_8_ | γ*_b_*_44_ | 0 | 0.00 | 0.00 | 0.00 | 0.00 | 0.05 | 0.03 | 0.02 | 0.02 | 0.05 | 0.03 | 0.02 | 0.02 |
| γ*_g_*_9_ | γ*_b_*_44_ | 0 | 0.00 | 0.00 | 0.00 | 0.00 | 0.05 | 0.03 | 0.02 | 0.02 | 0.05 | 0.03 | 0.02 | 0.02 |
| z_E_ | γ*_b_*_44_ | 0.2 | 0.00 | 0.00 | 0.00 | 0.00 | 0.05 | 0.03 | 0.02 | 0.02 | 0.05 | 0.03 | 0.02 | 0.02 |
| γ*_g_*_1×E_ | γ*_b_*_44_ | 0 | 0.00 | 0.00 | 0.00 | 0.00 | 0.05 | 0.03 | 0.02 | 0.02 | 0.05 | 0.03 | 0.02 | 0.02 |
| γ*_g_*_2×E_ | γ*_b_*_44_ | 0 | 0.00 | 0.00 | 0.00 | 0.00 | 0.05 | 0.03 | 0.02 | 0.02 | 0.05 | 0.03 | 0.02 | 0.02 |
| γ*_g_*_3×E_ | γ*_b_*_44_ | 0 | -0.04 | -0.02 | -0.01 | -0.01 | 0.05 | 0.04 | 0.02 | 0.02 | 0.06 | 0.04 | 0.03 | 0.02 |
| γ*_g_*_4×E_ | γ*_b_*_44_ | 0 | -0.01 | 0.00 | 0.00 | 0.00 | 0.05 | 0.04 | 0.03 | 0.02 | 0.05 | 0.04 | 0.03 | 0.02 |
| γ*_g_*_5×E_ | γ*_b_*_44_ | 0 | 0.05 | 0.03 | 0.02 | 0.01 | 0.04 | 0.03 | 0.02 | 0.02 | 0.07 | 0.04 | 0.03 | 0.02 |
| γ*_g_*_6×E_ | γ*_b_*_44_ | 0 | 0.00 | 0.00 | 0.00 | 0.00 | 0.05 | 0.04 | 0.03 | 0.02 | 0.05 | 0.04 | 0.03 | 0.02 |
| γ*_g_*_7×E_ | γ*_b_*_44_ | 0.4 | -0.01 | 0.00 | 0.00 | 0.00 | 0.05 | 0.04 | 0.03 | 0.02 | 0.05 | 0.04 | 0.03 | 0.02 |
| γ*_g_*_8×E_ | γ*_b_*_44_ | 0 | -0.01 | 0.00 | 0.00 | 0.00 | 0.05 | 0.04 | 0.03 | 0.02 | 0.05 | 0.04 | 0.03 | 0.02 |
| γ*_g_*_9×E_ | γ*_b_*_44_ | 0 | 0.00 | 0.00 | 0.00 | 0.01 | 0.05 | 0.04 | 0.03 | 0.02 | 0.05 | 0.04 | 0.03 | 0.02 |
| γ*_g_*_1_ | γ*_b_*_45_ | 0 | 0.00 | 0.00 | 0.00 | 0.00 | 0.05 | 0.04 | 0.03 | 0.02 | 0.05 | 0.04 | 0.03 | 0.02 |
| γ*_g_*_2_ | γ*_b_*_45_ | 0 | 0.00 | 0.00 | 0.00 | 0.00 | 0.05 | 0.04 | 0.03 | 0.02 | 0.05 | 0.04 | 0.03 | 0.02 |
| γ*_g_*_3_ | γ*_b_*_45_ | 0 | 0.00 | 0.00 | 0.00 | 0.00 | 0.05 | 0.04 | 0.03 | 0.02 | 0.05 | 0.04 | 0.03 | 0.02 |
| γ*_g_*_4_ | γ*_b_*_45_ | 0 | -0.02 | -0.01 | 0.00 | 0.00 | 0.05 | 0.04 | 0.03 | 0.02 | 0.05 | 0.04 | 0.03 | 0.02 |
| γ*_g_*_5_ | γ*_b_*_45_ | 0 | 0.01 | 0.00 | 0.00 | 0.00 | 0.05 | 0.04 | 0.03 | 0.02 | 0.05 | 0.04 | 0.03 | 0.02 |
| γ*_g_*_6_ | γ*_b_*_45_ | 0 | 0.00 | 0.00 | 0.00 | 0.00 | 0.05 | 0.04 | 0.03 | 0.02 | 0.05 | 0.04 | 0.03 | 0.02 |
| γ*_g_*_7_ | γ*_b_*_45_ | 0.6 | 0.01 | 0.00 | 0.01 | 0.00 | 0.05 | 0.04 | 0.03 | 0.02 | 0.05 | 0.04 | 0.03 | 0.02 |
| γ*_g_*_8_ | γ*_b_*_45_ | 0.4 | 0.00 | 0.00 | 0.00 | 0.00 | 0.05 | 0.04 | 0.03 | 0.02 | 0.05 | 0.04 | 0.03 | 0.02 |
| γ*_g_*_9_ | γ*_b_*_45_ | 0 | 0.00 | 0.00 | 0.00 | 0.00 | 0.05 | 0.04 | 0.03 | 0.02 | 0.05 | 0.04 | 0.03 | 0.02 |
| z_E_ | γ*_b_*_45_ | 0.2 | 0.00 | 0.00 | 0.00 | 0.00 | 0.05 | 0.04 | 0.03 | 0.02 | 0.05 | 0.04 | 0.03 | 0.02 |
| γ*_g_*_1×E_ | γ*_b_*_45_ | 0 | 0.00 | 0.00 | 0.00 | 0.00 | 0.05 | 0.04 | 0.03 | 0.02 | 0.05 | 0.04 | 0.03 | 0.02 |
| γ*_g_*_2×E_ | γ*_b_*_45_ | 0 | 0.00 | 0.00 | 0.00 | 0.00 | 0.05 | 0.04 | 0.03 | 0.02 | 0.05 | 0.04 | 0.03 | 0.02 |
| γ*_g_*_3×E_ | γ*_b_*_45_ | 0 | 0.04 | 0.02 | 0.01 | 0.01 | 0.04 | 0.03 | 0.02 | 0.04 | 0.06 | 0.04 | 0.02 | 0.04 |
| γ*_g_*_4×E_ | γ*_b_*_45_ | 0 | -0.01 | 0.00 | 0.00 | 0.00 | 0.04 | 0.03 | 0.02 | 0.02 | 0.05 | 0.03 | 0.02 | 0.02 |
| γ*_g_*_5×E_ | γ*_b_*_45_ | 0 | -0.01 | 0.00 | 0.00 | 0.00 | 0.05 | 0.03 | 0.02 | 0.02 | 0.05 | 0.03 | 0.02 | 0.02 |
| γ*_g_*_6×E_ | γ*_b_*_45_ | 0 | 0.00 | 0.00 | 0.00 | 0.00 | 0.04 | 0.03 | 0.02 | 0.02 | 0.04 | 0.03 | 0.02 | 0.02 |
| γ*_g_*_7×E_ | γ*_b_*_45_ | -0.4 | 0.00 | 0.00 | 0.00 | 0.00 | 0.04 | 0.03 | 0.02 | 0.02 | 0.04 | 0.03 | 0.02 | 0.02 |
| γ*_g_*_8×E_ | γ*_b_*_45_ | 0 | 0.00 | 0.00 | 0.00 | 0.00 | 0.04 | 0.03 | 0.02 | 0.02 | 0.05 | 0.03 | 0.02 | 0.02 |
| γ*_g_*_9×E_ | γ*_b_*_45_ | 0 | 0.00 | 0.00 | 0.00 | 0.00 | 0.04 | 0.03 | 0.02 | 0.02 | 0.04 | 0.03 | 0.02 | 0.02 |
| γ*_g_*_1_ | γ*_b_*_46_ | 0 | 0.00 | 0.00 | 0.00 | 0.00 | 0.04 | 0.03 | 0.02 | 0.02 | 0.04 | 0.03 | 0.02 | 0.02 |
| γ*_g_*_2_ | γ*_b_*_46_ | 0 | 0.00 | 0.00 | 0.00 | 0.00 | 0.04 | 0.03 | 0.02 | 0.02 | 0.04 | 0.03 | 0.02 | 0.02 |
| γ*_g_*_3_ | γ*_b_*_46_ | 0 | -0.01 | -0.01 | 0.00 | 0.00 | 0.05 | 0.03 | 0.03 | 0.02 | 0.05 | 0.03 | 0.03 | 0.02 |
| γ*_g_*_4_ | γ*_b_*_46_ | 0 | -0.04 | -0.02 | -0.01 | -0.01 | 0.03 | 0.02 | 0.04 | 0.01 | 0.05 | 0.03 | 0.04 | 0.01 |
| γ*_g_*_5_ | γ*_b_*_46_ | 0 | -0.02 | -0.01 | 0.00 | 0.00 | 0.03 | 0.02 | 0.03 | 0.01 | 0.04 | 0.02 | 0.03 | 0.01 |
| γ*_g_*_6_ | γ*_b_*_46_ | 0 | 0.01 | 0.01 | 0.00 | 0.00 | 0.03 | 0.02 | 0.01 | 0.01 | 0.03 | 0.02 | 0.01 | 0.01 |
| γ*_g_*_7_ | γ*_b_*_46_ | 0 | 0.00 | 0.00 | 0.00 | 0.00 | 0.03 | 0.02 | 0.01 | 0.01 | 0.03 | 0.02 | 0.01 | 0.01 |
| γ*_g_*_8_ | γ*_b_*_46_ | -0.6 | 0.00 | 0.00 | 0.00 | 0.00 | 0.03 | 0.02 | 0.01 | 0.01 | 0.03 | 0.02 | 0.01 | 0.01 |
| γ*_g_*_9_ | γ*_b_*_46_ | 0.4 | 0.00 | 0.00 | 0.00 | 0.00 | 0.03 | 0.02 | 0.01 | 0.01 | 0.03 | 0.02 | 0.01 | 0.01 |
| z_E_ | γ*_b_*_46_ | 0 | 0.00 | 0.00 | 0.00 | 0.00 | 0.03 | 0.02 | 0.01 | 0.01 | 0.03 | 0.02 | 0.01 | 0.01 |
| γ*_g_*_1×E_ | γ*_b_*_46_ | 0 | 0.00 | 0.00 | 0.00 | 0.00 | 0.03 | 0.02 | 0.01 | 0.01 | 0.03 | 0.02 | 0.01 | 0.01 |
| γ*_g_*_2×E_ | γ*_b_*_46_ | 0 |  | 0.00 | 0.00 | 0.00 | 0.03 | 0.02 | 0.01 | 0.01 | 0.03 | 0.02 | 0.01 | 0.01 |
| γ*_g_*_3×E_ | γ*_b_*_46_ | 0 | -0.02 | -0.01 | -0.01 | 0.00 | 0.03 | 0.02 | 0.02 | 0.01 | 0.04 | 0.03 | 0.02 | 0.01 |
| γ*_g_*_4×E_ | γ*_b_*_46_ | 0 | 0.03 | 0.01 | 0.01 | 0.00 | 0.03 | 0.02 | 0.03 | 0.01 | 0.04 | 0.03 | 0.03 | 0.01 |
| γ*_g_*_5×E_ | γ*_b_*_46_ | 0 | -0.01 | 0.00 | 0.00 | 0.00 | 0.03 | 0.02 | 0.01 | 0.01 | 0.03 | 0.02 | 0.01 | 0.01 |
| γ*_g_*_6×E_ | γ*_b_*_46_ | 0 | -0.01 | 0.00 | 0.00 | 0.00 | 0.03 | 0.02 | 0.01 | 0.01 | 0.03 | 0.02 | 0.01 | 0.01 |
| γ*_g_*_7×E_ | γ*_b_*_46_ | 0 | 0.00 | 0.00 | 0.00 | 0.00 | 0.03 | 0.02 | 0.01 | 0.01 | 0.03 | 0.02 | 0.01 | 0.01 |
| γ*_g_*_8×E_ | γ*_b_*_46_ | 0.2 | 0.00 | 0.00 | 0.00 | 0.00 | 0.03 | 0.02 | 0.01 | 0.01 | 0.03 | 0.02 | 0.01 | 0.01 |
| γ*_g_*_9×E_ | γ*_b_*_46_ | -0.4 | 0.00 | 0.00 | 0.00 | 0.00 | 0.03 | 0.02 | 0.01 | 0.01 | 0.03 | 0.02 | 0.01 | 0.01 |
| γ*_g_*_1_ | γ*_b_*_47_ | 0 | 0.00 | 0.00 | 0.00 | 0.00 | 0.03 | 0.02 | 0.01 | 0.01 | 0.03 | 0.02 | 0.01 | 0.01 |
| γ*_g_*_2_ | γ*_b_*_47_ | 0 | 0.00 | 0.00 | 0.00 | 0.00 | 0.03 | 0.02 | 0.01 | 0.01 | 0.03 | 0.02 | 0.01 | 0.01 |
| γ*_g_*_3_ | γ*_b_*_47_ | 0 | 0.00 | 0.00 | 0.00 | 0.00 | 0.03 | 0.02 | 0.01 | 0.01 | 0.03 | 0.02 | 0.01 | 0.01 |
| γ*_g_*_4_ | γ*_b_*_47_ | 0 | -0.01 | 0.00 | 0.00 | 0.00 | 0.05 | 0.03 | 0.02 | 0.02 | 0.05 | 0.03 | 0.02 | 0.02 |
| γ*_g_*_5_ | γ*_b_*_47_ | 0 | 0.05 | 0.02 | 0.01 | 0.01 | 0.04 | 0.05 | 0.04 | 0.04 | 0.06 | 0.05 | 0.05 | 0.04 |
| γ*_g_*_6_ | γ*_b_*_47_ | 0 | -0.04 | -0.02 | -0.01 | -0.01 | 0.05 | 0.04 | 0.04 | 0.03 | 0.06 | 0.05 | 0.04 | 0.03 |
| γ*_g_*_7_ | γ*_b_*_47_ | 0.4 | 0.01 | 0.00 | 0.00 | 0.00 | 0.04 | 0.03 | 0.02 | 0.02 | 0.04 | 0.03 | 0.02 | 0.02 |
| γ*_g_*_8_ | γ*_b_*_47_ | 0 | 0.00 | 0.00 | 0.00 | 0.00 | 0.04 | 0.03 | 0.02 | 0.02 | 0.04 | 0.03 | 0.02 | 0.02 |
| γ*_g_*_9_ | γ*_b_*_47_ | -0.6 | 0.00 | 0.00 | 0.00 | 0.00 | 0.05 | 0.03 | 0.02 | 0.02 | 0.05 | 0.03 | 0.02 | 0.02 |
| z_E_ | γ*_b_*_47_ | 0 | 0.00 | 0.00 | 0.00 | 0.00 | 0.04 | 0.03 | 0.02 | 0.02 | 0.04 | 0.03 | 0.02 | 0.02 |
| γ*_g_*_1×E_ | γ*_b_*_47_ | 0 | 0.00 | 0.00 | 0.00 | 0.00 | 0.05 | 0.03 | 0.02 | 0.02 | 0.05 | 0.03 | 0.02 | 0.02 |
| γ*_g_*_2×E_ | γ*_b_*_47_ | 0 | 0.00 | 0.00 | 0.00 | 0.00 | 0.05 | 0.03 | 0.02 | 0.02 | 0.05 | 0.03 | 0.02 | 0.02 |
| γ*_g_*_3×E_ | γ*_b_*_47_ | 0 | 0.00 | 0.00 | 0.00 | 0.00 | 0.05 | 0.04 | 0.02 | 0.02 | 0.05 | 0.04 | 0.02 | 0.02 |
| γ*_g_*_4×E_ | γ*_b_*_47_ | 0 | 0.00 | 0.00 | 0.00 | 0.00 | 0.05 | 0.03 | 0.02 | 0.02 | 0.05 | 0.03 | 0.02 | 0.02 |
| γ*_g_*_5×E_ | γ*_b_*_47_ | 0 | -0.02 | -0.01 | -0.01 | 0.00 | 0.05 | 0.04 | 0.03 | 0.02 | 0.05 | 0.04 | 0.03 | 0.02 |
| γ*_g_*_6×E_ | γ*_b_*_47_ | 0 | 0.03 | 0.02 | 0.01 | 0.00 | 0.05 | 0.04 | 0.04 | 0.03 | 0.06 | 0.05 | 0.04 | 0.03 |
| γ*_g_*_7×E_ | γ*_b_*_47_ | 0.2 | -0.01 | 0.00 | -0.01 | 0.00 | 0.05 | 0.03 | 0.02 | 0.02 | 0.05 | 0.03 | 0.03 | 0.02 |
| γ*_g_*_8×E_ | γ*_b_*_47_ | 0 | 0.00 | 0.00 | 0.00 | 0.00 | 0.05 | 0.03 | 0.02 | 0.02 | 0.05 | 0.03 | 0.02 | 0.02 |
| γ*_g_*_9×E_ | γ*_b_*_47_ | 0 | 0.00 | 0.00 | 0.00 | 0.00 | 0.05 | 0.03 | 0.02 | 0.02 | 0.05 | 0.03 | 0.02 | 0.02 |
| γ*_g_*_1_ | γ*_b_*_48_ | 0 | 0.00 | 0.00 | 0.00 | 0.00 | 0.05 | 0.03 | 0.02 | 0.02 | 0.05 | 0.03 | 0.02 | 0.02 |
| γ*_g_*_2_ | γ*_b_*_48_ | 0 | 0.00 | 0.00 | 0.00 | 0.00 | 0.05 | 0.04 | 0.02 | 0.02 | 0.05 | 0.04 | 0.02 | 0.02 |
| γ*_g_*_3_ | γ*_b_*_48_ | 0 | 0.00 | 0.00 | 0.00 | 0.00 | 0.05 | 0.03 | 0.02 | 0.02 | 0.05 | 0.03 | 0.02 | 0.02 |
| γ*_g_*_4_ | γ*_b_*_48_ | 0 | -0.04 | -0.02 | -0.01 | -0.01 | 0.05 | 0.04 | 0.03 | 0.03 | 0.06 | 0.04 | 0.03 | 0.03 |
| γ*_g_*_5_ | γ*_b_*_48_ | 0 | 0.00 | 0.00 | 0.00 | 0.00 | 0.05 | 0.04 | 0.03 | 0.02 | 0.05 | 0.04 | 0.03 | 0.02 |
| γ*_g_*_6_ | γ*_b_*_48_ | 0 | 0.05 | 0.02 | 0.01 | 0.01 | 0.04 | 0.03 | 0.02 | 0.04 | 0.06 | 0.04 | 0.02 | 0.04 |
| γ*_g_*_7_ | γ*_b_*_48_ | -0.6 | 0.00 | 0.00 | 0.00 | 0.00 | 0.05 | 0.04 | 0.03 | 0.02 | 0.05 | 0.04 | 0.03 | 0.02 |
| γ*_g_*_8_ | γ*_b_*_48_ | 0 | 0.00 | 0.00 | 0.00 | 0.00 | 0.05 | 0.04 | 0.03 | 0.02 | 0.05 | 0.04 | 0.03 | 0.02 |
| γ*_g_*_9_ | γ*_b_*_48_ | 0 | 0.01 | 0.01 | 0.01 | 0.00 | 0.05 | 0.04 | 0.03 | 0.02 | 0.05 | 0.04 | 0.03 | 0.02 |
| z_E_ | γ*_b_*_48_ | 0.2 | 0.00 | 0.00 | 0.00 | 0.00 | 0.05 | 0.04 | 0.03 | 0.02 | 0.05 | 0.04 | 0.03 | 0.02 |
| γ*_g_*_1×E_ | γ*_b_*_48_ | 0 | 0.00 | 0.00 | 0.00 | 0.00 | 0.05 | 0.04 | 0.03 | 0.02 | 0.05 | 0.04 | 0.03 | 0.02 |
| γ*_g_*_2×E_ | γ*_b_*_48_ | 0 | 0.00 | 0.00 | 0.00 | 0.00 | 0.05 | 0.04 | 0.03 | 0.02 | 0.05 | 0.04 | 0.03 | 0.02 |
| γ*_g_*_3×E_ | γ*_b_*_48_ | 0 | 0.00 | 0.00 | 0.00 | 0.00 | 0.05 | 0.03 | 0.03 | 0.02 | 0.05 | 0.03 | 0.03 | 0.02 |
| γ*_g_*_4×E_ | γ*_b_*_48_ | 0 | -0.02 | -0.01 | -0.01 | -0.01 | 0.05 | 0.04 | 0.03 | 0.02 | 0.05 | 0.04 | 0.03 | 0.02 |
| γ*_g_*_5×E_ | γ*_b_*_48_ | 0 | 0.01 | 0.00 | 0.00 | 0.00 | 0.05 | 0.04 | 0.03 | 0.02 | 0.05 | 0.04 | 0.03 | 0.02 |
| γ*_g_*_6×E_ | γ*_b_*_48_ | 0 | 0.00 | 0.00 | 0.00 | 0.00 | 0.05 | 0.04 | 0.03 | 0.02 | 0.05 | 0.04 | 0.03 | 0.02 |
| γ*_g_*_7×E_ | γ*_b_*_48_ | 0.4 | 0.01 | 0.01 | 0.01 | 0.01 | 0.05 | 0.04 | 0.03 | 0.02 | 0.05 | 0.04 | 0.03 | 0.02 |
| γ*_g_*_8×E_ | γ*_b_*_48_ | 0 | 0.00 | 0.00 | 0.00 | 0.00 | 0.05 | 0.04 | 0.03 | 0.02 | 0.05 | 0.04 | 0.03 | 0.02 |
| γ*_g_*_9×E_ | γ*_b_*_48_ | 0 | 0.00 | 0.00 | 0.00 | 0.00 | 0.05 | 0.04 | 0.03 | 0.02 | 0.05 | 0.04 | 0.03 | 0.02 |
| γ*_g_*_1_ | γ*_b_*_49_ | 0 | 0.00 | 0.00 | 0.00 | 0.00 | 0.05 | 0.04 | 0.03 | 0.02 | 0.05 | 0.04 | 0.03 | 0.02 |
| γ*_g_*_2_ | γ*_b_*_49_ | 0 | 0.00 | 0.01 | 0.00 | 0.00 | 0.05 | 0.04 | 0.03 | 0.02 | 0.05 | 0.04 | 0.03 | 0.02 |
| γ*_g_*_3_ | γ*_b_*_49_ | 0 | 0.00 | 0.00 | 0.00 | 0.00 | 0.05 | 0.04 | 0.03 | 0.02 | 0.05 | 0.04 | 0.03 | 0.02 |
| γ*_g_*_4_ | γ*_b_*_49_ | 0 | 0.04 | 0.02 | 0.01 | 0.00 | 0.04 | 0.05 | 0.04 | 0.04 | 0.06 | 0.05 | 0.04 | 0.04 |
| γ*_g_*_5_ | γ*_b_*_49_ | 0 | -0.01 | -0.01 | 0.00 | 0.00 | 0.04 | 0.03 | 0.02 | 0.02 | 0.04 | 0.03 | 0.02 | 0.02 |
| γ*_g_*_6_ | γ*_b_*_49_ | 0 | -0.01 | 0.00 | 0.00 | 0.00 | 0.04 | 0.03 | 0.02 | 0.02 | 0.05 | 0.03 | 0.02 | 0.02 |
| γ*_g_*_7_ | γ*_b_*_49_ | 0.6 | 0.00 | 0.00 | 0.00 | 0.00 | 0.04 | 0.03 | 0.02 | 0.02 | 0.04 | 0.03 | 0.02 | 0.02 |
| γ*_g_*_8_ | γ*_b_*_49_ | 0.4 | 0.00 | 0.00 | 0.00 | 0.00 | 0.04 | 0.03 | 0.02 | 0.02 | 0.04 | 0.03 | 0.02 | 0.02 |
| γ*_g_*_9_ | γ*_b_*_49_ | 0 | 0.00 | 0.00 | 0.00 | 0.00 | 0.04 | 0.03 | 0.02 | 0.02 | 0.04 | 0.03 | 0.02 | 0.02 |
| z_E_ | γ*_b_*_49_ | 0.2 | 0.00 | 0.00 | 0.00 | 0.00 | 0.04 | 0.03 | 0.02 | 0.02 | 0.04 | 0.03 | 0.02 | 0.02 |
| γ*_g_*_1×E_ | γ*_b_*_49_ | 0 | 0.00 | 0.00 | 0.00 | 0.00 | 0.04 | 0.03 | 0.02 | 0.02 | 0.04 | 0.03 | 0.02 | 0.02 |
| γ*_g_*_2×E_ | γ*_b_*_49_ | 0 | -0.01 | -0.01 | -0.01 | -0.01 | 0.04 | 0.03 | 0.02 | 0.02 | 0.04 | 0.03 | 0.02 | 0.02 |
| γ*_g_*_3×E_ | γ*_b_*_49_ | 0 | -0.02 | -0.01 | -0.01 | -0.01 | 0.05 | 0.04 | 0.03 | 0.02 | 0.05 | 0.04 | 0.03 | 0.02 |
| γ*_g_*_4×E_ | γ*_b_*_49_ | 0 | -0.03 | -0.02 | -0.01 | -0.01 | 0.05 | 0.04 | 0.03 | 0.03 | 0.05 | 0.04 | 0.04 | 0.03 |
| γ*_g_*_5×E_ | γ*_b_*_49_ | 0 | 0.01 | 0.00 | 0.00 | 0.00 | 0.05 | 0.03 | 0.02 | 0.02 | 0.05 | 0.03 | 0.02 | 0.02 |
| γ*_g_*_6×E_ | γ*_b_*_49_ | 0 | 0.00 | 0.00 | 0.00 | 0.00 | 0.05 | 0.03 | 0.02 | 0.02 | 0.05 | 0.03 | 0.02 | 0.02 |
| γ*_g_*_7×E_ | γ*_b_*_49_ | -0.4 | 0.00 | 0.00 | 0.00 | 0.00 | 0.04 | 0.03 | 0.02 | 0.02 | 0.04 | 0.03 | 0.02 | 0.02 |
| γ*_g_*_8×E_ | γ*_b_*_49_ | 0 | 0.01 | 0.01 | 0.01 | 0.01 | 0.05 | 0.03 | 0.02 | 0.02 | 0.05 | 0.03 | 0.02 | 0.02 |
| γ*_g_*_9×E_ | γ*_b_*_49_ | 0 | 0.00 | 0.00 | 0.00 | 0.00 | 0.05 | 0.03 | 0.02 | 0.02 | 0.05 | 0.03 | 0.02 | 0.02 |
| γ*_g_*_1_ | γ*_b_*_50_ | 0 | 0.00 | 0.00 | 0.00 | 0.00 | 0.05 | 0.03 | 0.02 | 0.02 | 0.05 | 0.03 | 0.02 | 0.02 |
| γ*_g_*_2_ | γ*_b_*_50_ | 0 | 0.00 | 0.00 | 0.00 | 0.00 | 0.04 | 0.03 | 0.02 | 0.02 | 0.04 | 0.03 | 0.02 | 0.02 |
| γ*_g_*_3_ | γ*_b_*_50_ | 0 | 0.00 | 0.00 | 0.00 | 0.00 | 0.05 | 0.03 | 0.02 | 0.02 | 0.05 | 0.03 | 0.02 | 0.02 |
| γ*_g_*_4_ | γ*_b_*_50_ | 0 | -0.04 | -0.02 | -0.01 | 0.00 | 0.03 | 0.02 | 0.02 | 0.01 | 0.05 | 0.03 | 0.02 | 0.01 |
| γ*_g_*_5_ | γ*_b_*_50_ | 0 | -0.02 | -0.01 | 0.00 | 0.00 | 0.03 | 0.02 | 0.02 | 0.01 | 0.03 | 0.02 | 0.02 | 0.01 |
| γ*_g_*_6_ | γ*_b_*_50_ | 0 | 0.02 | 0.01 | 0.00 | 0.00 | 0.03 | 0.02 | 0.01 | 0.01 | 0.03 | 0.02 | 0.01 | 0.01 |
| γ*_g_*_7_ | γ*_b_*_50_ | 0 | 0.00 | 0.00 | 0.00 | 0.00 | 0.03 | 0.02 | 0.01 | 0.01 | 0.03 | 0.02 | 0.01 | 0.01 |
| γ*_g_*_8_ | γ*_b_*_50_ | -0.6 | 0.00 | 0.00 | 0.00 | 0.00 | 0.03 | 0.02 | 0.01 | 0.01 | 0.03 | 0.02 | 0.01 | 0.01 |
| γ*_g_*_9_ | γ*_b_*_50_ | 0.4 | 0.00 | 0.00 | 0.00 | 0.00 | 0.03 | 0.02 | 0.01 | 0.01 | 0.03 | 0.02 | 0.01 | 0.01 |
| z_E_ | γ*_b_*_50_ | 0 | 0.00 | 0.00 | 0.00 | 0.00 | 0.03 | 0.02 | 0.01 | 0.01 | 0.03 | 0.02 | 0.01 | 0.01 |
| γ*_g_*_1×E_ | γ*_b_*_50_ | 0 | 0.00 | 0.00 | 0.00 | 0.00 | 0.03 | 0.02 | 0.01 | 0.01 | 0.03 | 0.02 | 0.01 | 0.01 |
| γ*_g_*_2×E_ | γ*_b_*_50_ | 0 | 0.00 | 0.00 | 0.00 | 0.00 | 0.03 | 0.02 | 0.01 | 0.01 | 0.03 | 0.02 | 0.01 | 0.01 |
| γ*_g_*_3×E_ | γ*_b_*_50_ | 0 | -0.02 | -0.01 | -0.01 | 0.00 | 0.03 | 0.02 | 0.02 | 0.01 | 0.04 | 0.03 | 0.02 | 0.01 |
| γ*_g_*_4×E_ | γ*_b_*_50_ | 0 | 0.03 | 0.02 | 0.01 | 0.01 | 0.03 | 0.02 | 0.02 | 0.01 | 0.04 | 0.03 | 0.02 | 0.01 |
| γ*_g_*_5×E_ | γ*_b_*_50_ | 0 | -0.01 | 0.00 | 0.00 | 0.00 | 0.03 | 0.02 | 0.01 | 0.01 | 0.03 | 0.02 | 0.01 | 0.01 |
| γ*_g_*_6×E_ | γ*_b_*_50_ | 0 | -0.01 | 0.00 | 0.00 | 0.00 | 0.03 | 0.02 | 0.01 | 0.01 | 0.03 | 0.02 | 0.01 | 0.01 |
| γ*_g_*_7×E_ | γ*_b_*_50_ | 0 | 0.00 | 0.00 | 0.00 | 0.00 | 0.03 | 0.02 | 0.01 | 0.01 | 0.03 | 0.02 | 0.01 | 0.01 |
| γ*_g_*_8×E_ | γ*_b_*_50_ | 0.2 | 0.00 | 0.00 | 0.00 | 0.00 | 0.03 | 0.02 | 0.01 | 0.01 | 0.03 | 0.02 | 0.01 | 0.01 |
| γ*_g_*_9×E_ | γ*_b_*_50_ | -0.4 | 0.00 | 0.00 | 0.00 | 0.00 | 0.03 | 0.02 | 0.01 | 0.01 | 0.03 | 0.02 | 0.01 | 0.01 |
| γ*_g_*_1_ | γ*_b_*_51_ | 0 | 0.00 | 0.00 | 0.00 | 0.00 | 0.03 | 0.02 | 0.01 | 0.01 | 0.03 | 0.02 | 0.01 | 0.01 |
| γ*_g_*_2_ | γ*_b_*_51_ | 0 | 0.00 | 0.00 | 0.00 | 0.00 | 0.03 | 0.02 | 0.01 | 0.01 | 0.03 | 0.02 | 0.01 | 0.01 |
| γ*_g_*_3_ | γ*_b_*_51_ | 0 | 0.00 | 0.00 | 0.00 | 0.00 | 0.03 | 0.02 | 0.01 | 0.01 | 0.03 | 0.02 | 0.01 | 0.01 |
| γ*_g_*_4_ | γ*_b_*_51_ | 0 | 0.00 | 0.00 | 0.00 | 0.00 | 0.05 | 0.03 | 0.02 | 0.02 | 0.05 | 0.03 | 0.02 | 0.02 |
| γ*_g_*_5_ | γ*_b_*_51_ | 0 | 0.05 | 0.02 | 0.01 | 0.00 | 0.07 | 0.05 | 0.02 | 0.02 | 0.09 | 0.05 | 0.02 | 0.02 |
| γ*_g_*_6_ | γ*_b_*_51_ | 0 | -0.04 | -0.02 | -0.01 | -0.01 | 0.06 | 0.04 | 0.03 | 0.02 | 0.07 | 0.05 | 0.03 | 0.02 |
| γ*_g_*_7_ | γ*_b_*_51_ | 0.4 | 0.00 | 0.00 | -0.01 | 0.00 | 0.05 | 0.03 | 0.02 | 0.02 | 0.05 | 0.03 | 0.02 | 0.02 |
| γ*_g_*_8_ | γ*_b_*_51_ | 0 | 0.00 | 0.00 | 0.00 | 0.00 | 0.05 | 0.03 | 0.03 | 0.02 | 0.05 | 0.03 | 0.03 | 0.02 |
| γ*_g_*_9_ | γ*_b_*_51_ | -0.6 | 0.00 | 0.00 | 0.00 | 0.00 | 0.05 | 0.03 | 0.02 | 0.02 | 0.05 | 0.03 | 0.02 | 0.02 |
| z_E_ | γ*_b_*_51_ | 0 | 0.00 | 0.00 | 0.00 | 0.00 | 0.04 | 0.03 | 0.02 | 0.02 | 0.04 | 0.03 | 0.02 | 0.02 |
| γ*_g_*_1×E_ | γ*_b_*_51_ | 0 | 0.00 | 0.00 | 0.00 | 0.00 | 0.05 | 0.03 | 0.02 | 0.02 | 0.05 | 0.03 | 0.02 | 0.02 |
| γ*_g_*_2×E_ | γ*_b_*_51_ | 0 | 0.00 | 0.00 | 0.00 | 0.00 | 0.05 | 0.04 | 0.02 | 0.02 | 0.05 | 0.04 | 0.02 | 0.02 |
| γ*_g_*_3×E_ | γ*_b_*_51_ | 0 | 0.00 | 0.00 | 0.00 | 0.00 | 0.05 | 0.04 | 0.02 | 0.02 | 0.05 | 0.04 | 0.03 | 0.02 |
| γ*_g_*_4×E_ | γ*_b_*_51_ | 0 | 0.00 | 0.00 | 0.00 | 0.01 | 0.05 | 0.04 | 0.02 | 0.02 | 0.05 | 0.04 | 0.02 | 0.02 |
| γ*_g_*_5×E_ | γ*_b_*_51_ | 0 | -0.03 | -0.01 | -0.01 | 0.00 | 0.05 | 0.04 | 0.02 | 0.02 | 0.06 | 0.04 | 0.02 | 0.02 |
| γ*_g_*_6×E_ | γ*_b_*_51_ | 0 | 0.04 | 0.02 | 0.01 | 0.01 | 0.06 | 0.04 | 0.03 | 0.02 | 0.07 | 0.05 | 0.03 | 0.02 |
| γ*_g_*_7×E_ | γ*_b_*_51_ | 0.2 | 0.00 | 0.00 | 0.00 | 0.00 | 0.05 | 0.03 | 0.02 | 0.02 | 0.05 | 0.03 | 0.02 | 0.02 |
| γ*_g_*_8×E_ | γ*_b_*_51_ | 0 | 0.00 | 0.00 | 0.00 | 0.00 | 0.05 | 0.03 | 0.02 | 0.02 | 0.05 | 0.03 | 0.02 | 0.02 |
| γ*_g_*_9×E_ | γ*_b_*_51_ | 0 | 0.00 | 0.00 | 0.00 | 0.00 | 0.05 | 0.03 | 0.02 | 0.02 | 0.05 | 0.03 | 0.02 | 0.02 |
| γ*_g_*_1_ | γ*_b_*_52_ | 0 | 0.00 | 0.00 | 0.00 | 0.00 | 0.05 | 0.03 | 0.02 | 0.02 | 0.05 | 0.03 | 0.02 | 0.02 |
| γ*_g_*_2_ | γ*_b_*_52_ | 0 | 0.00 | 0.00 | 0.00 | 0.00 | 0.05 | 0.03 | 0.02 | 0.02 | 0.05 | 0.03 | 0.02 | 0.02 |
| γ*_g_*_3_ | γ*_b_*_52_ | 0 | 0.00 | 0.00 | 0.00 | 0.00 | 0.05 | 0.03 | 0.02 | 0.02 | 0.05 | 0.03 | 0.02 | 0.02 |
| γ*_g_*_4_ | γ*_b_*_52_ | 0 | -0.04 | -0.02 | -0.01 | -0.01 | 0.05 | 0.04 | 0.02 | 0.02 | 0.06 | 0.04 | 0.03 | 0.02 |
| γ*_g_*_5_ | γ*_b_*_52_ | 0 | -0.01 | 0.00 | 0.00 | 0.00 | 0.05 | 0.04 | 0.03 | 0.02 | 0.05 | 0.04 | 0.03 | 0.02 |
| γ*_g_*_6_ | γ*_b_*_52_ | 0 | 0.05 | 0.03 | 0.02 | 0.01 | 0.04 | 0.03 | 0.02 | 0.02 | 0.07 | 0.04 | 0.03 | 0.02 |
| γ*_g_*_7_ | γ*_b_*_52_ | -0.6 | 0.00 | 0.00 | 0.00 | 0.00 | 0.05 | 0.04 | 0.03 | 0.02 | 0.05 | 0.04 | 0.03 | 0.02 |
| γ*_g_*_8_ | γ*_b_*_52_ | 0 | -0.01 | 0.00 | 0.00 | 0.00 | 0.05 | 0.04 | 0.03 | 0.02 | 0.05 | 0.04 | 0.03 | 0.02 |
| γ*_g_*_9_ | γ*_b_*_52_ | 0 | -0.01 | 0.00 | 0.00 | 0.00 | 0.05 | 0.04 | 0.03 | 0.02 | 0.05 | 0.04 | 0.03 | 0.02 |
| z_E_ | γ*_b_*_52_ | 0.2 | 0.00 | 0.00 | 0.00 | 0.01 | 0.05 | 0.04 | 0.03 | 0.02 | 0.05 | 0.04 | 0.03 | 0.02 |
| γ*_g_*_1×E_ | γ*_b_*_52_ | 0 | 0.00 | 0.00 | 0.00 | 0.00 | 0.05 | 0.04 | 0.03 | 0.02 | 0.05 | 0.04 | 0.03 | 0.02 |
| γ*_g_*_2×E_ | γ*_b_*_52_ | 0 | 0.00 | 0.00 | 0.00 | 0.00 | 0.05 | 0.04 | 0.03 | 0.02 | 0.05 | 0.04 | 0.03 | 0.02 |
| γ*_g_*_3×E_ | γ*_b_*_52_ | 0 | 0.00 | 0.00 | 0.00 | 0.00 | 0.05 | 0.04 | 0.03 | 0.02 | 0.05 | 0.04 | 0.03 | 0.02 |
| γ*_g_*_4×E_ | γ*_b_*_52_ | 0 | -0.02 | -0.01 | 0.00 | 0.00 | 0.05 | 0.04 | 0.03 | 0.02 | 0.05 | 0.04 | 0.03 | 0.02 |
| γ*_g_*_5×E_ | γ*_b_*_52_ | 0 | 0.01 | 0.00 | 0.00 | 0.00 | 0.05 | 0.04 | 0.03 | 0.02 | 0.05 | 0.04 | 0.03 | 0.02 |
| γ*_g_*_6×E_ | γ*_b_*_52_ | 0 | 0.00 | 0.00 | 0.00 | 0.00 | 0.05 | 0.04 | 0.03 | 0.02 | 0.05 | 0.04 | 0.03 | 0.02 |
| γ*_g_*_7×E_ | γ*_b_*_52_ | 0.4 | 0.01 | 0.00 | 0.01 | 0.00 | 0.05 | 0.04 | 0.03 | 0.02 | 0.05 | 0.04 | 0.03 | 0.02 |
| γ*_g_*_8×E_ | γ*_b_*_52_ | 0 | 0.00 | 0.00 | 0.00 | 0.00 | 0.05 | 0.04 | 0.03 | 0.02 | 0.05 | 0.04 | 0.03 | 0.02 |
| γ*_g_*_9×E_ | γ*_b_*_52_ | 0 | 0.00 | 0.00 | 0.00 | 0.00 | 0.05 | 0.04 | 0.03 | 0.02 | 0.05 | 0.04 | 0.03 | 0.02 |
| γ*_g_*_1_ | γ*_b_*_53_ | 0 | 0.00 | 0.00 | 0.00 | 0.00 | 0.05 | 0.04 | 0.03 | 0.02 | 0.05 | 0.04 | 0.03 | 0.02 |
| γ*_g_*_2_ | γ*_b_*_53_ | 0 | 0.00 | 0.00 | 0.00 | 0.00 | 0.05 | 0.04 | 0.03 | 0.02 | 0.05 | 0.04 | 0.03 | 0.02 |
| γ*_g_*_3_ | γ*_b_*_53_ | 0 | 0.00 | 0.00 | 0.00 | 0.00 | 0.05 | 0.04 | 0.03 | 0.02 | 0.05 | 0.04 | 0.03 | 0.02 |
| γ*_g_*_4_ | γ*_b_*_53_ | 0 | 0.04 | 0.02 | 0.01 | 0.01 | 0.04 | 0.03 | 0.02 | 0.04 | 0.06 | 0.04 | 0.02 | 0.04 |
| γ*_g_*_5_ | γ*_b_*_53_ | 0 | -0.01 | 0.00 | 0.00 | 0.00 | 0.04 | 0.03 | 0.02 | 0.02 | 0.05 | 0.03 | 0.02 | 0.02 |
| γ*_g_*_6_ | γ*_b_*_53_ | 0 | -0.01 | 0.00 | 0.00 | 0.00 | 0.05 | 0.03 | 0.02 | 0.02 | 0.05 | 0.03 | 0.02 | 0.02 |
| γ*_g_*_7_ | γ*_b_*_53_ | 0.6 | 0.00 | 0.00 | 0.00 | 0.00 | 0.04 | 0.03 | 0.02 | 0.02 | 0.04 | 0.03 | 0.02 | 0.02 |
| γ*_g_*_8_ | γ*_b_*_53_ | 0.4 | 0.00 | 0.00 | 0.00 | 0.00 | 0.04 | 0.03 | 0.02 | 0.02 | 0.04 | 0.03 | 0.02 | 0.02 |
| γ*_g_*_9_ | γ*_b_*_53_ | 0 | 0.00 | 0.00 | 0.00 | 0.00 | 0.04 | 0.03 | 0.02 | 0.02 | 0.05 | 0.03 | 0.02 | 0.02 |
| z_E_ | γ*_b_*_53_ | 0.2 | 0.00 | 0.00 | 0.00 | 0.00 | 0.04 | 0.03 | 0.02 | 0.02 | 0.04 | 0.03 | 0.02 | 0.02 |
| γ*_g_*_1×E_ | γ*_b_*_53_ | 0 | 0.00 | 0.00 | 0.00 | 0.00 | 0.04 | 0.03 | 0.02 | 0.02 | 0.04 | 0.03 | 0.02 | 0.02 |
| γ*_g_*_2×E_ | γ*_b_*_53_ | 0 | 0.00 | 0.00 | 0.00 | 0.00 | 0.04 | 0.03 | 0.02 | 0.02 | 0.04 | 0.03 | 0.02 | 0.02 |
| γ*_g_*_3×E_ | γ*_b_*_53_ | 0 | -0.01 | -0.01 | 0.00 | 0.00 | 0.05 | 0.03 | 0.03 | 0.02 | 0.05 | 0.03 | 0.03 | 0.02 |
| γ*_g_*_4×E_ | γ*_b_*_53_ | 0 | -0.04 | -0.02 | -0.01 | -0.01 | 0.03 | 0.02 | 0.04 | 0.01 | 0.05 | 0.03 | 0.04 | 0.01 |
| γ*_g_*_5×E_ | γ*_b_*_53_ | 0 | -0.02 | -0.01 | 0.00 | 0.00 | 0.03 | 0.02 | 0.03 | 0.01 | 0.04 | 0.02 | 0.03 | 0.01 |
| γ*_g_*_6×E_ | γ*_b_*_53_ | 0 | 0.01 | 0.01 | 0.00 | 0.00 | 0.03 | 0.02 | 0.01 | 0.01 | 0.03 | 0.02 | 0.01 | 0.01 |
| γ*_g_*_7×E_ | γ*_b_*_53_ | -0.4 | 0.00 | 0.00 | 0.00 | 0.00 | 0.03 | 0.02 | 0.01 | 0.01 | 0.03 | 0.02 | 0.01 | 0.01 |
| γ*_g_*_8×E_ | γ*_b_*_53_ | 0 | 0.00 | 0.00 | 0.00 | 0.00 | 0.03 | 0.02 | 0.01 | 0.01 | 0.03 | 0.02 | 0.01 | 0.01 |
| γ*_g_*_9×E_ | γ*_b_*_53_ | 0 | 0.00 | 0.00 | 0.00 | 0.00 | 0.03 | 0.02 | 0.01 | 0.01 | 0.03 | 0.02 | 0.01 | 0.01 |
| γ*_g_*_1_ | γ*_b_*_54_ | 0 | 0.00 | 0.00 | 0.00 | 0.00 | 0.03 | 0.02 | 0.01 | 0.01 | 0.03 | 0.02 | 0.01 | 0.01 |
| γ*_g_*_2_ | γ*_b_*_54_ | 0 | 0.00 | 0.00 | 0.00 | 0.00 | 0.03 | 0.02 | 0.01 | 0.01 | 0.03 | 0.02 | 0.01 | 0.01 |
| γ*_g_*_3_ | γ*_b_*_54_ | 0 |  | 0.00 | 0.00 | 0.00 | 0.03 | 0.02 | 0.01 | 0.01 | 0.03 | 0.02 | 0.01 | 0.01 |
| γ*_g_*_4_ | γ*_b_*_54_ | 0 | -0.02 | -0.01 | -0.01 | 0.00 | 0.03 | 0.02 | 0.02 | 0.01 | 0.04 | 0.03 | 0.02 | 0.01 |
| γ*_g_*_5_ | γ*_b_*_54_ | 0 | 0.03 | 0.01 | 0.01 | 0.00 | 0.03 | 0.02 | 0.03 | 0.01 | 0.04 | 0.03 | 0.03 | 0.01 |
| γ*_g_*_6_ | γ*_b_*_54_ | 0 | -0.01 | 0.00 | 0.00 | 0.00 | 0.03 | 0.02 | 0.01 | 0.01 | 0.03 | 0.02 | 0.01 | 0.01 |
| γ*_g_*_7_ | γ*_b_*_54_ | 0 | -0.01 | 0.00 | 0.00 | 0.00 | 0.03 | 0.02 | 0.01 | 0.01 | 0.03 | 0.02 | 0.01 | 0.01 |
| γ*_g_*_8_ | γ*_b_*_54_ | -0.6 | 0.00 | 0.00 | 0.00 | 0.00 | 0.03 | 0.02 | 0.01 | 0.01 | 0.03 | 0.02 | 0.01 | 0.01 |
| γ*_g_*_9_ | γ*_b_*_54_ | 0.4 | 0.00 | 0.00 | 0.00 | 0.00 | 0.03 | 0.02 | 0.01 | 0.01 | 0.03 | 0.02 | 0.01 | 0.01 |
| z_E_ | γ*_b_*_54_ | 0 | 0.00 | 0.00 | 0.00 | 0.00 | 0.03 | 0.02 | 0.01 | 0.01 | 0.03 | 0.02 | 0.01 | 0.01 |
| γ*_g_*_1×E_ | γ*_b_*_54_ | 0 | 0.00 | 0.00 | 0.00 | 0.00 | 0.03 | 0.02 | 0.01 | 0.01 | 0.03 | 0.02 | 0.01 | 0.01 |
| γ*_g_*_2×E_ | γ*_b_*_54_ | 0 | 0.00 | 0.00 | 0.00 | 0.00 | 0.03 | 0.02 | 0.01 | 0.01 | 0.03 | 0.02 | 0.01 | 0.01 |
| γ*_g_*_3×E_ | γ*_b_*_54_ | 0 | 0.00 | 0.00 | 0.00 | 0.00 | 0.03 | 0.02 | 0.01 | 0.01 | 0.03 | 0.02 | 0.01 | 0.01 |
| γ*_g_*_4×E_ | γ*_b_*_54_ | 0 | -0.01 | 0.00 | 0.00 | 0.00 | 0.05 | 0.03 | 0.02 | 0.02 | 0.05 | 0.03 | 0.02 | 0.02 |
| γ*_g_*_5×E_ | γ*_b_*_54_ | 0 | 0.05 | 0.02 | 0.01 | 0.01 | 0.04 | 0.05 | 0.04 | 0.04 | 0.06 | 0.05 | 0.05 | 0.04 |
| γ*_g_*_6×E_ | γ*_b_*_54_ | 0 | -0.04 | -0.02 | -0.01 | -0.01 | 0.05 | 0.04 | 0.04 | 0.03 | 0.06 | 0.05 | 0.04 | 0.03 |
| γ*_g_*_7×E_ | γ*_b_*_54_ | 0 | 0.01 | 0.00 | 0.00 | 0.00 | 0.04 | 0.03 | 0.02 | 0.02 | 0.04 | 0.03 | 0.02 | 0.02 |
| γ*_g_*_8×E_ | γ*_b_*_54_ | 0.2 | 0.00 | 0.00 | 0.00 | 0.00 | 0.04 | 0.03 | 0.02 | 0.02 | 0.04 | 0.03 | 0.02 | 0.02 |
| γ*_g_*_9×E_ | γ*_b_*_54_ | -0.4 | 0.00 | 0.00 | 0.00 | 0.00 | 0.05 | 0.03 | 0.02 | 0.02 | 0.05 | 0.03 | 0.02 | 0.02 |
| γ*_g_*_1_ | γ*_b_*_55_ | 0 | 0.00 | 0.00 | 0.00 | 0.00 | 0.04 | 0.03 | 0.02 | 0.02 | 0.04 | 0.03 | 0.02 | 0.02 |
| γ*_g_*_2_ | γ*_b_*_55_ | 0 | 0.00 | 0.00 | 0.00 | 0.00 | 0.05 | 0.03 | 0.02 | 0.02 | 0.05 | 0.03 | 0.02 | 0.02 |
| γ*_g_*_3_ | γ*_b_*_55_ | 0 | 0.00 | 0.00 | 0.00 | 0.00 | 0.05 | 0.03 | 0.02 | 0.02 | 0.05 | 0.03 | 0.02 | 0.02 |
| γ*_g_*_4_ | γ*_b_*_55_ | 0 | 0.00 | 0.00 | 0.00 | 0.00 | 0.05 | 0.04 | 0.02 | 0.02 | 0.05 | 0.04 | 0.02 | 0.02 |
| γ*_g_*_5_ | γ*_b_*_55_ | 0 | 0.00 | 0.00 | 0.00 | 0.00 | 0.05 | 0.03 | 0.02 | 0.02 | 0.05 | 0.03 | 0.02 | 0.02 |
| γ*_g_*_6_ | γ*_b_*_55_ | 0 | -0.02 | -0.01 | -0.01 | 0.00 | 0.05 | 0.04 | 0.03 | 0.02 | 0.05 | 0.04 | 0.03 | 0.02 |
| γ*_g_*_7_ | γ*_b_*_55_ | 0.4 | 0.03 | 0.02 | 0.01 | 0.00 | 0.05 | 0.04 | 0.04 | 0.03 | 0.06 | 0.05 | 0.04 | 0.03 |
| γ*_g_*_8_ | γ*_b_*_55_ | 0 | -0.01 | 0.00 | -0.01 | 0.00 | 0.05 | 0.03 | 0.02 | 0.02 | 0.05 | 0.03 | 0.03 | 0.02 |
| γ*_g_*_9_ | γ*_b_*_55_ | -0.6 | 0.00 | 0.00 | 0.00 | 0.00 | 0.05 | 0.03 | 0.02 | 0.02 | 0.05 | 0.03 | 0.02 | 0.02 |
| z_E_ | γ*_b_*_55_ | 0 | 0.00 | 0.00 | 0.00 | 0.00 | 0.05 | 0.03 | 0.02 | 0.02 | 0.05 | 0.03 | 0.02 | 0.02 |
| γ*_g_*_1×E_ | γ*_b_*_55_ | 0 | 0.00 | 0.00 | 0.00 | 0.00 | 0.05 | 0.03 | 0.02 | 0.02 | 0.05 | 0.03 | 0.02 | 0.02 |
| γ*_g_*_2×E_ | γ*_b_*_55_ | 0 | 0.00 | 0.00 | 0.00 | 0.00 | 0.05 | 0.04 | 0.02 | 0.02 | 0.05 | 0.04 | 0.02 | 0.02 |
| γ*_g_*_3×E_ | γ*_b_*_55_ | 0 | 0.00 | 0.00 | 0.00 | 0.00 | 0.05 | 0.03 | 0.02 | 0.02 | 0.05 | 0.03 | 0.02 | 0.02 |
| γ*_g_*_4×E_ | γ*_b_*_55_ | 0 | -0.04 | -0.02 | -0.01 | -0.01 | 0.05 | 0.04 | 0.03 | 0.03 | 0.06 | 0.04 | 0.03 | 0.03 |
| γ*_g_*_5×E_ | γ*_b_*_55_ | 0 | 0.00 | 0.00 | 0.00 | 0.00 | 0.05 | 0.04 | 0.03 | 0.02 | 0.05 | 0.04 | 0.03 | 0.02 |
| γ*_g_*_6×E_ | γ*_b_*_55_ | 0 | 0.05 | 0.02 | 0.01 | 0.01 | 0.04 | 0.03 | 0.02 | 0.04 | 0.06 | 0.04 | 0.02 | 0.04 |
| γ*_g_*_7×E_ | γ*_b_*_55_ | 0.2 | 0.00 | 0.00 | 0.00 | 0.00 | 0.05 | 0.04 | 0.03 | 0.02 | 0.05 | 0.04 | 0.03 | 0.02 |
| γ*_g_*_8×E_ | γ*_b_*_55_ | 0 | 0.00 | 0.00 | 0.00 | 0.00 | 0.05 | 0.04 | 0.03 | 0.02 | 0.05 | 0.04 | 0.03 | 0.02 |
| γ*_g_*_9×E_ | γ*_b_*_55_ | 0 | 0.01 | 0.01 | 0.01 | 0.00 | 0.05 | 0.04 | 0.03 | 0.02 | 0.05 | 0.04 | 0.03 | 0.02 |
| γ*_g_*_1_ | γ*_b_*_56_ | 0 | 0.00 | 0.00 | 0.00 | 0.00 | 0.05 | 0.04 | 0.03 | 0.02 | 0.05 | 0.04 | 0.03 | 0.02 |
| γ*_g_*_2_ | γ*_b_*_56_ | 0 | 0.00 | 0.00 | 0.00 | 0.00 | 0.05 | 0.04 | 0.03 | 0.02 | 0.05 | 0.04 | 0.03 | 0.02 |
| γ*_g_*_3_ | γ*_b_*_56_ | 0 | 0.00 | 0.00 | 0.00 | 0.00 | 0.05 | 0.04 | 0.03 | 0.02 | 0.05 | 0.04 | 0.03 | 0.02 |
| γ*_g_*_4_ | γ*_b_*_56_ | 0 | 0.00 | 0.00 | 0.00 | 0.00 | 0.05 | 0.03 | 0.03 | 0.02 | 0.05 | 0.03 | 0.03 | 0.02 |
| γ*_g_*_5_ | γ*_b_*_56_ | 0 | -0.02 | -0.01 | -0.01 | -0.01 | 0.05 | 0.04 | 0.03 | 0.02 | 0.05 | 0.04 | 0.03 | 0.02 |
| γ*_g_*_6_ | γ*_b_*_56_ | 0 | 0.01 | 0.00 | 0.00 | 0.00 | 0.05 | 0.04 | 0.03 | 0.02 | 0.05 | 0.04 | 0.03 | 0.02 |
| γ*_g_*_7_ | γ*_b_*_56_ | -0.6 | 0.00 | 0.00 | 0.00 | 0.00 | 0.05 | 0.04 | 0.03 | 0.02 | 0.05 | 0.04 | 0.03 | 0.02 |
| γ*_g_*_8_ | γ*_b_*_56_ | 0 | 0.01 | 0.01 | 0.01 | 0.01 | 0.05 | 0.04 | 0.03 | 0.02 | 0.05 | 0.04 | 0.03 | 0.02 |
| γ*_g_*_9_ | γ*_b_*_56_ | 0 | 0.00 | 0.00 | 0.00 | 0.00 | 0.05 | 0.04 | 0.03 | 0.02 | 0.05 | 0.04 | 0.03 | 0.02 |
| z_E_ | γ*_b_*_56_ | 0.2 | 0.00 | 0.00 | 0.00 | 0.00 | 0.05 | 0.04 | 0.03 | 0.02 | 0.05 | 0.04 | 0.03 | 0.02 |
| γ*_g_*_1×E_ | γ*_b_*_56_ | 0 | 0.00 | 0.00 | 0.00 | 0.00 | 0.05 | 0.04 | 0.03 | 0.02 | 0.05 | 0.04 | 0.03 | 0.02 |
| γ*_g_*_2×E_ | γ*_b_*_56_ | 0 | 0.00 | 0.01 | 0.00 | 0.00 | 0.05 | 0.04 | 0.03 | 0.02 | 0.05 | 0.04 | 0.03 | 0.02 |
| γ*_g_*_3×E_ | γ*_b_*_56_ | 0 | 0.00 | 0.00 | 0.00 | 0.00 | 0.05 | 0.04 | 0.03 | 0.02 | 0.05 | 0.04 | 0.03 | 0.02 |
| γ*_g_*_4×E_ | γ*_b_*_56_ | 0 | 0.04 | 0.02 | 0.01 | 0.00 | 0.04 | 0.05 | 0.04 | 0.04 | 0.06 | 0.05 | 0.04 | 0.04 |
| γ*_g_*_5×E_ | γ*_b_*_56_ | 0 | -0.01 | -0.01 | 0.00 | 0.00 | 0.04 | 0.03 | 0.02 | 0.02 | 0.04 | 0.03 | 0.02 | 0.02 |
| γ*_g_*_6×E_ | γ*_b_*_56_ | 0 | -0.01 | 0.00 | 0.00 | 0.00 | 0.04 | 0.03 | 0.02 | 0.02 | 0.05 | 0.03 | 0.02 | 0.02 |
| γ*_g_*_7×E_ | γ*_b_*_56_ | 0.4 | 0.00 | 0.00 | 0.00 | 0.00 | 0.04 | 0.03 | 0.02 | 0.02 | 0.04 | 0.03 | 0.02 | 0.02 |
| γ*_g_*_8×E_ | γ*_b_*_56_ | 0 | 0.00 | 0.00 | 0.00 | 0.00 | 0.04 | 0.03 | 0.02 | 0.02 | 0.04 | 0.03 | 0.02 | 0.02 |
| γ*_g_*_9×E_ | γ*_b_*_56_ | 0 | 0.00 | 0.00 | 0.00 | 0.00 | 0.04 | 0.03 | 0.02 | 0.02 | 0.04 | 0.03 | 0.02 | 0.02 |
| γ*_g_*_1_ | γ*_b_*_57_ | 0 | 0.00 | 0.00 | 0.00 | 0.00 | 0.04 | 0.03 | 0.02 | 0.02 | 0.04 | 0.03 | 0.02 | 0.02 |
| γ*_g_*_2_ | γ*_b_*_57_ | 0 | 0.00 | 0.00 | 0.00 | 0.00 | 0.04 | 0.03 | 0.02 | 0.02 | 0.04 | 0.03 | 0.02 | 0.02 |
| γ*_g_*_3_ | γ*_b_*_57_ | 0 | -0.01 | -0.01 | -0.01 | -0.01 | 0.04 | 0.03 | 0.02 | 0.02 | 0.04 | 0.03 | 0.02 | 0.02 |
| γ*_g_*_4_ | γ*_b_*_57_ | 0 | -0.02 | -0.01 | -0.01 | -0.01 | 0.05 | 0.04 | 0.03 | 0.02 | 0.05 | 0.04 | 0.03 | 0.02 |
| γ*_g_*_5_ | γ*_b_*_57_ | 0 | -0.03 | -0.02 | -0.01 | -0.01 | 0.05 | 0.04 | 0.03 | 0.03 | 0.05 | 0.04 | 0.04 | 0.03 |
| γ*_g_*_6_ | γ*_b_*_57_ | 0 | 0.01 | 0.00 | 0.00 | 0.00 | 0.05 | 0.03 | 0.02 | 0.02 | 0.05 | 0.03 | 0.02 | 0.02 |
| γ*_g_*_7_ | γ*_b_*_57_ | 0.6 | 0.00 | 0.00 | 0.00 | 0.00 | 0.05 | 0.03 | 0.02 | 0.02 | 0.05 | 0.03 | 0.02 | 0.02 |
| γ*_g_*_8_ | γ*_b_*_57_ | 0.4 | 0.00 | 0.00 | 0.00 | 0.00 | 0.04 | 0.03 | 0.02 | 0.02 | 0.04 | 0.03 | 0.02 | 0.02 |
| γ*_g_*_9_ | γ*_b_*_57_ | 0 | 0.01 | 0.01 | 0.01 | 0.01 | 0.05 | 0.03 | 0.02 | 0.02 | 0.05 | 0.03 | 0.02 | 0.02 |
| z_E_ | γ*_b_*_57_ | 0.2 | 0.00 | 0.00 | 0.00 | 0.00 | 0.05 | 0.03 | 0.02 | 0.02 | 0.05 | 0.03 | 0.02 | 0.02 |
| γ*_g_*_1×E_ | γ*_b_*_57_ | 0 | 0.00 | 0.00 | 0.00 | 0.00 | 0.05 | 0.03 | 0.02 | 0.02 | 0.05 | 0.03 | 0.02 | 0.02 |
| γ*_g_*_2×E_ | γ*_b_*_57_ | 0 | 0.00 | 0.00 | 0.00 | 0.00 | 0.04 | 0.03 | 0.02 | 0.02 | 0.04 | 0.03 | 0.02 | 0.02 |
| γ*_g_*_3×E_ | γ*_b_*_57_ | 0 | 0.00 | 0.00 | 0.00 | 0.00 | 0.05 | 0.03 | 0.02 | 0.02 | 0.05 | 0.03 | 0.02 | 0.02 |
| γ*_g_*_4×E_ | γ*_b_*_57_ | 0 | -0.04 | -0.02 | -0.01 | 0.00 | 0.03 | 0.02 | 0.02 | 0.01 | 0.05 | 0.03 | 0.02 | 0.01 |
| γ*_g_*_5×E_ | γ*_b_*_57_ | 0 | -0.02 | -0.01 | 0.00 | 0.00 | 0.03 | 0.02 | 0.02 | 0.01 | 0.03 | 0.02 | 0.02 | 0.01 |
| γ*_g_*_6×E_ | γ*_b_*_57_ | 0 | 0.02 | 0.01 | 0.00 | 0.00 | 0.03 | 0.02 | 0.01 | 0.01 | 0.03 | 0.02 | 0.01 | 0.01 |
| γ*_g_*_7×E_ | γ*_b_*_57_ | -0.4 | 0.00 | 0.00 | 0.00 | 0.00 | 0.03 | 0.02 | 0.01 | 0.01 | 0.03 | 0.02 | 0.01 | 0.01 |
| γ*_g_*_8×E_ | γ*_b_*_57_ | 0 | 0.00 | 0.00 | 0.00 | 0.00 | 0.03 | 0.02 | 0.01 | 0.01 | 0.03 | 0.02 | 0.01 | 0.01 |
| γ*_g_*_9×E_ | γ*_b_*_57_ | 0 | 0.00 | 0.00 | 0.00 | 0.00 | 0.03 | 0.02 | 0.01 | 0.01 | 0.03 | 0.02 | 0.01 | 0.01 |
| γ*_g_*_1_ | γ*_b_*_58_ | 0 | 0.00 | 0.00 | 0.00 | 0.00 | 0.03 | 0.02 | 0.01 | 0.01 | 0.03 | 0.02 | 0.01 | 0.01 |
| γ*_g_*_2_ | γ*_b_*_58_ | 0 | 0.00 | 0.00 | 0.00 | 0.00 | 0.03 | 0.02 | 0.01 | 0.01 | 0.03 | 0.02 | 0.01 | 0.01 |
| γ*_g_*_3_ | γ*_b_*_58_ | 0 | 0.00 | 0.00 | 0.00 | 0.00 | 0.03 | 0.02 | 0.01 | 0.01 | 0.03 | 0.02 | 0.01 | 0.01 |
| γ*_g_*_4_ | γ*_b_*_58_ | 0 | -0.02 | -0.01 | -0.01 | 0.00 | 0.03 | 0.02 | 0.02 | 0.01 | 0.04 | 0.03 | 0.02 | 0.01 |
| γ*_g_*_5_ | γ*_b_*_58_ | 0 | 0.03 | 0.02 | 0.01 | 0.01 | 0.03 | 0.02 | 0.02 | 0.01 | 0.04 | 0.03 | 0.02 | 0.01 |
| γ*_g_*_6_ | γ*_b_*_58_ | 0 | -0.01 | 0.00 | 0.00 | 0.00 | 0.03 | 0.02 | 0.01 | 0.01 | 0.03 | 0.02 | 0.01 | 0.01 |
| γ*_g_*_7_ | γ*_b_*_58_ | 0 | -0.01 | 0.00 | 0.00 | 0.00 | 0.03 | 0.02 | 0.01 | 0.01 | 0.03 | 0.02 | 0.01 | 0.01 |
| γ*_g_*_8_ | γ*_b_*_58_ | -0.6 | 0.00 | 0.00 | 0.00 | 0.00 | 0.03 | 0.02 | 0.01 | 0.01 | 0.03 | 0.02 | 0.01 | 0.01 |
| γ*_g_*_9_ | γ*_b_*_58_ | 0.4 | 0.00 | 0.00 | 0.00 | 0.00 | 0.03 | 0.02 | 0.01 | 0.01 | 0.03 | 0.02 | 0.01 | 0.01 |
| z_E_ | γ*_b_*_58_ | 0 | 0.00 | 0.00 | 0.00 | 0.00 | 0.03 | 0.02 | 0.01 | 0.01 | 0.03 | 0.02 | 0.01 | 0.01 |
| γ*_g_*_1×E_ | γ*_b_*_58_ | 0 | 0.00 | 0.00 | 0.00 | 0.00 | 0.03 | 0.02 | 0.01 | 0.01 | 0.03 | 0.02 | 0.01 | 0.01 |
| γ*_g_*_2×E_ | γ*_b_*_58_ | 0 | 0.00 | 0.00 | 0.00 | 0.00 | 0.03 | 0.02 | 0.01 | 0.01 | 0.03 | 0.02 | 0.01 | 0.01 |
| γ*_g_*_3×E_ | γ*_b_*_58_ | 0 | 0.00 | 0.00 | 0.00 | 0.00 | 0.03 | 0.02 | 0.01 | 0.01 | 0.03 | 0.02 | 0.01 | 0.01 |
| γ*_g_*_4×E_ | γ*_b_*_58_ | 0 | 0.00 | 0.00 | 0.00 | 0.00 | 0.05 | 0.03 | 0.02 | 0.02 | 0.05 | 0.03 | 0.02 | 0.02 |
| γ*_g_*_5×E_ | γ*_b_*_58_ | 0 | 0.05 | 0.02 | 0.01 | 0.00 | 0.07 | 0.05 | 0.02 | 0.02 | 0.09 | 0.05 | 0.02 | 0.02 |
| γ*_g_*_6×E_ | γ*_b_*_58_ | 0 | -0.04 | -0.02 | -0.01 | -0.01 | 0.06 | 0.04 | 0.03 | 0.02 | 0.07 | 0.05 | 0.03 | 0.02 |
| γ*_g_*_7×E_ | γ*_b_*_58_ | 0 | 0.00 | 0.00 | -0.01 | 0.00 | 0.05 | 0.03 | 0.02 | 0.02 | 0.05 | 0.03 | 0.02 | 0.02 |
| γ*_g_*_8×E_ | γ*_b_*_58_ | 0.2 | 0.00 | 0.00 | 0.00 | 0.00 | 0.05 | 0.03 | 0.03 | 0.02 | 0.05 | 0.03 | 0.03 | 0.02 |
| γ*_g_*_9×E_ | γ*_b_*_58_ | -0.4 | 0.00 | 0.00 | 0.00 | 0.00 | 0.05 | 0.03 | 0.02 | 0.02 | 0.05 | 0.03 | 0.02 | 0.02 |
| γ*_g_*_1_ | γ*_b_*_59_ | 0 | 0.00 | 0.00 | 0.00 | 0.00 | 0.04 | 0.03 | 0.02 | 0.02 | 0.04 | 0.03 | 0.02 | 0.02 |
| γ*_g_*_2_ | γ*_b_*_59_ | 0 | 0.00 | 0.00 | 0.00 | 0.00 | 0.05 | 0.03 | 0.02 | 0.02 | 0.05 | 0.03 | 0.02 | 0.02 |
| γ*_g_*_3_ | γ*_b_*_59_ | 0 | 0.00 | 0.00 | 0.00 | 0.00 | 0.05 | 0.04 | 0.02 | 0.02 | 0.05 | 0.04 | 0.02 | 0.02 |
| γ*_g_*_4_ | γ*_b_*_59_ | 0 | 0.00 | 0.00 | 0.00 | 0.00 | 0.05 | 0.04 | 0.02 | 0.02 | 0.05 | 0.04 | 0.03 | 0.02 |
| γ*_g_*_5_ | γ*_b_*_59_ | 0 | 0.00 | 0.00 | 0.00 | 0.01 | 0.05 | 0.04 | 0.02 | 0.02 | 0.05 | 0.04 | 0.02 | 0.02 |
| γ*_g_*_6_ | γ*_b_*_59_ | 0 | -0.03 | -0.01 | -0.01 | 0.00 | 0.05 | 0.04 | 0.02 | 0.02 | 0.06 | 0.04 | 0.02 | 0.02 |
| γ*_g_*_7_ | γ*_b_*_59_ | 0.4 | 0.04 | 0.02 | 0.01 | 0.01 | 0.06 | 0.04 | 0.03 | 0.02 | 0.07 | 0.05 | 0.03 | 0.02 |
| γ*_g_*_8_ | γ*_b_*_59_ | 0 | 0.00 | 0.00 | 0.00 | 0.00 | 0.05 | 0.03 | 0.02 | 0.02 | 0.05 | 0.03 | 0.02 | 0.02 |
| γ*_g_*_9_ | γ*_b_*_59_ | -0.6 | 0.00 | 0.00 | 0.00 | 0.00 | 0.05 | 0.03 | 0.02 | 0.02 | 0.05 | 0.03 | 0.02 | 0.02 |
| z_E_ | γ*_b_*_59_ | 0 | 0.00 | 0.00 | 0.00 | 0.00 | 0.05 | 0.03 | 0.02 | 0.02 | 0.05 | 0.03 | 0.02 | 0.02 |
| γ*_g_*_1×E_ | γ*_b_*_59_ | 0 | 0.00 | 0.00 | 0.00 | 0.00 | 0.05 | 0.03 | 0.02 | 0.02 | 0.05 | 0.03 | 0.02 | 0.02 |
| γ*_g_*_2×E_ | γ*_b_*_59_ | 0 | 0.00 | 0.00 | 0.00 | 0.00 | 0.05 | 0.03 | 0.02 | 0.02 | 0.05 | 0.03 | 0.02 | 0.02 |
| γ*_g_*_3×E_ | γ*_b_*_59_ | 0 | 0.00 | 0.00 | 0.00 | 0.00 | 0.05 | 0.03 | 0.02 | 0.02 | 0.05 | 0.03 | 0.02 | 0.02 |
| γ*_g_*_4×E_ | γ*_b_*_59_ | 0 | -0.04 | -0.02 | -0.01 | -0.01 | 0.05 | 0.04 | 0.02 | 0.02 | 0.06 | 0.04 | 0.03 | 0.02 |
| γ*_g_*_5×E_ | γ*_b_*_59_ | 0 | -0.01 | 0.00 | 0.00 | 0.00 | 0.05 | 0.04 | 0.03 | 0.02 | 0.05 | 0.04 | 0.03 | 0.02 |
| γ*_g_*_6×E_ | γ*_b_*_59_ | 0 | 0.05 | 0.03 | 0.02 | 0.01 | 0.04 | 0.03 | 0.02 | 0.02 | 0.07 | 0.04 | 0.03 | 0.02 |
| γ*_g_*_7×E_ | γ*_b_*_59_ | 0.2 | 0.00 | 0.00 | 0.00 | 0.00 | 0.05 | 0.04 | 0.03 | 0.02 | 0.05 | 0.04 | 0.03 | 0.02 |
| γ*_g_*_8×E_ | γ*_b_*_59_ | 0 | -0.01 | 0.00 | 0.00 | 0.00 | 0.05 | 0.04 | 0.03 | 0.02 | 0.05 | 0.04 | 0.03 | 0.02 |
| γ*_g_*_9×E_ | γ*_b_*_59_ | 0 | -0.01 | 0.00 | 0.00 | 0.00 | 0.05 | 0.04 | 0.03 | 0.02 | 0.05 | 0.04 | 0.03 | 0.02 |
| γ*_g_*_1_ | γ*_b_*_60_ | 0 | 0.00 | 0.00 | 0.00 | 0.01 | 0.05 | 0.04 | 0.03 | 0.02 | 0.05 | 0.04 | 0.03 | 0.02 |
| γ*_g_*_2_ | γ*_b_*_60_ | 0 | 0.00 | 0.00 | 0.00 | 0.00 | 0.05 | 0.04 | 0.03 | 0.02 | 0.05 | 0.04 | 0.03 | 0.02 |
| γ*_g_*_3_ | γ*_b_*_60_ | 0 | 0.00 | 0.00 | 0.00 | 0.00 | 0.05 | 0.04 | 0.03 | 0.02 | 0.05 | 0.04 | 0.03 | 0.02 |
| γ*_g_*_4_ | γ*_b_*_60_ | 0 | 0.00 | 0.00 | 0.00 | 0.00 | 0.05 | 0.04 | 0.03 | 0.02 | 0.05 | 0.04 | 0.03 | 0.02 |
| γ*_g_*_5_ | γ*_b_*_60_ | 0 | -0.02 | -0.01 | 0.00 | 0.00 | 0.05 | 0.04 | 0.03 | 0.02 | 0.05 | 0.04 | 0.03 | 0.02 |
| γ*_g_*_6_ | γ*_b_*_60_ | 0 | 0.01 | 0.00 | 0.00 | 0.00 | 0.05 | 0.04 | 0.03 | 0.02 | 0.05 | 0.04 | 0.03 | 0.02 |
| γ*_g_*_7_ | γ*_b_*_60_ | -0.6 | 0.00 | 0.00 | 0.00 | 0.00 | 0.05 | 0.04 | 0.03 | 0.02 | 0.05 | 0.04 | 0.03 | 0.02 |
| γ*_g_*_8_ | γ*_b_*_60_ | 0 | 0.01 | 0.00 | 0.01 | 0.00 | 0.05 | 0.04 | 0.03 | 0.02 | 0.05 | 0.04 | 0.03 | 0.02 |
| γ*_g_*_9_ | γ*_b_*_60_ | 0 | 0.00 | 0.00 | 0.00 | 0.00 | 0.05 | 0.04 | 0.03 | 0.02 | 0.05 | 0.04 | 0.03 | 0.02 |
| z_E_ | γ*_b_*_60_ | 0.2 | 0.00 | 0.00 | 0.00 | 0.00 | 0.05 | 0.04 | 0.03 | 0.02 | 0.05 | 0.04 | 0.03 | 0.02 |
| γ*_g_*_1×E_ | γ*_b_*_60_ | 0 | 0.00 | 0.00 | 0.00 | 0.00 | 0.05 | 0.04 | 0.03 | 0.02 | 0.05 | 0.04 | 0.03 | 0.02 |
| γ*_g_*_2×E_ | γ*_b_*_60_ | 0 | 0.00 | 0.00 | 0.00 | 0.00 | 0.05 | 0.04 | 0.03 | 0.02 | 0.05 | 0.04 | 0.03 | 0.02 |
| γ*_g_*_3×E_ | γ*_b_*_60_ | 0 | 0.00 | 0.00 | 0.00 | 0.00 | 0.05 | 0.04 | 0.03 | 0.02 | 0.05 | 0.04 | 0.03 | 0.02 |
| γ*_g_*_4×E_ | γ*_b_*_60_ | 0 | 0.04 | 0.02 | 0.01 | 0.01 | 0.04 | 0.03 | 0.02 | 0.04 | 0.06 | 0.04 | 0.02 | 0.04 |
| γ*_g_*_5×E_ | γ*_b_*_60_ | 0 | -0.01 | 0.00 | 0.00 | 0.00 | 0.04 | 0.03 | 0.02 | 0.02 | 0.05 | 0.03 | 0.02 | 0.02 |
| γ*_g_*_6×E_ | γ*_b_*_60_ | 0 | -0.01 | 0.00 | 0.00 | 0.00 | 0.05 | 0.03 | 0.02 | 0.02 | 0.05 | 0.03 | 0.02 | 0.02 |
| γ*_g_*_7×E_ | γ*_b_*_60_ | 0.4 | 0.00 | 0.00 | 0.00 | 0.00 | 0.04 | 0.03 | 0.02 | 0.02 | 0.04 | 0.03 | 0.02 | 0.02 |
| γ*_g_*_8×E_ | γ*_b_*_60_ | 0 | 0.00 | 0.00 | 0.00 | 0.00 | 0.04 | 0.03 | 0.02 | 0.02 | 0.04 | 0.03 | 0.02 | 0.02 |
| γ*_g_*_9×E_ | γ*_b_*_60_ | 0 | 0.00 | 0.00 | 0.00 | 0.00 | 0.04 | 0.03 | 0.02 | 0.02 | 0.05 | 0.03 | 0.02 | 0.02 |
| z_E_ | z_O_ | 0.4 | 0.00 | 0.00 | 0.00 | 0.00 | 0.04 | 0.03 | 0.02 | 0.02 | 0.04 | 0.03 | 0.02 | 0.02 |
| γ*_b_*_1_ | z_O_ | 0.2 | 0.00 | 0.00 | 0.00 | 0.00 | 0.04 | 0.03 | 0.02 | 0.02 | 0.04 | 0.03 | 0.02 | 0.02 |
| γ*_b_*_2_ | z_O_ | 0 | 0.00 | 0.00 | 0.00 | 0.00 | 0.04 | 0.03 | 0.02 | 0.02 | 0.04 | 0.03 | 0.02 | 0.02 |
| γ*_b_*_3_ | z_O_ | 0.4 | -0.01 | -0.01 | 0.00 | 0.00 | 0.05 | 0.03 | 0.03 | 0.02 | 0.05 | 0.03 | 0.03 | 0.02 |
| γ*_b_*_4_ | z_O_ | 0.6 | -0.04 | -0.02 | -0.01 | -0.01 | 0.03 | 0.02 | 0.04 | 0.01 | 0.05 | 0.03 | 0.04 | 0.01 |
| γ*_b_*_5_ | z_O_ | 0 | -0.02 | -0.01 | 0.00 | 0.00 | 0.03 | 0.02 | 0.03 | 0.01 | 0.04 | 0.02 | 0.03 | 0.01 |
| γ*_b_*_6_ | z_O_ | 0 | 0.01 | 0.01 | 0.00 | 0.00 | 0.03 | 0.02 | 0.01 | 0.01 | 0.03 | 0.02 | 0.01 | 0.01 |
| γ*_b_*_7_ | z_O_ | 0 | 0.00 | 0.00 | 0.00 | 0.00 | 0.03 | 0.02 | 0.01 | 0.01 | 0.03 | 0.02 | 0.01 | 0.01 |
| γ*_b_*_8_ | z_O_ | 0 | 0.00 | 0.00 | 0.00 | 0.00 | 0.03 | 0.02 | 0.01 | 0.01 | 0.03 | 0.02 | 0.01 | 0.01 |
| γ*_b_*_9_ | z_O_ | 0 | 0.00 | 0.00 | 0.00 | 0.00 | 0.03 | 0.02 | 0.01 | 0.01 | 0.03 | 0.02 | 0.01 | 0.01 |
| γ*_b_*_10_ | z_O_ | 0 | 0.00 | 0.00 | 0.00 | 0.00 | 0.03 | 0.02 | 0.01 | 0.01 | 0.03 | 0.02 | 0.01 | 0.01 |
| γ*_b_*_11_ | z_O_ | 0 | 0.00 | 0.00 | 0.00 | 0.00 | 0.03 | 0.02 | 0.01 | 0.01 | 0.03 | 0.02 | 0.01 | 0.01 |
| γ*_b_*_12_ | z_O_ | 0 |  | 0.00 | 0.00 | 0.00 | 0.03 | 0.02 | 0.01 | 0.01 | 0.03 | 0.02 | 0.01 | 0.01 |
| γ*_b_*_13_ | z_O_ | 0 | -0.02 | -0.01 | -0.01 | 0.00 | 0.03 | 0.02 | 0.02 | 0.01 | 0.04 | 0.03 | 0.02 | 0.01 |
| γ*_b_*_14_ | z_O_ | 0 | 0.03 | 0.01 | 0.01 | 0.00 | 0.03 | 0.02 | 0.03 | 0.01 | 0.04 | 0.03 | 0.03 | 0.01 |
| γ*_b_*_15_ | z_O_ | 0 | -0.01 | 0.00 | 0.00 | 0.00 | 0.03 | 0.02 | 0.01 | 0.01 | 0.03 | 0.02 | 0.01 | 0.01 |
| γ*_b_*_16_ | z_O_ | 0 | -0.01 | 0.00 | 0.00 | 0.00 | 0.03 | 0.02 | 0.01 | 0.01 | 0.03 | 0.02 | 0.01 | 0.01 |
| γ*_b_*_17_ | z_O_ | 0 | 0.00 | 0.00 | 0.00 | 0.00 | 0.03 | 0.02 | 0.01 | 0.01 | 0.03 | 0.02 | 0.01 | 0.01 |
| γ*_b_*_18_ | z_O_ | 0 | 0.00 | 0.00 | 0.00 | 0.00 | 0.03 | 0.02 | 0.01 | 0.01 | 0.03 | 0.02 | 0.01 | 0.01 |
| γ*_b_*_19_ | z_O_ | 0 | 0.00 | 0.00 | 0.00 | 0.00 | 0.03 | 0.02 | 0.01 | 0.01 | 0.03 | 0.02 | 0.01 | 0.01 |
| γ*_b_*_20_ | z_O_ | 0 | 0.00 | 0.00 | 0.00 | 0.00 | 0.03 | 0.02 | 0.01 | 0.01 | 0.03 | 0.02 | 0.01 | 0.01 |
| γ*_b_*_21_ | z_O_ | 0 | 0.00 | 0.00 | 0.00 | 0.00 | 0.03 | 0.02 | 0.01 | 0.01 | 0.03 | 0.02 | 0.01 | 0.01 |
| γ*_b_*_22_ | z_O_ | 0 | 0.00 | 0.00 | 0.00 | 0.00 | 0.03 | 0.02 | 0.01 | 0.01 | 0.03 | 0.02 | 0.01 | 0.01 |
| γ*_b_*_23_ | z_O_ | 0 | -0.01 | 0.00 | 0.00 | 0.00 | 0.05 | 0.03 | 0.02 | 0.02 | 0.05 | 0.03 | 0.02 | 0.02 |
| γ*_b_*_24_ | z_O_ | 0 | 0.05 | 0.02 | 0.01 | 0.01 | 0.04 | 0.05 | 0.04 | 0.04 | 0.06 | 0.05 | 0.05 | 0.04 |
| γ*_b_*_25_ | z_O_ | 0 | -0.04 | -0.02 | -0.01 | -0.01 | 0.05 | 0.04 | 0.04 | 0.03 | 0.06 | 0.05 | 0.04 | 0.03 |
| γ*_b_*_26_ | z_O_ | 0 | 0.01 | 0.00 | 0.00 | 0.00 | 0.04 | 0.03 | 0.02 | 0.02 | 0.04 | 0.03 | 0.02 | 0.02 |
| γ*_b_*_27_ | z_O_ | 0 | 0.00 | 0.00 | 0.00 | 0.00 | 0.04 | 0.03 | 0.02 | 0.02 | 0.04 | 0.03 | 0.02 | 0.02 |
| γ*_b_*_28_ | z_O_ | 0 | 0.00 | 0.00 | 0.00 | 0.00 | 0.05 | 0.03 | 0.02 | 0.02 | 0.05 | 0.03 | 0.02 | 0.02 |
| γ*_b_*_29_ | z_O_ | 0 | 0.00 | 0.00 | 0.00 | 0.00 | 0.04 | 0.03 | 0.02 | 0.02 | 0.04 | 0.03 | 0.02 | 0.02 |
| γ*_b_*_30_ | z_O_ | 0 | 0.00 | 0.00 | 0.00 | 0.00 | 0.05 | 0.03 | 0.02 | 0.02 | 0.05 | 0.03 | 0.02 | 0.02 |
| γ*_b_*_31_ | z_O_ | 0 | 0.00 | 0.00 | 0.00 | 0.00 | 0.05 | 0.03 | 0.02 | 0.02 | 0.05 | 0.03 | 0.02 | 0.02 |
| γ*_b_*_32_ | z_O_ | 0 | 0.00 | 0.00 | 0.00 | 0.00 | 0.05 | 0.04 | 0.02 | 0.02 | 0.05 | 0.04 | 0.02 | 0.02 |
| γ*_b_*_33_ | z_O_ | 0 | 0.00 | 0.00 | 0.00 | 0.00 | 0.05 | 0.03 | 0.02 | 0.02 | 0.05 | 0.03 | 0.02 | 0.02 |
| γ*_b_*_34_ | z_O_ | 0 | -0.02 | -0.01 | -0.01 | 0.00 | 0.05 | 0.04 | 0.03 | 0.02 | 0.05 | 0.04 | 0.03 | 0.02 |
| γ*_b_*_35_ | z_O_ | 0 | 0.03 | 0.02 | 0.01 | 0.00 | 0.05 | 0.04 | 0.04 | 0.03 | 0.06 | 0.05 | 0.04 | 0.03 |
| γ*_b_*_36_ | z_O_ | 0 | -0.01 | 0.00 | -0.01 | 0.00 | 0.05 | 0.03 | 0.02 | 0.02 | 0.05 | 0.03 | 0.03 | 0.02 |
| γ*_b_*_37_ | z_O_ | 0 | 0.00 | 0.00 | 0.00 | 0.00 | 0.05 | 0.03 | 0.02 | 0.02 | 0.05 | 0.03 | 0.02 | 0.02 |
| γ*_b_*_38_ | z_O_ | 0 | 0.00 | 0.00 | 0.00 | 0.00 | 0.05 | 0.03 | 0.02 | 0.02 | 0.05 | 0.03 | 0.02 | 0.02 |
| γ*_b_*_39_ | z_O_ | 0 | 0.00 | 0.00 | 0.00 | 0.00 | 0.05 | 0.03 | 0.02 | 0.02 | 0.05 | 0.03 | 0.02 | 0.02 |
| γ*_b_*_40_ | z_O_ | 0 | 0.00 | 0.00 | 0.00 | 0.00 | 0.05 | 0.04 | 0.02 | 0.02 | 0.05 | 0.04 | 0.02 | 0.02 |
| γ*_b_*_41_ | z_O_ | 0 | 0.00 | 0.00 | 0.00 | 0.00 | 0.05 | 0.03 | 0.02 | 0.02 | 0.05 | 0.03 | 0.02 | 0.02 |
| γ*_b_*_42_ | z_O_ | 0 | -0.04 | -0.02 | -0.01 | -0.01 | 0.05 | 0.04 | 0.03 | 0.03 | 0.06 | 0.04 | 0.03 | 0.03 |
| γ*_b_*_43_ | z_O_ | 0 | 0.00 | 0.00 | 0.00 | 0.00 | 0.05 | 0.04 | 0.03 | 0.02 | 0.05 | 0.04 | 0.03 | 0.02 |
| γ*_b_*_44_ | z_O_ | 0 | 0.05 | 0.02 | 0.01 | 0.01 | 0.04 | 0.03 | 0.02 | 0.04 | 0.06 | 0.04 | 0.02 | 0.04 |
| γ*_b_*_45_ | z_O_ | 0 | 0.00 | 0.00 | 0.00 | 0.00 | 0.05 | 0.04 | 0.03 | 0.02 | 0.05 | 0.04 | 0.03 | 0.02 |
| γ*_b_*_46_ | z_O_ | 0 | 0.00 | 0.00 | 0.00 | 0.00 | 0.05 | 0.04 | 0.03 | 0.02 | 0.05 | 0.04 | 0.03 | 0.02 |
| γ*_b_*_47_ | z_O_ | 0 | 0.01 | 0.01 | 0.01 | 0.00 | 0.05 | 0.04 | 0.03 | 0.02 | 0.05 | 0.04 | 0.03 | 0.02 |
| γ*_b_*_48_ | z_O_ | 0 | 0.00 | 0.00 | 0.00 | 0.00 | 0.05 | 0.04 | 0.03 | 0.02 | 0.05 | 0.04 | 0.03 | 0.02 |
| γ*_b_*_49_ | z_O_ | 0 | 0.00 | 0.00 | 0.00 | 0.00 | 0.05 | 0.04 | 0.03 | 0.02 | 0.05 | 0.04 | 0.03 | 0.02 |
| γ*_b_*_50_ | z_O_ | 0 | 0.00 | 0.00 | 0.00 | 0.00 | 0.05 | 0.04 | 0.03 | 0.02 | 0.05 | 0.04 | 0.03 | 0.02 |
| γ*_b_*_51_ | z_O_ | 0 | 0.00 | 0.00 | 0.00 | 0.00 | 0.05 | 0.03 | 0.03 | 0.02 | 0.05 | 0.03 | 0.03 | 0.02 |
| γ*_b_*_52_ | z_O_ | 0 | -0.02 | -0.01 | -0.01 | -0.01 | 0.05 | 0.04 | 0.03 | 0.02 | 0.05 | 0.04 | 0.03 | 0.02 |
| γ*_b_*_53_ | z_O_ | 0 | 0.01 | 0.00 | 0.00 | 0.00 | 0.05 | 0.04 | 0.03 | 0.02 | 0.05 | 0.04 | 0.03 | 0.02 |
| γ*_b_*_54_ | z_O_ | 0 | 0.00 | 0.00 | 0.00 | 0.00 | 0.05 | 0.04 | 0.03 | 0.02 | 0.05 | 0.04 | 0.03 | 0.02 |
| γ*_b_*_55_ | z_O_ | 0 | 0.01 | 0.01 | 0.01 | 0.01 | 0.05 | 0.04 | 0.03 | 0.02 | 0.05 | 0.04 | 0.03 | 0.02 |
| γ*_b_*_56_ | z_O_ | 0 | 0.00 | 0.00 | 0.00 | 0.00 | 0.05 | 0.04 | 0.03 | 0.02 | 0.05 | 0.04 | 0.03 | 0.02 |
| γ*_b_*_57_ | z_O_ | 0 | 0.00 | 0.00 | 0.00 | 0.00 | 0.05 | 0.04 | 0.03 | 0.02 | 0.05 | 0.04 | 0.03 | 0.02 |
| γ*_b_*_58_ | z_O_ | 0 | 0.00 | 0.00 | 0.00 | 0.00 | 0.05 | 0.04 | 0.03 | 0.02 | 0.05 | 0.04 | 0.03 | 0.02 |
| γ*_b_*_59_ | z_O_ | 0 | 0.00 | 0.01 | 0.00 | 0.00 | 0.05 | 0.04 | 0.03 | 0.02 | 0.05 | 0.04 | 0.03 | 0.02 |
| γ*_b_*_60_ | z_O_ | 0 | 0.00 | 0.00 | 0.00 | 0.00 | 0.05 | 0.04 | 0.03 | 0.02 | 0.05 | 0.04 | 0.03 | 0.02 |
