# Supplementary material for: A knowledge-based multivariate statistical method for examining gene-brain-behavioral/cognitive relationships: Imaging genetics generalized structured component analysis
Source: PLoS One. 2021 Mar 10;16(3):e0247592. doi: 10.1371/journal.pone.0247592 (PMC7946325; doi:10.1371/journal.pone.0247592)
Supplement: S1 Appendix — (DOCX) [file pone.0247592.s003.docx]

S1 Appendix: The data generation procedure for the simulation study.

Let **z**_G_ = [z*_g_*_1_; **z***_g_*_2_; **z***_g_*_3_; z*_g_*_4_; **z***_g_*_5_; **z***_g_*_6_; z*_g_*_7_; **z***_g_*_8_; **z***_g_*_9_] denote a 21 by 1 vector of SNPs associated with nine genes, where z*_g_*_1_, z*_g_*_4_, and z*_g_*_7_ are scalar; **z***_g_*_2_, **z***_g_*_5_, and **z***_g_*_8_ are 2 by 1 vectors; and **z***_g_*_3_, **z***_g_*_6_, and **z***_g_*_9_ are 4 by 1 vectors. Let **Ω**_G_ denote a 21 by 21 covariance matrix of **z**_G_. Let **γ**_G_ = [γ*_g_*_1_; γ*_g_*_2_; ···; γ*_g_*_9_] denote a 9 by 1 vector of nine genes, where γ*_gq_* is a single gene (*q* = 1,···,9). Let **z**_B_ = [**z***_b_*_1_; ···; **z***_b_*_60_] denote a 120 by 1 vector of brain-level observed variables associated with 60 ROIs, where **z***_bl_* is a 2 by 1 vector (*l* = 1,···,60) and **Ω***_bl_* denote a 2 by 2 covariance matrix of **z***_bl_*. Let **γ**_B_ = [γ*_b_*_1_; γ*_b_*_2_; ···; γ*_b_*_60_] denote a vector of 60 ROIs, where γ*_bl_* is a single ROI (*l* = 1,···,60). Let z_E_ and z_O_ denote environmental and behavioral/cognitive observed variables, respectively. Let **γ**_X1_ = [**γ**_G_; z_E_; **γ**_GxE_] denote a vector of all independent variables for **γ**_B_, where **γ**_GxE_ = [γ*_g_*_1xE_; γ*_g_*_2xE_; ···; γ*_g_*_9xE_] is a 9 by 1 vector of nine gene-environment interaction terms. Let **Φ**_X1_ denote a 19 by 19 covariance matrix of **γ**_X1_. Let **γ**_X2_ = [z_E_; **γ**_B_] denote a vector of independent variables for z_O_. Let **Φ**_X2_ denote a 61 by 61 covariance matrix of **γ**_X2_. As in the real data analysis, each SNP in **z**_G_ was considered a nominal variable with three categories, whereas the other observed variables were continuous ones. All the observed and components were standardized.

Let **W**_G_ = diag(1,**W***_g_*_2_,···,**W***_g_*_9_) and **C**_G_ = diag(1,**C***_g_*_2_,···,**C***_g_*_9_) denote matrices of weights and loadings for **z**_G_, respectively. Let **W**_B_ = diag(**W***_b_*_1_,···, **W***_b_*_60_) and **C**_B_ = diag(**C***_b_*_1_,···, **C***_b_*_60_) denote matrices of weights and loadings for **z**_B_, respectively. Let **B**_B_ = [**B**_G_; **b**_E_; **B**_GxE_] denote a matrix of path coefficients connecting **γ**_X_ to **z**_B_, where **B**_G_ is a matrix of path coefficients relating **γ**_G_ to **γ**_B_, **b**_E_ is a vector of path coefficients relating γ_E_ to **γ**_B_, and **B**_GxE_ is a matrix of path coefficients relating **γ**_GxE_ to **γ**_B_. Let **b**_O_ denote a vector of path coefficients relating both z_E_ and **γ**_B_ to z_O_. Let **ε**_G_ and **ε**_B_ denote vectors of residual terms for **z**_G_ and **z**_B_, respectively, in the measurement model. Let **ζ**_B_ and ζ_O_ denote vectors of residual terms for **γ**_B_ and for z_O_, respectively, in the structural model. Let **Ψ***_l_* denote the covariance matrix of **ε***_bl_*. Let **Π**_B_ denote the block-diagonal covariance matrix of **ζ**_B_. Let σ denote the variance of ζ_O_. The model specified in the simulation study can be written as

**γ**_G_ = **W**_G_**z**_G_ (A1)

**γ**_B_ = **W**_B_**z**_B_ (A2)

**z**_G_ = **C**_G_**γ**_G_ + **ε**_G_ (A3)

**z**_B_ = **C**_B_**γ**_B_ + **ε**_B_ (A4)

**γ**_B_ = **B**_B_**γ**_X1_ + **ζ**_B_ (A5)

z_O_ = **b**_O_**γ**_X2_ + ζ_O_, (A6)

where cov(**γ**_G_, **ε**_G_) = **0**, cov(**γ**_B_, **ε**_B_) = **0**, cov(**γ**_X1_, **ζ**_B_) = **0**, and cov(**γ**_X2_, ζ_O_) = **0.**

For our study, we prescribed **Ω**_G_, **B**_B_, **Ω***_bl_*, and **b**_O_ as follows. As the specified model involved a large number of variables, we divided **z**_G_ into three groups, denoted by **z**_G_*^x^* (*x* = 1, 2, 3): **z**_G_^1^ = [z*_g_*_1_; **z***_g_*_2_; **z***_g_*_3_], **z**_G_^2^ = [z*_g_*_4_; **z***_g_*_5_; **z***_g_*_6_], and **z**_G_^3^ = [z*_g_*_7_; **z***_g_*_8_; **z***_g_*_9_]. Each of these groups contained 7 SNPs and was to form a group of three genes. The three-gene groups, denoted by **γ**_G_*^x^*, included **γ**_G_^1^ = [γ*_g_*_1_; γ*_g_*_2_; γ*_g_*_3_], **γ**_G_^2^ = [γ*_g_*_4_; γ*_g_*_5_; γ*_g_*_6_], and **γ**_G_^3^ = [γ*_g_*_7_; γ*_g_*_8_; γ*_g_*_9_]. Similarly, we divided the gene-environment interaction terms into three groups, denoted by **γ**_GxE_*^x^*, which included **γ**_GxE_^1^ = [γ*_g_*_1xE_; γ*_g_*_2xE_; γ*_g_*_3xE_], **γ**_GxE_^2^ = [γ*_g_*_4xE_; γ*_g_*_5xE_; γ*_g_*_6xE_], and **γ**_GxE_^3^ = [γ*_g_*_7xE_; γ*_g_*_8xE_; γ*_g_*_9xE_]. We divided **γ**_B_ into 15 groups of four ROIs each, denoted by **γ**_B_*^y^* (*y* = 1, ···, 15), i.e., **γ**_B_^1^ = [γ*_b_*_1_; γ*_b_*_2_; γ*_b_*_3_; γ*_b_*_4_], ···, **γ**_B_^15^ = [γ*_b_*_57_; γ*_b_*_58_; γ*_b_*_59_; γ*_b_*_60_]. Let **Ω**_g_ denote a 7 by 7 covariance matrix of **z**_G_*^x^*. We assumed that **Ω**_G_ = diag($\mathbf{1}_{3}\otimes\Omega_{g}$), where **1***_T_* is a column vector of *T* ones, and is the Kronecker product. Let **B**_g_ denote a 4 by 3 matrix of path coefficients from **γ**_G_*^x^* to **γ**_B_*^y^*. Let **b**_e_ denote a 4 by 1 matrix of path coefficients from z_E_ to **γ**_B_*^y^*. Let **B**_gxE_ denote a 4 by 3 matrix of path coefficients from **γ**_GxE_*^x^* to **γ**_B_*^y^*. We assumed that the first five of **γ**_B_*^y^* (**γ***_b_*^1^, **···**, **γ***_g_*^5^) were affected by **γ**_G_^1^, z_E_, and **γ**_GxE_^1^; the second five (**γ***_b_*^6^, **···**, **γ***_g_*^10^) by **γ**_G_^2^, z_E_, and **γ**_GxE_^2^; and the rest (**γ***_b_*^11^, **···**, **γ***_g_*^15^) by **γ**_G_^3^, z_E_, and **γ**_GxE_^3^. We also assumed that **B**_G_ = diag($\mathbf{1}_{15}\otimes\mathbf{B}_{g}$), **b**_E_ = $\mathbf{1}_{15}\otimes\mathbf{b}_{e}$, and **B**_GxE_ = diag($\mathbf{1}_{15}\otimes\mathbf{B}_{gxE}$). Then, we determined **Ω**_g_, **B**_g_, **b**_e_, and **B**_gxE_ as follows: **Ω**_g_ = $\left[\begin{matrix} 1 & .3 & .3 & .3 & .3 & .3 & .3 \\ & 1 & .6 & .3 & .3 & .3 & .3 \\ & & 1 & .3 & .3 & .3 & .3 \\ & & & 1 & .8 & .7 & .6 \\ & & & & 1 & .7 & .7 \\ & & & & & 1 & .6 \\ & & & & & & 1 \end{matrix}\right]$, **B**_g_ =$\left[\begin{matrix} .6 & .4 & 0 \\ 0 & -.6 & .4 \\ .4 & 0 & -.6 \\ -.6 & 0 & 0 \end{matrix}\right]$, **b**_e_ = [.2; 0; 0; .2], and **B**_gxE_ = $\left[\begin{matrix} -.4 & 0 & 0 \\ 0 & .2 & -.4 \\ .2 & 0 & 0 \\ .4 & 0 & 0 \end{matrix}\right]$. For the four pairs of brain-level observed variables associated with **γ**_B_*^y^*, denoted by **z***_by_*_(4_*_y_*_-3)_, **z***_by_*_(4_*_y_*_-2)_, **z***_by_*_(4_*_y_*_-1)_, and **z***_by_*_(4_*_y_*), we determined their covariance matrices as follows: **Ω***_b_*_(4_*_y_*_-3)_ =$\left[\begin{matrix} 1 & .5 \\ .5 & 1 \end{matrix}\right]$, **Ω***_b_*_(4_*_y_*_-2)_ =$\left[\begin{matrix} 1 & .6 \\ .6 & 1 \end{matrix}\right]$, **Ω***_b_*_(4_*_y_*_-1)_ =$\left[\begin{matrix} 1 & .7 \\ .7 & 1 \end{matrix}\right]$, and **Ω***_b_*_(4_*_y_*) =$\left[\begin{matrix} 1 & .8 \\ .8 & 1 \end{matrix}\right]$. Lastly, we prescribed **b**_O_ as [.4; .2; 0; .4; .6; **0**_54x1_].

We began by drawing a very large sample for **z**_G_ from a multivariate normal distribution with the zero mean vector and **Ω**_G_ (*N* = 100000) and discretized the sample scores of **z**_G_ into three values (i.e., 0, 1, and 2). We assigned 2 to the values greater than 1, 1 to those between 0 and 1, and 0 to those smaller than 0. We standardized the discretized scores and applied a categorical principal component analysis [101] to them for obtaining **C**_G_ and **W**_G_, and then for obtaining the scores of **γ**_G_ based on (A1). In genome-wide association studies, principal component analysis or its variants have been widely used to generate genetic data (e.g., Abegaz et al., 2018; Dai et al., 2013; Horne & Camp, 2004). We also drew a sample of the same size for z_E_ from the standard normal distribution and multiplied its scores by each gene’s scores to obtain the scores of **γ**_GxE_ and **γ**_X1_. Then, we obtained **Φ**_X1_ from **γ**_X_.

Given the prescribed values of **B**_B_ and *N* = 100000, we generated **ξ**_B_ from a multivariate normal distribution with zero means and the covariance matrix **Π**_B_, where **Π**_B_ was obtained from the diagonal elements of **I** – **B**_B_**Φ**_X_**B**_B_', and obtained the scores of **γ**_B_ from (A5). We then generated **z***_bl_* from a multivariate normal distribution with zero means and the covariance matrix **Ψ***_l_*, where **Ψ***_l_* = **Ω***_bl_* – **C***_bl_***C***_bl_*'. Lastly, we generated ζ_O_ from a normal distribution with zero mean and the variance σ, where σ = 1 – **b**_O_**Φ**_X2_**b**_O_', and obtained z_O_ from (A6). In this way, we obtained **z** = [**z**_G_; z_E_; **z**_B_; z_O_] and treated it as the population for the simulation study, from which smaller samples were randomly drawn.

**References**

101. Linting M, Meulman JJ, Groenen PJF, van der Koojj AJ. Nonlinear principal components analysis: Introduction and application. Psychological Methods. American Psychological Association; 2007. pp. 336–358. doi:10.1037/1082-989X.12.3.336

102. Horne BD, Camp NJ. Principal component analysis for selection of optimal SNP-sets that capture intragenic genetic variation. Genet Epidemiol. 2004;26: 11–21. doi:10.1002/gepi.10292

103. Abegaz F, Chaichoompu K, Génin E, Fardo DW, König IR, Mahachie John JM, et al. Principals about principal components in statistical genetics. Brief Bioinform. 2018;20: 2200–2216. doi:10.1093/bib/bby081

104. Dai H, Zhao Y, Qian C, Cai M, Zhang R, Chu M, et al. Weighted SNP set analysis in genome-wide association study. PLoS One. 2013;8: e75897. doi:10.1371/journal.pone.0075897
